# A Counterion‐Directed Approach to the Diels–Alder Paradigm: Cascade Synthesis of Tricyclic Fused Cyclopropanes

**DOI:** 10.1002/anie.201608534

**Published:** 2016-10-07

**Authors:** Emily Kiss, Craig D. Campbell, Russell W. Driver, John D. Jolliffe, Rosemary Lang, Tetiana Sergeieva, Sergiy Okovytyy, Robert S. Paton, Martin D. Smith

**Affiliations:** ^1^ Chemistry Research LaboratoryUniversity of Oxford12 Mansfield RoadOX1 3TAOxfordUK; ^2^ Department of ChemistryDnipropetrovsk National UniversityDnipropetrovsk49010Ukraine

**Keywords:** cations, cycloadditions, diastereoselectivity, density-functional calculations, heterocycles

## Abstract

An approach to the intramolecular Diels–Alder reaction has led to a cascade synthesis of complex carbocycles composed of three fused rings and up to five stereocenters with complete stereocontrol. Computational analysis reveals that the reaction proceeds by a Michael/Michael/cyclopropanation/epimerization cascade in which size and coordination of the counterion is key.

The intramolecular Diels–Alder reaction is a powerful tool for the synthesis of complex frameworks bearing numerous stereocenters from simple precursors.^1^ This process can be catalyzed by both Brønsted^2^ and Lewis acids,^3^ and secondary amines.^4^ Conceptually, these modes of activation rely upon reducing the energy gap between the highest filled molecular orbital (HOMO) of the diene and the lowest unoccupied molecular orbital (LUMO) of the dienophile through lowering of the LUMO. An alternative strategy to reducing the energetic gap relies on elevating the energy of the HOMO of the system, and this has been achieved through Brønsted base^5^ and secondary amine catalysis.^6^ We reasoned that a related process could lead to the in situ generation of a diene (**2**) by treatment of the vinyl ketone **1** under basic conditions and subsequent trapping by an alkene bearing a suitable electron‐withdrawing group. (Figure 1). In this process, the counterion has the potential to play a key role in directing both reactivity and stereoselectivity of the subsequent annulation process to give **3**. Herein we report that readily synthesized linear α‐bromo ester derivatives such as **4** and **5** can be selectively converted into complex fused tricyclic cyclopropanes **6** and **7**, respectively, in a process that forges three carbon–carbon bonds and up to five contiguous stereocenters with complete diastereocontrol. We also demonstrate that the diastereoselectivity of the reaction can be reversed through judicious choice of solvent and counterion. Our initial studies focused on the cyclization of a substrate in which the dienophile was substituted with two electron‐withdrawing groups. Substrate **8** was treated with tetrabutylammonium hydrogen sulfate (TBAHS) and solid potassium hydroxide in toluene (Scheme 1). This treatment resulted in cyclization to the bicycle **9** (d.r. 4:1) in which the product bearing *trans* stereochemistry across the ring junction predominates.^7^

**Figure 1:**
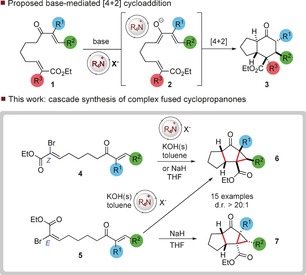
Phase‐transfer‐mediated intramolecular Diels–Alder‐type process. THF=tetrahydrofuran.

**Scheme 1:**
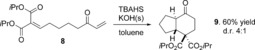
Reaction conditions: a) Substrate (0.17 mmol), TBAHS (0.1 equiv), KOH (s, 4 equiv), toluene. The d.r. value was established by ^1^H NMR analysis of the crude reaction mixture.

This chemistry could exemplify a useful approach to the synthesis of complex ring systems, and we rationalized that variation of substituents on the dienophile could offer the potential for the construction of quaternary all‐carbon centers. We reasoned that introduction of a halogen onto the dienophile would enable a range of carbon–carbon bonds to be made through metal‐mediated coupling reactions. To achieve this aim, the keto aldehyde **10** was treated with an in situ generated brominated Still–Gennari reagent,^8^ but rather than the anticipated brominated *E*‐olefin **11**, we isolated the 5,5,3‐tricycle **12** in moderate yield (40 %) as a single diastereoisomer (Scheme 2). We presume that the fused cyclopropane **12** 7 arises from a base‐promoted cyclization of the intermediate **11**.^9^ To probe this possibility, an alternative synthetic sequence was adopted in which the (*E*)‐α‐bromoacrylate could be isolated without initiating the cyclization process. Treatment of **11** with NaH in THF afforded the same tricycle **12** in 65 % yield as a single diastereomer.

**Scheme 2:**
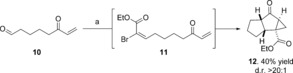
Reaction conditions: NaH (2 equiv), NBS (1 equiv), THF, ethyl *P*,*P*‐bis(2,2,2‐trifluoroethyl)phosphonoacetate (1 equiv), RT. The d.r. value was established ^1^H NMR analysis of the crude reaction mixture. NBS=*N*‐bromosuccinimide.

We examined the generality of this cascade process through the generation of a range of substituted (*E*)‐α‐bromoacrylates (**5**) and their treatment with NaH in THF at room temperature (Table 1). The cascade reaction is successful with substitution at both positions of the α,β‐unsaturated ketone. Alkyl and aryl groups (**13** R^2^=Me, **14** R^2^=Et, **15** R^2^=Ph) are smoothly incorporated into the tricyclic framework to form contiguous all‐carbon quaternary stereocenters with little variation in yield and with complete diastereoselectivity (>20:1 d.r.). For substrates bearing a substituent at the β‐position of the enone, (**16**; R^3^=*m*‐FC_6_H_4_, **17**; R^3^=Ph, **18**; R^3^=*p*‐CH_3_C_6_H_4_), tricycles bearing five contiguous stereocenters can be generated as a single diastereomer (d.r. >20:1). Intriguingly, when **11** was treated with TBAHS and solid KOH, cyclization to form a different 5,5,3‐tricycle (**19**) as a single diastereomer occurred.^7^ To probe the generality of this phenomenon, we examined the cyclization of a range of substrates under these reaction conditions. The cascade occurs smoothly to afford tricyclic products with four or five stereocenters as a single diastereoisomer (**19**–**25**), but in all cases the stereochemistry around the cyclopropane nucleus is reversed relative to that observed when using NaH and THF.

**Table 1:** Base‐mediated cascade cyclizations of (*E*)‐α‐bromoacrylates. 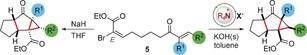

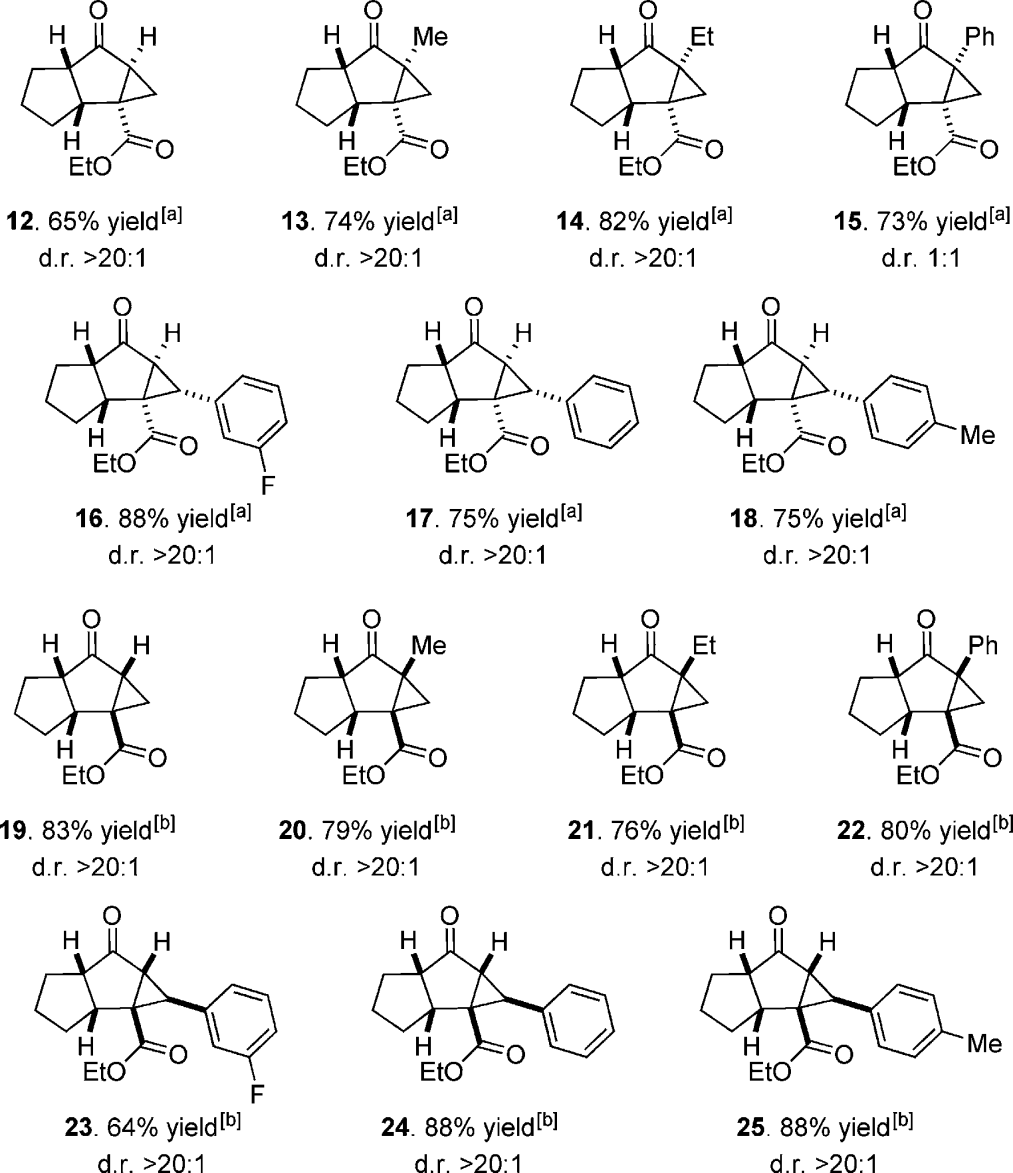

Reaction conditions: a) Substrate (0.17 mmol) NaH (2 equiv), THF, RT. b) substrate (0.17 mmol) KOH (2 equiv), TBAHS (0.1 equiv), RT. The d.r. value was established ^1^H NMR analysis of the crude reaction mixture. Yields are those of the isolated material.

Tricyclic products from the cascade processes can be chemoselectively derivatized, with the ketone in **19** enabling useful transformations (Scheme 3). Deprotonation using sodium in EtOD mediates selective deuteration only at the 5,5‐ring junction to yield **26**. Tricycle **19** undergoes highly chemo‐ and stereoselective Grignard addition (to give **27**), reduction (to give **28**), and reductive amination to afford amine **29**. In all cases the nucleophile approaches from the less hindered convex face of the system. Single‐electron reduction with sodium naphthalenide gave the *cis*‐indanyl framework **30** as the major diastereomer (d.r. 4:1).

**Scheme 3:**
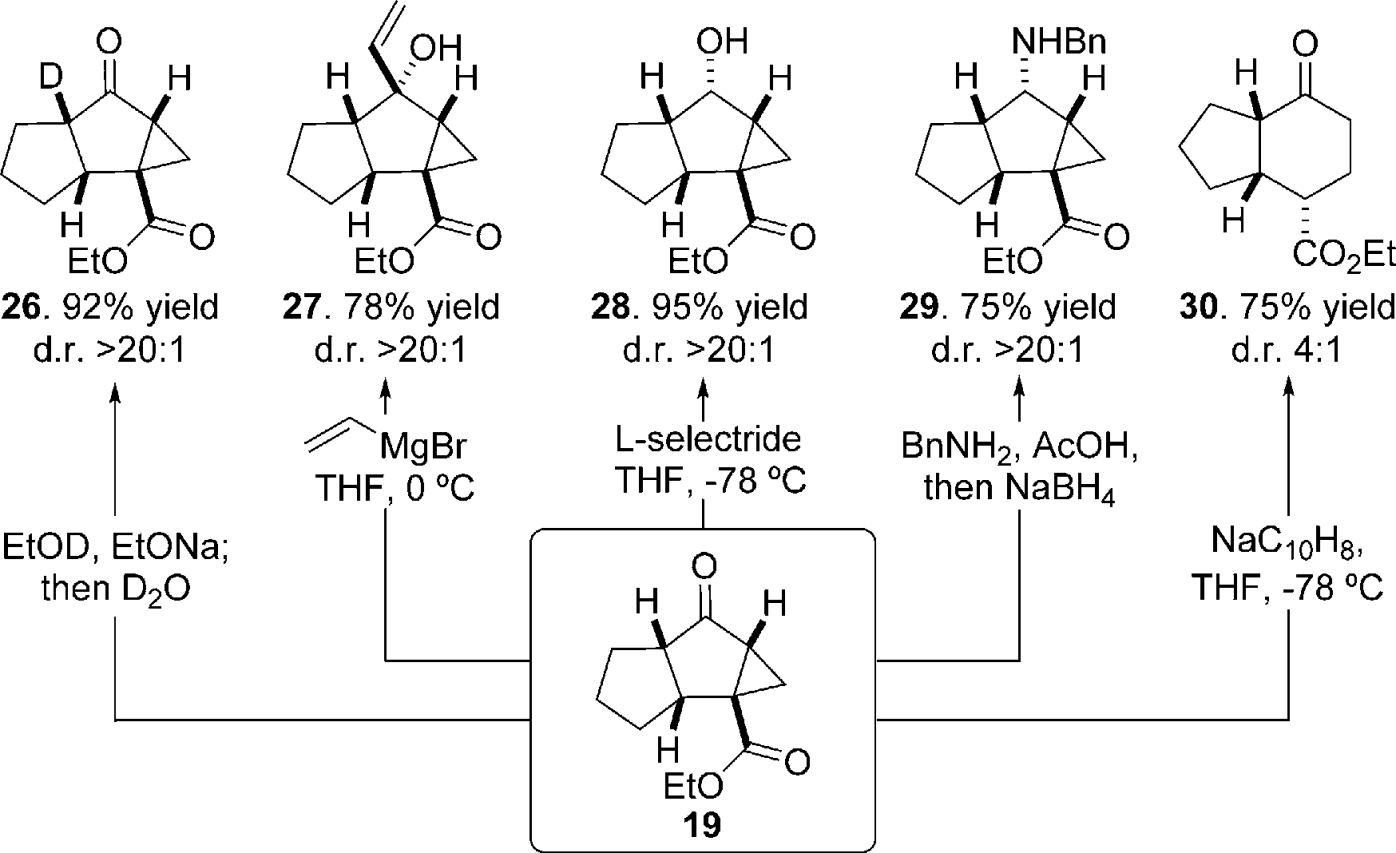
Diastereoselective derivatizations of the tricyclic scaffold. The d.r. value was established ^1^H NMR analysis of the crude reaction mixture.

The stereochemically distinct cyclization resulting from different reaction conditions could potentially be explained by: 1) interconversion between the two diastereoisomeric tricyclic systems under the basic reaction conditions, 2) *E*/*Z* isomerization of the bromoalkene prior to cyclization, or 3) the change of counterion (from sodium to ammonium) changing the nature of the cyclization cascade. Treatment of the diastereoisomeric cyclopropanes **12** and **9** under basic reaction conditions (either NaH in THF or TBAHS and KOH in toluene) did not lead to any change in diastereoisomeric ratio, and cyclopropanes were recovered unchanged, thus ruling out scenario 1. We reasoned that the stereochemical difference observed under different reaction conditions could reflect a stereospecific cyclization based on alkene geometry. To investigate this, we examined the cyclization of the (*Z*)‐α‐bromoacrylate **31** under both reaction conditions previously investigated (Scheme 4). The cyclopropane **19** was the only product obtained upon treatment with NaH in THF (d.r. >20:1), and is consistent with a stereospecific ring closure under these reaction conditions. However, treatment of **31** with TBAHS and KOH in toluene also led to formation of the all‐*cis* cyclopropane **19** as a single diastereoisomer.^10^ This outcome could be explained by interconversion of the *E*‐ and *Z*‐bromoacrylates under phase‐transfer conditions. We probed the potential for this interconversion under a range of reaction conditions but did not observe any evidence of a change in alkene geometry.^10^

**Scheme 4:**
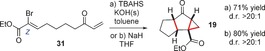
Reaction conditions: a) Substrate (0.17 mmol), TBAHS (0.1 equiv), KOH (s, 4 equiv), toluene. b) Substrate (0.17 mmol), NaH (2 equiv), THF, RT. The d.r. value was established ^1^H NMR analysis of the crude reaction mixture.

To probe either a potential change in mechanism or alkene geometry, we turned to quantum calculations. DFT calculations at the M06‐2X/6‐311++G(d,p) level were used with an implicit (CPCM) description of the solvents used experimentally.^11^ The LANL08 uncontracted basis set/relativistic ECP was used for Br atoms.^12^ This level of theory has been successfully applied to study anionic cyclizations^13^ and for several model systems gave kinetic and thermodynamic parameters within 1 kcal mol^−1^ of more expensive composite ab initio CBS‐Q//B3 calculations (see the Supporting Information).^10^ The cyclization was evaluated separately using descriptions of toluene and THF solvation, thus giving comparable results.

We found a stepwise mechanism for the formal [4+2] cyclization: the formation^14^ of either diastereomer proceeds via a stable intermediate enolate and is not concerted (Figure 2). Two‐dimensional scans of the potential energy surface confirmed the absence of a concerted TS (see the Supporting Information). The first C−C bond is formed irreversibly (via **TS 33** or **34**) with a kinetically controlled preference of 2.5 kcal mol^−1^ for the *trans*‐diastereomer. This step is the stereocontrolling step and is consistent with our observations in Figure 1. The second, ring‐closing step is reversible and has no impact upon stereoselectivity. Consistent with previous experiments and MP2 calculations, the *cis*‐fused bicyclic product **40** is more stable than the *trans* diastereomer; since it is not observed, the stereoselectivity of this reaction is kinetically controlled and product equilibration does not occur.^15^, ^16^

**Figure 2:**
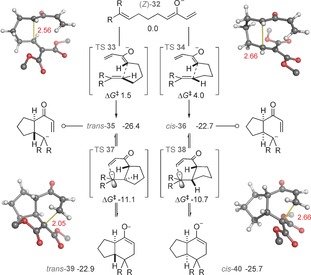
Formal [4+2] cyclization occurs by a stepwise Michael/Michael process. CPCM‐M062X/6–311++G(d,p) *G*_rel_(298 K), R=CO_2_Me.

We computed reaction pathways for the formation of 5,5,3‐fused tricyclic systems from bromoacrylates (Figure 3). As above, a *trans*‐selective, stepwise cyclization operates for both *E* and *Z* substrates. Based on the exergonicity of each C−C bond‐forming step, these are predicted to occur irreversibly, thus forming 5,6‐adducts epimeric at the brominated stereocenter (*trans*‐**47** and *trans*‐**53**). The subsequent intramolecular enolate displacement of bromide is rapid and irreversible via TS **49** and TS **55**. There is a substantial thermodynamic driving force (>9 kcal mol^−1^) for the epimerization of the 5,5,3‐fused products from 5,5‐*trans* to the observed 5,5‐*cis* relative stereochemistry. The computations predict that *cis*‐**57** will result from cyclization of the (*Z*)‐α‐bromoacrylate, and was observed under all experimental conditions studied.

**Figure 3:**
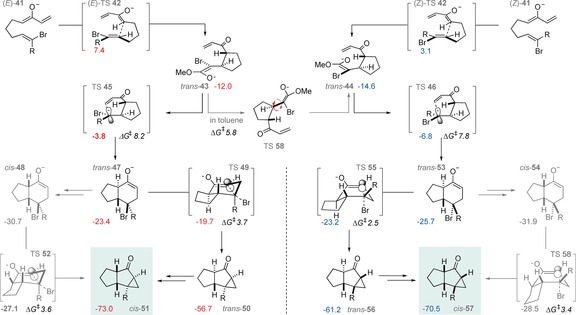
The 5,5,3‐fused tricyclic products are formed by stepwise [4+2] cycloaddition, irreversible enolate displacement of bromide, and epimerization to the more stable *cis*‐fused tricycle. CPCM‐M062X/6‐311++G(d,p)/LANL08 G_rel_(298 K), R=CO_2_Me.

The irreversible cyclization of the (*E*)‐α‐bromoacrylate is predicted to form *cis*‐**51**, which corresponds to the observed product in THF. Since the first C−C bond formation is predicted to occur irreversibly, *E* to *Z* interconversion of the starting material will be prevented, but why then is there a switch in diastereoselectivity to form *cis*‐**57**? We found that an exocyclic C−C rotation (via TS **58**) after the first C−C bond is formed, enables crossover to the diastereoisomeric pathway. For the *E* substrate in toluene this rotation occurs faster (ΔΔ*G* ^≠^=2.4 kcal mol^−1^) than the second C−C bond‐forming step. However, when a coordinating counterion (e.g. Na^+^) is present, the barrier for exocyclic C−C rotation rises to 14.2 kcal mol^−1^, and is now higher than for the subsequent C−C bond‐forming step. Under these reaction conditions the reaction is stereospecific because free‐rotation occurs more slowly than the second cyclization step.

With the conclusion that the counterion plays a key role in determining the ease of interconversion between *trans*‐**43** and *trans*‐**44**, we probed directly the effect that the counterion has on diastereoselectivity (Table 2). We observed that increasing solvent polarity favored **19**, with a complete reversal in diastereoselectivity for reactions in DMSO and DMF versus those in THF. We considered that this could may reflect solvation of the counterion by the dipolar aprotic solvent, thus favoring crossover from *trans*‐**43** to *trans*‐**44**. To probe this further, we added ligands to complex the sodium counterion. Addition of [2.2.1]cryptand in the presence of NaH in THF led to a complete reversal of diastereoselectivity from **12** to **19**, and is consistent with sequestration of the counterion, thus leading to rapid equilibration between *trans*‐**43** and *trans*‐**44** via TS **58**. Changing the size of the metal counterion also has an effect consistent with the observations above, with a larger counterion (Cs^+^) favoring the formation of **19** as opposed to a smaller counterion (Na^+^).

**Table 2:** Counterion, ligand, and solvent effects in cyclization of **5**. 
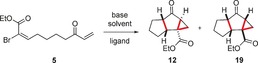

Solvent^[a]^ (d.r. **12**:**19**)	THF >95:5	MeCN 40:60	DMF >5:95	DMSO >5:95
Ligand^[b]^ (d.r. **12**:**19**)	none >95:5	15‐C‐5 71:29	15‐C‐5* 60:40	[2.2.1]cryptand >5:95
Base^[c]^ (d.r. **12**:**19**)	NaOH >95:5	KOH >95:5	RbOH >95:5	CsOH 61:39

Reaction conditions: [a] Substrate (0.17 mmol) NaH (2 equiv), solvent, RT. [b] Substrate (0.17 mmol) NaH (2 equiv), THF, ligand (1 equiv). * 2 equiv ligand used. [c] Substrate (0.17 mmol), base (2 equiv), THF, RT. DMF=*N*,*N*‐dimethylformamide, DMSO=dimethylsulfoxide.

We have demonstrated a robust synthetic method for the generation of 5,5,3‐fused tricyclic systems with excellent diastereoselectivity. Computation and experiment have demonstrated that the reaction proceeds by a stepwise Michael/Michael/cyclopropanation/epimerization cascade, in which the size and coordinating ability of the counterion is key to diastereoselectivity.

## Supporting information

As a service to our authors and readers, this journal provides supporting information supplied by the authors. Such materials are peer reviewed and may be re‐organized for online delivery, but are not copy‐edited or typeset. Technical support issues arising from supporting information (other than missing files) should be addressed to the authors.

Supplementary
