# Supplementary material for: A Counterion‐Directed Approach to the Diels–Alder Paradigm: Cascade Synthesis of Tricyclic Fused Cyclopropanes
Source: Angew Chem Int Ed Engl. 2016 Oct 7;55(44):13813–7. doi: 10.1002/anie.201608534 (PMC5113799; doi:10.1002/anie.201608534)
Supplement: Supplementary file 1 — Supplementary [file ANIE-55-13813-s001.pdf]

Angewandte Chemie
Eine Zeitschrift der Gesellschaft Deutscher Chemiker

Supporting Information

A Counterion-Directed Approach to the Diels–Alder Paradigm: Cascade Synthesis of Tricyclic Fused Cyclopropanes

Emily Kiss, Craig D. Campbell, Russell W. Driver, John D. Jolliffe, Rosemary Lang, Tetiana Sergeieva, Sergiy Okovytyy, Robert S. Paton, and Martin D. Smith**

anie_201608534_sm_miscellaneous_information.pdf

Supporting Information

1.1 General Experimental S2

1.2 General schemes for the synthesis of cyclization substrates S3

1.3 Synthetic Procedures S4

1.3.1 Synthesis of cyclization precursors S4

1.3.2 Base-promoted Cyclizations S24

1.3.3 Cyclization Product Derivatizations: S24

1.3.4 Experimental elucidation of the mechanism S41

2. X-Ray Crystallographic Data S45

3. Computational procedures S47

4. NMR spectra S67

1.1 General Experimental

NMR Spectra: Proton (^{1}H), carbon (^{13}C) and fluorine (^{19}F) NMR spectra were recorded on a Bruker AVC500 (500/126/471 MHz), Bruker DPX400 (400/101/377 MHz) or Bruker AVF400 (400/101/377 MHz). Proton, carbon and fluorine chemical shift data ($\delta_{H,}$ $\delta_{C,}$ δ_{F}) are quoted in ppm. ^{1}H NMR spectra were recorded using an internal deuterium lock for the residual protons in the respective deuterated solvent, *i.e.* $CDCl_3$ (δ 7.26), C_6D_6 (δ 7.16), toluene-d_8 (δ 2.09). ^{13}C NMR spectra were recorded using an internal deuterium lock for the relevant deuterated solvent, *i.e.* $CDCl_3$ (δ 77.0) C_6D_6 (δ 128.4), toluene-d_8 (δ 20.4). ^{19}F NMR spectra were referenced to trichlorofluoromethane (δ 0.00). Assignments were made on the basis of chemical shift, coupling constants, COSY, HSQC, HMBC, nOe and NOSEY data. Resonances are described using the following abbreviations; s (singlet), d (doublet), t (triplet), q (quartet), quin. (quintet), sext. (sextet), sept. (septet), m (multiplet), br. (broad) and dd (double doublet). Coupling constants (*J*) are rounded to the nearest 0.5 Hz.

Mass Spectra: Low resolution mass spectra were recorded on a Micromass LCT Premier spectrometer (ESI). High resolution mass spectra were recorded by the Mass Spectrometry service of the Chemistry Research Laboratory, University of Oxford, using a Bruker Daltronics microTOF spectrometer (ESI). *m/z* values are reported in Daltons with values being within a tolerance of 5 ppm of the calculated molecular formula.

Infrared Spectra: Infrared spectra were recorded on a Bruker Tensor 27 Fourier Transform Spectrometer, as a thin film on NaCl plates or a diamond ATR module. Absorption maxima (ν_{max}) are quoted in wavenumbers (cm^{-1}).

Chromatography techniques: TLC was performed on Merck Kieselgel 60 F_{254} 0.2 mm precoated plates and visualised using basic potassium permanganate dip, acidic vanillin dip or ultraviolet light. Column chromatography was performed on Merck Kieselgel 60 SiO_2 (40-63 μm) and the solvent system used is recorded in parentheses.

Solvents: Solvents were either used as commercially supplied, or as purified by standard techniques. Brine refers to a saturated aqueous solution of sodium chloride. Unless otherwise stated, reactions were carried out in oven-dried flasks under an atmosphere of dry argon.

Reagents: All reagents were obtained from commercial suppliers Sigma-Aldrich, Alfa-Aesar, Fisher Scientific, Apollo Scientific or Fluorochem and used as supplied unless otherwise specified. Anhydrous solvents were obtained following passage through columns packed with activated alumina beads. *N*-Bromosuccinimide was recrystallized from boiling H_2O.

Nomenclature: Compound names are generated using PerkinElmer ChemDraw® Professional, version 15.0.0.106, but the numbering on the compounds is arbitrary and indicated for ease of assignment.

1.2 General schemes for the synthesis of cyclization substrates

*Scheme for preparation of aldehyde **10:***

Scheme 1

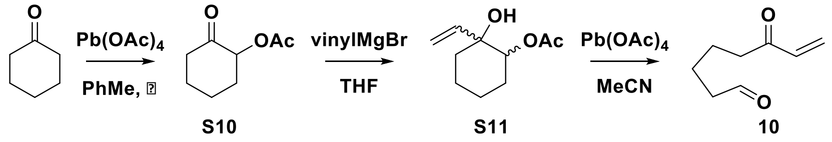

Schemes for preparation of cyclization substrates:

Scheme 2

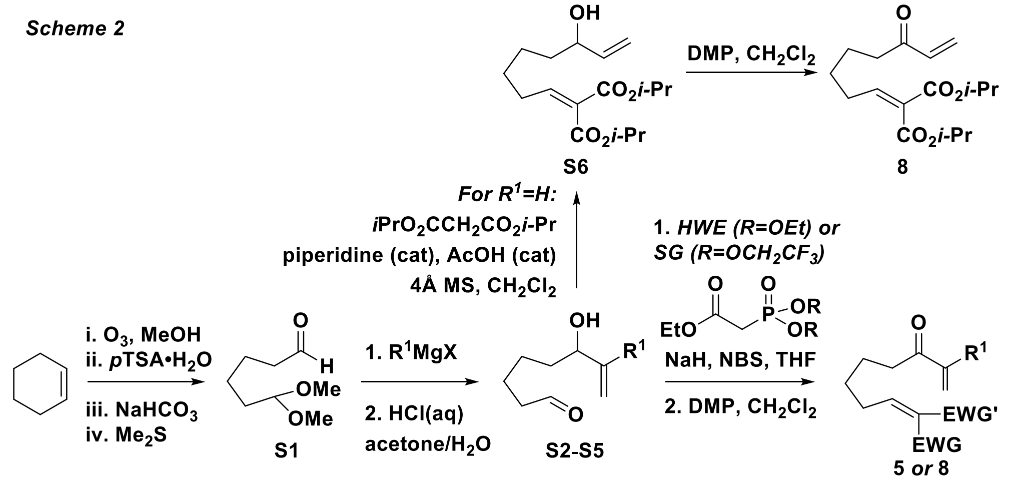

#	R^1
**S2**	H
**S3**	Me
**S4**	Et
**S5**	Ph

#	R^1	EWG	EWG'
**8**	H	CO_2iPr	CO_2iPr
**(*E*)-5a**	H	CO_2Et	Br
**(*Z*)-5a**	H	Br	CO_2Et
**(*E*)-5b**	Me	CO_2Et	Br
**(*Z*)-5b**	Me	Br	CO_2Et
**(*E*)-5c**	Et	CO_2Et	Br
**(*Z*)-5c**	Et	Br	CO_2Et
**(*E*)-5d**	Ph	CO_2Et	Br
**(*Z*)-5d**	Ph	Br	CO_2Et

HWE: Horner-Wadsworth-Emmons olefination
SG: Still-Gennari olefination

Scheme 3

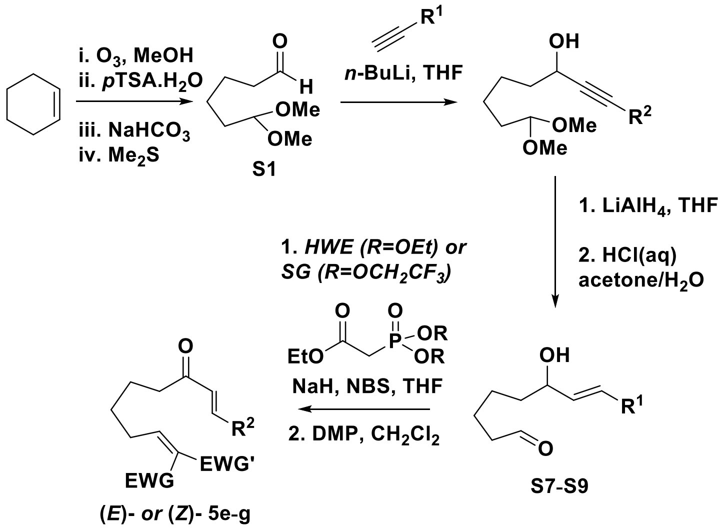

#	R^1	EWG	EWG'
**(*E*)-5e**	Ph	CO_2Et	Br
**(*Z*)-5e**	Ph	Br	CO_2Et
**(*E*)-5f**	3-FC_6H_4	CO_2Et	Br
**(*Z*)-5f**	3-FC_6H_4	Br	CO_2Et
**(*E*)-5g**	4-MeC_6H_4	CO_2Et	Br
**(*Z*)-5g**	4-MeC_6H_4	Br	CO_2Et

#	R^1
**S7**	Ph
**S8**	3-FC_6H_4
**S9**	4-MeC_6H_4

1.3 Synthetic Procedures

1.3.1 Synthesis of cyclization precursors

Ozonolysis:

Aldehyde **S1** was prepared according to a literature procedure.[1]

Grignard Addition and Deprotection *(Scheme 1)*:

Commercially available Grignard reagents were used directly. When the Grignard reagent was not commercially available, the following procedure was adopted:

Magnesium (1 eq.) was flame dried in an oven-dried flask under vacuum for 5 min. The flask was allowed to cool to RT, and I_2 (1 crystal) and THF (1 mL/mmol) were added. Appropriate bromo-olefin (1 eq.) was added dropwise to maintain a gentle reflux. Once the magnesium was consumed, the Grignard reagent was used immediately.

Grignard reagent (1.2 eq.) was added dropwise to a solution of aldehyde **S1** (1 eq.) in THF (8 mL/mmol) at −78 °C, and the resulting solution was stirred at this temperature for 3 hr. The reaction mixture was then allowed to warm to RT and NH_4Cl (sat. aq.) was added. The layers were separated and the aqueous layer was extracted with EtOAc (× 3). The combined

extracts were washed with H_2O, dried over $MgSO_4$, filtered and the filtrate concentrated under reduced pressure. Acetone (8 mL/mmol), H_2O (1.5 mL/mmol) and HCl (1.0 M aq., 0.5 mL/mmol) were added, and the resulting solution stirred at RT for 12 hr. $NaHCO_3$ (sat. aq.) was added, and the aqueous layer extracted with EtOAc (× 3). The combined extracts were washed with H_2O and dried over $MgSO_4$, filtered and the filtrate concentrated under reduced pressure to afford the title compound that was unstable to further purification. The crude aldehyde was either used immediately or stored under Ar at –20 °C.

Acetylide Addition, Alkyne Reduction and Acetal Deprotection *(Scheme 2)*:

N.B. Commercially available alkynes were used directly as supplied.

n-BuLi (1.15 eq. of a 2.5 M solution in hexanes) was added dropwise to a solution of terminal alkyne reagent (1.1 eq.) in THF (3 mL/mmol) at –78 °C, and the resulting solution was stirred at this temperature for 30 min. A solution of aldehyde **S1** (1 eq.) in THF (1.5 mL/mmol) was added and the mixture allowed to warm slowly to RT and H_2O was added. The layers were separated and the aqueous layer was extracted with Et_2O (× 3). The combined extracts were washed with H_2O, dried over $MgSO_4$, filtered and the filtrate concentrated under reduced pressure to afford the crude acetylide addition product compound that was unstable to further purification and used directly in subsequent reactions.

Propargylic Alcohol Reduction to trans-Allylic Alcohol

Lithium aluminium hydride (1.1 eq. of a 2 M solution in THF) was added dropwise to a solution of crude propargylic alcohol product (1 eq.) in THF (3 mL/mmol) at 0 °C (N.B. gas evolution), and the resulting solution was stirred at this temperature for 5 min. The reaction was quenched with Rochelle salt, stirred for 1 hr then extracted with Et_2O (× 3), dried over Na_2SO_4, filtered and the filtrate concentrated under reduced pressure to afford the crude allylic alcohol product that was unstable to purification. The layers were separated and the aqueous layer was extracted with Et_2O (× 3). The combined extracts were washed with H_2O, dried over $MgSO_4$, filtered and the filtrate concentrated under reduced pressure to afford the crude acetylide addition product compound that was unstable to further purification and used directly in subsequent reactions.

Acetal deprotection

To a solution of crude allylic alcohol (1 eq.) in acetone (8 mL/mmol) and H_2O (1.5 mL/mmol) was added HCl (1.0 M aq., 0.5 mL/mmol) and the resulting solution stirred at RT for 12 hr. $NaHCO_3$ (sat. aq.) was added, and the aqueous layer extracted with EtOAc (× 3). The combined extracts were washed with H_2O and dried over $MgSO_4$, filtered and the filtrate concentrated under reduced pressure to afford the crude aldehyde product that was

unstable to further purification. The crude aldehyde was either used immediately or stored under Ar at –20 °C.

N.B. An equilibrium mixture of hydroxy-aldehyde and hemiacetal are generally obtained but used as a mixture.

<u>Horner-Wadsworth-Emmons (HWE) or Still-Gennari (SG) bromo-olefination reactions (*Schemes 1 and 2*):</u>

Commercially available Horner-Wadworth-Emmons reagent, triethyl phosphonoacetate, was used. The Still-Gennari reagent, ethyl bis(2,2,2-trifluoroethyl)phosphonoacetate, was prepared using a known literature procedure.[2]

Horner-Wadworth-Emmons or Still-Gennari reagent (1 eq.) was dissolved in THF (5 mL/mmol). NaH (60% w/w dispersion in mineral oil, 2 eq.) and NBS (1 eq.) were added at RT and the resulting suspension stirred for 1 hr. Appropriate hydroxy-aldehyde (1 eq.) was dissolved in THF (2 mL/mmol), and added to the reaction mixture, and the resulting suspension stirred overnight. The reaction mixture was quenched with NH_4Cl (sat. aq.), the layers separated and the aqueous layer extracted with EtOAc (× 2). The combined extracts were washed with brine, dried over $MgSO_4$, filtered, and the filtrate concentrated under reduced pressure to afford the crude product. This crude product was adsorbed onto silica and purified by flash column chromatography (SiO_2, pet 40-60:Et_2O (8:2)) to afford the desired product.

Preparation of diisopropyl 2-(6-hydroxyoct-7-en-1-ylidene)malonate, S6

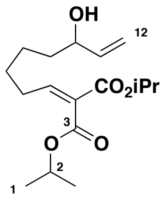

According to a modified literature procedure,[3] piperidine (70 µL, 7.0 µmol) and AcOH (40 µL, 7.0 µmol) were added to a solution of aldehyde **S1** (50 mg, 0.35 mmol) and diisopropyl malonate (70 µL, 0.40 mmol) in CH_2Cl_2 (5 mL) and the reaction mixture stirred at 0 °C for 45 min. 4 Å MS (~100 mg) were added, and the reaction mixture was allowed to warm to RT over 15 min. The solution was diluted with Et_2O, washed with H_2O, and the combined aqueous layers extracted with Et_2O (x 3). The combined organic extracts were washed with brine, dried over Na_2SO_4, filtered and the filtrate concentrated under reduced pressure to afford the crude product as a yellow oil. This crude product was purified by flash

column chromatography (SiO_2, pet 40-60: Et_2O (7:3)) to afford compound **S6** (60 mg, 60%) as a colorless oil.

^{1}H NMR (400 MHz, $CDCl_3$) δ_H 6.91 (t, *J* = 7.5 Hz, 1 H, *H*5), 5.84 (ddd, *J* = 17.0, 10.5, 6.5 Hz, 1 H, *H*11), 5.20 (dd, *J* = 17.0, 1.5 Hz, 1 H, *H*12), 5.11 (dd, *J* = 10.5, 1.5 Hz, 1 H, *H*12'), 5.08 (sept., *J* = 6.5 Hz, 1 H, *H*2), 5.06 (sept., *J* = 6.5 Hz, 1 H, *H2'*), 4.06 (t, *J* = 6.5 Hz, 1 H, *H*10), 2.30 (q, *J* = 7.5 Hz, 2 H, *H*6), 1.60-1.42 (m, 7 H, *H*7, *H*8, *H*9, O*H*), 1.23 (d, *J* = 6.5 Hz, 6 H, *H*1), 1.22 (d, *J* = 6.5 Hz, 6 H, *H*1'); **^{13}C NMR (101 MHz, $CDCl_3$)** δ_C 165.4 (*C*3), 163.7 (*C*3'), 148.2 (*C*5), 141.2 (*C*11), 129.7 (*C*4), 114.8 (*C*12), 73.1 (*C*10), 68.9 (*C*2, *C*2'), 36.7 (*C*9), 29.6 (*C*6), 28.3 (*C*7), 25.1 (*C*8), 21.8 (*C*1, *C*1'); **HRMS (ESI$^+$)** *m/z* calculated for $[M+Na]^+$ $C_{17}H_{28}NaO_5$ 335.1829, found 335.1819; **IR ν_{max}/cm^{-1} (thin film)** 3402 (br., OH), 2981 (C-H), 2936 (C-H), 1717 (C=O), 1646 (C=C), 1374, 1225, 1104.

Preparation of diisopropyl 2-(6-oxooct-7-en-1-ylidene)malonate, 8

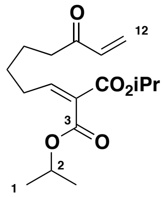

Following **General Procedure 3**, using DMP (116 mg, 0.270 mmol), alcohol **S6** (50 mg, 0.160 mmol) and CH_2Cl_2 (3 mL) afforded compound **8** (50 mg, quant.) as a colorless oil.

^{1}H NMR (400 MHz, $CDCl_3$) δ_H 6.91 (t, *J* = 7.5 Hz, 1 H, *H*5), 6.33 (dd, *J* = 17.0, 10.5 Hz, 1 H, *H*11), 6.20 (d, *J* = 17.0 Hz, 1 H, *H*12), 5.81 (d, *J* = 10.5 Hz, 1 H, *H*12'), 5.16 (sept., *J* = 6.5 Hz, 1 H, *H*2), 5.07 (sept., *J* = 6.5 Hz, 1 H, *H2'*), 2.59 (t, *J* = 7.5 Hz, 2 H, *H*9), 2.29 (q, *J* = 7.5 Hz, 2 H, *H*6), 1.65 (quin., *J* = 7.5 Hz, 2 H, *H*8), 1.50 (quin., *J* = 7.5 Hz, 2 H, *H*7), 1.29 (d, *J* = 6.5 Hz, 6 H, *H*1), 1.25 (d, *J* = 6.5 Hz, 6 H, *H*1'); **^{13}C NMR (101 MHz, $CDCl_3$)** δ_C 200.5 (*C*10), 165.3 (*C*3), 163.6 (*C*3'), 147.8 (*C*5), 136.6 (*C*11), 129.9 (*C*4), 128.2 (*C*12), 68.9 (*C*2, *C*2'), 39.2 (*C*9), 29.5 (*C*6), 28.0 (*C*7), 23.6 (*C*8), 21.9 (*C*1, *C*1'); **HRMS (ESI$^+$)** *m/z* calculated for $[M+Na]^+$ $C_{17}H_{26}NaO_5$ 333.1672, found 333.1667; **IR ν_{max}/cm^{-1} (thin film)** 2982 (C-H), 1720 (C=O), 1680 (C=O), 1646 (C=C), 1375, 1258, 1226.

Preparation of (3a*S,7a*S**)-diisopropyl 7-oxohexahydro-1*H*-indene-4,4(2*H*)-dicarboxylate, 9**

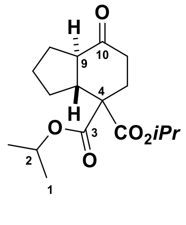

Tetrabutylammonium bisulfate (35 mg, 0.080 mmol, 10 mol%) was added to a solution of the cyclisation precursor **8** (1 eq.) in toluene (10 mL). Powdered KOH (74 mg, 1.32 mmol) was added and the resulting solution stirred at RT until the reaction was complete (determined by TLC). The mixture was quenched with NH_4Cl (sat. aq.) and allowed to warm to RT if appropriate. The layers were separated and the aqueous layer was extracted with CH_2Cl_2. The combined organic extracts were washed with brine, dried over $MgSO_4$, filtered and the filtrate concentrated under reduced pressure to afford the crude product. The crude product was adsorbed onto silica and purified by flash column chromatography (SiO_2, pet 40-60:Et_2O (8:2) to afford cyclized product **9** (60 mg, 60%, ~4:1 d.r.) as a colorless oil. .

^{1}H NMR (500 MHz, CDCl$_3$) δ_H 5.15 (sept., J = 6.5 Hz, 1 H, $H2_{major+minor}$), 5.07 (sept., J = 6.5 Hz, 1.25 H, $H2'_{major+minor}$), 3.11-3.06 (m, 0.25 H, $H9_{minor}$), 3.05-2.98 (m, 0.25 H, $H5_{minor}$), 2.74-2.59 (m, 3.25 H, $H9_{major}$, $H11_{major+minor}$, $H12_{major}$), 2.41-2.30 (m, 1.75 H, $H11'_{major,}$ $H6_{minor}$, $H8_{minor}$, $H12_{minor}$), 2.20-2.07 (m, 1.5 H, $H5_{major}$, $H11'_{minor,}$ $H12'_{minor}$), 2.06-1.90 (m, 3 H, $H12'_{major}$, $H6_{major}$, $H6'_{major}$), 1.80-1.72 (m, 2 H, $H8_{major}$, $H8'_{major}$), 1.69-1.57 (m, 2.5 H, $H7_{major}$, $H7'_{major}$, $H7_{minor}$, $H8'_{minor}$), 1.56-1.46 (m, 0.5 H, $H6'_{minor,}$ $H7'_{minor}$), 1.31-1.22 (m, 15 H, $H1_{major+minor}$, $H1'_{major+minor}$); **^{13}C NMR (126 MHz, CDCl$_3$)** δ_C 210.8 ($C10_{minor}$), 210.3 ($C10_{major}$), 170.9 ($C3_{minor}$), 170.7 ($C3_{major}$), 169.8 ($C3'_{minor}$), 169.6 ($C3'_{major}$), 69.4 ($C2_{minor}$), 69.3 ($C2_{major}$), 69.2 ($C2'_{minor}$), 69.1 ($C2'_{major}$), 56.7 ($C4_{major}$), 55.8 ($C4_{minor}$), 51.9 ($C9_{major}$), 50.9 ($C5_{major}$), 50.7 ($C9_{minor}$), 46.0 ($C5_{minor}$), 38.7 ($C11_{major}$), 37.3 ($C11_{minor}$), 34.3 ($C12$), 28.0 ($C6$), 22.6 ($C8_{major}$), 22.2 ($C7_{minor}$), 21.9 ($C1_{major}$), 21.8 ($C1_{minor}$), 21.7 (C1$'_{major}$), 21.7 (C1$'_{minor}$), 21.6 ($C8_{minor}$), 21.6 ($C7_{major}$); **HRMS (ESI$^+$)** *m/z* calculated for $[M+Na]^+$ $C_{17}H_{26}NaO_5$ 333.1672, found 333.1663; **IR ν_{max}/cm^{-1} (thin film)** 2979, 1717, 1707, 1455, 1375, 1233.

Preparation of 2-acetoxycyclohexanone S10

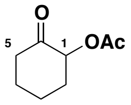

According to a literature procedure,[4] cyclohexanone (2.14 mL, 20.4 mmol) was dissolved in PhMe (40 mL), $Pb(OAc)_4$ (10.8 g, 12.2 mmol) was added and the solution heated to reflux for 2 hr. The reaction mixture was allowed to cool to RT, diluted with Et_2O and washed with HCl (1.0 M aq., × 3). The organic extract was dried over Na_2SO_4, filtered, and concentrated under reduced pressure to afford compound **S10** (3.00 g, 94%) as a yellow oil, which required no further purification.

^{1}H NMR (400 MHz, CDCl$_3$) δ_H 5.14 (dd, J = 6.5, 11.0 Hz, 1 H, $H1$), 2.54-2.46 (m, 1 H, $H5$), 2.45-2.23 (m, 2 H, $H2$, $H5$'), 2.16 (s, 3 H, COOCH_3), 2.12-2.04 (m, 1 H, $H2$'), 2.00-1.91 (m, 1 H, $H4$), 1.88-1.56 (m, 3 H, $H4$', $H3$, $H3$'); **^{13}C NMR (101 MHz, CDCl$_3$)** δ_C 204.6 ($C6$),

170.0 (CH_3CO_2), 76.7 (*C*1), 40.6 (*C*5), 32.8 (*C*2), 27.0 (*C*4), 23.7 (*C*3), 20.9 (CH_3CO_2); **LRMS (ESI$^+$)** *m/z* 157 ($[M+H]^+$ 11%), 179 ($[M+Na]^+$ 46%).
Spectroscopic properties of this compound are consistent with the data reported in the literature.[5]

Preparation of 1-vinylcyclohexane-1,2-diol, S11

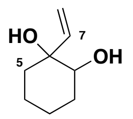

According to a literature procedure,[6] ketone **15** (2.6 g, 17 mmol) was stirred in THF (20 mL) at -78 °C for 15 min. Vinylmagnesium bromide (1.0 M in THF, 50 mL, 50 mmol) was added dropwise and the resulting solution was stirred at -78 °C for 1 hr. The reaction mixture was allowed to warm to RT and stirred for 1 hr, cooled to 0 °C, and quenched with H_2O. The mixture was then diluted with EtOAc, washed with NH_4Cl (sat. aq.) and brine (× 2), dried over K_2CO_3, filtered, and the filtrate concentrated under reduced pressure to afford the crude product as a yellow oil with 1:3 *dr* (by ^{1}H NMR spectroscopic analysis). The crude product was purified by flash column chromatography (SiO_2, pet 40-60:Et_2O (1:1)) to afford compound **S11** (1.4 g, 61%, 1:3 *dr*) as a yellow oil.
^{1}H NMR (400 MHz, $CDCl_3$) δ_H 5.90 (dd, *J* = 10.5, 17.5 Hz, 1 H, *H*7), 5.41 (dd, *J* = 1.5, 17.5 Hz, 1 H, *H*8), 5.21 (dd, *J* = 1.5, 10.5 Hz, 1 H, *H*8'), 3.51 (d, *J* = 10.0 Hz, 1 H, *H*1), 2.21 (s, 1 H, O*H*), 1.85-1.14 (m, 9 H, *H*2, *H*3, *H*4, *H*5, O*H*); **^{13}C NMR (101 MHz, $CDCl_3$)** δ_C 143.5 (*C*7), 116.2 (*C*8), 74.2 (*C*6), 73.2 (*C*1), 35.5 (*C*5), 28.9 (*C*2), 22.6 (*C*4), 20.6 (*C*3); **LRMS *m/z* (ESI$^+$)** 142 ($[M+Na]^+$ 100%).
Spectroscopic properties of this compound are consistent with the data reported in the literature.[3]

Preparation of 6-oxooct-7-enal, 10

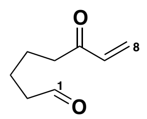

According to a literature procedure,[4] $Pb(OAc)_4$ (640 mg, 1.46 mmol) was added to a solution of diol **16** (200 mg, 1.46 mmol) in MeCN (10 mL) and the resulting solution was stirred at RT for 10 min. The solution was diluted with EtOAc and washed with HCl (1.0 M aq., × 3). The combined extracts were dried over $MgSO_4$, filtered, and the filtrate concentrated under reduced pressure to afford compound **14** (190 mg, 97%) as a colourless oil, which was unstable to further purification, so was used crude in the next reaction.

^{1}H NMR (200 MHz, $CDCl_3$) showed characteristic peaks at 9.81 (s, 1 H, *H*1), 6.35 (dd, *J* = 17.5, 9.5 Hz, 1 H, *H*7), 6.21 (dd, *J* = 17.5, 1.5 Hz, 1 H, *H*8), 5.84 (dd, *J* = 9.5, 1.5 Hz, 1 H, *H*8′).

Alternative preparation:
A solution of diol **16** (200 mg, 1.46 mmol) in CH_2Cl_2 (10 mL) was added to a solution of sodium (meta)periodate (600 mg, 2.82 mmol) in H_2O (10 mL). The resulting biphasic mixture was vigorously stirred for 2 h. The phases were separated, and the aqueous layer was extracted with CH_2Cl_2 (× 3). The combined extracts were washed with H_2O and brine, dried over $MgSO_4$, filtered, and the filtrate concentrated under reduced pressure to afford compound **14** (204 mg, quant.) as a colourless oil, which was unstable to further purification, so was used crude in the next reaction, with the same characteristic peaks as previously reported.

Preparation of (*E*)-ethyl 8-hydroxydeca-2,9-dienoate, S12

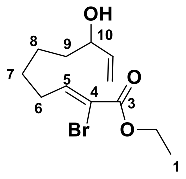

Using Horner-Wadworth-Emmons reagent (1.6 g, 7.00 mmol), NaH (560 mg, 14.0 mmol), NBS (1.2 g, 7.00 mmol) and aldehyde **S2** (1.0 g, 7.00 mmol) afforded the title compound **S12** (500 mg, 48% over two steps) as a 5:1 (*Z*:*E*) mixture of isomers.

^{1}H NMR (400 MHz, $CDCl_3$) δ_H 7.28 (t, *J* = 7.0 Hz, 1 H, *H*5), 5.86 (ddd, *J* = 17.0, 10.5, 6.5 Hz, 1 H, *H*11), 5.23 (dd, *J* = 17.0, 1.5 Hz, 1 H, *H*12), 5.11 (dd, *J* = 10.5, 1.5 Hz, 1 H, *H*12′), 4.27 (q, *J* = 7.0 Hz, 2 H, *H*2), 4.15-4.05 (m, 1 H, *H*10), 2.36 (d, *J* = 7.0 Hz, 2 H, *H*6), 1.65-1.40 (m, 7 H, *H*7, *H*8, *H*9, O*H*), 1.33 (t, *J* = 7.0 Hz, 3 H *H*1); **^{13}C NMR (101 MHz, $CDCl_3$)** δ_C 163.5, 148.3, 145.9, 141.1, 114.8, 73.0, 62.4, 36.7, 32.0, 28.6, 25.0, 14.3; **HRMS (ESI$^+$)** *m/z* calculated for $[M+Na]^+$ $C_{12}H_{19}BrNaO_3$ 313.0410, found 313.0400; **IR ν_{max}/cm^{-1} (thin film)** 3402, 2934, 1714, 1624, 1368, 1255, 1041.

E isomer data omitted for clarity.

Preparation of (*E*)-ethyl 2-bromo-8-hydroxydeca-2,9-dienoate, S13

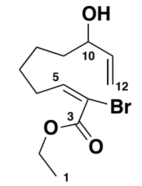

Using Still-Gennari reagent (2.3 g, 7.0 mmol), NaH (560 mg, 14.0 mmol), NBS (1.2 g, 7.0 mmol) and aldehyde **S2** (1.0 g, 7.0 mmol) afforded the title compound **S13** (533 mg, 53% over two steps).

^{1}H NMR (400 MHz, $CDCl_3$) δ_H 6.59 (t, *J* = 7.5 Hz, 1 H, *H*5) 5.79 (d, *J* = 17.0, 10.5, 6.0 Hz, 1 H, *H*11), 5.15 (dt, *J* = 17.0, 1.5 Hz, 1 H, *H*12), 5.04 (dt, *J* = 10.5, 1.5 Hz, 1 H, *H*12′), 4.20 (q, *J* = 7.0 Hz, 2 H, *H*2), 4.03 (q, *J* = 6.0 Hz, 1 H, *H*10), 2.44 (q, *J* = 7.5 Hz, 2 H, *H*6), 1.58 (br. s, 1 H, O*H*), 1.51-1.35 (m, 6 H, *H*7, *H*8, *H*9), 1.27 (t, *J* = 7.0 Hz, 3 H, *H*1); **^{13}C NMR (101 MHz, $CDCl_3$)** δ_C 163.0, 148.3, 141.1, 114.7, 111.3, 73.1, 62.4, 36.6, 31.3, 27.5, 25.0, 14.1; **HRMS (ESI$^+$)** *m/z* calculated for $[M+Na]^+$ $C_{12}H_{19}BrNaO_3$ 313.0410, found 313.0412; **IR ν_{max}/cm^{-1} (thin film)** 3401, 2934, 1715, 1370, 1226, 1027.

Preparation of (*Z*)-ethyl 2-bromo-8-hydroxy-9-methyldeca-2,9-dienoate, S14

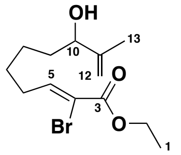

Using Horner-Wadworth-Emmons reagent (1.4 g, 6.4 mmol), NaH (512 mg, 12.8 mmol), NBS (1.1 g, 6.4 mmol) and aldehyde **S3** (1.0 g, 6.4 mmol) afforded the title compound **S14** (803 mg, 81% over two steps) as a 1:5 (*E*:*Z*) mixture of isomers.

E isomer data omitted for clarity.

^{1}H NMR (400 MHz, $CDCl_3$) δ_H 7.13 (t, *J* = 7.0 Hz, 1 H, *H*5) 4.80-4.77 (m, 1 H, *H*12) 4.70-4.67 (m, 1 H, *H*12′), 4.12 (q, *J* = 7.0 Hz, 2 H, *H*2), 3.90-3.82 (m, 1 H, *H*10), 2.20 (q, *J* = 7.0 Hz, 2 H, *H*6), 1.57 (s, 3 H, *H*13), 1.46-1.34 (m, 7 H, *H*7, *H*8, *H*9, O*H*), 1.18 (t, *J* = 7.0 Hz, 3 H, *H*1); **^{13}C NMR (101 MHz, $CDCl_3$)** δ_C 162.5, 147.5, 145.0, 116.5, 111.1, 75.8, 62.4, 34.5, 32.0, 27.5, 25.3, 17.5, 14.1; **HRMS (ESI$^+$)** *m/z* calculated for $[M+Na]^+$ $C_{13}H_{21}BrNaO_3$ 327.0566, found 327.0561; **IR ν_{max}/cm^{-1} (thin film)** 3411, 2936, 1714, 1624, 1447, 1369, 1257.

Preparation of (*E*)-ethyl 2-bromo-8-hydroxy-9-methyldeca-2,9-dienoate, S15

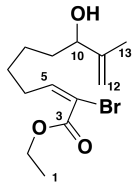

Using Still-Gennari reagent (2.1 g, 6.4 mmol), NaH (512 mg, 12.8 mmol), NBS (1.1 g, 6.4 mmol) and aldehyde **S3** (1.0 g, 6.4 mmol, prepared from isopropenylmagnesium bromide (0.5 M in THF)) afforded the title compound **S15** (461 mg, 46% over two steps).

^{1}H NMR (400 MHz, CDCl$_3$) δ_H 6.67 (t, *J* = 7.0 Hz, 1 H, *H*5) 4.93 (s, 1 H, *H*12), 4.84 (s, 1 H, *H*12′), 4.27 (qt, *J* = 7.0, 1.0 Hz, 2 H, *H*2), 4.05 (t, *J* = 6.5 Hz, 1 H, *H*10), 2.51 (q, *J* = 7.0 Hz, 2 H, *H*6), 1.71 (br. s, 1 H, O*H*), 1.72 (s, 3 H, *H*13), 1.60-1.40 (m, 6 H, *H*7, *H*8, *H*9), 1.34 (t, *J* = 7.0 Hz, 3 H, *H*1); **^{13}C NMR (101 MHz, CDCl$_3$)** δ_C 163.0, 148.4, 147.5, 146.0, 111.2, 75.7, 62.4, 34.5, 32.0, 27.5, 25.3, 17.5, 14.1; **HRMS (ESI$^+$)** *m/z* calculated for $[M+Na]^+$ $C_{13}H_{21}BrNaO_3$ 327.0566, found 327.0577; **IR ν_{max}/cm^{-1} (thin film)** 3415, 2936, 1715, 1447, 1370, 1224.

Z isomer data omitted for clarity.

Preparation of (*Z*)-ethyl 2-bromo-8-hydroxy-9-ethyldeca-2,9-dienoate, S16

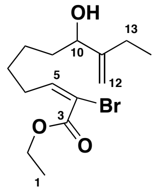

Using Horner-Wadworth-Emmons reagent (1.3 g, 5.9 mmol), NaH (472 mg, 11.8 mmol), NBS (1.0 g, 5.9 mmol) and aldehyde **S4** (1.0 g, 5.9 mmol, prepared from 2-bromo-1-butene) afforded the title compound **S16** (403 mg, 40% over two steps) as a 4:1 *Z*:*E* mixture of isomers.

E isomer data omitted for clarity.

^{1}H NMR (400 MHz, CDCl$_3$) δ_H 7.30 (t, *J* = 7.5 Hz, 1 H, *H*5) 5.03 (s, 1 H, *H*12), 4.86 (s, 1 H, *H*12′), 4.29 (q, *J* = 7.0 Hz, 2 H, *H*2), 4.12-4.09 (m, 1 H, *H*10), 2.37 (q, *J* = 7.5 Hz, 2 H, *H*6), 2.19-1.95 (m, 2 H, *H*13), 1.63-1.45 (m, 7 H, *H*7, *H*8, *H*9, O*H*), 1.34 (t, *J* = 7.0 Hz, 3 H, *H*1), 1.09 (t, *J* = 7.5 Hz, 3 H, *H*14); **^{13}C NMR (101 MHz, CDCl$_3$)** δ_C 163.0, 153.5, 148.4, 116.5, 108.5, 75.4, 62.4, 35.1, 31.4, 27.5, 25.4, 23.9, 14.2, 12.2; **HRMS (ESI$^+$)** *m/z* calculated for $[M+Na]^+$ $C_{14}H_{23}BrNaO_3$ 341.0723, found 341.0717; **IR ν_{max}/cm^{-1} (thin film)** 3420, 2936, 1715, 1650, 1624, 1461.

Preparation of (*E*)-ethyl 2-bromo-8-hydroxy-9-ethyldeca-2,9-dienoate, S17

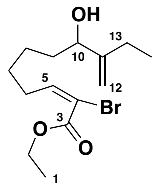

Using Still-Gennari reagent (2.0 g, 5.9 mmol), NaH (472 mg, 11.8 mmol), NBS (1.0 g, 5.9 mmol) and aldehyde **S4** (1.0 g, 5.9 mmol, prepared from 2-bromo-1-butene) afforded the title compound **S17** (410 mg, 41% over two steps).

^{1}H NMR (400 MHz, CDCl$_3$) δ_H 6.59 (t, *J* = 7.5 Hz, 1 H, *H*5) 4.94 (t, *J* = 1.0 Hz, 1 H, *H*12), 4.78 (s, 1 H, *H*12′), 4.20 (q, *J* = 7.0 Hz, 2 H, *H*2), 4.04-4.00 (m, 1 H, *H*10), 2.44 (q, *J* = 7.5 Hz, 2 H, *H*6), 2.09-1.87 (m, 2 H, *H*13), 1.52-1.35 (m, 7 H, *H*7, *H*8, *H*9, O*H*), 1.27 (t, *J* = 7.0 Hz, 3 H, *H*1), 1.01 (t, *J* = 7.5 Hz, 3 H, *H*14); **^{13}C NMR (101 MHz, CDCl$_3$)** δ_C 163.0, 153.5, 148.4, 111.1, 108.5, 75.5, 62.9, 34.2, 31.4, 28.7, 25.2, 23.9, 14.1, 12.2; **HRMS (ESI$^+$)** *m/z* calculated for $[M+Na]^+$ $C_{14}H_{23}BrNaO_3$ 341.0723, found 341.0718; **IR ν_{max}/cm^{-1} (thin film)** 3409, 2936, 1715, 1461, 1369, 1224.

Preparation of (*Z* and *E*)-ethyl 2-bromo-8-hydroxy-9-phenyldeca-2,9-dienoate, S18

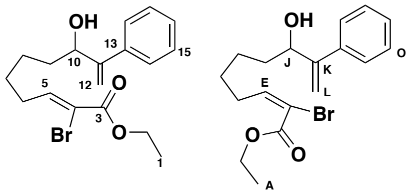

Using Horner-Wadworth-Emmons reagent (1.1 g, 4.6 mmol), NaH (368 mg, 9.2 mmol), NBS (819 g, 4.6 mmol) and aldehyde **S5** (1.0 g, 4.6 mmol, prepared from α-bromostyrene) afforded the title compounds **S18** (548 mg, 55% over two steps) as an inseparable 1:5 (*E*:*Z*) mixture of isomers.

N.B. The bromoacrylate geometric isomers were inseparable at this stage, but could be separated in sufficient quantities in the subsequent oxidation step.

^{1}H NMR (400 MHz, CDCl$_3$) δ_H 7.43-7.25 (m, 6 H_{major} + 6 H_{minor}, Ar*H*, *H*5), 6.64 (t, *J* = 7.5 Hz, 1 H_{minor}, *H*E), 5.81 (t, *J* = 1.5 Hz, 1 H_{minor}, *H*L), 5.78 (t, *J* = 1.5 Hz, 1 H_{minor}, *H*L′), 5.38 (s, 1 H_{major}, *H*12), 5.33 (s, 1 H_{major}, *H*12′), 4.66 (quin, *J* = 4.0 Hz, 1 H_{major}, *H*10), 4.29 (q, *J* = 7.0 Hz, 2 H_{major}, *H*2), 4.27 (q, *J* = 7.0 Hz, 2 H_{minor}, *H*B), 4.20 (q, *J* = 7.0 Hz, 1 H_{minor}, *H*J), 2.48 (q, *J* = 7.5 Hz, 2 H_{minor}, *H*F), 2.32 (q, *J* = 7.0 Hz, 2 H_{major}, *H*6), 1.80 (br. s, 5 H_{major} + 1 H_{minor}, O*H*), 1.68-1.39 (m, 6 H_{major} + 6 H_{minor}, *H*7, *H8*, *H*9, *H*G, *H*H, *H*I), 1.35 (t, *J* = 7.0 Hz, 3 H_{major},

*H*1), 1.31 (t, *J* = 7.0 Hz, 3 H_{minor}, *H*A); **^{13}C NMR (101 MHz, $CDCl_3$)** δ_C 166.8, 162.5, 152.0, 149.1, 148.4, 145.9, 128.4, 127.7, 126.9, 121.4, 116.5, 112.7, 111.3, 73.4, 62.4, 60.2, 35.7, 35.6, 32.0, 31.3, 28.5, 27.8, 27.4, 25.3, 25.2, 14.3, 14.1 [N.B. Some resonances are overlapping]; **HRMS (ESI$^+$)** *m/z* calculated for $[M+Na]^+$ $C_{18}H_{23}BrNaO_3$ 389.0723, found 389.0721; **IR ν_{max}/cm^{-1} (thin film)** 3436, 2936, 1714, 1625, 1493, 1444, 1368.

Preparation of (2*Z*,9*E*)-ethyl 2-bromo-8-hydroxy-10-phenyldeca-2,9-dienoate, S19

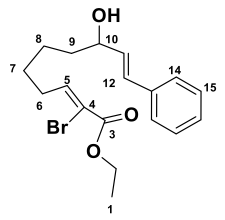

Using Horner-Wadworth-Emmons reagent (360 mg, 1.61 mmol), NaH (126 mg, 3.11 mmol), NBS (285 mg, 1.60 mmol) and aldehyde **S7** (350 mg, 1.57 mmol) afforded the title compound **S19** (400 mg, 68% over two steps) as a 3:1 (*Z*:*E*) mixture of isomers. Minor isomer not included for clarity.

^{1}H NMR (400 MHz, $CDCl_3$) δ_H 7.41 (d, *J* = 7.5 Hz, 1 H, Ar*H*), 7.36-7.30 (m, 2 H, *H*5, Ar*H*), 7.30-7.25 (m, 3 H, Ar*H*), 6.59 (d, *J* = 16.0 Hz, 1 H, *H*12), 6.24 (ddd, *J* = 16.0, 7.0, 3.0 Hz, 1 H, *H*11), 4.31-4.25 (m, 3 H, *H*2, *H*10), 2.54 (q, *J* = 7.5 Hz, 2 H, *H*6), 1.86 (br. s, 1 H, O*H*), 1.72-1.65 (m, 2 H, *H*9), 1.60-1.45 (m, 4 H, *H*7, *H*8), 1.35 (t, *J* = 7.0 Hz, 3 H *H*1); **^{13}C NMR (101 MHz, $CDCl_3$)** δ_C 163.0, 148.3, 132.4, 130.4, 128.6, 128.4, 127.7, 126.5, 116.6, 72.9, 62.4, 37.0, 32.0, 27.5, 25.2, 14.2; **HRMS (ESI$^+$)** *m/z* calculated for $[M+Na]^+$ $C_{18}H_{23}BrNaO_3$ 389.0723, found 389.0717; **IR ν_{max}/cm^{-1} (thin film)** 3480, 2933, 1712, 1623, 1493, 1448, 1368.

Preparation of (2*E*,9*E*)-ethyl 2-bromo-8-hydroxy-10-phenyldeca-2,9-dienoate, S20

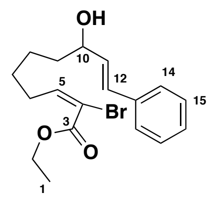

Using Still-Gennari reagent (535 mg, 1.61 mmol), NaH (126 mg, 3.11 mmol), NBS (285 mg, 1.60 mmol) and aldehyde **S7** (350 mg, 1.57 mmol) afforded the title compound **S20** (300 mg, 51% over two steps) as a single isomer.

^{1}**H NMR (400 MHz, CDCl$_3$)** δ_H 7.40-7.38 (m, 2 H, Ar*H*), 7.33 (t, *J* = 8.0 Hz, 2 H, Ar*H*), 7.26-7.20 (m, 1 H, Ar*H*), 6.67 (t, *J* = 7.5 Hz, 1 H, *H*5), 6.58 (d, *J* = 16.0 Hz, 1 H, *H*12), 6.22 (dd, *J* = 16.0, 7.0 Hz, 1 H, *H*11), 4.30-4.24 (m, 3 H, *H*2, *H*10), 2.52 (q, *J* = 7.0 Hz, 2 H, *H*6), 1.68-1.60 (m, 2 H, *H*9), 1.55-1.45 (m, 5 H, *H*7, *H*8, O*H*), 1.33 (t, *J* = 7.0 Hz, 3 H *H*1); ^{13}**C NMR (101 MHz, CDCl$_3$)** δ_C 163.0, 148.3, 136.6, 132.3, 130.5, 128.6, 127.7, 126.5, 111.3, 72.9, 62.1, 37.0, 31.3, 28.7, 25.0, 14.1; **HRMS (ESI$^+$)** *m/z* calculated for $[M+Na]^+$ $C_{18}H_{23}BrNaO_3$ 389.0723, found 389.0717; **IR ν_{max}/cm^{-1} (thin film)** 3470, 2934, 1713, 1494, 1448, 1369, 1342, 1224.

Preparation of (2*Z*,9*E*)-ethyl 2-bromo-10-(3-fluorophenyl)-8-hydroxydeca-2,9-dienoate, S21

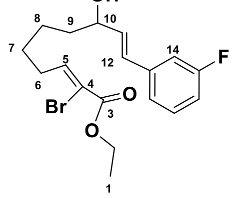

Using Horner-Wadworth-Emmons reagent (631 mg, 2.82 mmol), NaH (224 mg, 5.64 mmol), NBS (498 mg, 2.80 mmol) and aldehyde **S8** (400 mg, 2.82 mmol) afforded the title compound **S21** (410 mg, 38% over three steps), which was inseparable from an unknown impurity, so was used crude in the next reaction.

Preparation of (2*E*,9*E*)-ethyl 2-bromo-10-(3-fluorophenyl)-8-hydroxydeca-2,9-dienoate, S22

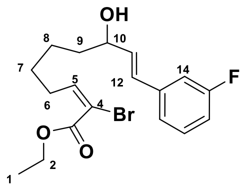

Using Still-Gennari reagent (700 mg, 2.10 mmol), NaH (170 mg, 2.10 mmol), NBS (378 mg, 2.10 mmol) and aldehyde **S8** (500 mg, 2.12 mmol) afforded the title compound **S22** (500 mg, 62% over three steps) as a single isomer.

^{1}**H NMR (400 MHz, CDCl$_3$)** δ_H 7.33-7.27 (m, 1 H, Ar*H*), 7.16 (d, *J* = 8.0 Hz, 1 H, Ar*H*), 7.10 (dt, *J* = 10.0, 2.0 Hz, 1 H, Ar*H*), 7.00-6.92 (m, 1 H, Ar*H*), 6.72-6.66 (m, 1 H, *H*5), 6.57 (d, *J* = 16.0 Hz, 1 H, *H*12), 6.25 (dd, *J* = 16.0, 6.5 Hz, 1 H, *H*11), 4.33-4.25 (m, 3 H, *H*10, *H*2), 2.57-2.51 (m, 2 H, *H*6), 1.72-1.63 (m, 2 H, *H*9), 1.56-1.50 (m, 5 H, *H*7, *H*8, O*H*), 1.35 (t, *J* = 7.0 Hz, 1 H, *H*1);^{13}**C NMR (101 MHz, CDCl$_3$)** δ_C 163.5 (d, *J* = 245.5 Hz), 162.9, 148.3, 139.0, 133.7, 130.0, 129.8, 124.1, 114.5 (d, *J* = 21.5 Hz), 112.9 (d, *J* = 21.5 Hz), 111.3, 72.6, 62.4, 38.7, 31.8, 28.7, 25.2, 14.1; ^{19}**F NMR (377 MHz, CDCl$_3$)** δ_F −113.5; **HRMS**

(ESI$^+$) *m/z* calculated for $[M+Na]^+$ $C_{18}H_{22}BrFNaO_3$ 407.0629, found 407.0622; **IR ν_{max}/cm^{-1} (thin film)** 3470, 2934, 1714, 1611, 1584, 1488, 1446, 1369.

Preparation of (2*Z*,9*E*)-ethyl 2-bromo-8-hydroxy-10-(*p*-tolyl)deca-2,9-dienoate, S23

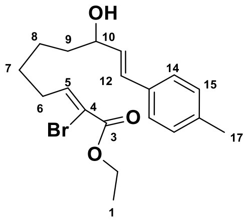

Using Horner-Wadworth-Emmons reagent (772 mg, 3.45 mmol), NaH (272 mg, 6.89 mmol), NBS (605 mg, 3.45 mmol) and aldehyde **S9** (800 mg, 3.45 mmol) afforded the title compound **S23** (760 mg, 58% over three steps) as a 2:1 mixture of brominated and non-brominated isomers. Minor isomer not included for clarity.

^{1}H NMR (400 MHz, CDCl$_3$) δ_H 7.30-7.28 (m, 3 H, *H*5, Ar*H*), 7.16-7.12 (m, 2 H, Ar*H*), 6.56 (d, *J* = 16.0 Hz, 1 H, *H*12), 6.22-6.14 (m, 1 H, *H*11), 4.32-4.25 (m, 1 H, *H*10), 4.20 (q, *J* = 7.0 Hz, 2 H, *H*2), 2.41-2.34 (m, 5 H, *H*6, *H*17), 1.76-1.60 (m, 3 H, *H*9, O*H*), 1.55-1.40 (m, 4 H, *H*7, *H*8), 1.35 (t, *J* = 7.0 Hz, 3 H *H*1); **^{13}C NMR (101 MHz, CDCl$_3$)** δ_C 166.7, 145.9, 137.6, 133.8, 131.3, 130.4, 129.3, 129.1, 116.6, 73.0, 60.2, 37.0, 31.6, 28.7, 25.2, 21.2, 14.1; **HRMS (ESI$^+$)** *m/z* calculated for $[M+Na]^+$ $C_{19}H_{25}BrNaO_3$ 403.0879, found 403.0873; **IR ν_{max}/cm^{-1} (thin film)** 3480, 2932, 2859, 1715, 1652, 1513, 1446, 1367, 1258, 1040.

Preparation of (2*E*,9*E*)-ethyl 2-bromo-8-hydroxy-10-(*p*-tolyl)deca-2,9-dienoate, S24

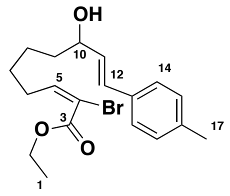

Using Still-Gennari reagent (564 mg, 1.70 mmol), NaH (138 mg, 3.40 mmol), NBS (302 mg, 1.70 mmol) and aldehyde **S9** (400 mg, 1.70 mmol) afforded the title compound **S24** (390 mg, 60% over three steps) as a single isomer.

^{1}H NMR (400 MHz, CDCl$_3$) δ_H 7.31 (d, *J* = 8.0 Hz, 2 H, Ar*H*), 7.16 (d, *J* = 8.0 Hz, 2 H, Ar*H*), 6.69 (t, *J* = 8.0 Hz, 1 H, *H*5), 6.56 (d, *J* = 16.0 Hz, 1 H, *H*12), 6.18 (dd, *J* = 16.0, 7.0 Hz, 1 H, *H*11), 4.31-4.26 (m, 3 H, *H*10, *H*2), 2.53 (q, *J* = 7.0 Hz, 2 H, *H*6), 2.37 (s, 3 H, *H*17), 1.70-1.63 (m, 3 H, *H*9, O*H*), 1.56-1.46 (m, 4 H, *H*7, *H*8), 1.35 (t, *J* = 7.0 Hz, 3 H *H*1);

^{13}**C NMR (101 MHz, $CDCl_3$)** δ_C 163.0, 148.5, 137.6, 133.8, 131.3, 130.4, 129.3, 126.4, 111.3, 73.0, 62.1, 36.9, 31.4, 28.8, 25.0, 21.2, 14.1; **HRMS (ESI$^+$)** *m/z* calculated for $[M+Na]^+$ $C_{19}H_{25}BrNaO_3$ 403.0879, found 403.0876; **IR ν_{max}/cm^{-1} (thin film)** 3480, 2933, 2859, 1716, 1513, 1369, 1224, 1027.

<u>Oxidations *(Schemes 1 and 2)*:</u>

DMP (1.5 eq.) was added to a solution of appropriate alcohol (1 eq.) in CH_2Cl_2 (4 mL/mmol), and the resulting suspension was stirred at RT overnight. The solution was diluted with CH_2Cl_2, washed with $Na_2S_2O_3$ (sat. aq.) and $NaHCO_3$ (5% aq.), and the combined aqueous layers extracted with CH_2Cl_2. The combined organic extracts were dried over $MgSO_4$, filtered and the filtrate concentrated under reduced pressure to afford the crude product. This crude product was adsorbed onto silica and purified by flash column chromatography (SiO_2, pet 40-60:Et_2O (9:1)) to afford the title compound.

Preparation of (*Z*)-ethyl 2-bromo-8-oxodeca-2,9-dienoate, (*Z*)-5a

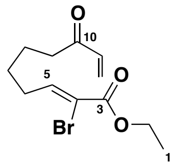

Using DMP (1.0 g, 2.6 mmol) and alcohol **S12** (500 mg, 1.7 mmol) afforded the title compound **(*Z*)-5a** (390 mg, 80%) as a colorless oil.

^{1}**H NMR (400 MHz, $CDCl_3$)** δ_H 7.27 (t, *J* = 7.5 Hz, 1 H, *H*5), 6.35 (dd, *J* = 18.0, 10.5 Hz, 1 H, *H*11), 6.22 (dd, *J* = 18.0, 2.5 Hz, 1 H, *H*12), 5.83 (dd, *J* = 10.5, 2.5 Hz, 1 H, *H*12′), 4.26 (q, *J* = 7.5 Hz, 2 H, *H*2), 2.62 (t, *J* = 7.5 Hz, 2 H, *H*9), 2.36 (q, *J* = 7.5 Hz, 2 H, *H*6), 1.69 (quin, *J* = 7.5 Hz, 2 H, *H*8), 1.53 (quin, *J* = 7.5 Hz, 2 H, *H*7), 1.32 (t, *J* = 7.5 Hz, 3 H, *H*1); ^{13}**C NMR (101 MHz, $CDCl_3$)** δ_C 200.4, 162.3, 145.5, 136.5, 128.2, 116.8, 62.4, 39.1, 31.9, 27.0, 23.4, 14.5; **HRMS (ESI$^+$)** *m/z* calculated for $[M+Na]^+$ $C_{12}H_{17}NaO_3Br$ 311.0253, found 311.0248; **IR ν_{max}/cm^{-1} (thin film)** 2937, 1722, 1681, 1620.

Preparation of (*E*)-ethyl 2-bromo-8-oxodeca-2,9-dienoate, (*E*)-5a

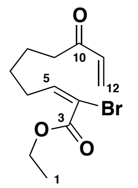

Using DMP (1.17 g, 2.76 mmol) and alcohol **S13** (533 mg, 1.85 mmol) afforded the title compound **(*E*)-5a** (395 mg, 76%) as a colorless oil.

^{1}H NMR (400 MHz, $CDCl_3$) δ_H 6.66 (t, *J* = 7.5 Hz, 1 H, *H*5), 6.35 (dd, *J* = 17.5, 10.5 Hz, 1 H, *H*11), 6.22 (dd, *J* = 17.5, 1.5 Hz, 1 H, *H*12), 5.83 (dd, *J* = 10.5, 1.5 Hz, 1 H, *H*12′), 4.27 (q, *J* = 7.5 Hz, 2 H, *H*2), 2.61 (t, *J* = 7.5 Hz, 2 H, *H*9), 2.52 (q, *J* = 7.5 Hz, 2 H, *H*6), 1.73-1.62 (m, 2 H, *H*8), 1.58-1.45 (m, 2 H, *H*7), 1.34 (t, *J* = 7.5 Hz, 3H, *H*1); **^{13}C NMR (101 MHz, $CDCl_3$)** δ_C 200.4, 162.9, 148.0, 136.5, 128.1, 111.5, 62.1, 39.1, 31.3, 28.3, 23.4, 14.1; **HRMS (ESI$^+$)** *m/z* calculated for $[M+Na]^+$ $C_{12}H_{17}NaO_3Br$ 311.0253, found 311.0249; **IR ν_{max}/cm^{-1} (thin film)** 2937, 1715, 1682, 1615.

Preparation of (*Z*)-ethyl 2-bromo-9-methyl-8-oxodeca-2,9-dienoate (*Z*)-5b

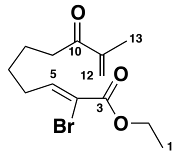

Using DMP (1.68 g, 3.96 mmol) and alcohol **S14** (803 mg, 2.66 mmol) afforded the title compound **(*Z*)-5b** (745 mg, 95%) as a colorless oil.

^{1}H NMR (400 MHz, $CDCl_3$) δ_H 7.38 (t, *J* = 7.0 Hz, 1 H, *H*5), 5.96 (s, 1 H, *H*12), 5.78 (s, 1 H, *H*12′), 4.27 (q, *J* = 7.0 Hz, 2 H, *H*2), 2.72 (t, *J* = 7.0 Hz, 2 H, *H*9), 2.37 (q, *J* = 7.0 Hz, 2 H, *H*6), 1.88 (s, 3 H, *H*13), 1.69 (quin, *J* = 7.0 Hz, 2 H, *H*8), 1.54 (quin, *J* = 7.0 Hz, 2 H, *H*7), 1.33 (t, *J* = 7.0 Hz, 3 H, *H*1); **^{13}C NMR (126 MHz, $CDCl_3$)** δ_C 201.6, 162.4, 148.0, 144.5, 124.5, 116.7, 62.6, 36.9, 31.9, 27.2, 24.0, 17.6, 14.1; **HRMS (ESI$^+$)** *m/z* calculated for $[M+Na]^+$ $C_{13}H_{19}BrNaO_3$ 325.0410, found 325.0409; **IR ν_{max}/cm^{-1} (thin film)** 2931, 1724, 1676, 1625, 1452, 1367.

Preparation of (*E*)-ethyl 2-bromo-9-methyl-8-oxodeca-2,9-dienoate (*E*)-5b

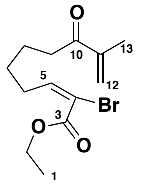

Using DMP (965 mg, 2.27 mmol) and alcohol **S15** (461 mg, 1.52 mmol) afforded the title compound **(*E*)-5b** (295 mg, 65%) as a colorless oil.

^{1}H NMR (400 MHz, $CDCl_3$) δ_H 6.59 (t, *J* = 7.5 Hz, 2 H, *H*5), 5.90 (s, 1 H, *H*12), 5.71 (s, 1 H, *H*12′), 4.20 (q, *J* = 7.0 Hz, 2 H, *H*2), 2.63 (t, *J* = 7.5 Hz, 2 H, *H*9), 2.45 (q, *J* = 7.5 Hz, 2 H, *H*6), 1.80 (s, 3 H, *H*13), 1.65-1.55 (m, 2 H, *H*8), 1.50-1.38 (m, 2 H, *H*7), 1.27 (t, *J* = 7.0 Hz, 3 H, *H*1); **^{13}C NMR (126 MHz, $CDCl_3$)** δ_C 201.7, 162.9, 148.1, 145.6, 124.5,

111.5, 62.4, 36.9, 31.9, 28.4, 24.0, 17.6, 14.1; **HRMS (ESI$^+$)** *m/z* calculated for $[M+Na]^+$ $C_{13}H_{19}BrNaO_3$ 325.0410, found 325.0417; **IR ν_{max}/cm^{-1} (thin film)** 2930, 1715, 1676, 1629, 1452, 1369.

Preparation of (*E*)-ethyl 2-bromo-9-ethyl-8-oxodeca-2,9-dienoate (*E*)-5c and (*Z*)-ethyl 2-bromo-9-ethyl-8-oxodeca-2,9-dienoate (*Z*)-5c

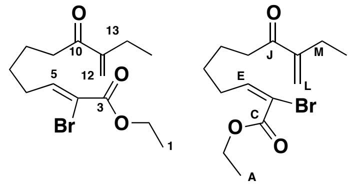

Using DMP (806 mg, 1.90 mmol) and alcohol mixture **S16** (403 mg, 1.27 mmol), SiO_2 purification afforded the single *E* isomer **(*E*)-5c** (220 mg, 55%) as a colorless oil, and a 3:1 *Z*:*E* mixture of isomers **(*Z*)-5c / (*E*)-5c** (36 mg, 9%).

***E* isomer:**

^{1}H NMR (400 MHz, $CDCl_3$) δ_H 6.59 (t, *J* = 7.5 Hz, 1 H, *H*E), 5.90 (s, 1 H, *H*L), 5.65 (s, 1 H, *H*L′), 4.20 (q, *J* = 7.0 Hz, 2 H, *H*B), 2.63 (t, *J* = 7.5 Hz, 2 H, *H*I), 2.45 (q, *J* = 7.5 Hz, 2 H, *H*F), 2.22 (q, *J* = 7.5 Hz, 2 H, *H*M), 1.62-1.54 (m, 2 H, *H*H), 1.46-1.39 (m, 2 H, *H*G), 1.27 (t, *J* = 7.0 Hz, 3 H, *H*A), 0.96 (t, *J* = 7.5 Hz, 3 H, *H*N); **^{13}C NMR (126 MHz, $CDCl_3$)** δ_C 201.7, 162.9, 150.4, 148.1, 122.7, 111.5, 62.1, 37.4, 31.2, 28.4, 23.9, 23.8, 14.1, 12.6; **HRMS (ESI$^+$)** *m/z* calculated for $[M+Na]^+$ $C_{14}H_{21}BrNaO_3$ 339.0566, found 339.0575; **IR ν_{max}/cm^{-1} (thin film)** 2935, 1716, 1676, 1459, 1369, 1223.

3:1 *Z:E* isomer mixture, *E* isomer data omitted for clarity:

^{1}H NMR (400 MHz, $CDCl_3$) δ_H 7.30 (t, *J* = 7.5 Hz, 1 H, *H*5), 6.00 (s, 1 H, *H*12), 5.72 (s, 1 H, *H*12′), 4.26 (q, *J* = 7.5 Hz, 2 H, *H*2), 2.73 (t, *J* = 7.0 Hz, 2 H, *H*9), 2.39 (q, *J* = 7.5 Hz, 2 H, *H*6), 2.31 (q, *J* = 7.5 Hz, 2 H, *H*13), 1.74-1.64 (m, 2 H, *H*8), 1.59-1.47 (m, 2 H, *H*7), 1.36 (t, *J* = 7.5 Hz, 3 H, *H*1), 1.05 (t, *J* = 7.5 Hz, 3 H, *H*14); **^{13}C NMR (126 MHz, $CDCl_3$)** δ_C 201.7, 162.9, 150.4, 148.6, 145.6, 122.7, 62.4, 37.3, 31.9, 28.4, 23.9, 23.8, 14.1, 12.6; **HRMS (ESI$^+$)** *m/z* calculated for $[M+Na]^+$ $C_{14}H_{21}BrNaO_3$ 339.0566, found 339.0573; **IR ν_{max}/cm^{-1} (thin film)** 2936, 1724, 1676, 1625, 1459, 1368.

Preparation of (*E*)-ethyl 2-bromo-9-ethyl-8-oxodeca-2,9-dienoate (*E*)-5c

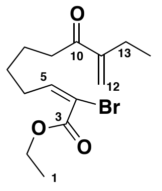

Using DMP (820 mg, 1.93 mmol) and alcohol **S17** (410 mg, 1.29 mmol) afforded the title compound **(*E*)-5c** (275 mg, 67%) as a colorless oil.

^{1}H NMR (400 MHz, CDCl$_3$) δ_H 6.59 (t, *J* = 7.5 Hz, 1 H, *H*5), 5.90 (s, 1 H, *H*12), 5.65 (s, 1 H, *H*12′), 4.20 (q, *J* = 7.0 Hz, 2 H, *H*2), 2.63 (t, *J* = 7.5 Hz, 2 H, *H*9), 2.45 (q, *J* = 7.5 Hz, 2 H, *H*6), 2.22 (q, *J* = 7.5 Hz, 2 H, *H*13), 1.62-1.54 (m, 2 H, *H*8), 1.46-1.39 (m, 2 H, *H*7), 1.27 (t, *J* = 7.0 Hz, 3 H, *H*1), 0.96 (t, *J* = 7.5 Hz, 3 H, *H*14); **^{13}C NMR (126 MHz, CDCl$_3$)** δ_C 201.7, 162.9, 150.4, 148.1, 122.7, 111.5, 62.1, 37.4, 31.2, 28.4, 23.9, 23.8, 14.1, 12.6; **HRMS (ESI$^+$)** *m/z* calculated for $[M+Na]^+$ $C_{14}H_{21}BrNaO_3$ 339.0566, found 339.0575; **IR ν_{max}/cm^{-1} (thin film)** 2935, 1716, 1676, 1459, 1369, 1223.

Preparation of (*Z and E*)-ethyl 2-bromo-8-oxo-9-phenyldeca-2,9-dienoate (*Z*)-5d /(*E*)-5d

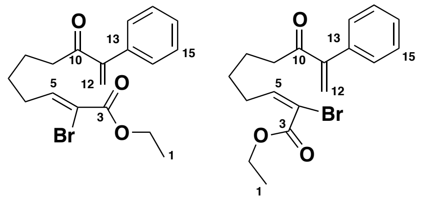

Using DMP (950 mg, 2.24 mmol) and alcohol mixture **S18** (548 mg, 1.50 mmol) afforded, separately upon chromatography, the *Z* isomer **(*Z*)-5d** (350 mg, 64%) as a colorless oil, and the *E* isomer **(*E*)-5d** (80 mg, 15%) as a colorless oil.

***Z* isomer (*Z*)-5d:**

^{1}H NMR (400 MHz, CDCl$_3$) δ_H 7.41-7.36 (m, 3 H, Ar*H*) 7.33-7.29 (m, 3 H, Ar*H*, *H*5), 6.14 (s, 1 H, *H*12), 5.92 (s, 1 H, *H*12′), 4.30 (q, *J* = 7.0 Hz, 2 H, *H*2), 2.80 (t, *J* = 7.0 Hz, 2 H, *H*9), 2.39 (q, *J* = 7.5 Hz, 2 H, *H*6), 1.75 (quin, *J* = 7.5 Hz, 2 H, *H*8), 1.57 (quin, *J* = 7.5 Hz, 2 H, *H*7), 1.36 (t, *J* = 7.0 Hz, 3 H, *H*1); **^{13}C NMR (126 MHz, CDCl$_3$)** δ_C 201.7, 162.5, 149.5, 145.5, 137.2, 128.3, 128.2, 128.1, 124.4, 116.8, 62.4, 39.2, 31.9, 28.3, 23.9, 14.2; **HRMS (ESI$^+$)** *m/z* calculated for $[M+Na]^+$ $C_{18}H_{21}BrNaO_3$ 387.0566, found 387.0574; **IR ν_{max}/cm^{-1} (thin film)** 2936, 1721, 1624, 1494, 1445, 1367.

***E* isomer (*E*)-5d:**

^{1}H NMR (400 MHz, CDCl$_3$) δ_H 7.43-7.30 (m, 5 H, Ar*H*), 6.68 (t, *J* = 7.5 Hz, 1 H, *H*5), 6.13 (s, 1 H, *H*12), 5.91 (s, 1 H, *H*12′), 4.29 (q, *J* = 7.0 Hz, 2 H, *H*2), 2.78 (t, *J* = 7.5 Hz, 2 H, *H*9), 2.55 (q, *J* = 7.5 Hz, 2 H, *H*6), 1.72 (quin, *J* = 7.5 Hz, 2 H, *H*8), 1.52 (quin, *J* = 7.5 Hz, 2 H, *H*7), 1.36 (t, *J* = 7.0 Hz, 3 H, *H*1); **^{13}C NMR (126 MHz, CDCl$_3$)** δ_C 201.8, 162.9, 149.5, 148.4, 137.2, 128.3, 128.2, 128.1, 124.3, 111.5, 62.1, 36.2, 31.9, 28.3, 23.9, 14.2; **HRMS (ESI$^+$)** *m/z* calculated for $[M+Na]^+$ $C_{18}H_{21}BrNaO_3$ 387.0566, found 387.0572; **IR ν_{max}/cm^{-1} (thin film)** 2937, 1715, 1612, 1494, 1445, 1369.

Preparation of (2*Z*,9*E*)-ethyl 2-bromo-8-oxo-10-phenyldeca-2,9-dienoate, (*Z*)-5e

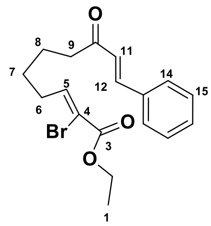

Using DMP (695 mg, 1.64 mmol) and alcohol **S19** (400 mg, 1.10 mmol) afforded the title compound **(*Z*)-5e** (350 mg, 88%*) as a 5:1 mixture with the non-brominated substrate. Minor contaminant not included for clarity.

^{1}H NMR (400 MHz, CDCl$_3$) δ_H 7.61-7.57 (m, 3 H, *H*12, Ar*H*), 7.44-7.42 (m, 2 H, Ar*H*), 7.32 (m, 2 H, *H*5, Ar*H*), 6.77 (d, *J* = 16.0 Hz, 1 H, *H*11), 4.30 (q, *J* = 7.0 Hz, 2 H, *H*2), 2.74 (t, *J* = 7.5 Hz, 2 H, *H*9), 2.42 (q, *J* = 7.5 Hz, 2 H, *H*6), 1.82-1.75 (m, 2 H, *H*8), 1.65-1.59 (m, 2 H, *H*7), 1.35 (t, *J* = 7.0 Hz, 3 H, *H*1); **^{13}C NMR (101 MHz, CDCl$_3$)** δ_C 200.0, 162.5, 145.6, 142.6, 134.5, 130.5, 128.9, 128.3, 126.1, 116.8, 62.4, 40.5, 32.0, 28.3, 23.8, 14.3; **HRMS (ESI$^+$)** *m/z* calculated for $[M+Na]^+$ $C_{18}H_{21}NaO_3Br$ 387.0566, found 387.0560; **IR ν_{max}/cm^{-1} (thin film)** 2936, 1718, 1690, 1662, 1611, 1576, 1495, 1449.

Preparation of (2*E*,9*E*)-ethyl 2-bromo-8-oxo-10-phenyldeca-2,9-dienoate, (*E*)-5e

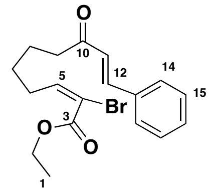

Using DMP (524 mg, 1.24 mmol) and alcohol **S20** (300 mg, 0.82 mmol) afforded the title compound **(*E*)-5e** (250 mg, 83%) as a single isomer.

^{1}H NMR (400 MHz, $CDCl_3$) δ_H 7.60-7.55 (m, 3 H, *H*12, Ar*H*), 7.43-7.40 (m, 3 H, Ar*H*), 6.77 (d, *J* = 16.0 Hz, 1 H, *H*11), 6.70 (t, *J* = 7.5 Hz, 1 H, *H*5), 4.29 (q, *J* = 7.0 Hz, 2 H, *H*2), 2.72 (t, *J* = 7.0 Hz, 2 H, *H*9), 2.57 (q, *J* = 7.5 Hz, 2 H, *H*6), 1.79-1.72 (m, 2 H, *H*8), 1.60-1.52 (m, 2 H, *H*7), 1.36 (t, *J* = 7.0 Hz, 3 H, *H*1); **^{13}C NMR (101 MHz, $CDCl_3$)** δ_C 200.0, 162.9, 148.0, 142.6, 134.5, 130.5, 129.0, 128.3, 126.2, 111.5, 62.4, 40.4, 31.2, 28.3, 23.8, 14.1; **HRMS (ESI$^+$)** *m/z* calculated for $[M+Na]^+$ $C_{18}H_{21}NaO_3Br$ 387.0566, found 387.0560; **IR ν_{max}/cm^{-1} (thin film)** 2937, 1715, 1663, 1610, 1577, 1495, 1449, 1369.

Preparation of (2*Z*,9*E*)-ethyl 2-bromo-10-(3-fluorophenyl)-8-oxodeca-2,9-dienoate, (*Z*)-5f

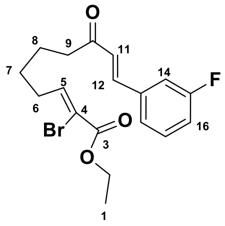

Using DMP (331 mg, 0.78 mmol) and alcohol **S21** (200 mg, 0.52 mmol) afforded the title compound **(*Z*)-5f** (120 mg, 60%), contaminated with <5% of an unknown impurity.

^{1}H NMR (500 MHz, $CDCl_3$) δ_H 7.53 (d, *J* = 16.0 Hz, 1 H, *H*12) 7.41-7.37 (m, 1 H, Ar*H*), 7.35-7.30 (m, 2 H, Ar*H*, *H*5), 7.28-7.25 (m, 1 H, Ar*H*), 7.12 (td, *J* = 8.0, 2.5 Hz, 1 H, Ar*H*), 6.75 (d, *J* = 16.0 Hz, 1 H, *H*11), 4.29 (q, *J* = 7.0 Hz, 2 H, *H*2), 2.72 (t, *J* = 7.5 Hz, 2 H, *H*9), 2.42 (q, *J* = 7.5 Hz, 2 H, *H*6), 1.77 (quin, 2 H, *J* = 7.5 Hz, *H*8), 1.64-1.57 (m, 2 H, *H*7), 1.35 (t, *J* = 7.0 Hz, 3 H, *H*1); **^{13}C NMR (126 MHz, $CDCl_3$)** δ_C 199.6, 162.9 (d, *J* = 247.0 Hz), 162.5, 145.5, 141.1, 136.8, 130.5, 127.1, 124.4, 117.4 (d, *J* = 22.0 Hz), 116.8, 114.4 (d, *J* = 22.0 Hz), 62.5, 40.7, 31.9, 27.1, 23.7, 14.2; **^{19}F NMR (377 MHz, $CDCl_3$)** δ_F -112.4; **HRMS (ESI$^+$)** *m/z* calculated for $[M+H]^+$ $C_{18}H_{21}O_3BrFNa$ 405.0472, found 405.0465; **IR ν_{max}/cm^{-1} (thin film)** 2938, 1719, 1667, 1616, 1583, 1487, 1447, 1368, 1257.

Preparation of (2*E*,9*E*)-ethyl 2-bromo-10-(3-fluorophenyl)-8-oxodeca-2,9-dienoate, (*E*)-5f

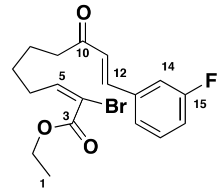

Using DMP (663 mg, 1.56 mmol) and alcohol **S22** (400 mg, 1.04 mmol) afforded the title compound **(*E*)-5f** (350 mg, 88%) as a single isomer.

^{1}H NMR (400 MHz, CDCl$_3$) δ_H 7.52 (d, *J* = 16.0 Hz, 1 H, *H*12) 7.40-7.32 (m, 2 H, Ar*H*), 7.30-7.25 (m, 1 H, Ar*H*), 7.11 (tdd, *J* = 8.0, 2.5, 1.0 Hz, 1 H, Ar*H*), 6.73 (d, *J* = 16.0 Hz, 1 H, *H*11), 6.69 (t, *J* = 7.5 Hz, 1 H, *H*5), 4.28 (q, *J* = 7.0 Hz, 2 H, *H*2), 2.70 (t, *J* = 7.0 Hz, 2 H, *H*9), 2.57 (q, *J* = 7.5 Hz, 2 H, *H*6), 1.78-1.70 (m, 2 H, *H*8), 1.59-1.50 (m, 2 H, *H*7), 1.36 (t, *J* = 7.0 Hz, 3 H, *H*1); **^{13}C NMR (101 MHz, CDCl$_3$)** δ_C 199.6, 162.9, 163.1 (d, *J* = 247.0 Hz), 148.0, 141.2, 136.9, 130.5, 127.2, 124.3, 117.3 (d, *J* = 20.0 Hz), 114.2 (d, *J* = 22.0), 111.5, 62.4, 40.6, 31.1, 28.3, 23.6, 14.1; **^{19}F NMR (377 MHz, CDCl$_3$)** δ_F −112.4; **HRMS (ESI$^+$)** *m/z* calculated for $[M+H]^+$ $C_{18}H_{21}O_3BrF$ 383.0653, found 383.0647; **IR ν_{max}/cm^{-1} (thin film)** 2937, 1714, 1666, 1613, 1582, 1485, 1447, 1369.

Preparation of (2*Z*,9*E*)-ethyl 2-bromo-8-oxo-10-(*p*-tolyl)deca-2,9-dienoate, (*Z*)-5g

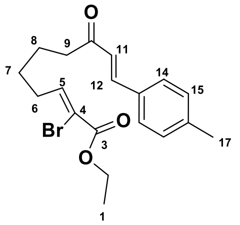

Using DMP (642 mg, 1.52 mmol) and alcohol **S23** (400 mg, 1.01 mmol) afforded the title compound **(*Z*)-5g** (322 mg, 84%) contaminated with <5% of an unknown impurity.

^{1}H NMR (500 MHz, CDCl$_3$) δ_H 7.56 (d, *J* = 16.0 Hz, 1 H, *H*12), 7.47 (d, *J* = 8.0 Hz, 2 H, Ar*H*), 7.31 (t, 1 H, *J* = 7.0 Hz, *H*5), 7.23 (d, *J* = 8.0 Hz, 2 H, Ar*H*), 6.73 (d, *J* = 16.0 Hz, 1 H, *H*11), 4.29 (q, *J* = 7.0 Hz, 2 H, *H*2), 2.72 (t, *J* = 7.5 Hz, 2 H, *H*9), 2.42 (t, *J* = 7.5 Hz, 2 H, *H*6), 2.41 (s, 3 H, *H*17), 1.77 (quin, 2 H, *J* = 7.5 Hz, *H*8), 1.63-1.55 (m, 2 H, *H*7), 1.35 (t, *J* = 7.0 Hz, 3 H, *H*1); **^{13}C NMR (126 MHz, CDCl$_3$)** δ_C 200.1, 162.5, 145.6, 142.7, 141.0, 131.7, 129.7, 128.3, 125.2, 116.8, 62.4, 40.3, 31.9, 27.2, 23.9, 21.5, 14.2; **HRMS (ESI$^+$)** *m/z* calculated for $[M+Na]^+$ $C_{19}H_{23}NaO_3Br$ 401.0723, found 401.0718; **IR ν_{max}/cm^{-1} (thin film)** 2936, 1717, 1655, 1606, 1513, 1368, 1259, 1180, 1041.

Preparation of (2*E*,9*E*)-ethyl 2-bromo-8-oxo-10-(*p*-tolyl)deca-2,9-dienoate, (*E*)-5g

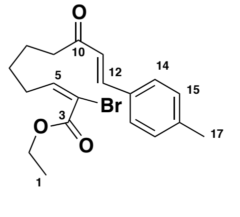

Using DMP (321 mg, 0.75 mmol) and alcohol **S24** (200 mg, 0.51 mmol) afforded the title compound **(*E*)-5g** (180 mg, 93%) as a single isomer.

^{1}H NMR (400 MHz, CDCl$_3$) δ_H 7.56 (d, *J* = 16.0 Hz, 1 H, *H*12), 7.48 (d, *J* = 8.0 Hz, 2 H, Ar*H*), 7.23 (d, *J* = 8.0 Hz, 2 H, Ar*H*), 6.72 (d, *J* = 16.0 Hz, 1 H, *H*11), 6.70 (d, *J* = 7.5 Hz,

1 H, *H*5), 4.30 (q, *J* = 7.0 Hz, 2 H, *H*2), 2.71 (t, *J* = 7.0 Hz, 2 H, *H*9), 2.57 (q, *J* = 7.5 Hz, 2 H, *H*6), 2.41 (s, 3 H, *H*17), 1.79-1.70 (m, 2 H, *H*8), 1.60-1.52 (m, 2 H, *H*7), 1.37 (t, *J* = 7.0 Hz, 3 H, *H*1); **^{13}C NMR (101 MHz, $CDCl_3$)** δ_C 200.1, 162.9, 148.2, 142.6, 141.0, 131.7, 129.7, 128.3, 125.2, 111.5, 62.1, 40.3, 31.2, 28.4, 27.2, 21.5, 14.1; **HRMS (ESI$^+$)** *m/z* calculated for $[M+Na]^+$ $C_{19}H_{23}NaO_3Br$ 401.0723, found 401.0715; **IR ν_{max}/cm^{-1} (thin film)** 2935, 1715, 1658, 1606, 1512, 1458, 1368, 1339, 1222, 1179, 1029.

1.3.2 Base-promoted Cyclizations

Two different cyclization conditions were carried out on each substrate.

General Procedure A

Phase-transfer catalyst (PTC) (10 mol%) was added to a solution of the cyclization precursor (1 eq.) in toluene (~24 mL/mmol), and the resulting solution was cooled to the appropriate temperature. Powdered KOH(s) (4 eq.) was added and the resulting solution stirred until the reaction was complete (determined by TLC). The mixture was quenched with NH_4Cl (sat. aq.) and allowed to warm to RT if appropriate. The layers were separated and the aqueous layer was extracted with CH_2Cl_2. The combined organics were washed with brine, dried over $MgSO_4$, filtered and the filtrate concentrated under reduced pressure to afford the crude product. The crude product was adsorbed onto silica and purified by flash column chromatography (SiO_2, pet 40-60:Et_2O (8:2)) to afford the cyclised product.

General Procedure B

NaH (60% w/w dispersion in mineral oil, 2 eq.) was added to a solution of the cyclization precursor (1 eq.) in THF (~24 mL/mmol), and the resulting solution stirred until the reaction was complete (determined by TLC). The solution was quenched with NH_4Cl (sat. aq.) and allowed to warm to RT if appropriate. The layers were separated and the aqueous layer was extracted with CH_2Cl_2. The combined organics were washed with brine, dried over $MgSO_4$, filtered and the filtrate concentrated under reduced pressure to afford the crude product. The crude product was adsorbed onto silica and purified by flash column chromatography (SiO_2, pet 40-60:Et_2O (8:2)) to afford the cyclised product.

Preparation of (1a*S, 1b*R**, 4a*S**, 5a*R**)-ethyl 5-oxooctahydro-1*H*-cyclopropa[*a*]pentalene-1*a*-carboxylate, 19**

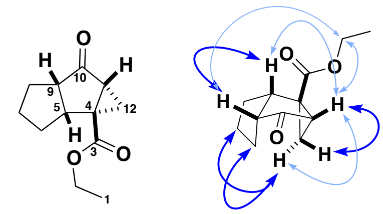

Following **General Procedure A**, using cyclization precursor **(*Z*)-5a** (50 mg, 0.17 mmol), toluene (4 mL), KOH (39 mg, 0.68 mmol), tetrabutylammonium bisulfate (6 mg, 0.02 mmol) at RT afforded compound **19** (25 mg, 71%).

Following **General Procedure A**, using cyclization precursor **(*E*)-5g** (50 mg, 0.17 mmol), toluene (4 mL), KOH (39 mg, 0.68 mmol), tetrabutylammonium bisulfate (6 mg, 0.02 mmol) at RT afforded compound **19** (29 mg, 83%).

Following **General Procedure B**, using cyclization precursor, **(*Z*)-5a** (50 mg, 0.17 mmol), THF (4 mL), NaH (7 mg, 0.17 mmol) afforded compound **19** (28 mg, 80%).

N.B.: Key nOe interactions are highlighted. Bold dark blue arrows indicate a strong correlation and light blue arrows indicate a weak correlation.

^{1}H NMR (500 MHz, CDCl$_3$) δ_H 4.17 (q, *J* = 7.0 Hz, 2 H, *H*2), 3.60-3.50 (m, 1 H, *H*5), 2.87 (dd, *J* = 10.5, 9.5 Hz, 1 H, *H*9), 2.44 (ddd, *J* = 10.0, 4.5, 1.0 Hz, 1 H, *H*11), 2.10-2.05 (m, 1 H, *H*6), 1.92-1.73 (m, 3 H, *H*7, *H*8, *H*12), 1.67-1.57 (m, 2 H, *H*6', *H*7'), 1.51-1.42 (m, 1 H, *H*8'), 1.39 (t, *J* = 4.5 Hz, 1 H, *H*12'), 1.26 (t, *J* = 7.0 Hz, 3 H, *H*1); **^{13}C NMR (126 MHz, CDCl$_3$)** δ_C 213.2, 172.1, 61.1, 54.0, 40.2, 40.1, 37.7, 30.9, 30.2, 29.5, 18.5, 14.2; **HRMS (ESI$^+$)** *m/z* calculated for $[M+Na]^+$ $C_{12}H_{16}NaO_3$ 231.0992, found 231.0986; **IR ν_{max}/cm^{-1} (thin film)** 2929, 2855, 1730, 1670.

Preparation of (1a*R, 1b*R**, 4a*S**, 5a*S**)-ethyl 5-oxooctahydro-1*H*-cyclopropa[*a*]pentalene-1*a*-carboxylate, 12**

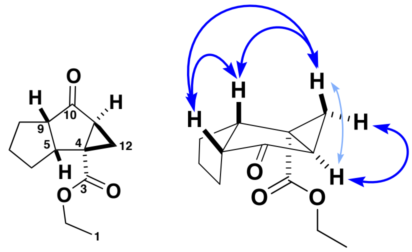

Following **General Procedure B**, using cyclization precursor **(*E*)-5a** (50 mg, 0.17 mmol), THF (4 mL), NaH (7 mg, 0.17 mmol) afforded compound **12** (26 mg, 65%) as a colorless oil.

N.B.: Key nOe interactions are highlighted. Bold dark blue arrows indicate a strong correlation and light blue arrows indicate a weak correlation.

^{1}H NMR (500 MHz, CDCl$_3$) δ_H 4.16 (q, *J* = 7.0 Hz, 2 H, *H*2), 2.90 (q, *J* = 7.5 Hz, 1 H, *H*5), 2.56 (q, *J* = 7.5 Hz, 1 H, *H*9), 2.42 (dd, *J* = 9.5, 4.0 Hz, 1 H, *H*11), 2.23 (ddd, *J* = 13.5, 7.0, 4.0 Hz, 1 H, *H*6), 1.82 (dt, *J* = 7.5, 2.0 Hz, 2 H, *H*8), 1.76 (dd, *J* = 4.5, 1.0 Hz, 1 H, *H*12), 1.71-1.63 (m, 1 H, *H*7), 1.55-1.46 (m, 1 H, *H*7′), 1.38 (t, *J* = 4.5 Hz, 1 H, *H*12′), 1.3 (m, 1 H, *H*6′), 1.26 (t, *J* = 7.0 Hz, 3 H, *H*1); **^{13}C NMR (126 MHz, CDCl$_3$)** δ_C 214.0, 171.0, 60.9, 48.6, 43.0, 36.7, 34.8, 31.9, 26.7, 26.6, 22.7, 14.2; **HRMS (ESI$^+$)** *m/z* calculated for $[M+Na]^+$ $C_{12}H_{16}NaO_3$ 231.0992, found 231.0986; **IR ν_{max}/cm^{-1} (thin film)** 2929, 2855, 1730, 1670.

Preparation of (1a*S,1b*R**,4a*S**,5a*R**)-ethyl 5a-methyl-5-oxooctahydro-1*H*-cyclopropa[*a*]-pentalene-1a-carboxylate, 20**

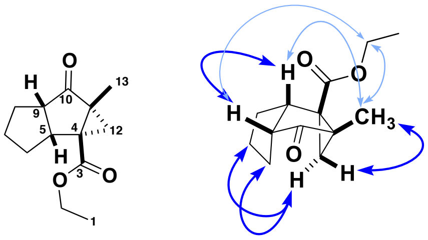

Following **General Procedure A**, using cyclization precursor **(*Z*)-5b** (50 mg, 0.17 mmol), toluene (4 mL), KOH (37 mg, 0.66 mmol), tetrabutylammonium bisulfate (6 mg, 0.02 mmol) at RT afforded compound **20** (27 mg, 71%) as a colorless oil.

Following **General Procedure A**, using cyclization precursor **(*E*)-5b** (50 mg, 0.17 mmol), toluene (4 mL), KOH (37 mg, 0.66 mmol), tetrabutylammonium bisulfate (6 mg, 0.02 mmol) at RT afforded compound **20** (30 mg, 79%) as a colorless oil.

Following **General Procedure B**, using cyclization precursor **(*Z*)-5b** (50 mg, 0.17 mmol), THF (4 mL), NaH (7 mg, 0.17 mmol) afforded compound **20** (28 mg, 74%) as a colorless oil.

N.B.: Key nOe interactions are highlighted. Bold dark blue arrows indicate a strong correlation and light blue arrows indicate a weak correlation. Stereochemical analysis similar to compound **19**.

^{1}H NMR (500 MHz, CDCl$_3$) δ_H 4.26-4.15 (m, 2 H, *H*2), 3.62-3.55 (m, 1 H, *H*5), 2.82 (q, *J* = 9.5 Hz, 1 H, *H*9), 2.06-1.99 (m, 1 H, *H*6), 1.96-1.89 (m, 1 H, *H*8), 1.84-1.76 (m, 1 H, *H*7), 1.69 (dd, *J* = 5.0, 1.5 Hz, 1 H, *H*12), 1.61 (s, 1 H, *H*7′), 1.57-1.50 (m, 1 H, *H*6′), 1.47-1.41 (m, 1 H, *H*8′), 1.39 (d, 1 H, *J* = 5.0 Hz, 1 H, *H*12′), 1.36 (s, 3 H, *H*13), 1.29 (t, *J* = 7.0 Hz, 3 H, *H*1); **^{13}C NMR (126 MHz, CDCl$_3$)** δ_C 214.9, 171.3, 61.0, 52.6, 45.9, 42.5, 39.9, 30.4, 30.2, 29.4, 23.5, 14.3, 11.7; **HRMS (ESI$^+$)** *m/z* calculated for $[M+Na]^+$ $C_{13}H_{18}NaO_3$ 245.1148, found 245.1158; **IR ν_{max}/cm^{-1} (thin film)** 2960, 1719, 1451, 1374, 1246.

Preparation of (1a*R,1b*R**,4a*S**,5a*S**)-ethyl 5a-methyl-5-oxooctahydro-1*H*-cyclopropa[*a*]-pentalene-1a-carboxylate, 13**

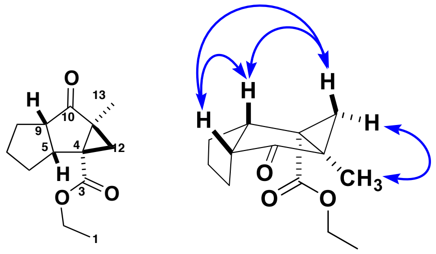

Following **General Procedure B**, using cyclization precursor **(*E*)-5b** (50 mg, 0.17 mmol), THF (4 mL), NaH (7 mg, 0.17 mmol) afforded compound **13** (29 mg, 74%) as a colorless oil.

N.B.: Key nOe interactions are highlighted. Bold dark blue arrows indicate a strong correlation. Stereochemical analysis similar to compound **12**.

^{1}H NMR (500 MHz, CDCl$_3$) δ_H 4.17-4.10 (m, 2 H, *H*2), 2.76 (dd, *J* = 9.0, 7.5 Hz, 1 H, *H*5), 2.49 (dd, *J* = 7.5, 6.5 Hz, 1 H, *H*9), 2.19 (dtd, *J* = 13.0, 6.5, 3.5 Hz, 1 H, *H*6), 1.78-1.74 (m, 2 H, *H*8), 1.60-1.58 (m, 1 H, *H*12), 1.55-1.45 (m, 2 H, *H*7), 1.36 (d, *J* = 4.5 Hz, 1 H, *H*12′), 1.37 (s, 3 H, *H*13), 1.24 (t, *J* = 7.0 Hz, 3 H, *H*1), 1.18-1.10 (m, 1 H, *H*6′); **^{13}C NMR (126 MHz, CDCl$_3$)** δ_C 215.4, 171.4, 60.8, 47.9, 43.8, 40.4, 40.6, 32.6, 26.7, 26.6, 26.5, 14.2, 11.2; **HRMS (ESI$^+$)** *m/z* calculated for $[M+Na]^+$ $C_{13}H_{18}NaO_3$ 245.1148, found 245.1153; **IR ν_{max}/cm^{-1} (thin film)** 2960, 1724, 1372, 1241, 1185.

Preparation of (1a*S,1b*R**,4a*S**,5a*R**)-ethyl 5a-ethyl-5-oxooctahydro-1*H*-cyclopropa[*a*]-pentalene-1a-carboxylate, 21**

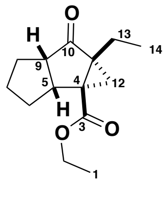

Following **General Procedure A**, using cyclization precursor **(*Z*)-5c** (50 mg, 0.16 mmol), toluene (4 mL), KOH (35 mg, 0.63 mmol), tetrabutylammonium bisulfate (5 mg, 0.02 mmol) at RT afforded compound **21** (25 mg, 63%) as a colorless oil.

Following **General Procedure A**, using cyclization precursor **(*E*)-5c** (50 mg, 0.16 mmol), toluene (4 mL), KOH (35 mg, 0.63 mmol), tetrabutylammonium bisulfate (5 mg, 0.02 mmol) at RT afforded compound **21** (29 mg, 76%) as a colorless oil.

Following **General Procedure B**, using cyclization precursor **(*Z*)-5c** (50 mg, 0.16 mmol), THF (4 mL), NaH (6 mg, 0.16 mmol) afforded compound **21** (30 mg, 77%) as a colorless oil. N.B. Stereochemistry assigned by nOe analysis similar to compound **19**.

^{1}H NMR (500 MHz, CHCl$_3$) δ_H 4.19-4.08 (m, 2 H, *H*2), 3.56-3.49 (m, 1 H, *H*5), 2.76-2.69 (m, 1 H, *H*9), 2.08 (sext, *J* = 7.5 Hz, 1 H, *H*13), 1.97-1.90 (m, 1 H, *H*6), 1.86-1.80 (m, 1 H, *H*8), 1.72-1.67 (m, 1 H, *H*7), 1.66 (dd, *J* = 5.0, 1.5 Hz, 1 H, *H*12), 1.58-1.50 (m, 1 H, *H*7′), 1.48-1.37 (m, 3 H, *H*6′, *H*8′, *H*13′), 1.25 (d, *J* = 5.0 Hz, 1 H, *H*12′), 1.22 (t, *J* = 7.0 Hz, 3 H, *H*1), 0.77 (t, *J* = 7.5 Hz, 3 H, *H*14); **^{13}C NMR (126 MHz, CHCl$_3$)** δ_C 214.8, 171.5, 61.1, 53.3, 52.1, 41.8, 39.9, 30.6, 30.2, 29.3, 22.9, 20.1, 14.3, 11.9; **HRMS (ESI$^+$)** *m/z* calculated for $[M+Na]^+$ $C_{14}H_{20}NaO_3$ 259.1305, found 259.1314; **IR ν_{max}/cm^{-1} (thin film)** 2963, 1720, 1455, 1377, 1281.

Preparation of (1a*R,1b*R**,4a*S**,5a*S**)-ethyl 5a-ethyl-5-oxooctahydro-1*H*-cyclopropa[*a*]-pentalene-1a-carboxylate, 14**

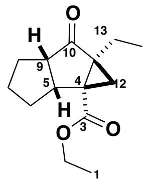

Following **General Procedure B**, using cyclization precursor **(*E*)-5c** (*E* isomer) (50 mg, 0.16 mmol), THF (4 mL), NaH (6 mg, 0.16 mmol) afforded compound **14** (31 mg, 82%) as a colorless oil.

N.B. Stereochemistry assigned by nOe analysis similar to compound **12**.

^{1}H NMR (500 MHz, CDCl$_3$) δ_H 4.13 (qd, *J* = 7.0, 1.5 Hz, 2 H, *H*2), 2.75 (dd, *J* = 8.5, 8.0 Hz, 1 H, *H*5), 2.47-2.43 (m, 1 H, *H*9), 2.25-2.19 (m, 1 H, *H*6), 2.17 (q, *J* = 7.0 Hz, 1 H, *H*13), 1.77-1.73 (m, 2 H, *H*8), 1.64 (dd, *J* = 4.5, 1.5 Hz, 1 H, *H*12), 1.56 (q, *J* = 7.0 Hz, 1 H, *H*13′), 1.55-1.45 (m, 2 H, *H*7), 1.31 (d, *J* = 4.5 Hz, 1 H, *H*12′), 1.22 (t, *J* = 7.0 Hz, 3 H, *H*1), 1.20-1.15 (m, 1 H, *H*6′), 0.77 (t, *J* = 7.0 Hz, 3 H, *H*14); **^{13}C NMR (126 MHz, CDCl$_3$)** δ_C 214.9,

171.5, 60.8, 48.2, 46.7, 43.5, 40.1, 32.6, 28.5, 26.7, 26.5, 18.8, 14.2, 11.9; **HRMS (ESI$^+$)** *m/z* calculated for $[M+Na]^+$ $C_{14}H_{20}NaO_3$ 259.1305, found 259.1313; **IR ν_{max}/cm^{-1} (thin film)** 2962, 1721, 1449, 1374, 1286.

Preparation of (1a*S,1b*R**,4a*S**,5a*S**)-ethyl 5-oxo-5a-phenyloctahydro-1*H*-cyclopropa[*a*]-pentalene-1a-carboxylate, 22**

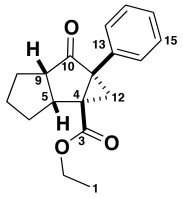

Following **Geneal Procedure A**, using cyclization precursor, **(*Z*)-5d** (50 mg, 0.14 mmol), toluene (4 mL), KOH (31 mg, 0.55 mmol), tetrabutylammonium bisulfate (5 mg, 0.02 mmol) at RT afforded compound **22** (32 mg, 80%) as a colorless oil.

Following **General Procedure B**, using cyclization precursor **(*Z*)-5d** (50 mg, 0.11 mmol), THF (4 mL), NaH (5 mg, 0.66 mmol) afforded compound **22** (26 mg, 65%) as a colorless oil. N.B. Stereochemistry assigned by nOe analysis similar to compound **19**.

^{1}H NMR (500 MHz, toluene-*d*$_8$) δ_H 7.15-7.03 (m, 5 H, Ar*H*), 3.81-3.70 (m, 2 H, *H*5, *H*2), 3.63-3.57 (m, 1 H, *H*2′), 2.76-2.70 (m, 1 H, *H*9), 2.24 (dd, *J* = 5.5, 1.5 Hz, 1 H, *H*12), 1.84-1.76 (m, 1 H, *H*6), 1.68-1.61 (m, 1 H, *H*8), 1.48-1.29 (m, 4 H, *H*6′, *H*8′, *H*7), 1.26 (d, *J* = 5.5 Hz, 1 H, *H*12′), 0.56 (t, *J* = 7.0 Hz, 3 H, *H*1); **^{13}C NMR (126 MHz, toluene-*d*$_8$)** δ_C 210.3, 169.2, 137.1, 134.5, 128.3, 128.3, 60.1, 54.2, 53.5, 44.2, 39.7, 30.5, 30.0, 29.0, 19.8, 13.3; **HRMS (ESI$^+$)** *m/z* calculated for $[M+Na]^+$ $C_{18}H_{20}NaO_3$ 307.1305, found 307.1311; **IR ν_{max}/cm^{-1} (thin film)** 2950, 2870, 1703, 1450, 1275, 1255, 1221, 1130.

Preparation of (1a*R,1b*R**,4a*S**,5a*R**)-ethyl 5-oxo-5a-phenyloctahydro-1*H*-cyclopropa[*a*]-pentalene-1a-carboxylate, 15**

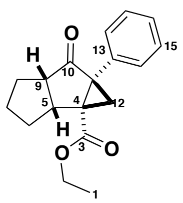

Following **General Procedure A**, using cyclization precursor **(*E*)-5d** (50 mg, 0.14 mmol), toluene (4 mL), KOH (31 mg, 0.55 mmol), tetrabutylammonium bisulfate (5 mg, 0.02 mmol) at RT afforded an inseparable 1:1 mixture of compound **15** and compound **22** (29 mg, 73%) as a colorless oil.

Following **General Procedure B**, using cyclization precursor **(*E*)-5d** (50 mg, 0.11 mmol), THF (4 mL), NaH (5 mg, 0.66 mmol) afforded compound **15** (33 mg, 83%) as a colorless oil. N.B. Stereochemistry assigned by nOe analysis similar to compound **12**.

^{1}H NMR (500 MHz, CDCl$_3$) δ_H 7.26-7.16 (m, 3 H, Ar*H*), 7.08-7.04 (m, 2 H, Ar*H*), 3.85-3.75 (m, 2 H, *H*2), 2.89 (dd, *J* = 17.5, 7.5 Hz, 1 H, *H*5), 2.68 (dd, *J* = 13.5, 7.5 Hz, 1 H, *H*9), 2.42-2.37 (m, 1 H, *H*6), 2.36 (dd, *J* = 5.0, 1.5 Hz, 1 H, *H*12), 1.80 (dt, *J* = 8.5, 6.5 Hz, 2 H, *H*8), 1.77-1.71 (m, 1 H, *H*7), 1.70 (d, *J* = 5.0 Hz, 1 H, *H*12′), 1.57-1.48 (m, 1 H, *H*7′), 1.48-1.38 (m, 1 H, *H*6′), 0.75 (m, *J* = 7.5 Hz, 3 H, *H*1); **^{13}C NMR (126 MHz, CDCl$_3$)** δ_C 213.5, 169.9, 133.4, 130.4, 128.3, 127.6, 60.5, 49.9, 48.9, 43.7, 43.1, 32.8, 26.8, 26.7, 25.7, 13.6; **HRMS (ESI$^+$)** *m/z* calculated for [M+Na]$^+$ $C_{18}H_{20}NaO_3$ 307.1305, found 307.1313; **IR ν_{max}/cm^{-1} (thin film)** 2980, 2875, 1723, 1484, 1376, 1248, 1128.

Preparation of (1*S,1a*R**,1b*R**,4a*S**,5a*R**)-ethyl 5-oxo-1-phenyloctahydro-1*H*-cyclopropa[*a*]pentalene-1a-carboxylate, 24**

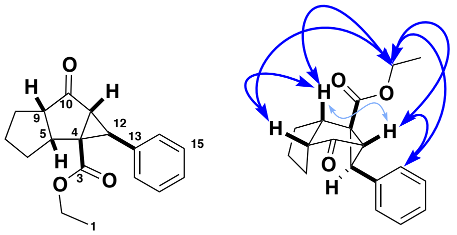

Following **General Procedure A**, using cyclization precursor **(*E*)-5e** (50 mg, 0.13 mmol), toluene (3 mL), KOH (30 mg, 0.53 mmol), tetrabutylammonium bisulfate (4 mg, 0.01 mmol) at RT afforded compound **24** (34 mg, 88%) as a colorless oil.

Following **General Procedure A**, using cyclization precursor **(*E*)-5e** (50 mg, 0.13 mmol), toluene (3 mL), KOH (30 mg, 0.53 mmol), tetrabutylammonium bisulfate (4 mg, 0.01 mmol) at RT afforded compound **24** (29 mg, 75%) as a colorless oil.

Following **General Procedure B**, using cyclization precursor **(*Z*)-5d** (50 mg, 0.13 mmol), THF (3 mL), NaH (10 mg, 0.26 mmol) afforded compound **24** (32 mg, 83%) as a colorless oil.

N.B.: Key nOe interactions are highlighted. Bold dark blue arrows indicate a strong correlation and light blue arrows indicate a weak correlation.

^{1}H NMR (500 MHz, CDCl$_3$) δ_H 7.32-7.28 (m, 2 H, Ar*H*), 7.26-7.20 (m, 3 H, Ar*H*), 3.88-3.79 (m, 2 H, *H*2), 3.52-3.45 (m, 1 H, *H*5), 3.06 (dd, *J* = 3.5, 1.5 Hz, 1 H, *H*11), 2.99-2.93 (m, *2* H, *H*12, *H*9), 2.34-2.28 (m, 1 H, *H*6), 2.01-1.88 (m, 3 H, *H*6', *H*7 *H8*), 1.78-1.70 (m, 1 H, *H*7'), 1.65-1.55 (m, 1 H, *H*8'), 0.83 (t, *J* = 7.0 Hz, 3 H, *H*1); **^{13}C NMR (126 MHz, CDCl$_3$)** δ_C 212.7, 170.1, 134.0, 128.8, 128.3, 127.3, 60.7, 54.4, 44.5, 42.8, 42.1, 34.3, 31.8, 30.4, 29.7, 13.7; **HRMS (ESI$^+$)** *m/z* calculated for $[M+Na]^+$ $C_{18}H_{20}NaO_3$ 307.1310, found 307.1303; **IR ν_{max}/cm^{-1} (thin film)** 2950, 2870, 1703, 1450, 1275, 1255, 1221, 1130.

Preparation of (1*R,1a*S**,1b*R**,4a*S**,5a*S**)-ethyl 5-oxo-1-phenyloctahydro-1*H*-cyclopropa[*a*]pentalene-1a-carboxylate, 17**

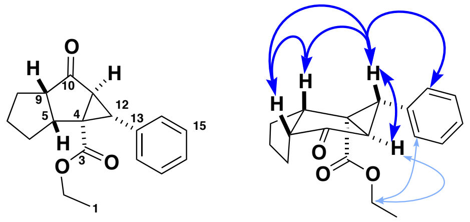

Following **General Procedure B**, using cyclization precursor **(*E*)-5e** (50 mg, 0.13 mmol), THF (3 mL), NaH (10 mg, 0.26 mmol) afforded compound **17** (29 mg, 75%) as a colorless oil.

N.B.: Key nOe interactions are highlighted. Bold dark blue arrows indicate a strong correlation and light blue arrows indicate a weak correlation.

^{1}H NMR (500 MHz, CDCl$_3$) δ_H 7.32-7.27 (m, 2 H, Ar*H*), 7.26-7.22 (m, 3 H, Ar*H*), 3.93-3.82 (m, 2 H, *H*2), 3.23 (q, *J* = 8.0 Hz, 1 H, *H*5), 2.97 (d, *J* = 4.5 Hz, 1 H, *H*11), 2.87 (d, *J* = 4.5 Hz, 1 H, *H*12), 2.77-2.72 (m, 1 H, *H*9), 2.14-2.08 (m, 1 H, *H*6), 1.95-1.89 (m, 2 H, *H*8), 1.70-1.53 (m, 2 H, *H*7), 1.41-1.30 (m, 1 H, *H*6'), 0.89 (t, *J* = 7.0 Hz, 3 H, *H*1); **^{13}C NMR (126 MHz, CDCl$_3$)** δ_C 213.7, 168.6, 134.1, 128.5, 128.3, 127.4, 60.7, 49.9, 45.2, 44.8, 38.4, 37.4, 31.5, 28.2, 26.5, 13.8; **HRMS (ESI$^+$)** *m/z* calculated for $[M+Na]^+$ $C_{18}H_{20}NaO_3$ 307.1310, found 307.1302; **IR ν_{max}/cm^{-1} (thin film)** 2980, 2875, 1723, 1484, 1376, 1248, 1128.

Preparation of (1a*R,1b*R**,4a*S**,5a*R**)-ethyl 1-(3-fluorophenyl)-5-oxooctahydro-1*H*-cyclopropa[*a*]pentalene-1a-carboxylate, 23**

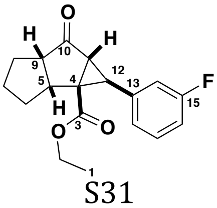

Following **General Procedure A**, using cyclization precursor **(*E*)-5f** (50 mg, 0.13 mmol), toluene (4 mL), KOH (29 mg, 0.52 mmol), tetrabutylammonium bisulfate (4 mg, 0.01 mmol) at RT afforded compound **23** (25 mg, 64%) as a colorless oil.

Following **General Procedure A**, using cyclization precursor **(*Z*)-5f** (50 mg, 0.13 mmol), toluene (4 mL), KOH (29 mg, 0.52 mmol), tetrabutylammonium bisulfate (4 mg, 0.01 mmol) at RT afforded compound **23** (22 mg, 56%) as a colorless oil.

Following **General Procedure B**, using cyclization precursor **(*Z*)-5f** (50 mg, 0.13 mmol), THF (4 mL), NaH (6 mg, 0.26 mmol) afforded compound **23** (26 mg, 66%) as a colorless oil.

N.B. Stereochemistry assigned by nOe analysis similar to compound **19**.

^{1}H NMR (500 MHz, $CDCl_3$) δ_H 7.19-7.15 (m, 1 H, Ar*H*), 6.93 (d, *J* = 8.0 Hz, 1 H, Ar*H*), 6.88-6.85 (m, 2 H, Ar*H*), 3.79 (q, *J* = 7.0 Hz, 2 H, *H*2), 3.44-3.39 (m, 1 H, *H*5), 2.94 (dd, *J* = 5.0, 1.0 Hz, 1 H, *H*11), 2.87 (q, *J* = 10.5 Hz, 1 H, *H*9), 2.82 (d, *J* = 5.0 Hz, 1 H, *H*12), 2.25-2.19 (m, 1 H, *H*6), 1.92-1.85 (m, 1 H, *H*8), 1.85-1.78 (m, 2 H, *H*6', *H*7), 1.68-1.60 (m, 1 H, *H*7'), 1.54-1.45 (m, 1 H, *H*8') 0.81 (t, *J* = 7.0 Hz, 3 H, *H*1); **^{13}C NMR (126 MHz, $CDCl_3$)** δ_C 212.0, 169.1, 162.6 (d, *J* = 246.0 Hz), 136.6 (d, *J* = 7.5 Hz), 129.8 (d, *J* = 8.5 Hz,), 124.5 (d, *J* = 2.5 Hz), 115.9 (d, *J* = 22.0 Hz), 114.3 (d, *J* = 21.0 Hz), 60.8, 54.4, 44.5, 42.8, 42.2, 33.7, 31.7, 30.4, 29.7, 13.7; **^{19}F NMR (377 MHz, $CDCl_3$)** δ_F -113.2; **HRMS (ESI$^+$)** *m/z* calculated for $[M+Na]^+$ $C_{18}H_{19}NaFO_3$ 325.1210, found 325.1207; **IR ν_{max}/cm^{-1} (thin film)** 2959, 1722, 1613, 1587, 1446, 1373, 1211, 1149.

Preparation of (1*S,1a*S**,1b*R**,4a*S**,5a*S**)-ethyl 1-(3-fluorophenyl)-5-oxooctahydro-1*H*-cyclopropa[*a*]pentalene-1a-carboxylate, 16**

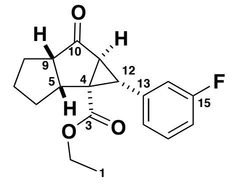

Following **General Procedure B**, using cyclization precursor **(*E*)-5f** (50 mg, 0.13 mmol), THF (4 mL), NaH (6 mg, 0.26 mmol) afforded compound **16** (35 mg, 88%) as a colorless oil.

N.B. Stereochemistry assigned by nOe analysis similar to compound **12**.

^{1}H NMR (500 MHz, $CDCl_3$) δ_H 7.19-7.14 (m, 1 H, Ar*H*), 6.94 (d, *J* = 8.0 Hz, 1 H, Ar*H*), 6.88-6.84 (m, 2 H, Ar*H*), 3.84 (qd, *J* = 7.0, 2.0 Hz, 2 H, *H*2), 3.12 (q, *J* = 8.0 Hz, 1 H, *H*5), 2.84 (d, *J* = 4.5 Hz, 1 H, *H*11), 2.74 (d, *J* = 4.5 Hz, 1 H, *H*12), 2.65 (q, *J* = 7.5 Hz, 1 H, *H*9), 2.05-1.99 (m, 1 H, *H*6), 1.83 (q, *J* = 6.5 Hz, 2 H, *H*8), 1.61-1.45 (m, 2 H, *H*7), 1.30-1.25 (m, 1 H, *H*6'), 0.86 (t, *J* = 7.0 Hz, 3 H, *H*1); **^{13}C NMR (126 MHz, $CDCl_3$)** δ_C 213.3, 168.3, 162.6 (d, *J* = 246.0 Hz), 136.6 (d, *J* = 7.5 Hz), 129.9 (d, *J* = 8.5 Hz), 124.2, 115.5 (d, *J* = 22.0 Hz), 114.4 (d, *J* = 21.0 Hz), 60.9, 49.8, 45.2, 44.8, 38.4, 36.9, 31.5, 28.2, 26.5, 14.1; **^{19}F NMR (377 MHz, $CDCl_3$)** δ_F −113.2; **HRMS (ESI$^+$)** *m/z* calculated for $[M+H]^+$ $C_{18}H_{20}FO_3$ 303.1397, found 303.1390; **IR ν_{max}/cm^{-1} (thin film)** 2980, 2890, 1745, 1625, 1595, 1452, 1238, 1166.

Preparation of (1*R1a*R**,1b*R**4a*S**,5a*R**)-ethyl 5-oxo-1-(*p*-tolyl)octahydro-1*H*-cyclopropa[*a*]pentalene-1a-carboxylate, 25**

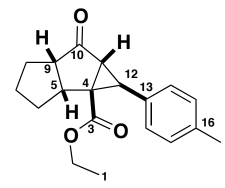

Following **General Procedure A**, using cyclization precursor **(*E*)-5g** (50 mg, 0.13 mmol), toluene (3 mL), KOH (30 mg, 0.53 mmol), tetrabutylammonium bisulfate (4 mg, 0.01 mmol) at RT afforded compound **25** (34 mg, 88%) as a colorless oil.

Following **General Procedure A**, using cyclization precursor **(*Z*)-5g** (50 mg, 0.13 mmol), toluene (3 mL), KOH (30 mg, 0.53 mmol), tetrabutylammonium bisulfate (4 mg, 0.01 mmol) at RT afforded compound **25** (23 mg, 59%) as a colorless oil.

Following **General Procedure B**, using cyclization precursor **(*Z*)-5g** (50 mg, 0.13 mmol), THF (3 mL), NaH (10 mg, 0.26 mmol) afforded compound **25** (26 mg, 74%) as a colorless oil.

N.B. Stereochemistry assigned by nOe analysis similar to compound **19**.

^{1}H NMR (500 MHz, $CDCl_3$) δ_H 7.12-7.08 (m, 4 H, Ar*H*), 3.86 (qd, *J* = 7.0, 1.0 Hz, 2 H, *H*2), 3.50-3.45 (m, 1 H, *H*5), 3.03 (dd, *J* = 5.0, 1.5 Hz, 1 H, *H*11), 2.98-2.93 (m, 1 H, *H*9), 2.91 (d, *J* = 5.0 Hz, 1 H, *H*12), 2.33 (s, 3 H, *H*17), 2.31-2.25 (m, 1 H, *H*6), 1.98-1.89 (m, 3 H, *H*8, *H*7, *H*6'), 1.76-1.68 (m, 1 H, *H*7'), 1.60-1.55 (m, 1 H, *H*8') 0.88 (t, *J* = 7.0 Hz, 3 H, *H*1); **^{13}C NMR (126 MHz, $CDCl_3$)** δ_C 213.1, 170.2, 136.9, 130.8, 128.9, 128.6, 60.7, 54.4, 44.5, 42.8, 42.3, 34.1, 31.7, 30.4, 29.7, 21.1, 13.7; **HRMS (ESI$^+$)** *m/z* calculated for $[M+Na]^+$

$C_{19}H_{22}NaO_3$ 321.1467, found 321.1458; **IR ν_{max}/cm^{-1} (thin film)** 2983, 1742, 1371, 1248, 1180.

Preparation of (1*R,1a*S**,1b*R**,4a*S**,5a*S**)-ethyl 5-oxo-1-(*p*-tolyl)octahydro-1*H*-cyclopropa[*a*]pentalene-1a-carboxylate, 18**

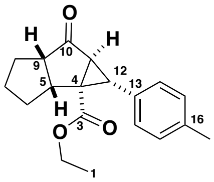

Following **General Procedure B**, using cyclization precursor **(*E*)-5g** (50 mg, 0.13 mmol), THF (3 mL), NaH (10 mg, 0.26 mmol) afforded compound **18** (29 mg, 75%) as a colorless oil.

N.B. Stereochemistry assigned by nOe analysis similar to compound **12**.

^{1}H NMR (500 MHz, CDCl$_3$) δ_H 7.03-6.99 (m, 4 H, Ar*H*), 3.81 (qd, *J* = 7.0, 2.0 Hz, 2 H, *H*2), 3.12 (q, 1 H, *J* = 8.0 Hz, *H*5), 2.84 (d, *J* = 4.5 Hz, 1 H, *H*11), 2.74 (d, 1 H, *J* = 4. Hz, *H*12), 2.65 (dt, *J* = 8.0, 4.5 Hz, 1 H, *H*9), 2.23 (s, 3 H, *H*17), 2.64-1.98 (m, 1 H, *H*6), 1.85-1.80 (m, 2 H, *H*8) 1.60-1.44 (m, 2 H, *H*7), 1.31-1.24 (m, 1 H, *H*6’), 0.85 (t, *J* = 7.0 Hz, 3 H, *H*1); **^{13}C NMR (126 MHz, CDCl$_3$)** δ_C 213.1, 168.7, 137.0, 131.0, 129.0, 128.3, 60.7, 49.9, 45.2, 44.9, 38.5, 37.3, 31.6, 28.2, 26.5, 21.1, 13.9; **HRMS (ESI$^+$)** *m/z* calculated for [M+Na]$^+$ $C_{19}H_{22}O_3$ 299.1642, found 299.1637; **IR ν_{max}/cm^{-1} (thin film)** 2956, 2945, 1742, 1520, 1320, 1240, 1160.

1.3.3 Cyclization Product Derivatizations:

Preparation of (1a*S, 1b*R**, 4a*S**, 5a*R**)-ethyl 5-oxooctahydro-1*H*-cyclopropa[*a*]pentalene-1*a*-carboxylate-4a-*d*, 26**

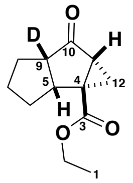

Sodium (6 mg, 0.25 mmol) was dissolved in EtOD (3 mL) and the reaction mixture stirred until the sodium was fully dissolved. Ketone **19** (50 mg, 0.25 mmol) was dissolved in EtOD (2 mL) and added dropwise into the solution of sodium ethoxide in ethanol. The resulting solution was stirred for 24 hrs, quenched with D_2O and extracted with Et_2O (× 3). The

combined organics were dried over $MgSO_4$, filtered and the filtrate concentrated under reduced pressure to afford the crude product. This was purified by flash column chromatography (SiO_2, pet 40-60:Et_2O (9:1)) to afford deuterated compound **26** (48 mg, 92%).

Stereochemical assignment given by comparison with non-deuterated product **19**.

^{1}H NMR (500 MHz, CDCl$_3$) δ_H 4.09 (q, J = 7.0 Hz, 2 H, *H*2), 3.51-3.46 (m, 1 H, *H*5), 2.36 (dd, J = 10.0, 4.5 Hz, 1 H, *H*11), 2.02-1.94 (m, 1 H, *H*6), 1.83-1.67 (m, 3 H, *H*7, *H*8, *H*12), 1.59-1.51 (m, 2 H, *H*6', *H*7'), 1.43-1.37 (m, 1 H, *H*8'), 1.32 (t, J = 5.0 Hz, 1 H, *H*12'), 1.19 (t, J = 7.0 Hz, 3 H, *H*1); **^{13}C NMR (126 MHz, CDCl$_3$)** δ_C 213.2, 172.1, 61.1, {53.8, 53.6, 53.4, 1:1:1 t, $J_{C\text{–}D}$ = 20.5 Hz}, 40.2, 40.1, 37.7, 30.9, 30.1, 29.5, 22.7, 14.2; **HRMS (ESI$^+$)** *m/z* calculated for $[M+H]^+$ $C_{12}H_{16}{}^2HO_3$ 210.1235, found 210.1236; **IR ν_{max}/cm^{-1} (thin film)** 2957, 1720, 1451, 1380, 1247, 1202, 1132.

Preparation of (1a*S,1b*R**,4a*S**,5*S**,5a*R**)-ethyl 5-hydroxy-5-vinyloctahydro-1*H*-cyclopropa[*a*]pentalene-1a-carboxylate, 27**

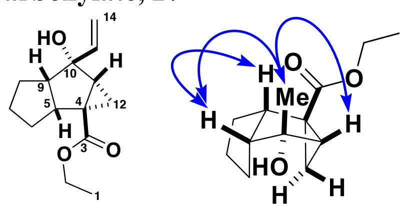

Ketone **19** (50 mg, 0.25 mmol) was dissolved in THF (5 mL) and cooled to 0 °C. Vinylmagnesium bromide (1.0 M in THF, 0.25 mL) was added, the reaction stirred for 1 hr and then quenched with NH_4Cl (sat. aq.). The reaction mixture was allowed to warm to RT and then extracted with EtOAc (× 3). The combined organics were dried over $MgSO_4$, filtered and the filtrate concentrated under reduced pressure to afford the crude product. This was purified by flash column chromatography (SiO_2, pet 40-60:Et_2O (8:2)) to afford compound **27** (46 mg, 78%) as a single diastereomer.

N.B. Stereochemistry assigned by nOe analysis, with blue arrows indicating through-space interaction.

^{1}H NMR (500 MHz, CDCl$_3$) δ_H 6.07 (dd, J = 17.5, 10.5 Hz, 1 H, *H*13), 5.16 (dd, J = 17.5, 1.0 Hz, 1 H, *H*14), 4.96 (dd, J = 10.5, 1.0 Hz, 1 H, *H*14′), 4.04 (q, J = 7.0 Hz, 2 H, *H*2), 3.24-3.19 (m, 1 H, *H*5), 2.41 (q, J = 9.0 Hz, 1 H, *H*9), 2.05 (dd, J = 9.0, 5.0 Hz, 1 H, *H*11), 2.00-1.95 (m, 1 H, *H*6), 1.85-1.80 (m, 1 H, *H*7), 1.62 (br. s, 1 H, O*H*), 1.53-1.46 (m, 2 H, *H*6′, *H*8), 1.45-1.39 (m, 2 H, *H*8′, *H*7′) 1.26 (ddd, J = 9.0, 5.0, 1.5 Hz, 1 H, *H*12), 1.21 (t, J = 5.0 Hz,

1 H, *H*12')1.16 (t, *J* = 7.0 Hz, 3 H, *H*1); **^{13}C NMR (126 MHz, $CDCl_3$)** δ_C 173.7, 145.9, 109.8, 80.0, 60.5, 54.7, 43.7, 42.0, 37.0, 30.9, 28.7, 28.6, 17.4, 14.2; **HRMS (ESI$^+$)** *m/z* calculated for $[M+Na]^+$ $C_{14}H_{20}NaO_3$ 259.1305, found 259.1312; **IR ν_{max}/cm^{-1} (thin film)** 3483, 2957, 1719, 1449, 1382, 1285.

Preparation of (1a*S,1b*R**,4a*S**,5*S**,5a*R**)-ethyl 5-hydroxyoctahydro-1*H*-cyclopropa[*a*]pentalene-1a-carboxylate, 28**

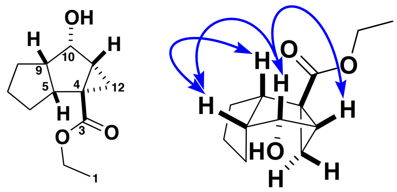

Ketone **19** (50 mg, 0.25 mmol) was dissolved in THF (5 mL) and cooled to –78 °C. L-Selectride (1.0 M in THF, 0.25 mL) was added, the reaction stirred for 5 min at –78 °C and then quenched with H_2O. The reaction mixture was allowed to warm to RT and then extracted with Et_2O (× 3). The combined organics were dried over $MgSO_4$, filtered and the filtrate concentrated under reduced pressure to afford the crude product. This was purified by flash column chromatography (SiO_2, pet 40-60:Et_2O (6:4)) to afford compound **28** (50 mg, 95%) as a single diastereomer.
N.B. Stereochemistry assigned by nOe analysis, with blue arrows indicating through-space interaction).

^{1}H NMR (500 MHz, $CDCl_3$) δ_H 4.65 (dd, *J* = 9.5, 5.5 Hz, 1 H, *H*10), 4.03 (q, *J* = 7.0 Hz, 2 H, *H*2), 3.24-3.17 (m, 1 H, *H*5), 2.69-2.62 (m, 1 H, *H*9), 2.21 (dt, *J* = 9.0, 5.5 Hz, 1 H, *H*11), 1.98-1.93 (m, 1 H, *H*6), 1.83-1.78 (m, 1 H, *H*8), 1.50-1.35 (m, 5 H, *H*6′, *H*8′, *H*7, O*H*), 1.25-1.22 (m, 1 H, *H*12), 1.16 (t, *J* = 7.0 Hz, 3 H, *H*1), 1.12 (t, *J* = 5.5 Hz, 1 H, *H*12′); **^{13}C NMR (126 MHz, $CDCl_3$)** δ_C 174.9, 72.5, 60.5, 48.8, 44.1, 38.8, 36.5, 30.8, 28.9, 27.7, 16.8, 14.3; **HRMS (ESI$^+$)** *m/z* calculated for $[M+Na]^+$ $C_{12}H_{18}NaO_3$ 233.1148, found 233.1155; **IR ν_{max}/cm^{-1} (thin film)** 3434, 2955, 1718, 1449, 1382, 1286.

Preparation of (1a*S,1b*R**,4a*S**,5*S**,5a*R**)-ethyl 5-(benzylamino)octahydro-1*H*-cyclopropa[*a*]pentalene-1a-carboxylate, 29**

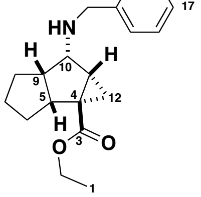

Ketone **19** (130 mg, 0.63 mmol) was dissolved in THF (10 mL) and benzylamine (60 μL, 0.50 mmol) and AcOH (26 μL, 0.50 mmol) were added. The reaction mixture was stirred overnight, then $NaBH_4$ (10 mg, 0.25 mmol) was added. The reaction was quenched with NH_4Cl (sat. aq.) and extracted with EtOAc (× 3). The combined organics were dried over $MgSO_4$, filtered and the filtrate concentrated under reduced pressure to afford the crude product. This was purified by flash column chromatography (SiO_2, pet 40-60:Et_2O (9:1)) to afford compound **29** (54 mg, 75% based on benzylamine as limiting reagent) as a single diastereomer.

N.B. Stereochemistry assigned by nOe analysis similar to compound **28**.

^{1}H NMR (500 MHz, $CDCl_3$) δ_H 7.40-7.33 (m, 3 H, Ar*H*), 7.30-7.26 (m, 2 H, Ar*H*), 4.12 (q, *J* = 7.0 Hz, 2 H, *H*2), 3.85 (q, *J* = 12.5 Hz, 2 H, *H*13), 3.71 (dd, *J* = 9.0, 5.5 Hz, 1 H, *H*10), 3.33-3.25 (m, 1 H, *H*5), 2.77-2.66 (m, 1 H, *H*9) 2.21 (dt, *J* = 9.0, 5.0 Hz, 1 H, *H*11), 2.10-2.03 (m, 1 H, *H*6), 1.67-1.60 (m, 1 H, *H*7) 1.56-1.45(m, 1 H, *H*8), 1.46-1.31 (m, 3 H, *H*6′, *H*7′, *H*8′), 1.31-1.28 (m, 1 H, *H*12), 1.25 (t, *J* = 7.0 Hz, 3 H, *H*1), 1.07 (t, *J* = 5.0 Hz, 1 H, *H*12′); **^{13}C NMR (126 MHz, $CDCl_3$)** δ_C 154.4, 140.4, 128.5, 128.2, 127.0, 60.4, 58.8, 53.0, 47.5, 44.0, 37.6, 36.6, 30.6, 28.6, 28.2, 17.2, 14.3; **HRMS (ESI$^+$)** *m/z* calculated for $[M+H]^+$ $C_{19}H_{26}NO_2$ 300.1958, found 30.1963; **IR ν_{max}/cm^{-1} (thin film)** 2951, 2316, 2314, 1703, 1496, 1454.

Preparation of (3a*R,4*R**,7a*S**)-ethyl 7-oxooctahydro-1*H*-indene-4-carboxylate, 30**

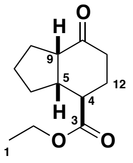

N.B. Teflon-coated magnetic stirring bars must not used in this reaction, due to their ability to quench the sodium naphthalenide reagent. Glass stirring bars, or no stirring bar, must be employed.

Preparation of sodium naphthalenide:
Naphthalene (256 mg, 2.0 mmol) was dissolved in THF (10 mL) and freshly cut Na (92 mg, 4.0 mmol) was added (N.B. no magnetic stirring bar). The colorless solution was sonicated under Ar for 30 min, with intermittent swirling, after which time the solution had turned deep green as the Na was consumed. This was used immediately, and assumed to be a 0.2 M solution of sodium naphthalenide.

*Preparation of **30**:*

Ketone **19** (50 mg, 0.2 mmol) was dissolved in THF (6 mL) and cooled to –78 °C. Sodium naphthalenide (~ 4 eq.) was added dropwise, with intermittent swirling, until the green color persisted in the reaction mixture. After this time, MeOH (1 drop) was added and the reaction mixture was slowly warmed to RT. H_2O was added and extracted with Et_2O (× 3). The combined organics were dried over $MgSO_4$, filtered and the filtrate concentrated under reduced pressure to afford the crude product. This was purified by flash column chromatography (SiO_2, pet 40-60:Et_2O (8:2)) to afford compound **19** (32 mg, 75%) as an inseparable 4:1 mixture of isomers. The relative stereochemistry of the minor isomer could not be ascertained.

N.B. Stereochemistry assigned by nOe analysis similar to compound **19**

For clarity, the minor isomer was omitted from the NMR data assignments:

^{1}H NMR (500 MHz, CDCl$_3$) δ_H 4.23-4.15 (m, 2 H, *H*2), 3.13 (dt, *J* = 12.5, 4.5 Hz, 1 H, *H*4), 2.86-2.79 (m, 1 H, *H*5), 2.76-2.69 (m, 1 H, *H*9), 2.49 (ddd, *J* = 14.5, 4.0, 3.0 Hz, 1 H, *H*11), 2.41-2.32 (m, 2 H, *H*11′, *H*8), 2.18-2.12 (m, 1 H, *H*12), 2.11-2.01 (m, 1 H, *H*12′), 1.71-1.65 (m, 1 H, *H*7), 1.58-1.52 (m, 3 H, *H*8′, *H*7′, *H*6), 1.32-1.26 (m, 1 H, *H*6′), 1.29 (t, *J* = 7.0 Hz, 3 H, *H*1); **^{13}C NMR (126 MHz, CDCl$_3$)** δ_C 211.1, 173.6, 60.7, 52.4, 44.5, 42.4, 40.1, 26.9, 24.9, 24.4, 23.5, 22.2; **HRMS (ESI$^+$)** *m/z* calculated for $[M+Na]^+$ $C_{12}H_{18}NaO_3$ 233.1148, found 233.1145; **IR ν_{max}/cm^{-1} (thin film)** 2953, 1710, 1594, 1433, 1375, 1314.

Preparation of (1a*S,1b*R**,4a*S**,5a*R**)-5-oxooctahydro-1*H*-cyclopropa[*a*]pentalene-1a-carboxylic acid, S25**

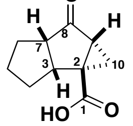

Ketone **19** (50 mg, 0.25 mmol) was dissolved in THF:H_2O (6 mL:2 mL) and LiOH (5 mg, 0.25 mmol) was added. The reaction stirred for 4 hr, quenched with NH_4Cl (sat. aq.) and extracted with EtOAc (× 3). The combined organics were dried over $MgSO_4$, filtered and the filtrate concentrated under reduced pressure to afford the crude product. This was purified by flash column chromatography (SiO_2, pet 40-60:Et_2O (7:3)) to afford compound **S25** (41 mg, 91%) as a single diastereomer.

N.B. Stereochemistry assigned by nOe analysis similarities to compound **19**.

^{1}H NMR (500 MHz, $CDCl_3$) δ_H 3.50-3.45 (m, 1 H, *H*3), 2.82 (q, J = 10.0 Hz, 1 H, *H*7), 2.45 (ddd, J = 10.5, 5.0, 1.5 Hz, 1 H, *H*9), 2.04-1.96 (m, 1 H, *H*4), 1.85-1.77 (m, 2 H, *H*10, *H*6), 1.73-1.67 (m, 1 H, *H*5), 1.60-1.50 (m, 2 H, *H*4′, *H*5′), 1.45-1.42 (m, 1 H, *H*6′), 1.40 (t, J = 5.0 Hz, 1 H, *H*10′); [COO*H* not observed]; **^{13}C NMR (126 MHz, $CDCl_3$)** δ_C 212.6, 179.0, 54.1, 40.8, 39.9, 37.2, 30.9, 30.2, 29.7, 19.1; **HRMS (ESI$^+$)** *m/z* calculated for $[M+Na]^+$ $C_{10}H_{12}NaO_3$ 203.0679, found 203.0682; **IR ν_{max}/cm^{-1} (thin film)** 3232, 2958, 1728, 1448, 1228, 1156.

Preparation of (1a*R, 1b*R**, 4a*S**, 5a*S**)-ethyl 5-oxooctahydro-1*H*-cyclopropa[*a*]pentalene-1*a*-carboxylate-4a-*d*, S26**

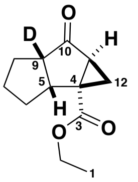

Sodium (6 mg, 0.25 mmol) was dissolved in EtOD (3 mL) and the reaction mixture stirred until the sodium was fully dissolved. Ketone **12** (50 mg, 0.25 mmol) was dissolved in EtOD (2 mL) and added dropwise into the solution of sodium ethoxide in ethanol. The resulting solution was stirred for 24 hrs, quenched with D_2O and extracted with Et_2O (× 3). The combined organics were dried over $MgSO_4$, filtered and the filtrate concentrated under reduced pressure to afford the crude product. This was purified by flash column chromatography (SiO_2, pet 40-60:Et_2O (9:1)) to afford compound **S26** (50 mg, 96%).
Stereochemical assignment given by comparison with non-deuterated product **12**.

^{1}H NMR (500 MHz, $CDCl_3$) δ_H 4.17-4.02 (m, 2 H, *H*2), 2.83 (t, J = 8.5 Hz, 1 H, *H*5), 2.36 (dd, J = 9.5, 4.0 Hz, 1 H, *H*11), 2.18 (dtd, J = 13.0, 7.0, 4.0 Hz, 1 H, *H*6), 1.75 (dd, J = 8.5, 6.0 Hz, 2 H, *H*8), 1.69 (dd, J = 9.5, 4.5 Hz, 1 H, *H*12), 1.65-1.56 (m, 1 H, *H*7), 1.48-1.40 (m, 1 H, *H*7′), 1.31 (t, J = 4.5 Hz, 1 H, *H*12′), 1.20-1.16 (m, 1 H, *H*6′), 1.19 (t, J = 7.0 Hz, 3 H, *H*1); **^{13}C NMR (126 MHz, $CDCl_3$)** δ_C 213.7, 171.4, 60.9, {48.8, 48.6, 48.4, 1:1:1 t, $J_{C\text{–}D}$ = 20.5 Hz}, 43.5, 36.7, 34.8, 31.9, 26.7, 26.6, 22.7, 14.2; **HRMS (ESI$^+$)** *m/z* calculated for $[M+H]^+$ $C_{12}H_{16}{}^2HO_3$ 210.1235, found 210.1236; **IR ν_{max}/cm^{-1} (thin film)** 2958, 1721, 1449, 1375, 1234, 1147, 1017.

Preparation of (1a*S,1b*R**,4a*S**,5a*R**,*E*)-ethyl 5-(2-(2,4-dinitrophenyl)hydrazono) octahydro-1*H*-cyclopropa[*a*]pentalene-1a-carboxylate, S27**

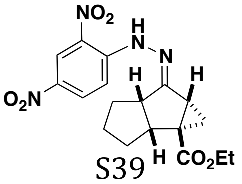

Ketone **19** (20 mg, 0.97 mmol) was dissolved in MeOH (5 mL) and the solution cooled to 0 °C. 2,4-Dinitrophenylhydrazine (190 mg, 50% in H_2O, 0.48 mmol) and H_2SO_4 (0.01 mL) were added and the reaction mixture was stirred at 0 °C for 30 min, After this time, the reaction mixture was let warm to RT, and stirred at this temperature for 90 min. The reaction mixture was diluted with Et_2O and passed through a small plug of silica, eluting with Et_2O. The reaction mixture was concentrated under reduced pressure to afford the crude product. This was purified by flash column chromatography (SiO_2, pet 40-60:Et_2O (8:2)) to afford compound **S27** (25 mg, 66%). X-ray diffraction quality crystals were grown by vapour diffusion from EtOAc and pentanes. An X-ray crystal structure confirmed the identity of this product (CCDC 1501148).

Preparation of (1a*R,1b*R**,4a*S**,5a*S**,*Z*)-ethyl 5-(2-(2,4-dinitrophenyl)hydrazono) octahydro-1*H*-cyclopropa[*a*]pentalene-1a-carboxylate, S28**

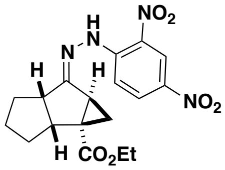

Ketone **12** (20 mg, 0.97 mmol) was dissolved in MeOH (5 mL) and the solution cooled to 0 °C. 2,4-Dinitrophenylhydrazine (190 mg, 50% in H_2O, 0.48 mmol) and H_2SO_4 (0.01 mL) were added and the reaction mixture was stirred at 0 °C for 30 min, After this time, the reaction mixture was let warm to RT, and stirred at this temperature for 90 min. The reaction mixture was diluted with Et_2O and passed through a small plug of silica, eluting with Et_2O. The reaction mixture was concentrated under reduced pressure to afford the crude product. This was purified by flash column chromatography (SiO_2, pet 40-60:Et_2O (8:2)) to afford compound **S28** (28 mg, 74%). X-ray diffraction quality crystals were grown by vapour diffusion from EtOAc and pentanes. An X-ray crystal structure confirmed the identity of this product (CCDC 1501149).

1.3.4 Experimental elucidation of the mechanism

Investigation of (*E*)- to (*Z*)-isomerization of bromoacrylates 5a

Treatment of either isomer with bromide led to no interconversion of bromoacrylates:

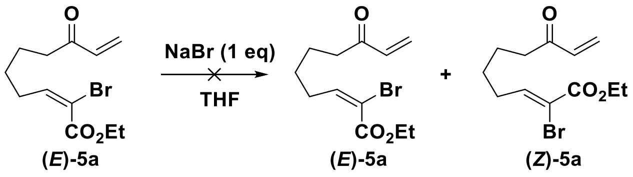

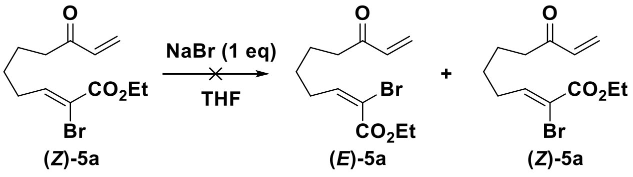

Altering the size of the counter-cation:

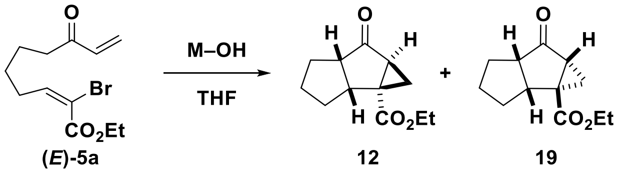

Cyclization precursor **(*E*)-5a** (10 mg, 0.034 mmol) was dissolved in THF (1 mL) and solid M–OH (0.07 mmol) was added. The reaction mixture was stirred until TLC showed complete consumption of starting material. The crude reaction mixture was filtered through a small plug of silica, eluting with Et_2O. The filtrate was concentrated under reduced pressure and subsequent ^1H NMR spectroscopic analysis of the crude reaction product was used to determine the diastereomeric ratio.

Base (M–OH)	Product ratio **(12** : **19)**
NaOH	>95 : 5
KOH	>95 :5
RbOH·$x$$H_2O$	>95 : 5
CsOH·H_2O	61 : 39

Variation of the solvent:

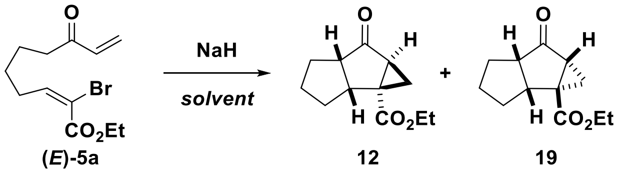

Cyclization precursor **(*E*)-5a** (10 mg, 0.034 mmol) was dissolved in solvent (1 mL) and NaH (2.8 mg, 0.07 mmol) was added. The reaction mixture was stirred until TLC showed complete consumption of starting material. The crude reaction mixture was filtered through a small plug of silica, eluting with Et_2O. The filtrate was concentrated under reduced pressure and subsequent ^{1}H NMR spectroscopic analysis of the crude reaction product was used to determine the diastereomeric ratio.

Solvent	Product ratio **(12** : **19)**
THF	>95 : 5
MeCN	40 : 60
DMF	<5 : 95
DMSO	<5 : 95

Sequestration of the cation:

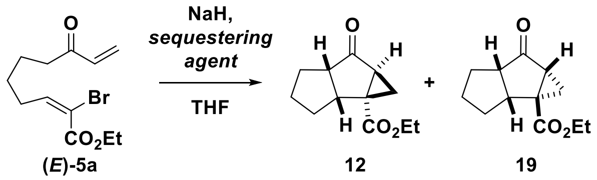

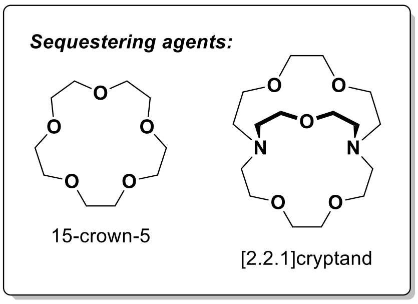

NaH (2.8 mg, 0.07 mmol) was dissolved in THF (0.5 mL) and the Na^+ sequestering agent (0-2 equiv.) was added. The resulting solution was stirred for one hour. After this time, cyclization precursor **(*E*)-5a** (10 mg, 0.034 mmol) was dissolved in THF (0.5 mL) and this was added to the reaction mixture. The resulting solution was stirred until TLC showed complete consumption of starting material. The crude reaction mixture was filtered through a small plug of silica, eluting with Et_2O. The filtrate was concentrated under reduced pressure

and the subsequent ^{1}H NMR spectroscopic analysis of the crude reaction product was used to determine the diastereomeric ratio.

Sequestering agent	Equivalents wrt NaH	Product ratio **(12** : **19)**
15-crown-5	0	>95 : 5
15-crown-5	1	71 : 29
15-crown-5	2	60 : 40
[2.2.1]cryptand	1	<5 : 95

Hydrazone derivatisation of 6,5-bicyclic system products 9:

Preparation of (3a*S,7a*R**,*E*)-diisopropyl 7-(2-(2,4-dinitrophenyl)hydrazono) hexahydro-1*H*-indene-4,4(2*H*)-dicarboxylate, S29 and (3a*S**,7a*S**,*E*)-diisopropyl 7-(2-(2,4-dinitrophenyl)hydrazono) hexahydro-1*H*-indene-4,4(2*H*)-dicarboxylate, S30**

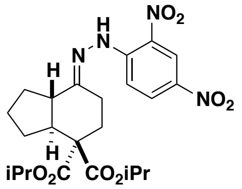

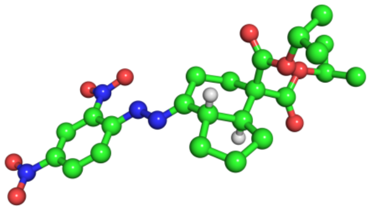

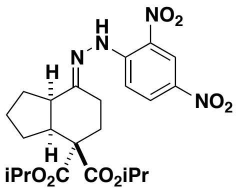

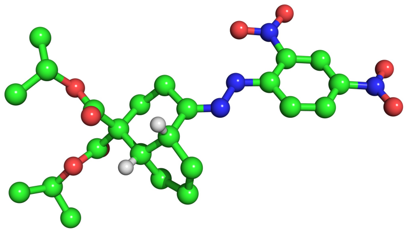

Ketone **9** (20 mg, 0.06 mmol) was dissolved in MeOH (3 mL) and the solution cooled to 0 °C. 2,4-Dinitrophenylhydrazine (119 mg, 50% in H_2O, 0.30 mmol) and H_2SO_4 (1 drop) were added and the reaction mixture was stirred at 0 °C for 30 min, After this time, the reaction mixture was let warm to RT, and stirred at this temperature for 90 min. The reaction mixture was diluted with Et_2O and passed through a small plug of silica, eluting with Et_2O. The reaction mixture was concentrated under reduced pressure to afford the crude products. The mixture was purified by flash column chromatography (SiO_2, pet 40-60:Et_2O (8:2)) to separate diastereomers **S29** and **S30**. X-ray diffraction quality crystals were grown of each diastereomer by vapor diffusion from EtOAc and pentanes. X-ray crystal structures confirmed the identity of the products (**S29**: CCDC 1501147; **S30**: CCDC 1501150).

2. X-Ray Crystallographic Data

X-ray crystallographic data (cif files) can be found in related supporting documentation. Compounds registered with the Cambridge Crystallographic Data Centre (CCDC) have the following summary data:

Summary of Data CCDC 1501147 (**S29)**

Compound Name:
Formula: C23 H30 N4 O8
Unit Cell Parameters: a 8.6692(2) b 9.3702(2) c 16.0180(4) P-1

Summary of Data CCDC 1501148 **(S27)**

Compound Name:
Formula: C18 H20 N4 O6
Unit Cell Parameters: a 19.3518(5) b 6.3277(1) c 16.0896(4) P21/c

----

Summary of Data CCDC 1501149 **(S28)**

Compound Name:
Formula: C18 H20 N4 O6
Unit Cell Parameters: a 7.289(7) b 11.811(11) c 21.18(2) P21/n

----

Summary of Data CCDC 1501150 **(S30)**

Compound Name:
Formula: C23 H30 N4 O8
Unit Cell Parameters: a 14.7496(4) b 8.4562(2) c 38.6953(12) P21/n

----

References

[1] J. He, K. Techabanenko, R. M. Adlington, A. R. Cowley, J. E. Baldwin, *Eur. J. Org. Chem.* **2006**, 4003.

[2] C. Patois, P. Savignac, E. About-Jaudet, N. Collignon, *Synth. Commun.* **1991**, *21*, 2391; a recently reported alternative synthesis was also employed, see F. Messik, M. Oberthür, *Synthesis* **2013**, *45*, 167.

[3] C. Milite, S. Castellano, R. Benedetti, A. Tosco, C. Ciliberti, C. Vicidomini, L. Bailly, G. Franci L. Altucci, A. Mai and G. Sbardella, *Bioorg. Med. Chem.* **2011**, *19*, 3690

[4] J. E. Yeo, X. Yang, H. J. Kim and S. Koo, *Chem. Commun.* **2004**, *2*, 236

[5] J. Sheng, X. Li, M. Tang, B. Gao and G. Huang, *Synthesis* **2007**, *8*, 1165

[6] P. Fourgeaud, C. Midrier, J.-P. Vors, J.-N. Volle, J.-L. Pirat and D. Virieux, *Tetrahedron* **2010,** *66*, 758

Computational Supporting Information

A Cation-Directed Approach to the Diels-Alder Paradigm: Cascade Synthesis of Tricyclic Fused Cyclopropanes

Table of Contents

1. Computational Methods S47
2. Benchmarking Studies S49
3. Comparison of cyclization in THF and Toluene S50
4. 2D potential energy surface S51
5. Cyclopropane formation S52
8. Cartesian Coordinates S54

1. Computational Methods

Density functional theory (DFT) calculations were performed with *Gaussian09* rev D.01.[1] The hybrid meta-generalized gradient approximation (meta-GGA) M06-2X exchange-correlation functional[2] was used for all geometry optimizations with a fine integration grid. The 6-311++G(d,p) basis set was used for all elements apart from Br, which was described with the LANL08 uncontracted basis set/relativistic ECP.[3] Effects of solvation were included for all calculations (including geometry optimization) with a conductor-like polarizable continuum model (CPCM).[4] All stationary points were verified as either minima or saddle points by the presence of zero or a single imaginary harmonic vibrational frequency, respectively. This level of theory has been applied previously to anionic cyclizations of enolates[5] and was also compared against composite CBS-Q//B3 calculations,[6] giving results within 1 kcal/mol of the *ab initio* benchmark. Relative free energies were evaluated at 298.15 K applying a haptic translational entropy correction to adjust

---

[1] Gaussian 09, Revision D.01, Frisch, M. J.; Trucks, G. W.; Schlegel, H. B.; Scuseria, G. E.; Robb, M. A.; Cheeseman, J. R.; Scalmani, G.; Barone, V.; Mennucci, B.; Petersson, G. A.; Nakatsuji, H.; Caricato, M.; Li, X.; Hratchian, H. P.; Izmaylov, A. F.; Bloino, J.; Zheng, G.; Sonnenberg, J. L.; Hada, M.; Ehara, M.; Toyota, K.; Fukuda, R.; Hasegawa, J.; Ishida, M.; Nakajima, T.; Honda, Y.; Kitao, O.; Nakai, H.; Vreven, T.; Montgomery, J. A., Jr.; Peralta, J. E.; Ogliaro, F.; Bearpark, M.; Heyd, J. J.; Brothers, E.; Kudin, K. N.; Staroverov, V. N.; Kobayashi, R.; Normand, J.; Raghavachari, K.; Rendell, A.; Burant, J. C.; Iyengar, S. S.; Tomasi, J.; Cossi, M.; Rega, N.; Millam, J. M.; Klene, M.; Knox, J. E.; Cross, J. B.; Bakken, V.; Adamo, C.; Jaramillo, J.; Gomperts, R.; Stratmann, R. E.; Yazyev, O.; Austin, A. J.; Cammi, R.; Pomelli, C.; Ochterski, J. W.; Martin, R. L.; Morokuma, K.; Zakrzewski, V. G.; Voth, G. A.; Salvador, P.; Dannenberg, J. J.; Dapprich, S.; Daniels, A. D.; Farkas, Ö.; Foresman, J. B.; Ortiz, J. V.; Cioslowski, J.; Fox, D. J. Gaussian, Inc., Wallingford CT, 2009.

[2] Zhao, Y.; Truhlar, D. G. *Theor. Chem. Acc.* **2008**, *120*, 215.

[3] Roy, L. E.; Hay, P. J.; Martin, R. L. *J. Chem. Theory Comput*. **2008**, *4*, 1029.

[4] (a) Barone, V.; Cossi, M. *J. Phys. Chem. A* **1998**, *102*, 1995; (b) Cossi, M.; Rega, N.; Scalmani, G.; Barone, V. *J. Comput. Chem.* **2003**, *24*, 669; (c) Solute cavities were defined by UFF atomic radii: Takano, Y.; Houk, K. N. *J. Chem. Theory. Comput.* **2005**, *1*, 70.

[5] (a) Johnston, C. P.; Kothari, A.; Sergeieva, T.; Okovytyy, S. I.; Jackson, K. E.; Paton, R. S.; Smith, M. D. *Nature Chem.* **2015**, *7*, 171; (b) Peng, Q.; Paton, R. S. *Acc. Chem. Res.* **2016**, *49*, 1042.

[6] (a) Montgomery Jr., J. A.; Frisch, M. J.; Ochterski, J. W.; Petersson, G. A. *J. Chem. Phys.* **1999**, *110*, 2822; (b) Montgomery Jr., J. A.; Frisch, M. J.; Ochterski, J. W.; Petersson, G. A. *J. Chem. Phys.* **2000**, *112*, 6532.

from a standard-state of 1 atm to 1 mol/l, while a quasi-harmonic approximation was used in which the treatment of vibrational entropies switches from a rigid-rotor harmonic oscillator to a free rotor at frequencies below 100 cm^{-1}, implemented in Python as described previously.[7] All *Gaussian* output files have been made openly accessible for download using a CC0 license for reuse: http://dx.doi.org/10.5281/zenodo.60147

[7] (a) Funes-Ardoiz, I.; Paton, R. S. GoodVibes v1.0.0 DOI: 10.5281/zenodo.56091 (accessed 21st June 2016); (b) Choi, H.; Min, M.; Peng, Q.; Kang, D.; Paton, R.S.; Hong, S. *Chem. Sci.* **2016**, *7*, 3900; (c) Simón, L.; Paton, R. S. *Org. Biomol. Chem.* **2016**, *14*, 3031; (d) Cortopassi, W. E.; Simion, R.; Hornsby, C. E.; Costa Franca, T. C.; Paton, R. S. *Chem. Eur. J.* **2015**, *21*, 18983; (e) Jackson, K. E.; Mortimer, C. L.; Odell, B.; McKenna, J. M.; Claridge, T. D. W.; Paton, R. S.; Hodgson, D. M. *J. Org. Chem.* **2015**, *80*, 9838.

2. Benchmarking Studies

For a series of concerted and stepwise intramolecular [4 + 2] cycloadditions we compared the energy profile obtained from DFT calculations (M06-2X and B3LYP were examined) with CBS-Q//B3 results (**Fig. S1**). The M06-2X results were far superior to those obtained with B3LYP, and for the stepwise cycloaddition had a root-mean-squared error (RMSE) of 3.4 kJ/mol. Additionally, a comparison of relative energies of TS conformers showed an RMSE of 2.3 kJ/mol in a comparison of M06-2X with CBS-Q//B3.

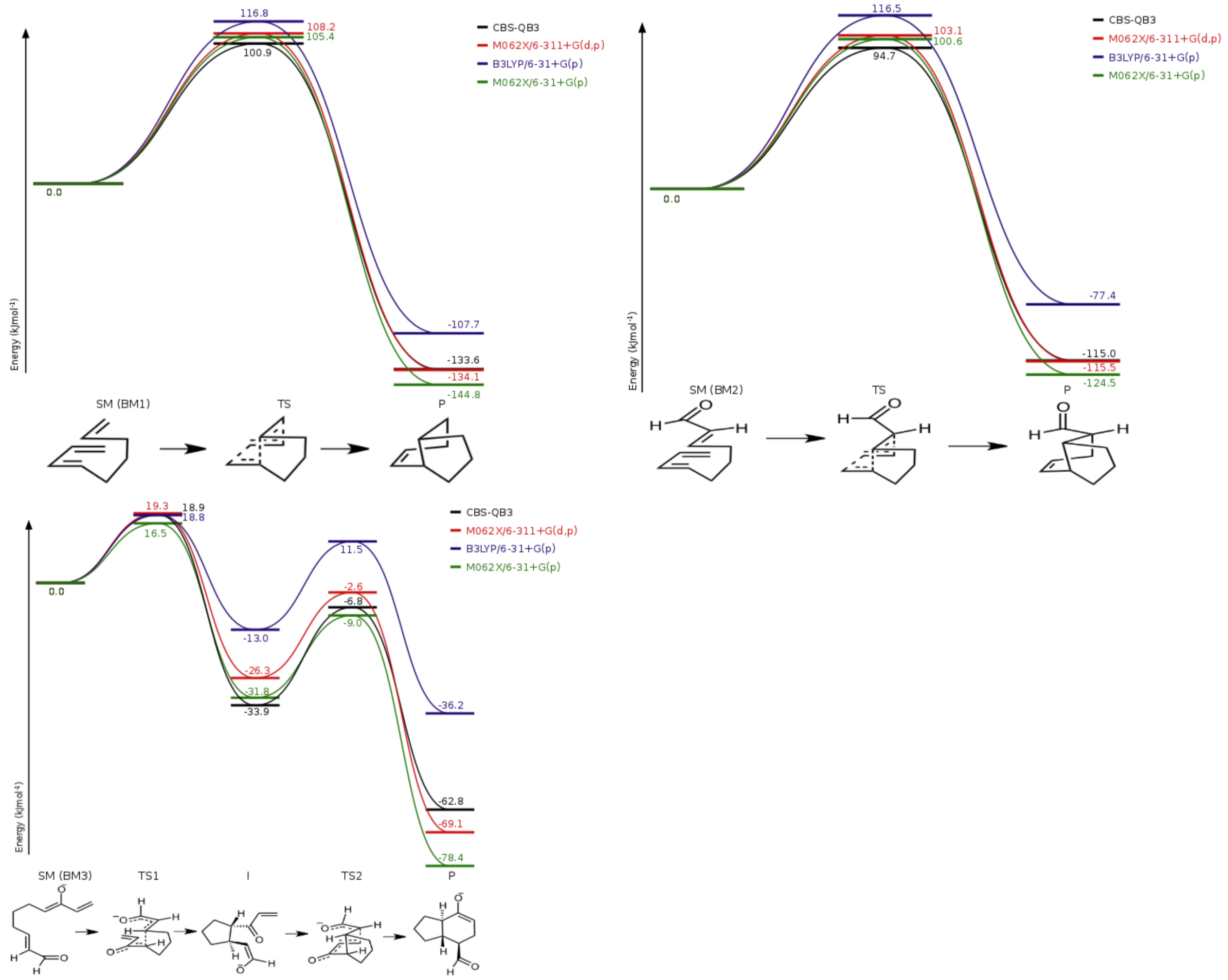

Figure S1. Comparison of energy profiles for concerted and stepwise intramolecular [4 + 2]-cycloadditions at different levels of theory.

3. Comparison of cyclization in THF and Toluene

Separate optimizations for the cyclization were performed in THF and Toluene solvent. The stepwise nature of the [4 + 2] cycloaddition and the *trans*-diastereoselectivity of the first step was consistent across both sets of calculations.

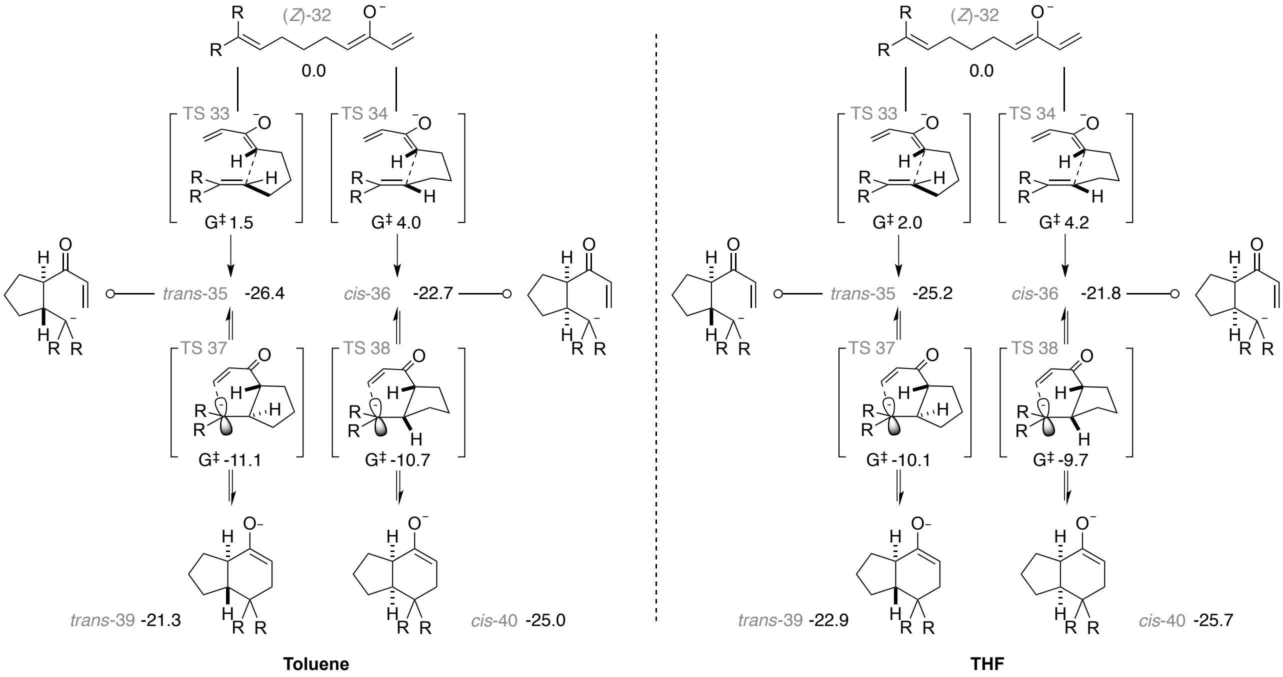

Figure S2. Trans-selective stepwise cyclization found in both solvents.

4. 2D potential energy surface

To confirm the absence of a concerted intramolecular [4 + 2] TS we performed 2-dimensional scans of the PES, constraining the two forming C-C bonds in regular increments at the CPCM-M06-2X/6-311++G(d,p) level of theory. As shown in **Fig. S3** there are two distinct saddle-points corresponding to the stepwise formation of the two C-C bonds, confirming the absence of a concerted TS in this reaction.

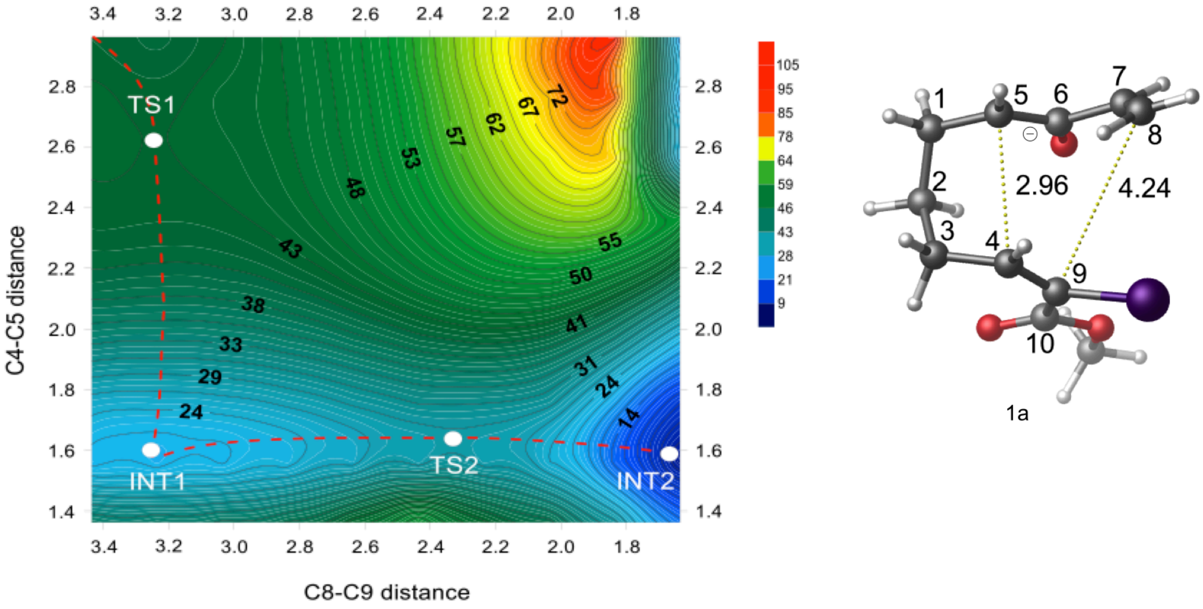

Figure S3. 2D-PES for the intramolecular cycloaddition showing the presence of 2 separate saddle-points corresponding to stepwise C-C formation.

5. Cyclopropane formation

Key transition structures are shown for the Michael-Michael-cyclopropanation sequence of steps starting from either alkene stereochemistry, with forming/breaking bond distances in Å.

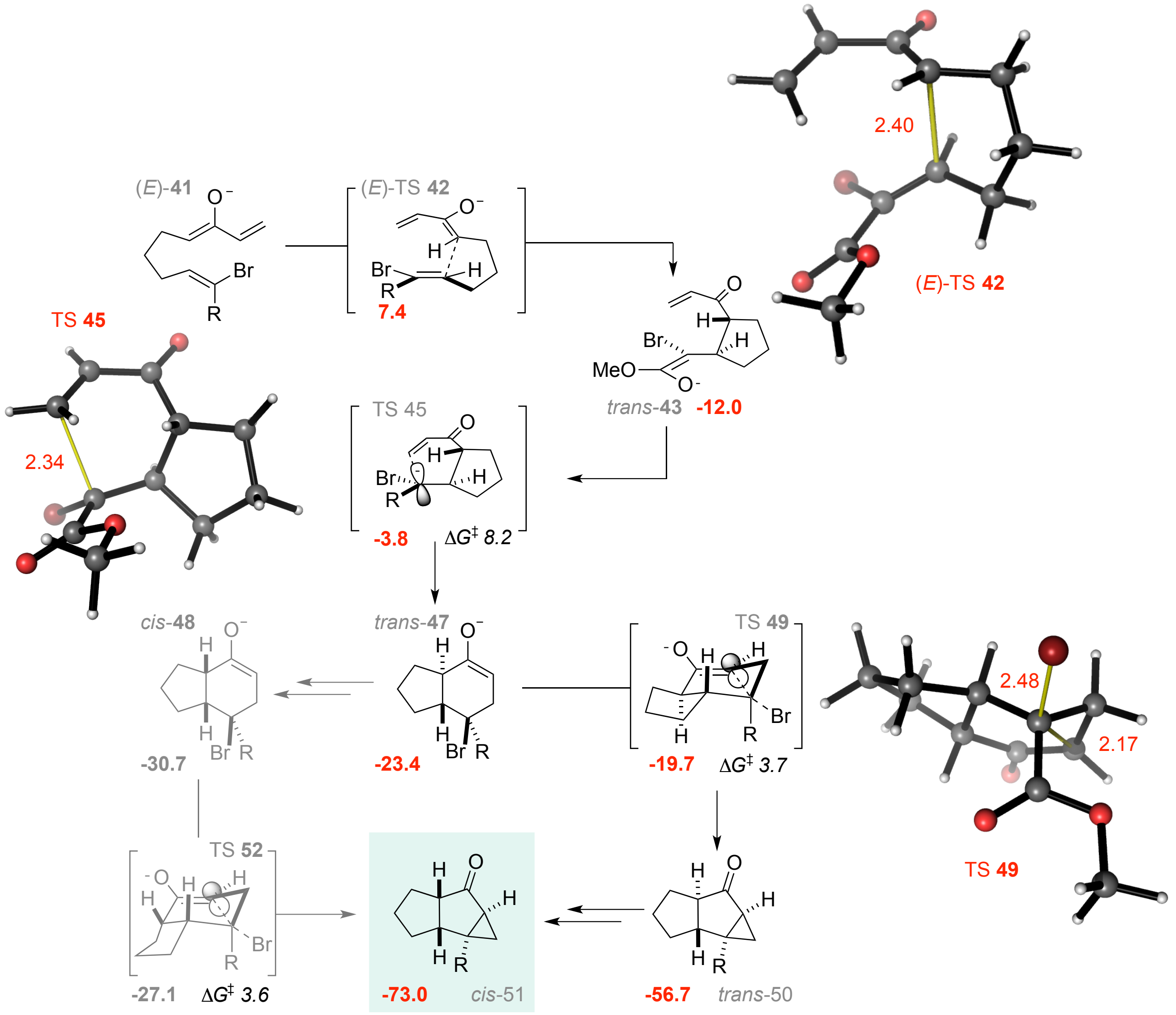

Figure S4. TS structures obtained for the cyclization from an (*E*)-enolate starting geometry.

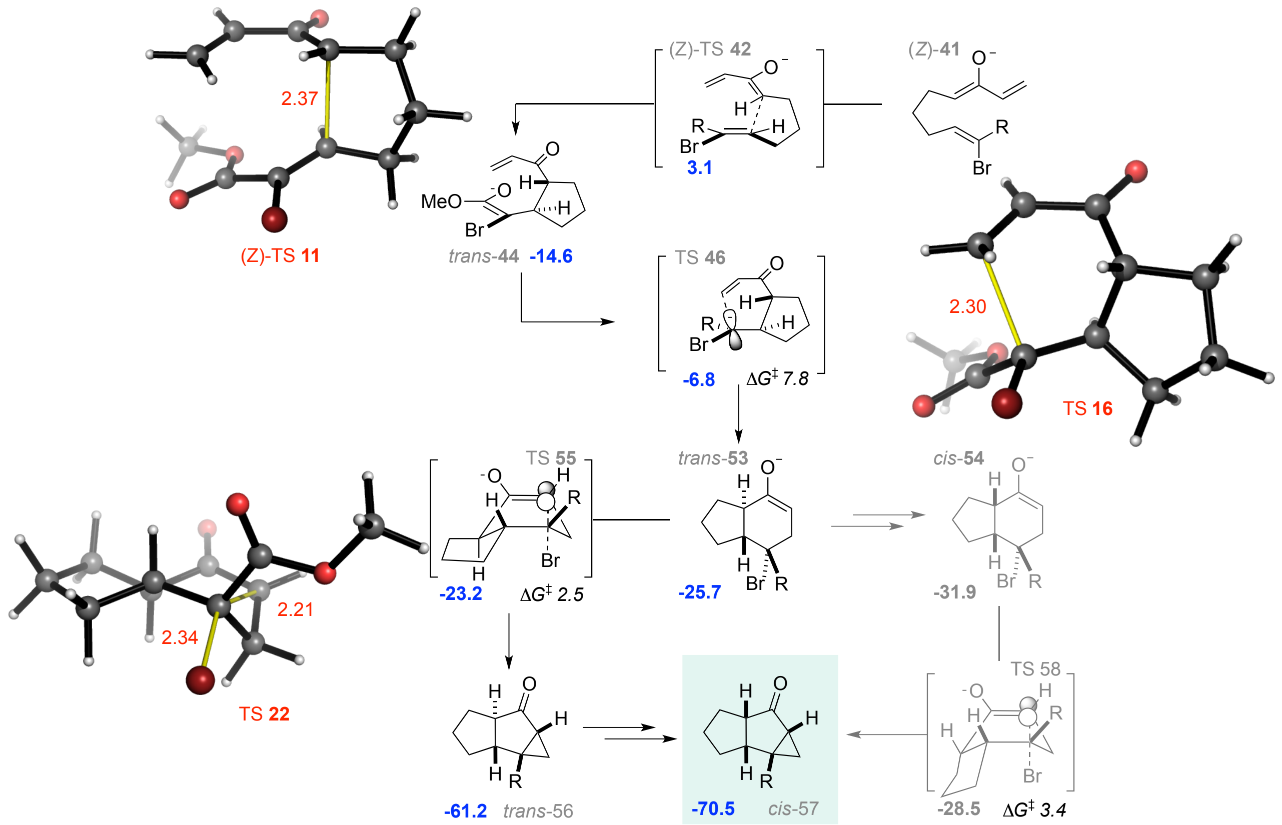

Figure S5. TS structures obtained for the cyclization from a (*Z*)-enolate starting geometry.

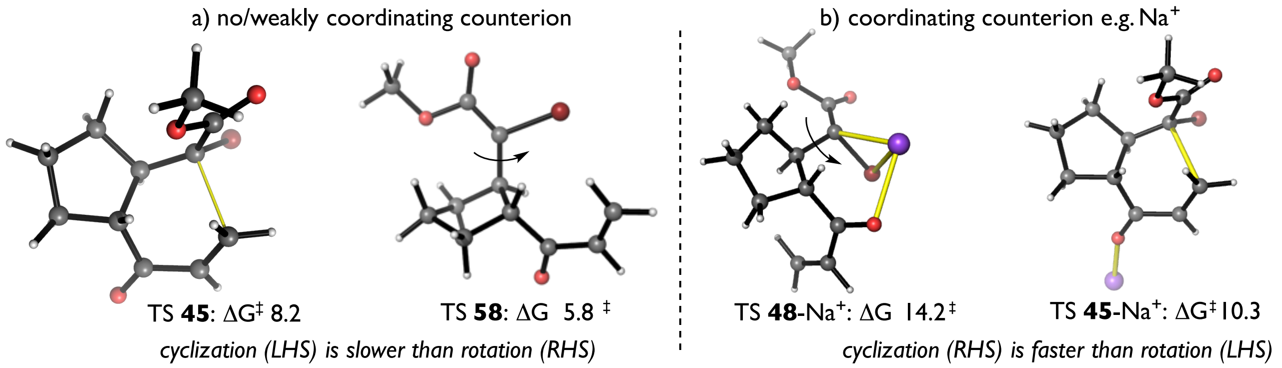

Figure S6. CPCM-M062X/6-311+G(d,p) C-C rotation between *trans*-**12** and *trans*-**14**: a) without and b) with a coordinating counterion. Structures were obtained from a dihedral scan about the exocyclic C-C bond. The direction of rotation was considered in either sense, and the more facile sense of rotation is indicated above. The barrier for rotation is compared against the competing cyclization step in each case.

8. Cartesian Coordinates

Geometries and absolute values (Hartree) for SCF energy, zero-point vibrational energy (ZPE), enthalpy, RRHO Gibbs energy and quasi-harmonic Gibbs energy (at 298.15 K) for all intermediates and transition structures.

(Z)-32:

E: -881.709654
ZPE: 0.284059
H: -881.405212
qh-G: -881.471089

-1 1
C 1.021953 3.064518 -0.073244
C -0.484052 2.908871 -0.336782
C -0.825356 1.796326 -1.346637
C -0.23045 0.459418 -1.02213
C 1.582777 1.95282 0.764185
C 2.477272 1.020973 0.298886
C 2.840831 -0.118574 1.206693
C 2.008221 -0.759757 2.028749
C -0.867219 -0.606169 -0.500606
O 3.017439 0.985543 -0.871211
C -2.292677 -0.587901 -0.072758
H 1.213105 1.858145 1.780764
H 0.809659 0.318963 -1.304955
H 1.175065 4.039037 0.409371
H 1.556889 3.097669 -1.028498
H -0.901866 3.843727 -0.726584
H -0.997687 2.690872 0.604904
H -0.424683 2.09297 -2.322213
H -1.910206 1.722115 -1.441712
H 3.862594 -0.480454 1.093286
H 2.334531 -1.60676 2.624152
H 0.969054 -0.45798 2.122045
C -0.156875 -1.890255 -0.278054
O -3.096866 -1.455994 -0.31244
O -2.603768 0.518995 0.61309
C -3.976119 0.650197 0.998039
O -0.514526 -2.74123 0.505217
O 0.943474 -2.008272 -1.025803
C 1.78202 -3.126243 -0.72919
H -4.043952 1.597424 1.525987
H -4.616808 0.656974 0.115946
H -4.265114 -0.173522 1.650494
H 2.63344 -3.03959 -1.399081
H 2.109556 -3.077076 0.309782
H 1.248442 -4.060201 -0.907413

TS-33:

E: -881.707468
ZPE: 0.283977
H: -881.404001
qh-G: -881.467933

-1 1
C 0.130659 3.153918 -0.074821
C -1.270741 2.761489 -0.5453
C -1.19371 1.528951 -1.457287
C -0.233042 0.460716 -0.987487
C 0.715425 2.031759 0.724879
C 1.980266 1.499422 0.51863
C 2.419158 0.370612 1.404199
C 1.637369 -0.438253 2.121773
C -0.570939 -0.780168 -0.505787
O 2.791233 1.837271 -0.405373
C -1.926057 -1.107961 -0.030109
H 0.157614 1.718168 1.600973
H 0.777031 0.555339 -1.368134
H 0.067658 4.083878 0.506429
H 0.773063 3.358749 -0.937376
H -1.767207 3.579993 -1.076286
H -1.884162 2.508921 0.325302
H -0.831823 1.853722 -2.438835
H -2.190512 1.108668 -1.595901
H 3.492214 0.186838 1.377655
H 2.055665 -1.257263 2.697556
H 0.558136 -0.323861 2.142999
C 0.438198 -1.845359 -0.401518
O -2.477806 -2.183776 -0.107048
O -2.540564 -0.038547 0.517558
C -3.902168 -0.229187 0.900801
O 0.315961 -2.888579 0.207502
O 1.571126 -1.549864 -1.070178
C 2.671372 -2.423905 -0.837299
H -4.240659 0.733201 1.276736
H -4.501781 -0.533261 0.041881
H -3.978691 -0.988797 1.679194

H 3.503867 -2.00745 -1.399597
H 2.909748 -2.448111 0.227397
H 2.445492 -3.432971 -1.183716

TS-34:

E: -881.703435
ZPE: 0.283443
H: -881.400487
qh-G: -881.464346

-1 1
C -1.992595 -2.45434 -0.54589
C -2.538682 -1.096894 -1.000279
C -1.511924 -0.424326 -1.916598
C -0.152774 -0.215636 -1.294325
C -0.592068 -2.338661 -0.016224
C -0.335459 -1.694273 1.191165
C 1.0588 -1.70236 1.74387
C 2.189976 -1.900312 1.06801
C 0.288016 0.820908 -0.492392
O -1.187592 -1.035978 1.858853
C 1.737645 1.002432 -0.3004
O 2.474209 0.254368 -1.165315
O 2.287567 1.754834 0.475292
H 0.179019 -2.972118 -0.438219
H 0.645912 -0.773437 -1.763802
H -2.008977 -3.150777 -1.391209
H -2.668177 -2.860878 0.218866
H -2.708469 -0.47146 -0.12565
H -3.489001 -1.210998 -1.533199
H -1.897596 0.537773 -2.25777
H -1.369409 -1.063071 -2.794618
H 1.104604 -1.46469 2.805186
H 3.153452 -1.860403 1.565527
H 2.190686 -2.090587 -0.000141
C -0.59167 1.732995 0.255987
C 3.884013 0.316856 -0.981792
H 4.308939 -0.36507 -1.715103
H 4.253833 1.32926 -1.148349
H 4.152547 0.000142 0.027586
O -0.286656 2.438126 1.194084
O -1.850601 1.753808 -0.240734
C -2.80605 2.477124 0.52839
H -3.758891 2.340239 0.021687
H -2.857363 2.077467 1.542632
H -2.549891 3.536262 0.571988

***trans*-35:**

E: -881.752079
ZPE: 0.285875
H: -881.446486
qh-G: -881.511283

-1 1
C -1.450171 2.604246 -0.182827
C -2.467502 1.810331 -1.008173
C -1.607231 0.781454 -1.757388
C -0.333785 0.545403 -0.88414
C -0.527698 1.508332 0.348186
C 0.8122 1.992746 0.833062
C 1.578769 1.188506 1.822945
C 1.143213 0.082312 2.424211
C 0.01281 -0.883547 -0.539842
O 1.318207 3.016969 0.399479
C -0.9511 -1.742604 0.059644
H -1.04257 0.953027 1.133188
H 0.508442 0.9498 -1.443522
H -1.90161 3.200252 0.614167
H -0.877185 3.27722 -0.827875
H -3.059922 2.441125 -1.674655
H -3.152457 1.288198 -0.333917
H -1.302635 1.182707 -2.728258
H -2.14695 -0.14714 -1.936563
H 2.572207 1.57738 2.029873
H 1.774922 -0.449964 3.126746
H 0.162261 -0.336155 2.232077
C 1.363902 -1.328021 -0.605504
O -0.876355 -2.929429 0.370628
O -2.132464 -1.066776 0.32615
C -3.181883 -1.841824 0.880275
O 1.844368 -2.421287 -0.3123
O 2.224488 -0.334235 -1.047413
C 3.600417 -0.674006 -1.052948
H -4.02733 -1.16373 0.990043
H -3.45346 -2.670044 0.222575
H -2.903538 -2.247464 1.855019
H 4.126622 0.214957 -1.397797
H 3.943462 -0.943032 -0.051567
H 3.802152 -1.509041 -1.726891

***cis*-36:**

E: -881.748056
ZPE: 0.286480
H: -881.442322

qh-G: -881.505799

-1 1
C -1.921777 -2.377917 0.175193
C -2.18288 -1.922918 -1.265007
C -0.817382 -1.419011 -1.759207
C 0.022516 -1.090807 -0.484307
C -0.977583 -1.304512 0.724593
C -1.750688 -0.104116 1.241793
C -1.041393 0.924988 2.052993
C 0.222907 0.844994 2.462793
C 0.87851 0.150297 -0.557807
O -2.958289 0.004978 1.103693
C 2.271511 0.098403 -0.276407
O 2.685517 -1.156995 0.153393
O 3.116506 0.989208 -0.345807
H -0.399081 -1.676309 1.576193
H 0.71522 -1.920904 -0.361407
H -1.399973 -3.340614 0.175193
H -2.833977 -2.479921 0.764593
H -2.895484 -1.095221 -1.244807
H -2.598976 -2.71622 -1.889907
H -0.918986 -0.546512 -2.401707
H -0.300778 -2.194409 -2.332807
H -1.659497 1.777785 2.320593
H 0.663203 1.633296 3.063293
H 0.859511 0.005797 2.207593
C 0.281804 1.422394 -0.783407
C 4.072017 -1.283488 0.423593
H 4.228622 -2.322987 0.708093
H 4.670216 -1.045185 -0.457807
H 4.377914 -0.626286 1.240093
O 0.778299 2.543196 -0.859807
O -1.093095 1.299987 -0.885207
C -1.831101 2.508384 -0.933007
H -2.8803 2.215979 -0.940907
H -1.623804 3.126285 -0.056207
H -1.594104 3.082285 -1.831107

TS 37:

E: -881.731027
ZPE: 0.286725
H: -881.426171
qh-G: -881.487182

-1 1
C -2.69172 -1.343055 0.611305
C -2.755097 -0.162854 1.587805
C -1.27929 0.168917 1.890105
C -0.461001 -0.398199 0.691805
C -1.545012 -0.953178 -0.309495
C -0.895834 -2.053091 -1.123795
C 0.254575 -1.610013 -1.839695
C 0.550202 -0.241119 -1.874895
C 0.608316 0.48828 0.077005
O -1.256058 -3.242983 -1.007695
C 0.276244 1.881986 -0.210095
H -1.865196 -0.130971 -0.955195
H 0.055081 -1.286409 1.053205
H -3.629123 -1.513536 0.077105
H -2.435438 -2.26826 1.137505
H -3.332501 -0.380842 2.488805
H -3.22398 0.690556 1.088405
H -0.9579 -0.323189 2.811705
H -1.129669 1.240014 2.024305
H 1.013861 -2.344228 -2.088795
H 1.507408 0.078562 -2.279595
H -0.252484 0.461097 -2.076095
C 2.015311 0.208252 0.350805
O 1.018162 2.818372 -0.433995
O -1.074353 2.042313 -0.303995
C -1.526926 3.352522 -0.629695
O 2.979525 0.911232 0.104705
O 2.198786 -1.036451 0.869905
C 3.553579 -1.431879 1.059205
H -2.612728 3.291943 -0.658795
H -1.210212 4.069916 0.128905
H -1.14062 3.667414 -1.599995
H 3.513459 -2.439578 1.466505
H 4.093379 -1.42929 0.111305
H 4.058092 -0.762489 1.757105

TS 38:

E: -881.730209
ZPE: 0.286462
H: -881.425620
qh-G: -881.486524

-1 1
C 2.323369 1.900054 -0.175239
C 1.472904 2.682083 -1.179276
C 0.354448 1.691591 -1.506849
C -0.02594 1.095956 -0.135498
C 1.309665 1.19203 0.733824

C 1.877569 -0.067581 1.389296
C 0.921464 -0.964302 1.954911
C -0.435006 -0.666405 1.851709
C -0.788671 -0.217304 -0.169811
O 3.104112 -0.275714 1.373823
C -2.241167 -0.195549 -0.167511
O -2.733487 0.958997 0.377224
O -3.01228 -1.068979 -0.526883
H 1.061181 1.852624 1.575099
H -0.708502 1.811508 0.322074
H 3.024194 2.523102 0.383497
H 2.906274 1.139046 -0.701594
H 2.037608 3.001352 -2.058209
H 1.053612 3.577425 -0.706068
H 0.738134 0.9108 -2.166687
H -0.510362 2.153807 -1.990081
H 1.244665 -1.987046 2.122828
H -1.159139 -1.443941 2.084714
H -0.795271 0.338121 2.056177
C -0.150228 -1.396344 -0.734385
C -4.150767 1.034459 0.484935
H -4.363405 2.006962 0.923783
H -4.619409 0.953625 -0.496722
H -4.534314 0.24037 1.127137
O -0.600972 -2.519235 -0.871892
O 1.156551 -1.140698 -0.994062
C 1.983513 -2.269756 -1.248533
H 2.986849 -1.87492 -1.39079
H 1.966729 -2.949601 -0.394755
H 1.652841 -2.801797 -2.141511

***trans*-39:**

E: -881.753526
ZPE: 0.289048
H: -881.446203
qh-G: -881.507537

-1 1
C -2.819074 0.34024 1.255388
C -1.921081 1.307127 2.046085
C -0.476518 0.785866 1.835751
C -0.581013 -0.174983 0.6265
C -1.979988 0.045819 0.019026
C -2.327183 -1.170843 -0.847933
C -1.310476 -1.489716 -1.721199
C -0.116405 -0.573425 -1.845801
C 0.520938 -0.186187 -0.463615
O -3.434574 -1.783336 -0.65561
C 1.204068 1.163148 -0.607903
H -1.947181 0.948923 -0.60611
H -0.607458 -1.194417 1.023141
H -3.799466 0.761098 1.025099
H -2.978312 -0.588379 1.813358
H -2.190374 1.377545 3.101965
H -2.015588 2.309489 1.61697
H -0.119581 0.241995 2.712161
H 0.22186 1.60936 1.671599
H -1.385707 -2.351664 -2.376347
H 0.664061 -1.024222 -2.460566
H -0.386334 0.374725 -2.328297
C 1.625848 -1.168014 -0.08317
O 2.39886 1.343073 -0.59323
O 0.326407 2.152907 -0.786996
C 0.882092 3.464021 -0.935378
O 2.158791 -1.953968 -0.823726
O 1.957658 -1.051056 1.207783
C 3.030703 -1.883855 1.65647
H 0.033671 4.131188 -1.05913
H 1.453648 3.732458 -0.046569
H 1.530253 3.502167 -1.81073
H 3.156751 -1.661447 2.712456
H 2.779236 -2.934886 1.514665
H 3.941903 -1.650068 1.105255

***cis*-40:**

E: -881.758891
ZPE: 0.289465
H: -881.451367
qh-G: -881.512026

-1 1
C -2.352726 -1.534012 -0.582929
C -1.428635 -2.379241 -1.466094
C -0.06687 -1.676206 -1.358821
C -0.016613 -1.14931 0.09532
C -1.479788 -1.212185 0.639191
C -1.954319 -0.038193 1.508509
C -0.988213 0.748236 2.089711
C 0.486462 0.572938 1.810856
C 0.666792 0.220624 0.314476
O -3.223802 0.075543 1.657931
C 2.111958 0.071814 -0.122897
O 2.862249 -0.546808 0.79376

O 2.543938 0.412902 -1.198232
H -1.525614 -2.090309 1.297585
H 0.591211 -1.836759 0.690644
H -3.269551 -2.056068 -0.307093
H -2.629704 -0.611164 -1.0958
H -1.775457 -2.467969 -2.498116
H -1.35576 -3.391454 -1.052758
H -0.019289 -0.84992 -2.069145
H 0.770294 -2.34377 -1.577557
H -1.297556 1.557134 2.744872
H 1.031314 1.496184 2.025819
H 0.968524 -0.221928 2.39319
C 0.09553 1.417921 -0.445689
C 4.218249 -0.809085 0.416621
H 4.666717 -1.319221 1.264492
H 4.246338 -1.441936 -0.470476
H 4.740325 0.125689 0.213278
O 0.545978 2.532521 -0.332911
O -0.951101 1.138158 -1.216903
C -1.621079 2.261752 -1.794201
H -2.461387 1.850296 -2.347394
H -1.972195 2.927393 -1.004829
H -0.950795 2.804958 -2.460201

***E*-41:**

E: -666.369197
ZPE: 0.230607
H: -666.120908
qh-G: -666.182503

-1 1
C 2.02173 2.582256 0.213341
C 0.510007 2.77777 0.015115
C -0.073926 1.948202 -1.143874
C -0.046666 0.466463 -0.898811
C 2.440518 1.244804 0.755001
C 2.963085 0.244309 -0.026472
C 3.389526 -1.032437 0.639821
C 2.740115 -1.677909 1.608877
C -1.019361 -0.300874 -0.390412
O 3.122951 0.2711 -1.306547
C -2.353526 0.113669 0.115049
H 2.374594 1.084946 1.826537
H 0.8682 -0.027732 -1.216553
H 2.368632 3.378089 0.884655
H 2.516032 2.744127 -0.751692
H 0.304741 3.832143 -0.203769
H -0.028754 2.528717 0.934957
H 0.533049 2.135287 -2.034893
H -1.089846 2.282498 -1.347618
H 4.288982 -1.476827 0.214237
H 3.108887 -2.615105 2.013241
H 1.811562 -1.290374 2.016852
O -3.283455 -0.632012 0.306464
O -2.414045 1.426495 0.356392
C -3.676705 1.91721 0.820922
H -3.533731 2.982375 0.980251
H -4.44885 1.742173 0.071846
H -3.952305 1.423094 1.752137
Br -0.739817 -2.198538 -0.292629

***Z*-41:**

E: -666.376547
ZPE: 0.230742
H: -666.128386
qh-G: -666.188964

-1 1
C -1.868941 -2.687683 0.190385
C -0.372409 -2.941954 -0.058923
C 0.211763 -2.104668 -1.212785
C 0.097151 -0.626437 -1.010707
C -2.151939 -1.362786 0.836159
C -2.690072 -0.284762 0.180279
C -2.806374 1.007776 0.936114
C -1.906174 1.502806 1.78716
C 0.997858 0.175441 -0.436884
O -3.082922 -0.2384 -1.048432
H -1.864387 -1.24399 1.876363
H -0.805709 -0.144615 -1.380308
H -2.242273 -3.508757 0.815877
H -2.404217 -2.748214 -0.763481
H -0.20262 -3.996658 -0.302164
H 0.190643 -2.728234 0.856368
H -0.325378 -2.36298 -2.129994
H 1.262584 -2.372135 -1.351044
H -3.681785 1.604002 0.678179
H -2.046981 2.465775 2.268088
H -0.996646 0.957555 2.023219
C 0.797644 1.637252 -0.267982
O 1.419956 2.340686 0.492004
O -0.160319 2.096447 -1.075534
C -0.531198 3.464202 -0.88683
H -1.339716 3.645913 -1.58974

H -0.874965 3.617574 0.136176
H 0.314935 4.118368 -1.098021
Br 2.598255 -0.50196 0.362293

***E*-TS 42:**

E: -666.361608
ZPE: 0.230078
H: -666.114729
qh-G: -666.174074

-1 1
C 2.946669 1.358301 -0.37231
C 2.002725 2.465892 -0.845128
C 0.741579 1.849209 -1.470382
C 0.430094 0.444888 -0.983363
C 2.205364 0.530833 0.630563
C 2.266149 -0.856045 0.718753
C 1.466853 -1.513548 1.803271
C 0.418753 -0.992187 2.443649
C -0.789337 -0.001993 -0.553148
O 2.888311 -1.621445 -0.083241
C -1.840534 0.753787 0.106597
H 1.733865 1.075033 1.44241
H 1.069558 -0.318077 -1.419626
H 3.860897 1.804555 0.040174
H 3.252402 0.726631 -1.213525
H 2.484634 3.143015 -1.555857
H 1.711621 3.064699 0.024468
H 0.906128 1.732974 -2.548376
H -0.116854 2.503319 -1.340885
H 1.765031 -2.539495 2.012746
H -0.12293 -1.563854 3.189545
H 0.056538 0.008083 2.229718
O -2.968198 0.367459 0.349793
O -1.421274 1.986206 0.478813
C -2.406428 2.812909 1.097808
H -1.90607 3.75356 1.314492
H -3.247996 2.978018 0.42411
H -2.767336 2.355145 2.019191
Br -1.180615 -1.876962 -0.743982

***Z*-TS 42:**

E: -666.371396
ZPE: 0.229865
H: -666.124744
qh-G: -666.184050

-1 1
C -2.873526 -1.416243 0.078405
C -1.901169 -2.490082 -0.41128
C -0.927362 -1.848864 -1.40589
C -0.413469 -0.485957 -0.985176
C -2.071032 -0.321018 0.70608
C -2.27454 1.039558 0.482966
C -1.412815 2.005769 1.237486
C -0.314267 1.732069 1.945052
C 0.837464 -0.238946 -0.490009
O -3.077127 1.538848 -0.365895
H -1.438057 -0.616236 1.536631
H -0.86878 0.36787 -1.476888
H -3.591155 -1.859065 0.78145
H -3.448031 -1.01427 -0.761975
H -2.414105 -3.335676 -0.879239
H -1.337232 -2.878247 0.444065
H -1.445161 -1.715761 -2.36029
H -0.076481 -2.511318 -1.582328
H -1.721338 3.043083 1.117509
H 0.257474 2.525993 2.414457
H 0.061728 0.72224 2.070864
C 1.466629 1.068702 -0.447381
O 2.507974 1.355919 0.115551
O 0.773549 1.980854 -1.170087
C 1.19479 3.331465 -1.011891
H 0.523181 3.925628 -1.627218
H 1.10923 3.629856 0.035033
H 2.225578 3.45998 -1.343522
Br 1.790393 -1.628793 0.422992

***trans*-43:**

E: -666.398163
ZPE: 0.232761
H: -666.148495
qh-G: -666.208090

-1 1
C 3.082153 0.170263 -0.386767
C 2.796814 1.376515 -1.291384
C 1.351401 1.169385 -1.794098
C 0.729491 0.042336 -0.915367
C 1.734091 -0.073545 0.286955
C 1.606118 -1.404909 0.974357
C 0.683711 -1.55269 2.130915
C -0.063231 -0.574407 2.641996
C -0.722425 0.198882 -0.582301

O 2.219287 -2.386624 0.582688
C -1.304385 1.22006 0.144686
H 1.518772 0.74179 0.978862
H 0.843787 -0.894608 -1.4688
H 3.889634 0.348474 0.327237
H 3.346219 -0.707697 -0.983079
H 3.521102 1.468953 -2.102938
H 2.853261 2.293448 -0.697753
H 1.327256 0.865133 -2.843132
H 0.766392 2.08204 -1.697394
H 0.638221 -2.559126 2.537424
H -0.731307 -0.769473 3.473664
H -0.048094 0.435332 2.247829
O -2.476592 1.368969 0.542332
O -0.36133 2.210146 0.467515
C -0.89566 3.395919 1.02987
H -0.046149 4.05535 1.206171
H -1.598753 3.878985 0.346614
H -1.409013 3.195619 1.972285
Br -1.826021 -1.365172 -0.842013

***trans*-44:**

E: -666.402631
ZPE: 0.232814
H: -666.153108
qh-G: -666.212201

-1 1
C -2.865124 1.086611 0.031675
C -3.222233 -0.263147 -0.593455
C -2.024224 -0.566746 -1.504267
C -0.787671 0.139154 -0.865561
C -1.388387 0.910229 0.37886
C -0.639899 2.195614 0.609315
C 0.578514 2.205087 1.459596
C 1.096518 1.143166 2.075909
C 0.38787 -0.729064 -0.571256
O -0.990027 3.238818 0.077003
H -1.300695 0.256438 1.250944
H -0.427767 0.898467 -1.563181
H -3.475003 1.342993 0.901289
H -2.970222 1.887392 -0.706066
H -4.173416 -0.249183 -1.12931
H -3.292643 -1.020859 0.193959
H -2.198516 -0.156508 -2.502286
H -1.849067 -1.637569 -1.613439
H 1.058244 3.177443 1.531324
H 2.008253 1.233899 2.656423
H 0.656282 0.154643 2.008695
C 1.718467 -0.338966 -0.614705
O 2.734305 -0.906135 -0.171137
O 1.875906 0.872945 -1.299111
C 3.151528 1.47218 -1.17002
H 3.104593 2.403976 -1.733203
H 3.378553 1.688563 -0.122149
H 3.939698 0.835164 -1.575435
Br 0.01566 -2.285274 0.518053

TS-45:

E: -666.386573
ZPE: 0.232660
H: -666.138312
qh-G: -666.195034

-1 1
C 3.012323 0.519521 -0.697791
C 2.324307 1.557078 -1.589313
C 0.927765 0.965077 -1.866176
C 0.657057 -0.074587 -0.73239
C 1.896282 0.084559 0.2402
C 2.10934 -1.217334 0.977261
C 0.983344 -1.62522 1.786226
C -0.033004 -0.741499 2.035356
C -0.705719 -0.047818 -0.096739
O 3.092618 -1.935401 0.751728
C -1.351825 1.132986 0.336242
H 1.66824 0.895655 0.936514
H 0.777718 -1.070806 -1.167504
H 3.8826 0.911702 -0.167015
H 3.342023 -0.34116 -1.288328
H 2.881535 1.773435 -2.502987
H 2.219214 2.494392 -1.03468
H 0.902014 0.461608 -2.835656
H 0.16146 1.739726 -1.877258
H 0.863721 -2.687181 1.975152
H -0.959218 -1.086122 2.485954
H 0.15945 0.318795 2.131855
O -2.539728 1.332122 0.591966
O -0.421243 2.122373 0.581164
C -0.938732 3.336552 1.110759
H -0.083914 4.001083 1.222489
H -1.668274 3.778565 0.430242
H -1.413343 3.172455 2.07961
Br -1.916378 -1.424308 -0.70883

TS-46:

E: -666.391433
ZPE: 0.232734
H: -666.143100
qh-G: -666.199823

-1 1
C 3.082863 -0.297011 0.55836
C 2.505112 -1.455455 1.375222
C 1.124963 -0.940787 1.81721
C 0.664551 0.067692 0.720927
C 1.892277 0.149865 -0.278282
C 1.942242 1.550208 -0.84469
C 0.739294 1.942366 -1.535538
C -0.209939 1.003317 -1.859295
C -0.652798 -0.178521 0.059874
O 2.865011 2.328885 -0.56207
H 1.71595 -0.582549 -1.074365
H 0.570113 1.05437 1.179691
H 3.944653 -0.579384 -0.05031
H 3.391994 0.521523 1.215896
H 3.135691 -1.750059 2.216551
H 2.382293 -2.329548 0.726902
H 1.209066 -0.418304 2.773357
H 0.404968 -1.749569 1.948009
H 0.50959 3.002762 -1.566105
H -1.185963 1.322224 -2.215605
H 0.065486 0.000025 -2.157095
C -1.862506 0.491554 0.385405
O -3.016158 0.171494 0.099056
O -1.621327 1.68059 1.032663
C -2.759478 2.509246 1.229976
H -2.394253 3.398548 1.740134
H -3.211385 2.786126 0.27563
H -3.509127 2.007292 1.843455
Br -0.904251 -1.976745 -0.605382

***trans*-47:**

E: -666.421150
ZPE: 0.235599
H: -666.170174
qh-G: -666.226245

-1 1
C -2.902328 0.677469 0.896054
C -2.053205 1.687688 1.687145
C -0.627911 1.07767 1.739969
C -0.643645 -0.028303 0.660431
C -1.930963 0.178872 -0.166703
C -2.241366 -1.133094 -0.901119
C -1.13626 -1.640453 -1.551294
C 0.123784 -0.816314 -1.626938
C 0.57955 -0.229927 -0.250648
O -3.408612 -1.642141 -0.803679
C 1.353492 1.070825 -0.457082
H -1.761425 0.982566 -0.895399
H -0.796452 -0.986107 1.16191
H -3.809001 1.119106 0.479032
H -3.203508 -0.161658 1.531634
H -2.454253 1.898986 2.680173
H -2.022481 2.633808 1.138412
H -0.416553 0.643626 2.71939
H 0.131554 1.84343 1.564177
H -1.183712 -2.586113 -2.08031
H 0.950258 -1.379736 -2.062723
H -0.014537 0.067037 -2.266231
O 2.512744 1.282572 -0.216886
O 0.537797 2.002184 -0.964018
C 1.119722 3.29329 -1.178419
H 0.320684 3.913004 -1.575195
H 1.488113 3.699237 -0.235875
H 1.940947 3.222504 -1.891235
Br 1.824278 -1.51077 0.642872

***cis*-48:**

E: -666.433436
ZPE: 0.235928
H: -666.182334
qh-G: -666.237823

-1 1
C 2.741503 -0.742613 0.851645
C 2.178983 -2.101165 1.275549
C 0.690824 -2.023939 0.895833
C 0.644634 -1.126831 -0.365814
C 2.068229 -0.496739 -0.506316
C 2.140163 0.950001 -1.011252
C 1.013411 1.484972 -1.597822
C -0.288037 0.727834 -1.725334
C -0.445235 -0.058988 -0.433048
O 3.266886 1.536098 -0.868065
C -0.513232 0.899206 0.75565
O -1.376812 1.894431 0.536522
O 0.105039 0.78674 1.783276

H 2.611773 -1.099573 -1.246001
H 0.456711 -1.750488 -1.243578
H 2.435135 0.030357 1.560083
H 3.829171 -0.727525 0.775899
H 2.670889 -2.894182 0.701532
H 2.327269 -2.320867 2.335098
H 0.124882 -1.568476 1.708299
H 0.256286 -3.006354 0.701434
H 1.05234 2.505322 -1.966475
H -0.33197 0.014653 -2.56038
H -1.122222 1.419067 -1.847346
Br -2.234514 -1.026111 -0.410027
C -1.483165 2.863485 1.582162
H -2.210244 3.592413 1.235033
H -1.824704 2.391387 2.503614
H -0.516702 3.338653 1.752353

TS-49:

E: -666.414185
ZPE: 0.234897
H: -666.163929
qh-G: -666.220286

-1 1
C 0.93904 -1.85295 0.978756
C 0.884224 -0.803496 -0.129477
C 2.139061 0.023829 0.177261
C 2.241028 1.241572 -0.717876
C 1.030249 1.642084 -1.341684
C -0.13442 0.756623 -1.735471
C -0.23614 0.162554 -0.393728
O 3.287884 1.924395 -0.713802
C -0.786479 0.9948 0.721189
O -1.640549 1.929751 0.294072
O -0.517578 0.825989 1.887761
H 1.050713 -1.32455 -1.080797
H 0.936494 2.697963 -1.575017
H 0.041124 0.009633 -2.512703
H -0.990344 1.372382 -1.998412
Br -2.176966 -1.370251 -0.495195
C -2.217062 2.750101 1.312112
H -2.867803 3.448757 0.793582
H -2.791726 2.138705 2.008446
H -1.43727 3.285447 1.854509
H 1.967677 0.45466 1.174982
C 3.219108 -1.042293 0.317106
H 4.089197 -0.694818 0.874506
H 3.559757 -1.365146 -0.670653
C 2.460216 -2.193849 1.045811
H 2.787052 -2.283313 2.082903
H 2.661821 -3.151787 0.563381
H 0.592304 -1.421214 1.918336
H 0.323811 -2.722873 0.750524

***trans*-50:**

E: -653.161536
ZPE: 0.235751
H: -652.912177
qh-G: -652.964055

-1 1
C -3.054358 0.429331 -0.294207
C -2.680082 1.923696 0.002605
C -1.130886 1.98958 0.18783
C -0.805677 0.541638 0.543129
C -1.684378 -0.199611 -0.468415
C -1.256473 -1.63784 -0.350319
C 0.227496 -1.595864 -0.028622
C 0.642304 -1.366521 1.391312
C 0.543586 -0.1782 0.463396
O -1.906861 -2.640053 -0.513632
C 1.78148 0.511146 0.027851
O 2.796713 -0.345015 -0.144684
O 1.884708 1.700063 -0.151705
H -1.221825 0.363042 1.542596
H -3.691561 0.325482 -1.171724
H -3.574173 -0.017719 0.556204
H -3.18424 2.260404 0.909811
H -3.001353 2.580705 -0.806183
H -0.637469 2.267999 -0.744841
H -0.834968 2.706113 0.95392
H 0.907942 -2.141299 -0.669931
H -0.097984 -1.42508 2.179897
H 1.632457 -1.718563 1.647168
C 4.040647 0.233487 -0.556382
H 4.737827 -0.595451 -0.637393
H 4.385825 0.952713 0.186012
H 3.923375 0.729962 -1.519388
H -1.282298 0.086685 -1.455045

***cis*-51:**

E: -653.188513
ZPE: 0.236281
H: -652.938863

qh-G: -652.990091

-1 1
C 3.036918 0.490429 -0.14773
C 2.480547 1.917857 -0.293871
C 0.964625 1.720957 -0.361392
C 0.703549 0.659167 0.717666
C 1.91003 -0.30116 0.553586
C 1.36288 -1.454717 -0.287911
C -0.11618 -1.520956 -0.135642
C -0.609802 -1.472626 1.287727
C -0.535258 -0.199704 0.498099
O 2.0211 -2.166742 -1.007798
C -1.81566 0.456057 0.125265
O -2.727424 -0.418247 -0.312542
O -2.025957 1.639897 0.225698
H 2.242992 -0.723347 1.505924
H 0.686401 1.125748 1.706293
H 3.236358 0.046965 -1.126328
H 3.96796 0.461816 0.417603
H 2.729868 2.508824 0.592566
H 2.883165 2.438106 -1.164093
H 0.68641 1.315546 -1.342903
H 0.393273 2.633926 -0.201554
H -0.713761 -2.018695 -0.885791
H 0.099349 -1.603731 2.095418
H -1.592257 -1.89493 1.447576
C -4.0115 0.126388 -0.641521
H -4.613461 -0.71712 -0.966783
H -4.454808 0.597958 0.235206
H -3.914131 0.859523 -1.441663

TS-52:

E: -666.426635
ZPE: 0.234931
H: -666.176636
qh-G: -666.232152

-1 1
C 2.872486 -0.837197 0.778563
C 2.237142 -2.161671 1.198519
C 0.74645 -1.941057 0.906416
C 0.726646 -1.073468 -0.373513
C 2.177304 -0.520674 -0.554976
C 2.209675 0.964959 -0.905683
C 1.031678 1.531705 -1.412383
C -0.195817 0.738006 -1.790127
C -0.270178 0.068453 -0.472437
O 3.26399 1.606131 -0.672154
C -0.530502 0.986033 0.694247
O -1.369936 1.977637 0.371231
O -0.074046 0.851887 1.804986
H 2.684645 -1.076499 -1.35236
H 0.485614 -1.710014 -1.227865
H 2.619715 -0.058706 1.505246
H 3.958066 -0.873027 0.681993
H 2.630248 -2.973261 0.576409
H 2.426919 -2.419783 2.242399
H 0.281718 -1.409184 1.734607
H 0.197843 -2.871275 0.755269
H 0.972423 2.614089 -1.456455
H -0.091717 0.017312 -2.605346
H -1.0331 1.401414 -1.987484
Br -2.306017 -1.132136 -0.364472
C -1.64294 2.917083 1.411574
H -2.324601 3.645162 0.980274
H -2.107915 2.417732 2.261918
H -0.720986 3.402522 1.734229

***trans*-53:**

E: -666.424751
ZPE: 0.235704
H: -666.173585
qh-G: -666.229998

-1 1
C -1.271332 -1.741945 0.912163
C -0.922724 -0.317964 0.481947
C -2.135786 0.091523 -0.370872
C -2.054169 1.592427 -0.687508
C -0.81908 1.943355 -1.199542
C 0.179324 0.851441 -1.486367
C 0.411585 0.04035 -0.201162
O -3.045139 2.348207 -0.426944
C 1.359383 0.722788 0.787285
O 2.302454 1.444103 0.18456
O 1.289546 0.60206 1.985092
H -0.964379 0.301299 1.384478
H -0.581462 2.973138 -1.443693
H -0.171813 0.138193 -2.245165
H 1.128662 1.248351 -1.842848
Br 1.488611 -1.613604 -0.700304
C 3.25786 2.06597 1.049937
H 3.93219 2.613921 0.398033

H 2.755566 2.744587 1.739023
H 3.800948 1.307899 1.61461
H -2.08645 -0.473575 -1.313392
C -3.317088 -0.412147 0.460324
H -4.170065 -0.684498 -0.163258
H -3.649689 0.388634 1.123773
C -2.769158 -1.622539 1.272998
H -3.304507 -2.546573 1.048299
H -2.883678 -1.439965 2.343949
H -1.136491 -2.425246 0.070088
H -0.657991 -2.104561 1.73984

***cis*-54:**

E: -666.434431
ZPE: 0.235743
H: -666.183381
qh-G: -666.239319

-1 1
C -2.677955 1.365885 -1.082172
C -2.214669 2.375147 -0.027398
C -0.690145 2.154442 0.058853
C -0.435202 0.735914 -0.541045
C -1.829212 0.123503 -0.774262
C -2.41861 -0.657569 0.411889
C -1.631183 -0.906399 1.505698
C -0.204212 -0.462965 1.672611
C 0.463158 -0.106696 0.354015
O -3.63627 -1.040498 0.257646
C 1.838879 0.514677 0.577117
O 2.271893 1.189499 -0.487825
O 2.471386 0.412564 1.597527
H -1.815647 -0.561258 -1.628744
H 0.058641 0.857555 -1.506535
H -2.440529 1.738367 -2.0852
H -3.743833 1.143215 -1.035725
H -2.681294 2.132611 0.930668
H -2.472389 3.406932 -0.27455
H -0.14721 2.895927 -0.530774
H -0.329537 2.243581 1.086928
H -2.06506 -1.475516 2.32339
H 0.398498 -1.22104 2.18047
H -0.109287 0.437799 2.300208
Br 0.918019 -1.806263 -0.636964
C 3.57835 1.764585 -0.375905
H 3.760776 2.26566 -1.322186
H 3.606372 2.477412 0.447929
H 4.318552 0.982288 -0.20732

TS-55:

E: -666.419347
ZPE: 0.234514
H: -666.169454
qh-G: -666.225888

-1 1
C -1.346009 -1.87045 0.609549
C -1.007424 -0.391523 0.409668
C -2.222085 0.10172 -0.387712
C -2.015842 1.59388 -0.619803
C -0.748341 1.846619 -1.17461
C 0.121624 0.691576 -1.610023
C 0.274864 0.128635 -0.24041
O -2.869306 2.439568 -0.263951
C 1.179635 0.906554 0.687117
O 2.109837 1.609005 0.036572
O 1.101243 0.874843 1.891758
H -1.065757 0.085066 1.394366
H -0.366852 2.858442 -1.244049
H -0.334067 -0.02467 -2.298628
H 1.070701 1.02678 -2.01794
Br 1.72985 -1.695246 -0.429508
C 3.028714 2.329267 0.860907
H 3.70038 2.839925 0.176352
H 2.495927 3.049594 1.482045
H 3.583632 1.639324 1.497229
H -2.217207 -0.405226 -1.362539
C -3.385687 -0.436983 0.432217
H -4.307582 -0.523007 -0.144353
H -3.583133 0.236322 1.269738
C -2.86489 -1.818036 0.936811
H -3.391456 -2.642887 0.453757
H -3.029947 -1.919649 2.011165
H -1.163712 -2.418229 -0.317773
H -0.757238 -2.343059 1.396102

***trans*-56:**

E: -653.169630
ZPE: 0.236507
H: -652.919669
qh-G: -652.971242

-1 1
C -2.255687 2.227197 0.154004

C -0.836229 1.921153 0.737003
C -0.499654 0.609128 0.04187
C -1.836784 -0.12868 0.10669
C -1.479818 -1.495356 -0.428434
C -0.089971 -1.792419 0.071766
C 0.224498 -1.425017 1.499265
C 0.539994 -0.428751 0.430129
O -2.118383 -2.203405 -1.168738
C 1.9768 -0.245497 0.091993
O 2.210646 0.956228 -0.436923
O 2.835101 -1.07731 0.265996
H -0.316299 0.833526 -1.017726
H -0.121927 2.718701 0.533178
H -0.885784 1.771877 1.819585
H 0.491732 -2.569429 -0.406212
H 1.080617 -1.93799 1.91672
H -0.579832 -1.218524 2.191347
C 3.569655 1.223746 -0.807471
H 3.56769 2.225987 -1.225754
H 4.213134 1.175886 0.070634
H 3.90632 0.498834 -1.54794
H -2.088295 -0.27948 1.165396
C -2.808891 0.893888 -0.459001
H -2.919931 2.600564 0.93412
H -2.195813 2.999548 -0.613896
H -3.845234 0.708082 -0.179532
H -2.748765 0.911968 -1.549481

***cis*-57:**

E: -653.184752
ZPE: 0.236471
H: -652.935016
qh-G: -652.986066

-1 1
C 2.812054 -0.940891 -0.491049
C 2.348212 -1.573687 0.826873
C 0.868773 -1.919925 0.575331
C 0.335703 -0.83131 -0.388802
C 1.595818 -0.129867 -0.955375
C 1.552042 1.315859 -0.467208
C 0.233872 1.595235 0.185275
C 0.026081 0.909745 1.504487
C -0.51144 0.274675 0.252373
O 2.43559 2.123792 -0.619999
C -1.991275 0.316857 0.106343
O -2.483724 -0.790071 -0.450886
O -2.680007 1.243705 0.462738
H 1.553963 -0.050613 -2.046129
H -0.249415 -1.283081 -1.187247
H 3.025013 -1.722699 -1.224344
H 3.704619 -0.320468 -0.390999
H 2.450964 -0.84895 1.639605
H 2.938384 -2.448815 1.102634
H 0.806284 -2.894868 0.086045
H 0.281258 -1.975868 1.494274
H -0.267646 2.537132 0.007275
H -0.696601 1.38003 2.157916
H 0.860912 0.429296 1.996416
C -3.907757 -0.828728 -0.613985
H -4.123406 -1.786356 -1.078761
H -4.397869 -0.752939 0.356257
H -4.233821 -0.008983 -1.25335

TS-58:

E: -666.428814
ZPE: 0.234978
H: -666.178821
qh-G: -666.234308

-1 1
C 3.277598 -0.70074 -0.627049
C 2.761786 -1.75959 0.350143
C 1.334756 -2.026159 -0.148151
C 0.815951 -0.667565 -0.692538
C 2.076401 0.244834 -0.771283
C 2.071173 1.347027 0.290064
C 1.087182 1.320956 1.289174
C 0.160204 0.165065 1.591996
C -0.223388 0.00234 0.180266
O 2.908337 2.274319 0.153002
C -1.008847 1.111496 -0.459774
O -1.791522 1.763297 0.404306
O -0.961 1.369492 -1.639384
H 2.091709 0.775903 -1.725682
H 0.388996 -0.785985 -1.686608
H 3.506958 -1.16836 -1.590084
H 4.169128 -0.17215 -0.28459
H 3.3745 -2.662619 0.377176
H 2.737765 -1.339502 1.361677
H 0.680487 -2.446876 0.614851
H 1.363633 -2.740757 -0.974736
H 0.917034 2.245575 1.83106
H 0.604892 -0.735596 2.018901

H -0.660383 0.501055 2.220169
Br -2.028068 -1.580422 0.061429
C -2.55339 2.84015 -0.145044
H -3.112347 3.260262 0.686667
H -1.890031 3.590519 -0.57623
H -3.233033 2.46957 -0.912827

NMR Spectra

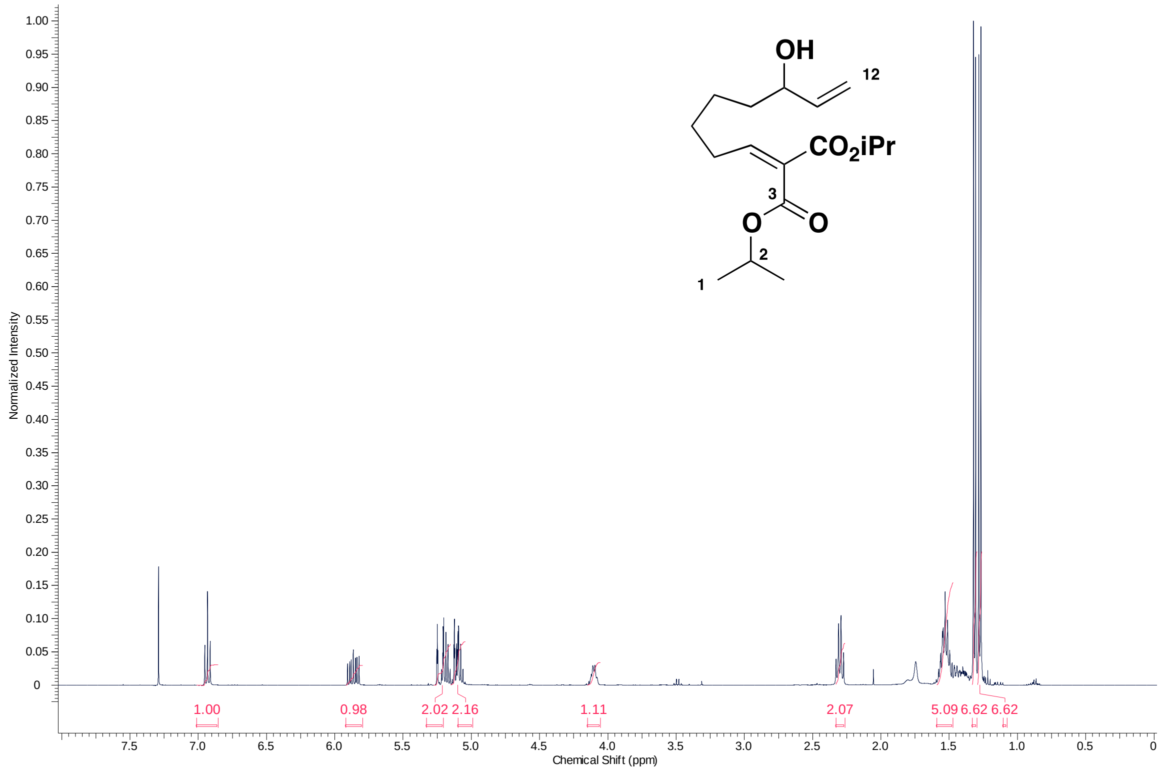

OH
12
CO2iPr
3
O
O
2
1
Normalized Intensity
1.00
0.98
2.02
2.16
1.11
2.07
5.09
6.62
6.62
Chemical Shift (ppm)

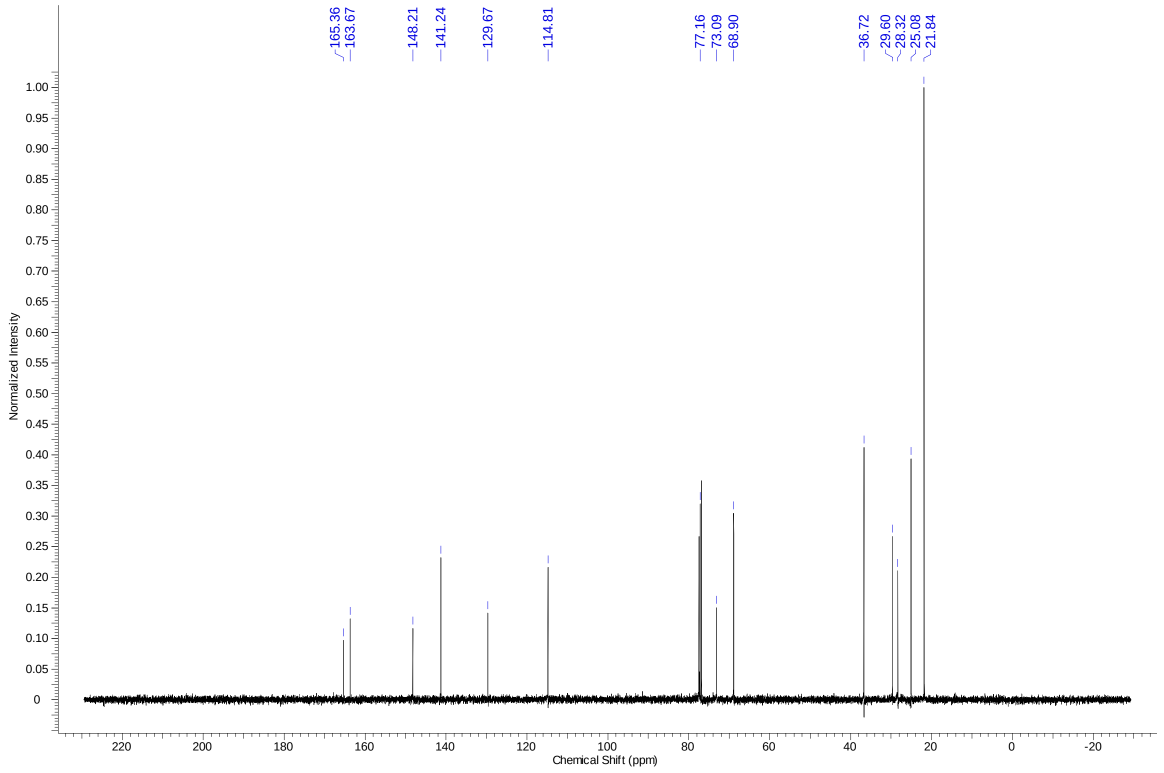

165.36
163.67
148.21
141.24
129.67
114.81
77.16
73.09
68.90
36.72
29.60
28.32
25.08
21.84
Normalized Intensity
Chemical Shift (ppm)

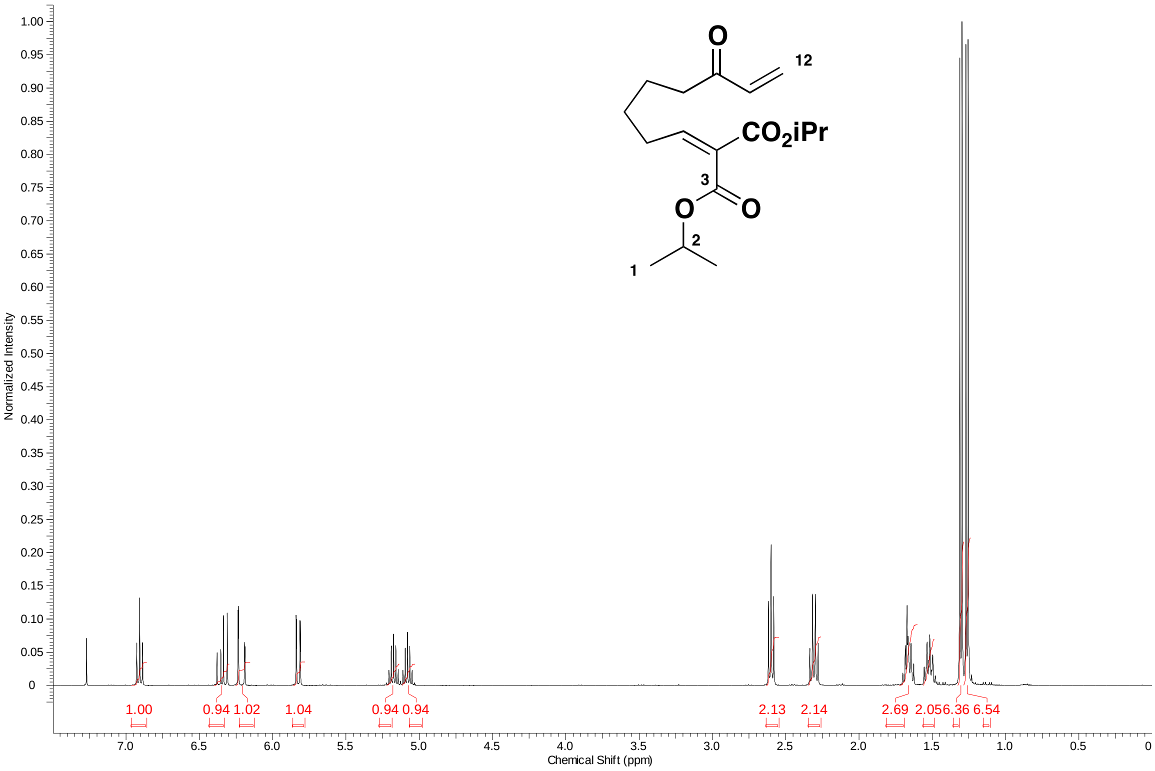
O
12
CO2iPr
3
O
O
2
1
Normalized Intensity
Chemical Shift (ppm)
1.00
0.94
1.02
1.04
0.94
0.94
2.13
2.14
2.69
2.05
6.36
6.54

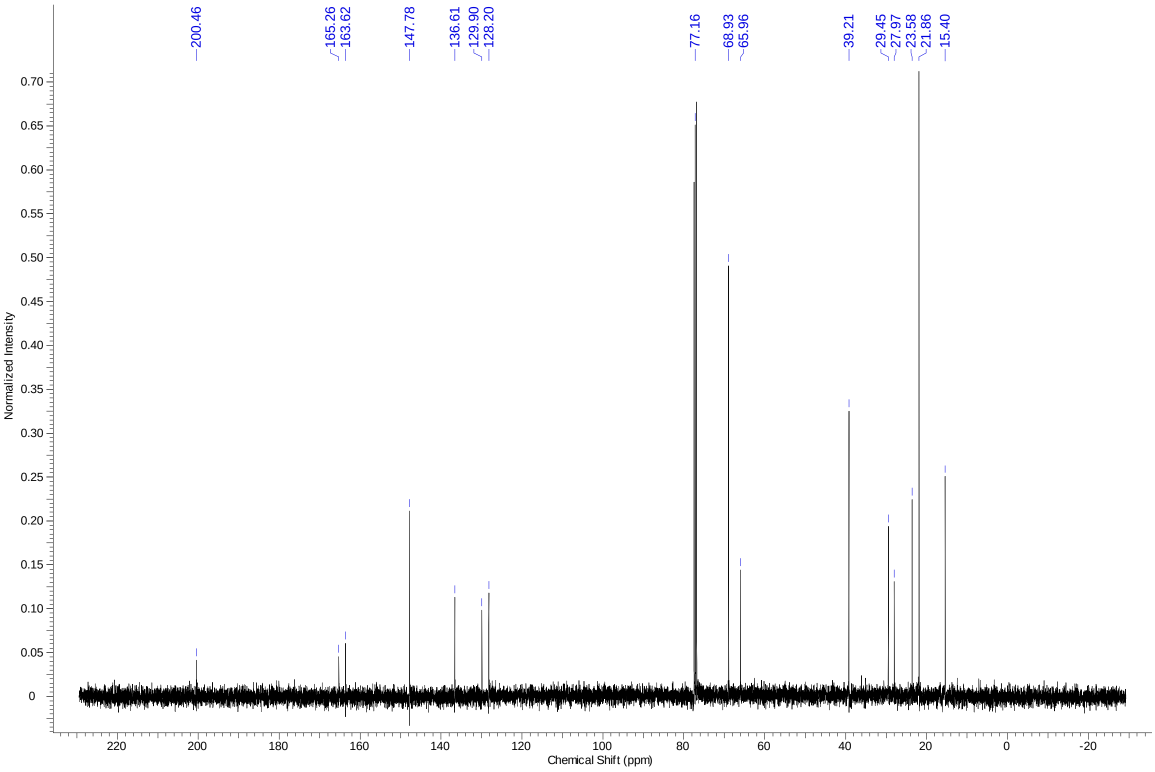
200.46
165.26
163.62
147.78
136.61
129.90
128.20
77.16
68.93
65.96
39.21
29.45
27.97
23.58
21.86
15.40
Normalized Intensity
Chemical Shift (ppm)

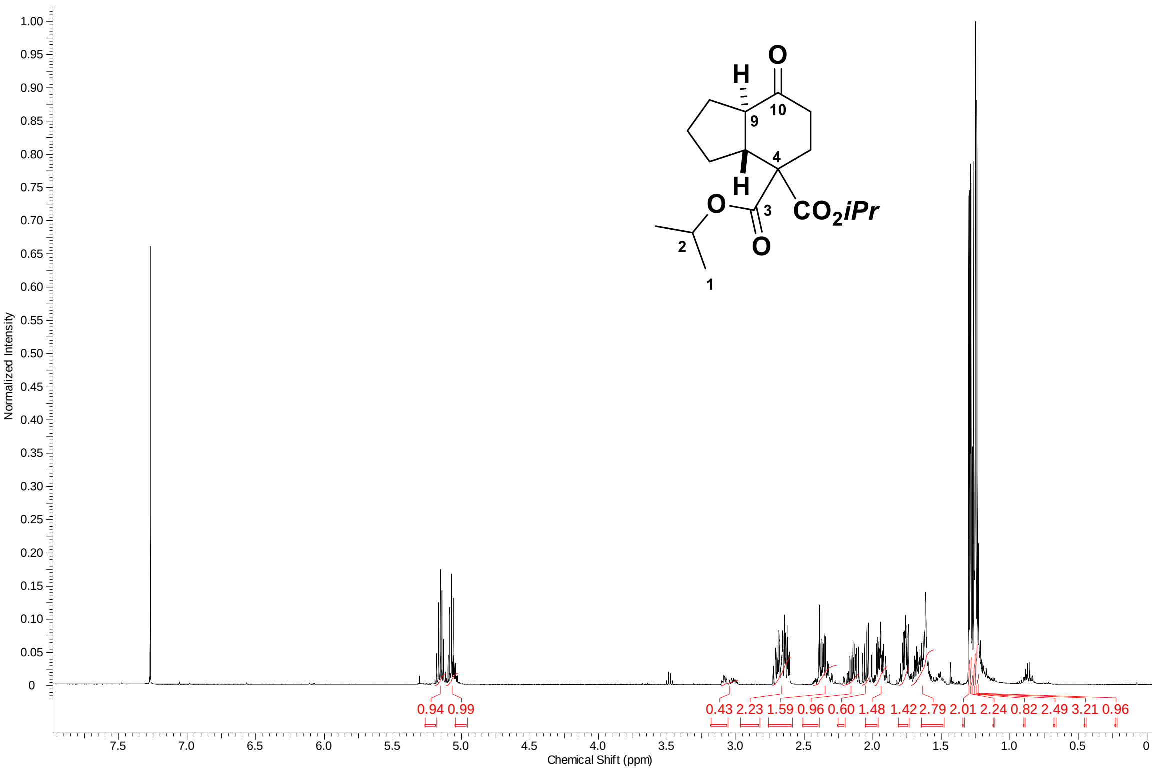
H
O
10
9
4
H
O
3
CO2iPr
O
2
1
Normalized Intensity
0.94 0.99
0.43 2.23 1.59 0.96 0.60 1.48 1.42 2.79 2.01 2.24 0.82 2.49 3.21 0.96
Chemical Shift (ppm)

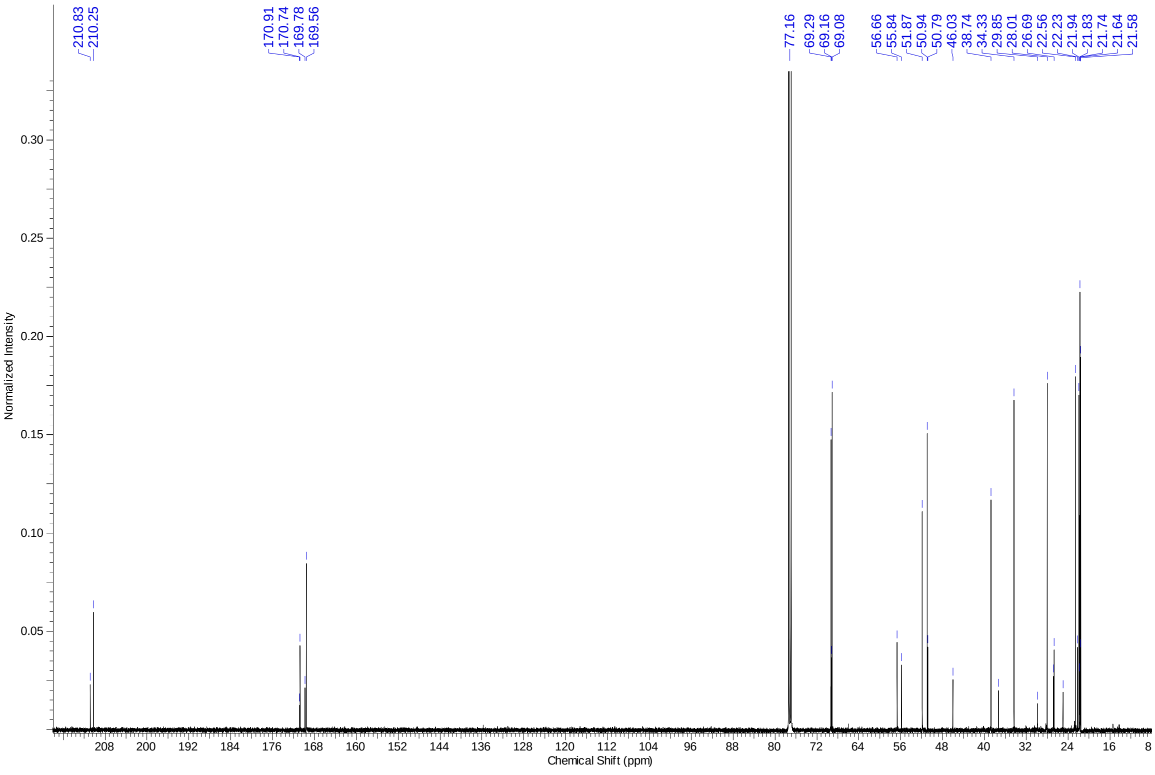
210.83
210.25
170.91
170.74
169.78
169.56
77.16
69.29
69.16
69.08
56.66
55.84
51.87
50.94
50.79
46.03
38.74
34.33
29.85
28.01
26.69
22.56
22.23
21.94
21.83
21.74
21.64
21.58
Normalized Intensity
Chemical Shift (ppm)

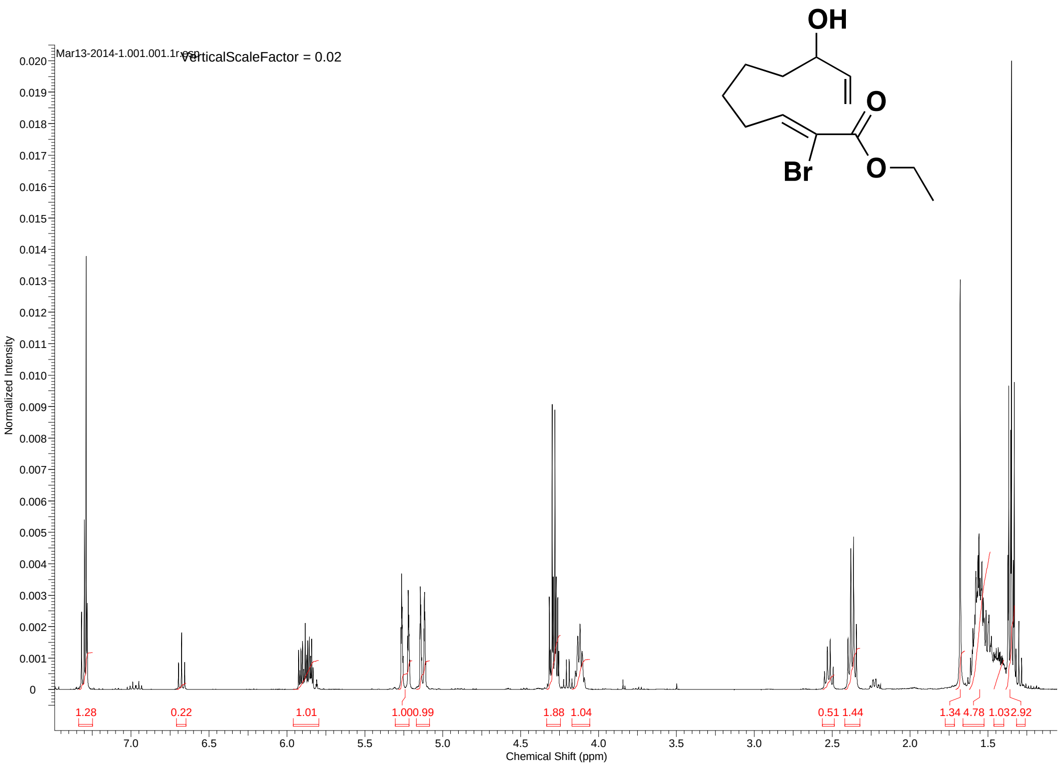
Mar13-2014-1.001.001.1r.esp
VerticalScaleFactor = 0.02
OH
O
Br
O
Normalized Intensity
1.28
0.22
1.01
1.00
0.99
1.88
1.04
0.51
1.44
1.34
4.78
1.03
2.92
Chemical Shift (ppm)

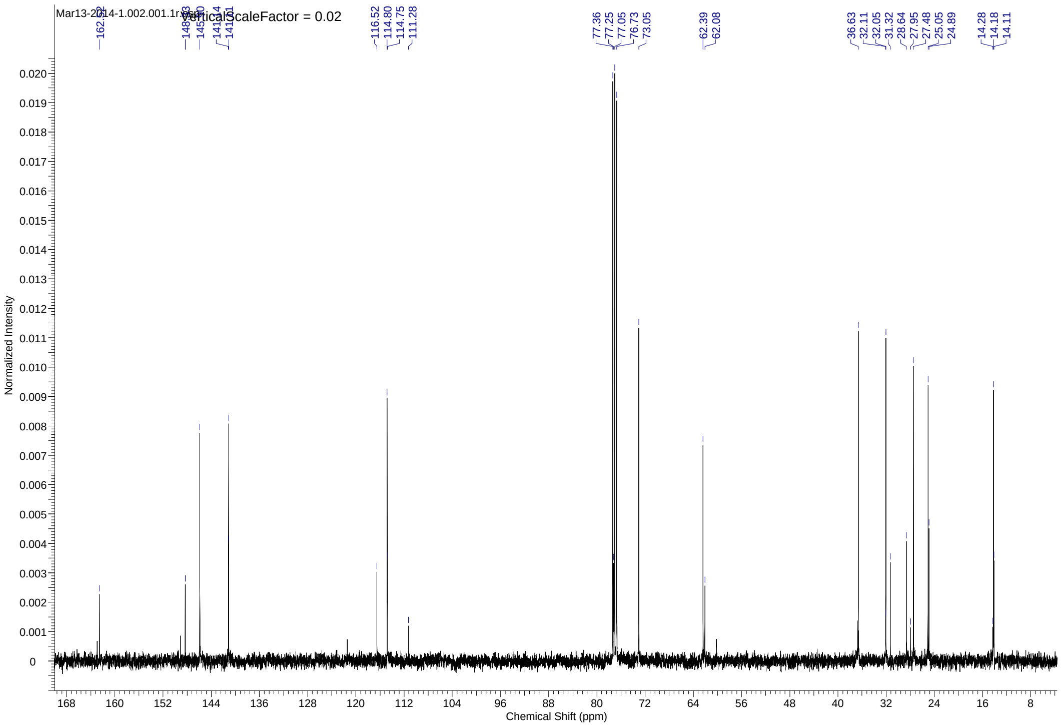
VerticalScaleFactor = 0.02
116.52
114.80
114.75
111.28
77.36
77.25
77.05
76.73
73.05
62.39
62.08
36.63
32.11
32.05
31.32
28.64
27.95
27.48
25.05
24.89
14.28
14.18
14.11
Normalized Intensity
Chemical Shift (ppm)

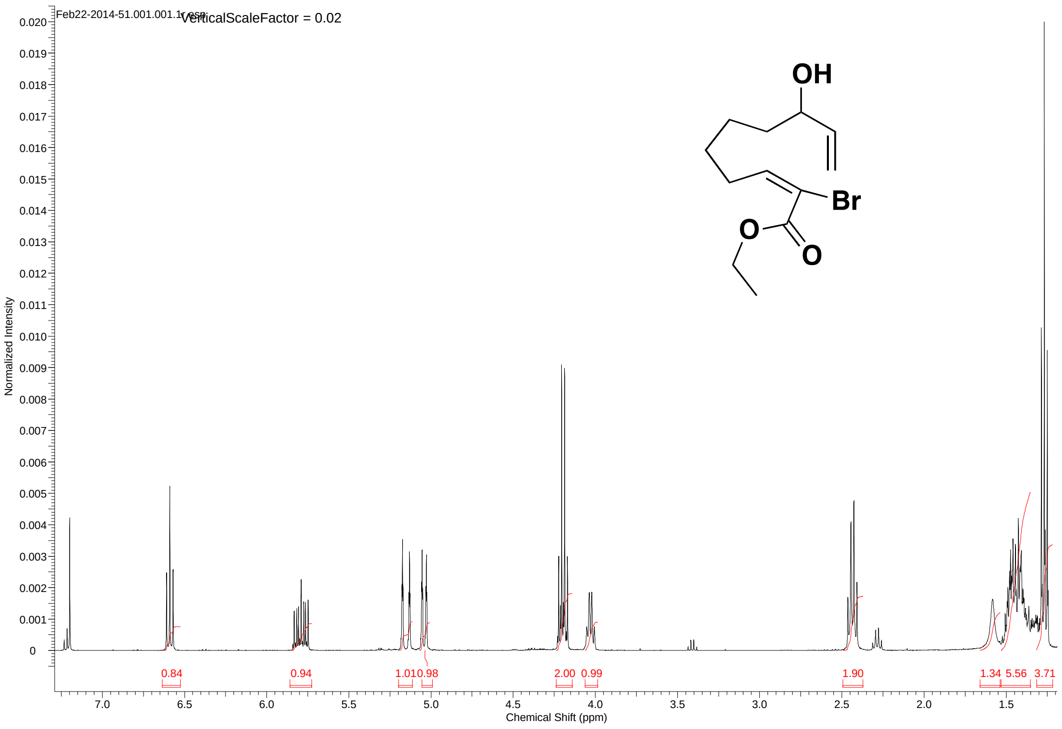
Feb22-2014-51.001.001.1r.esp
VerticalScaleFactor = 0.02
OH
Br
O
O
Normalized Intensity
Chemical Shift (ppm)
0.84
0.94
1.01
0.98
2.00
0.99
1.90
1.34
5.56
3.71

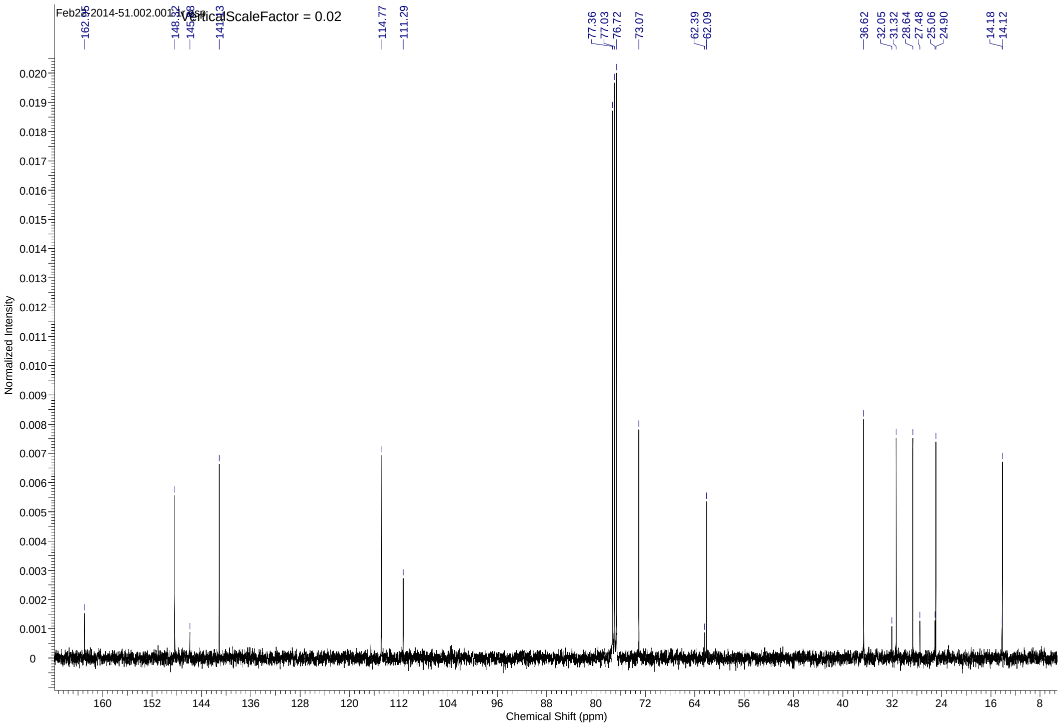
VerticalScaleFactor = 0.02
162.95
114.77
111.29
77.36
77.03
76.72
73.07
62.39
62.09
36.62
32.05
31.32
28.64
27.48
25.06
24.90
14.18
14.12
Normalized Intensity
Chemical Shift (ppm)

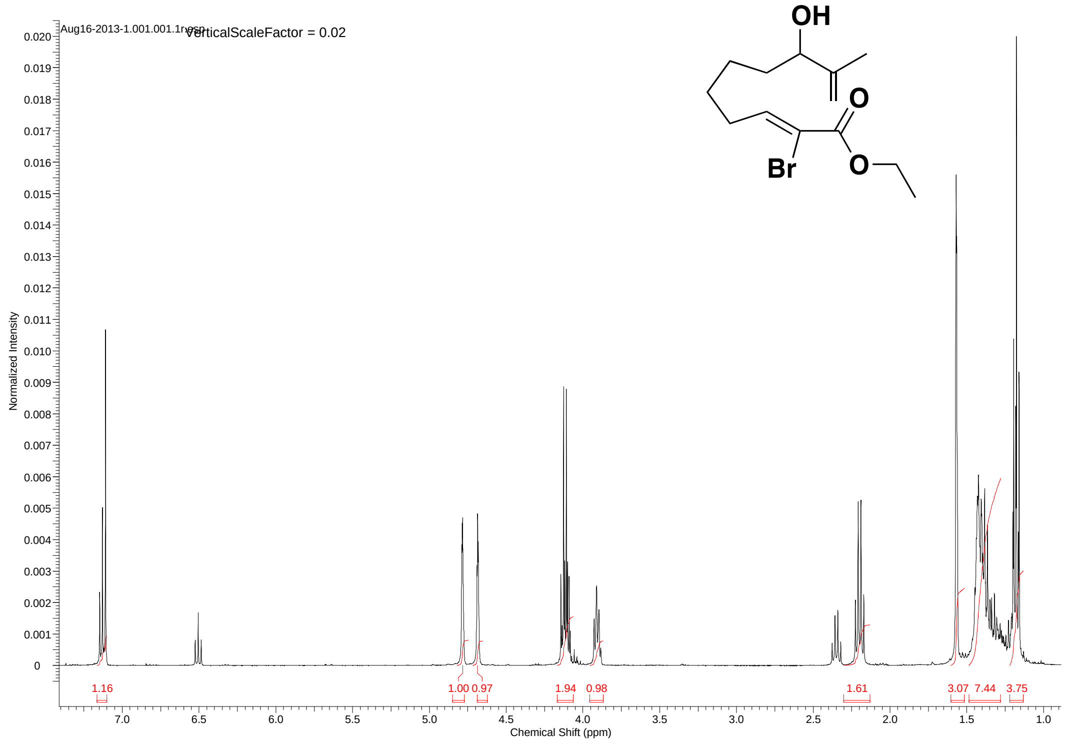

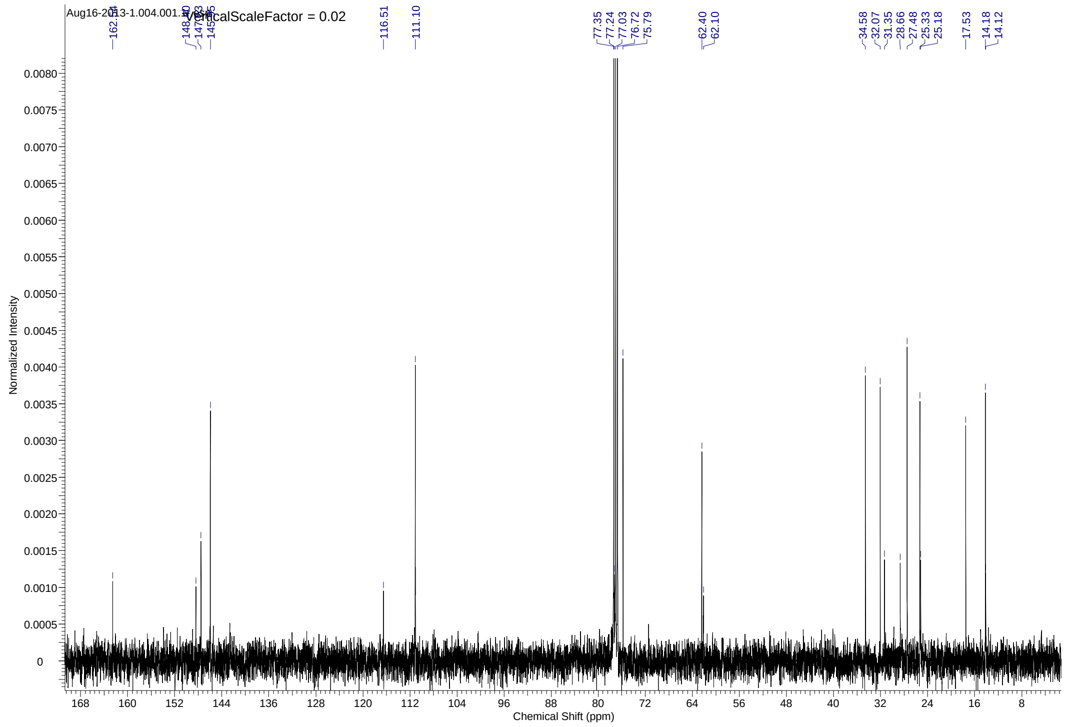

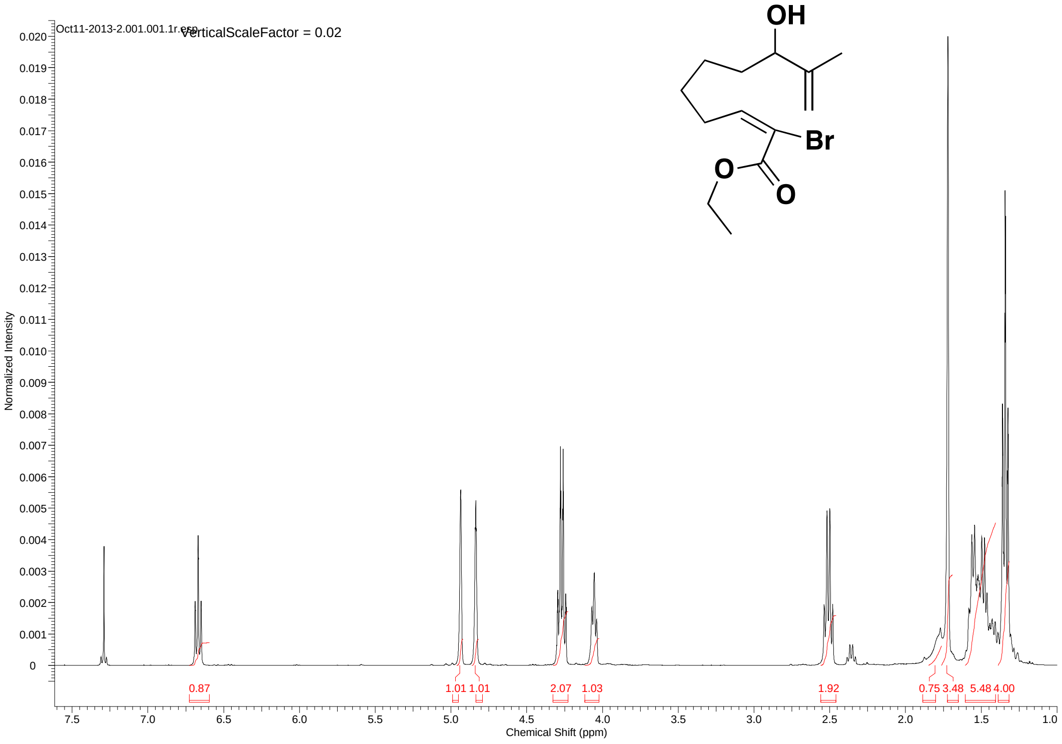
Oct11-2013-2.001.001.1r.esp
VerticalScaleFactor = 0.02
Normalized Intensity
Chemical Shift (ppm)
0.87
1.01 1.01
2.07 1.03
1.92
0.75 3.48 5.48 4.00
OH
Br
O
O

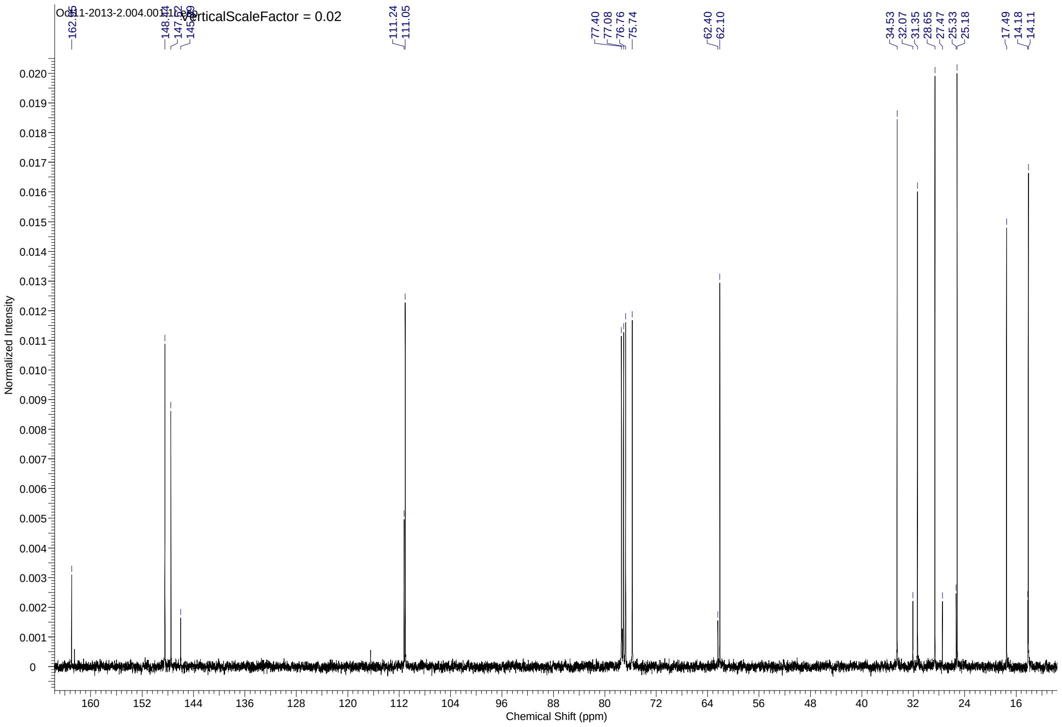
Oct11-2013-2.004.001.1r.esp
VerticalScaleFactor = 0.02
148.44
147.52
145.89
111.24
111.05
77.40
77.08
76.76
75.74
62.40
62.10
34.53
32.07
31.35
28.65
27.47
25.33
25.18
17.49
14.18
14.11
Normalized Intensity
Chemical Shift (ppm)

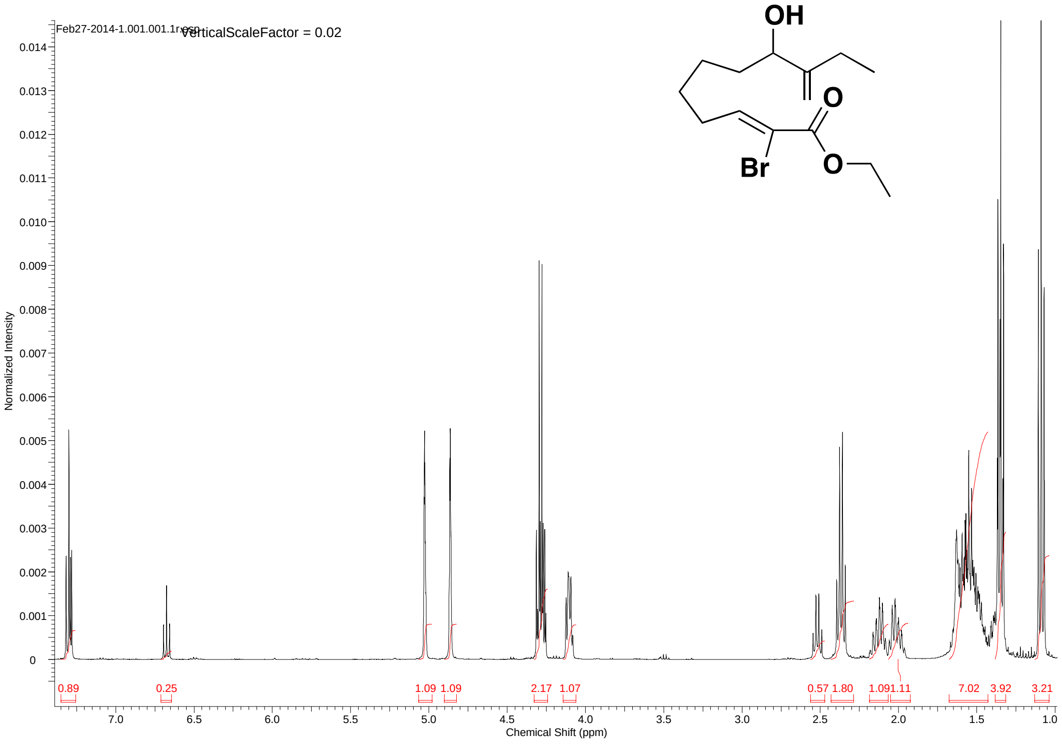

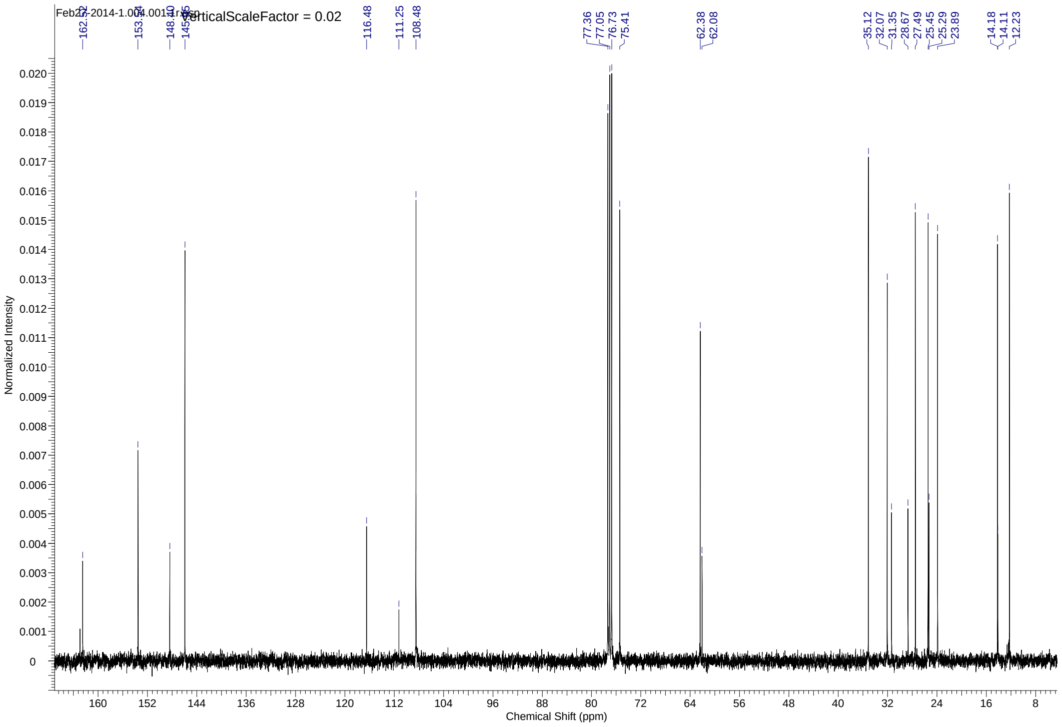

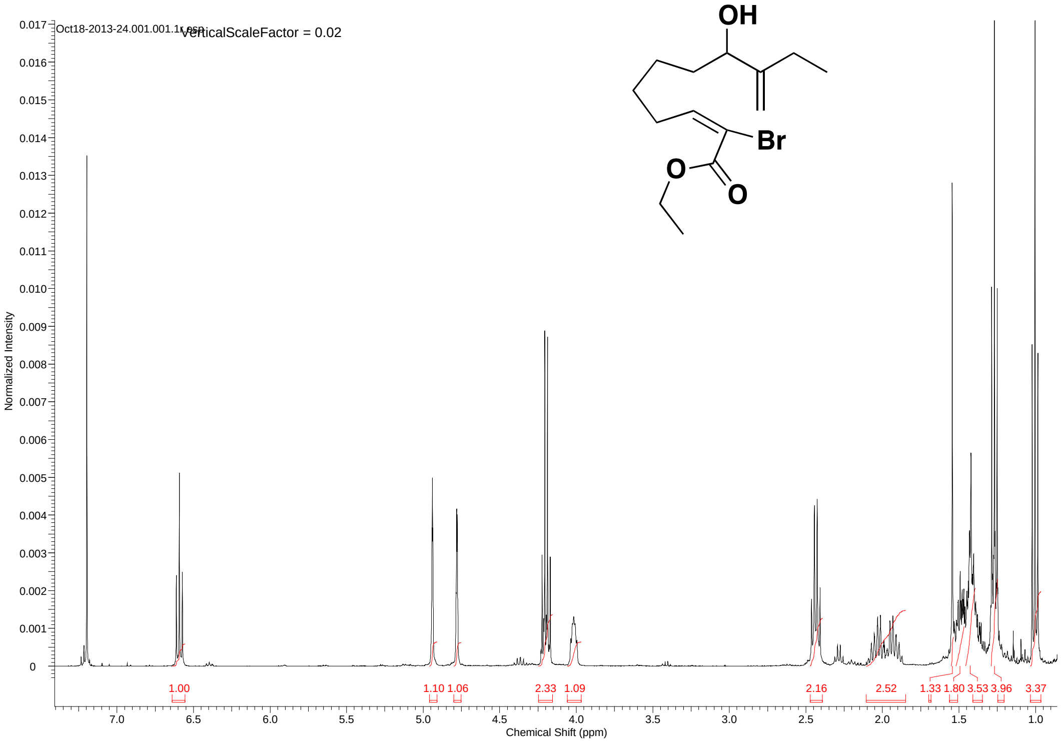

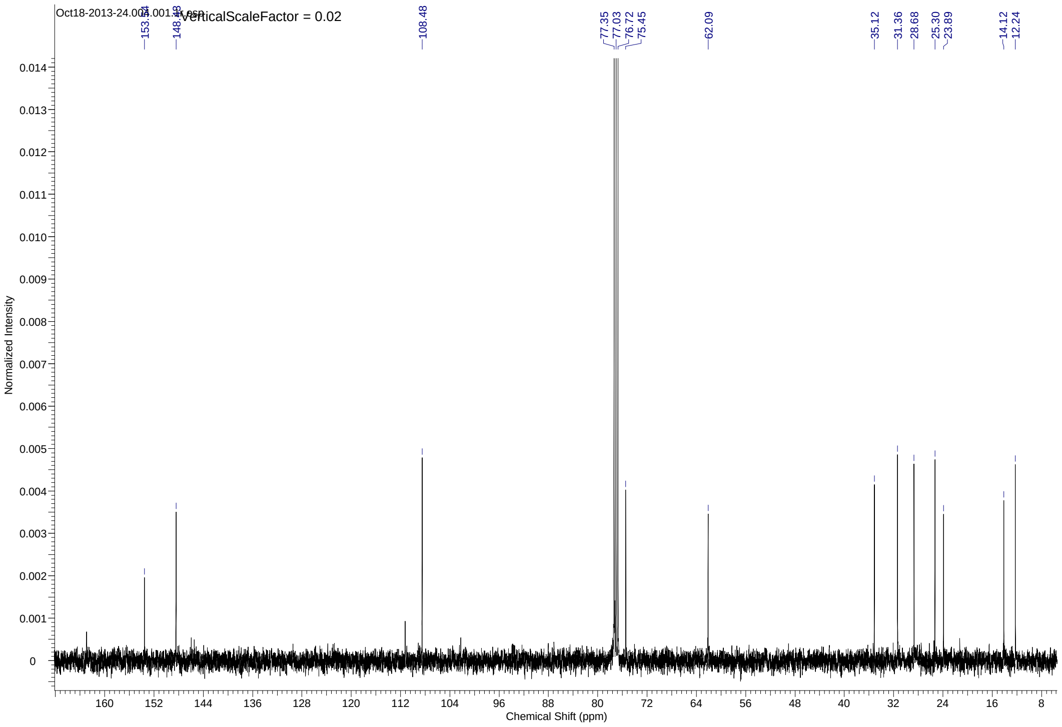

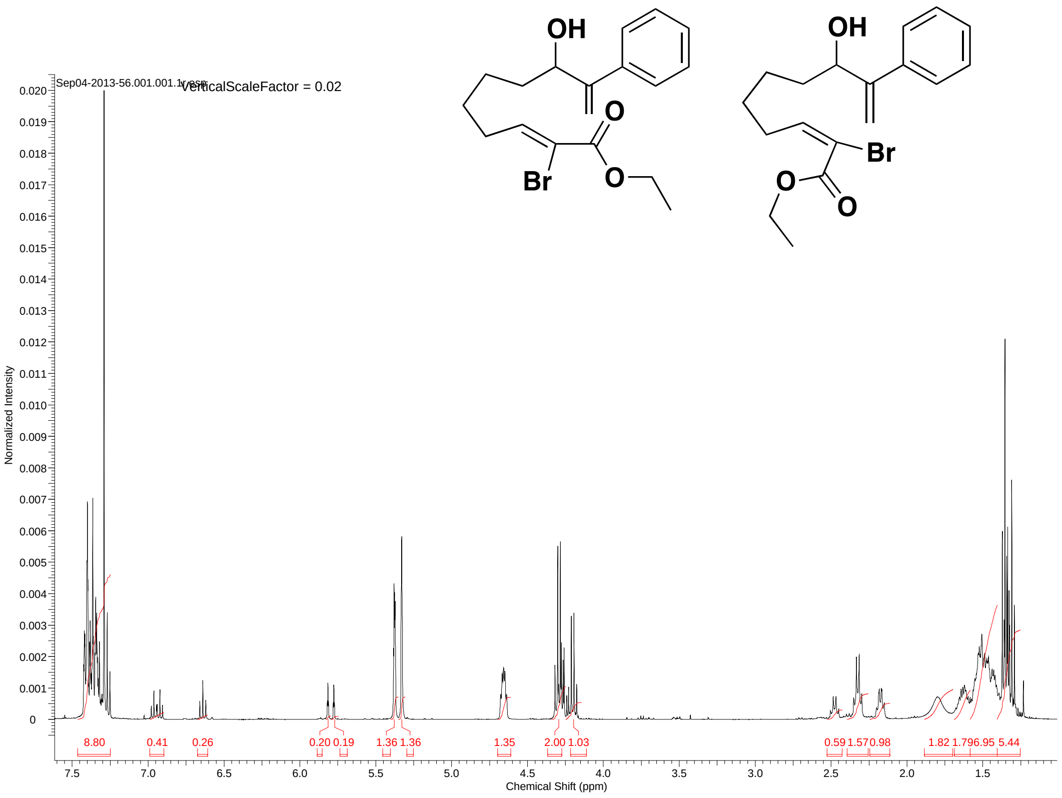

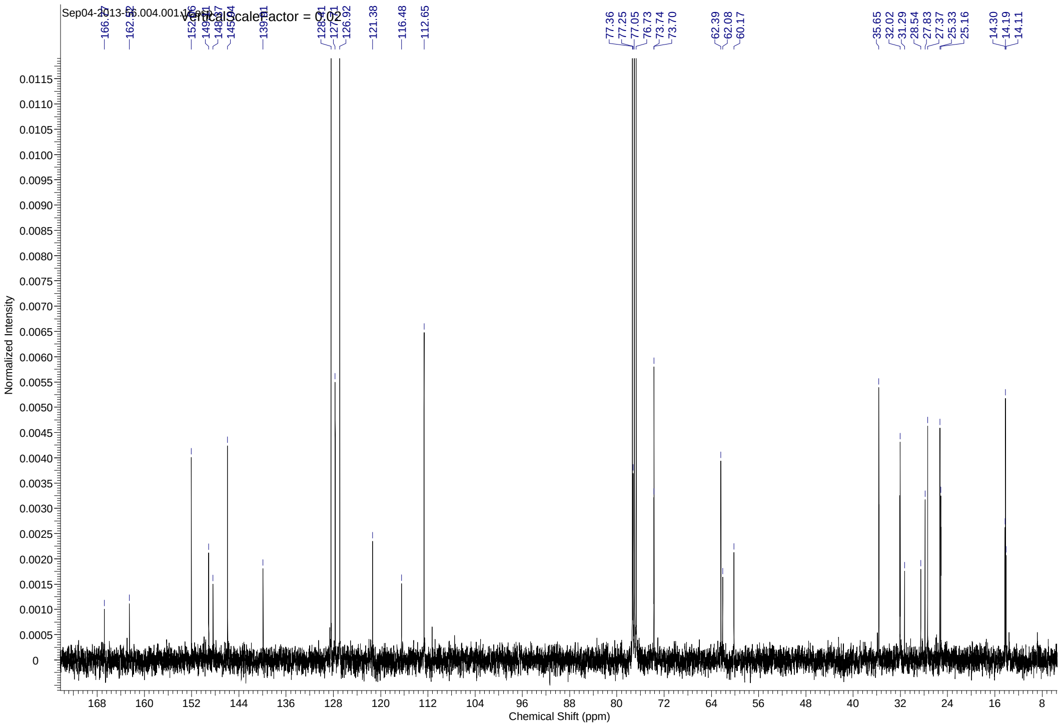

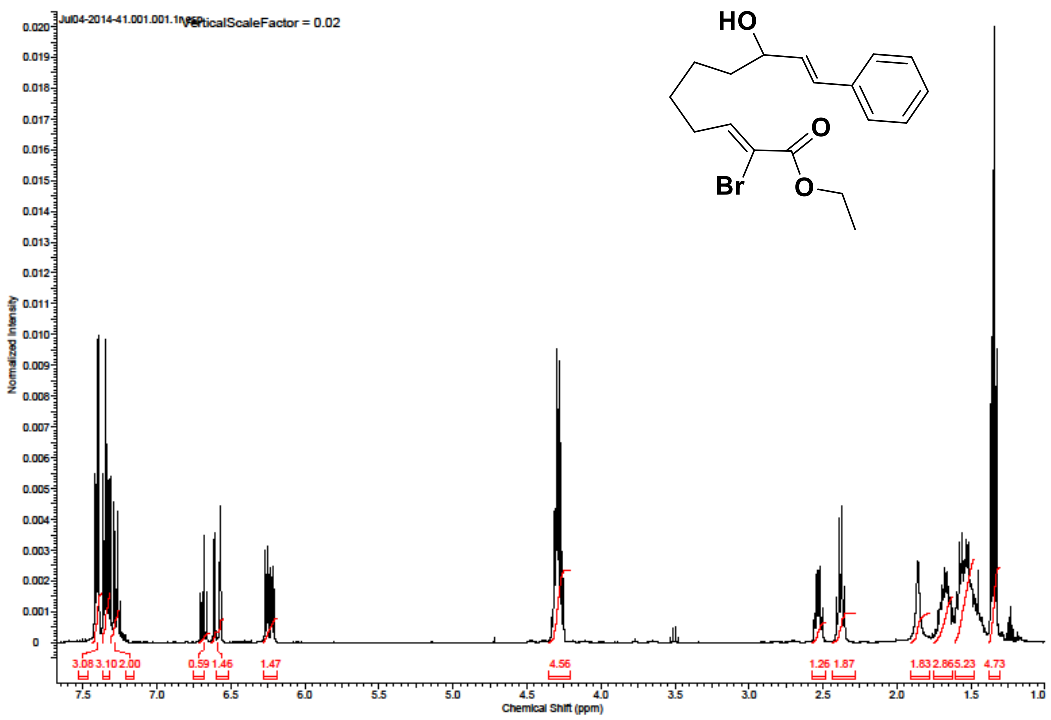

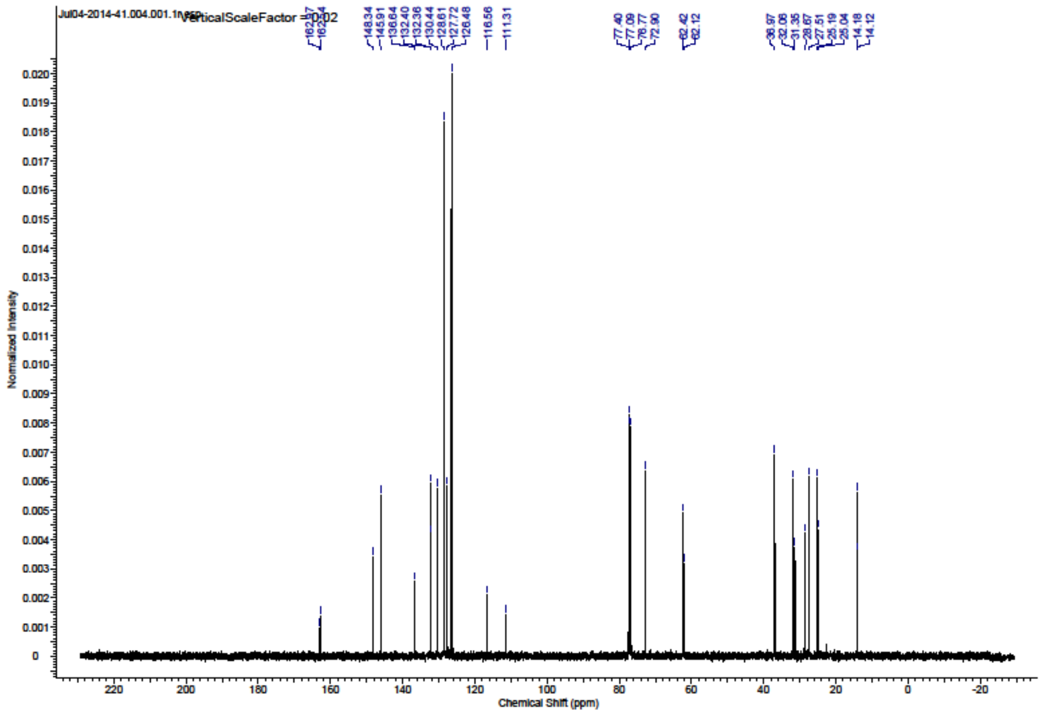

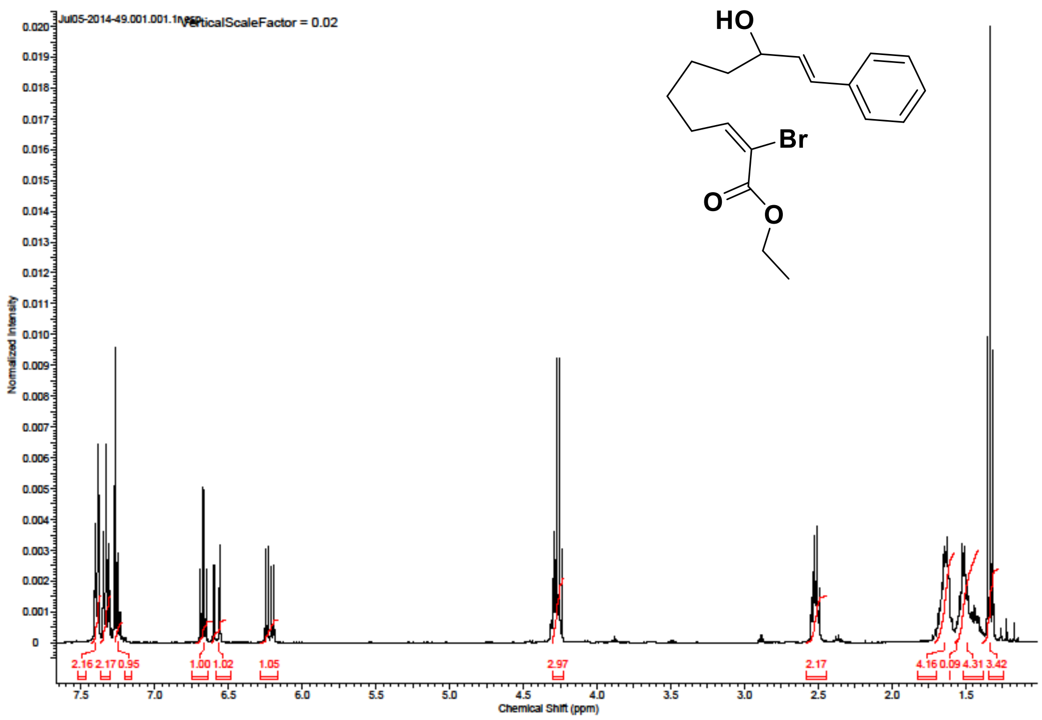

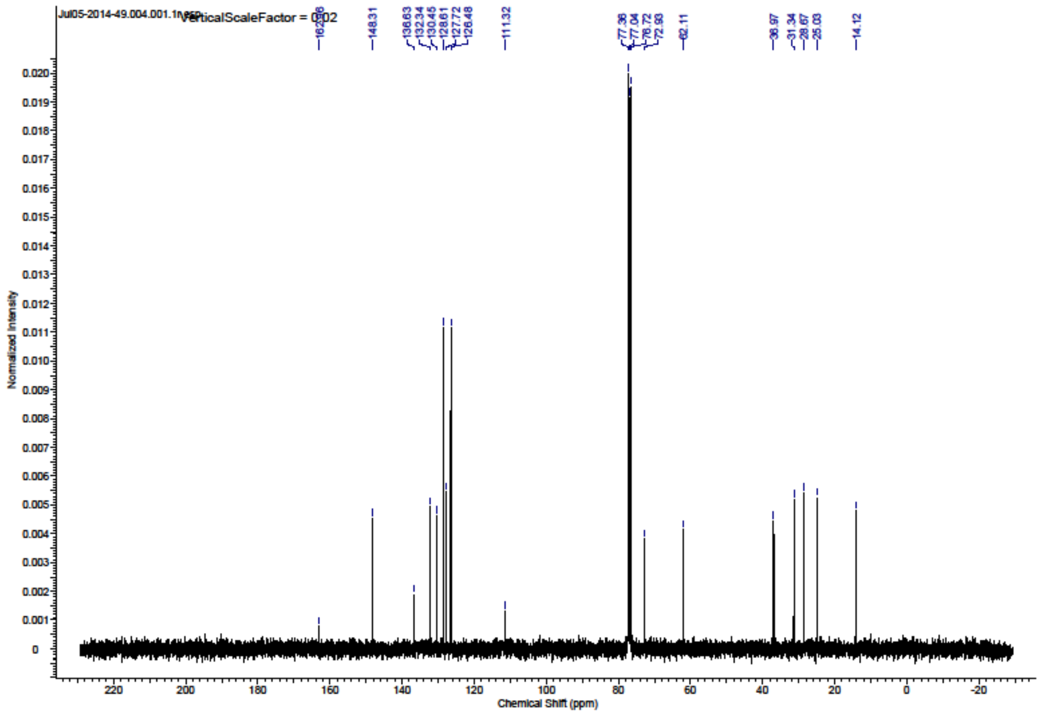

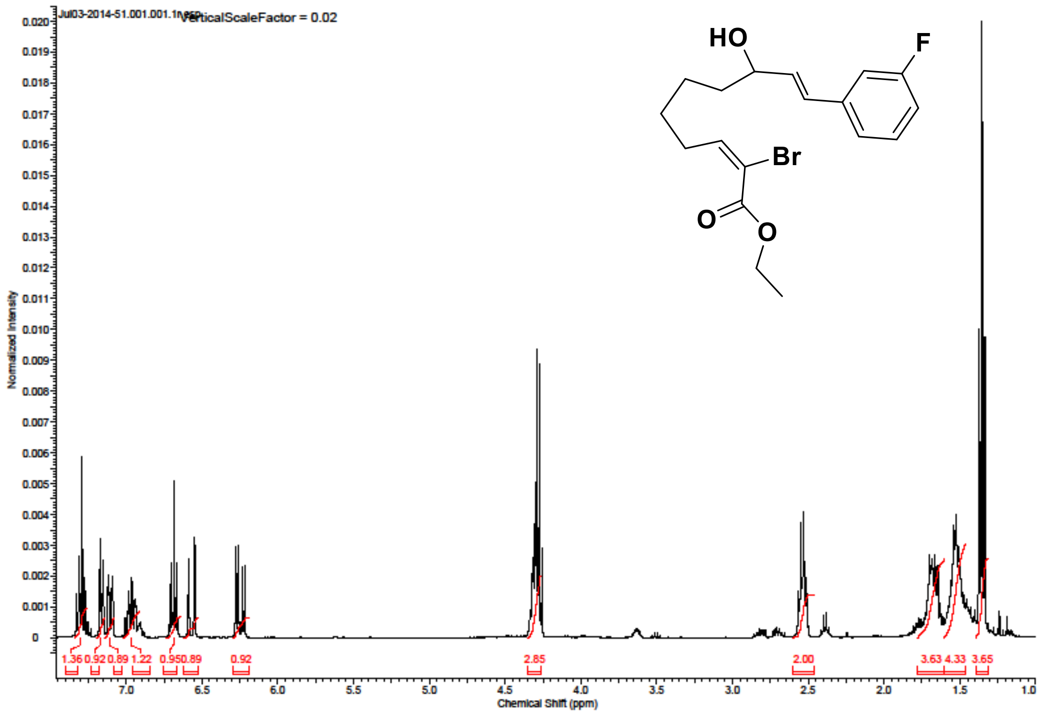

Jul03-2014-51.001.001.1r.esp
VerticalScaleFactor = 0.02
HO
F
Br
O
O
Normalized Intensity
1.36 0.92 0.89 1.22 0.95 0.89 0.92 2.85 2.00 3.63 4.33 3.65
Chemical Shift (ppm)

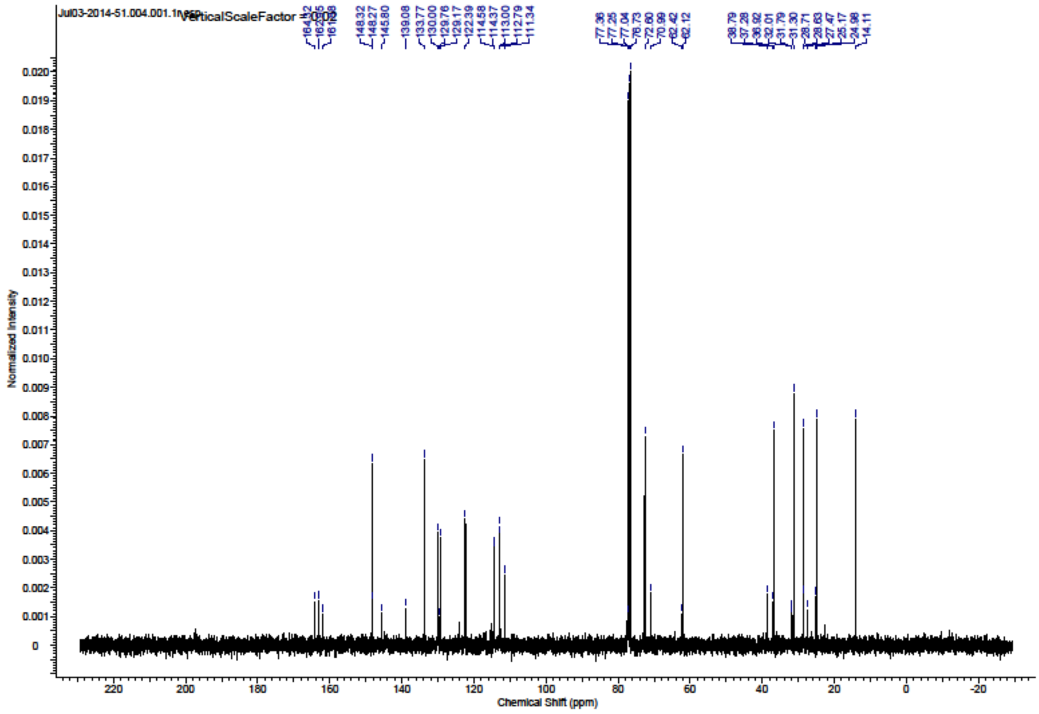

Jul03-2014-51.004.001.1r.esp
VerticalScaleFactor = 0.02
148.32
148.27
145.80
139.08
133.77
130.00
129.76
129.17
122.39
114.58
114.37
113.00
112.79
111.34
77.36
77.25
77.04
76.73
72.60
70.99
62.42
62.12
38.79
37.28
36.92
32.01
31.79
31.30
28.71
28.63
27.47
25.17
24.98
14.11
Normalized Intensity
Chemical Shift (ppm)

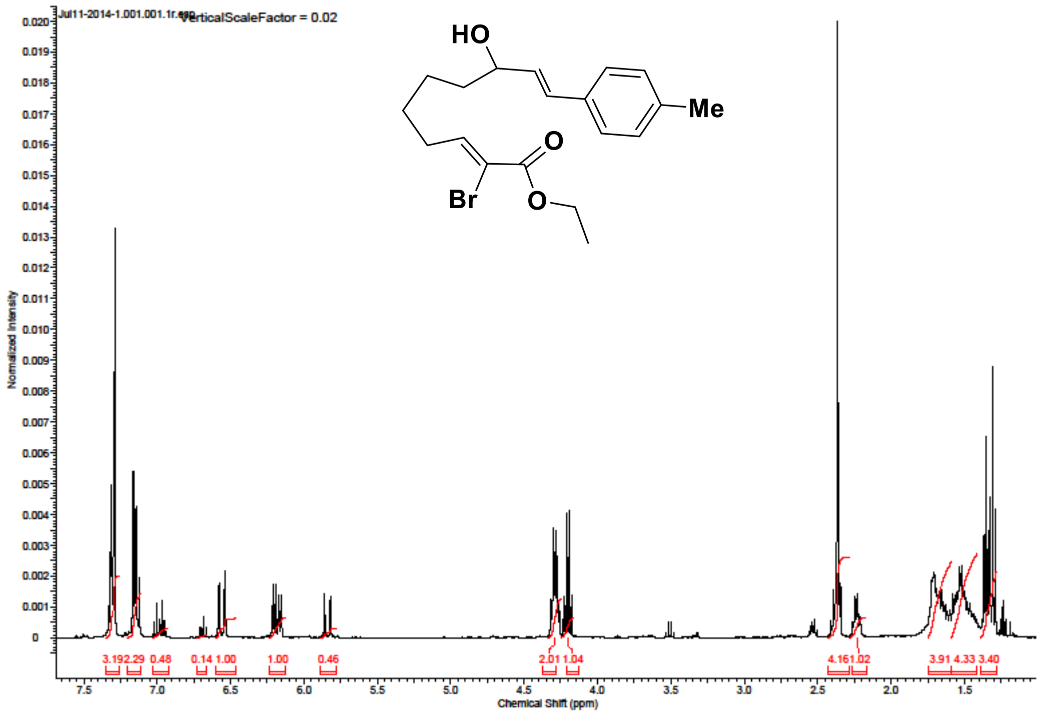

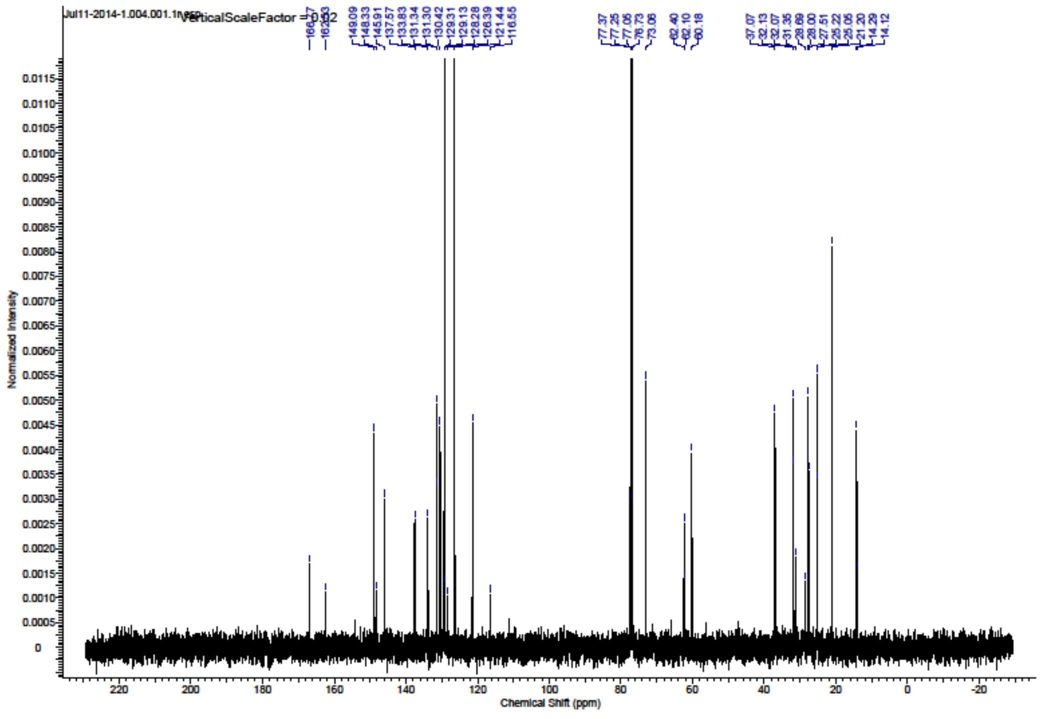

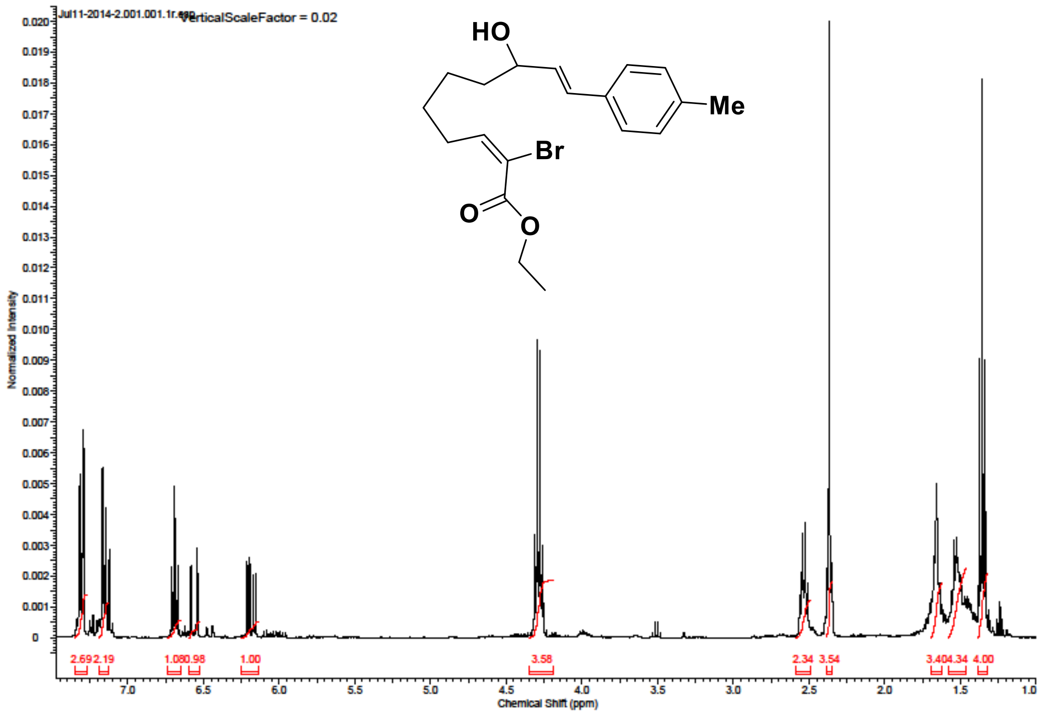
Jul11-2014-2.001.001.1r.esp
VerticalScaleFactor = 0.02
HO
Me
Br
O
O
2.69
2.19
1.08
0.98
1.00
3.58
2.34
3.54
3.40
4.34
4.00
Chemical Shift (ppm)
Normalized Intensity

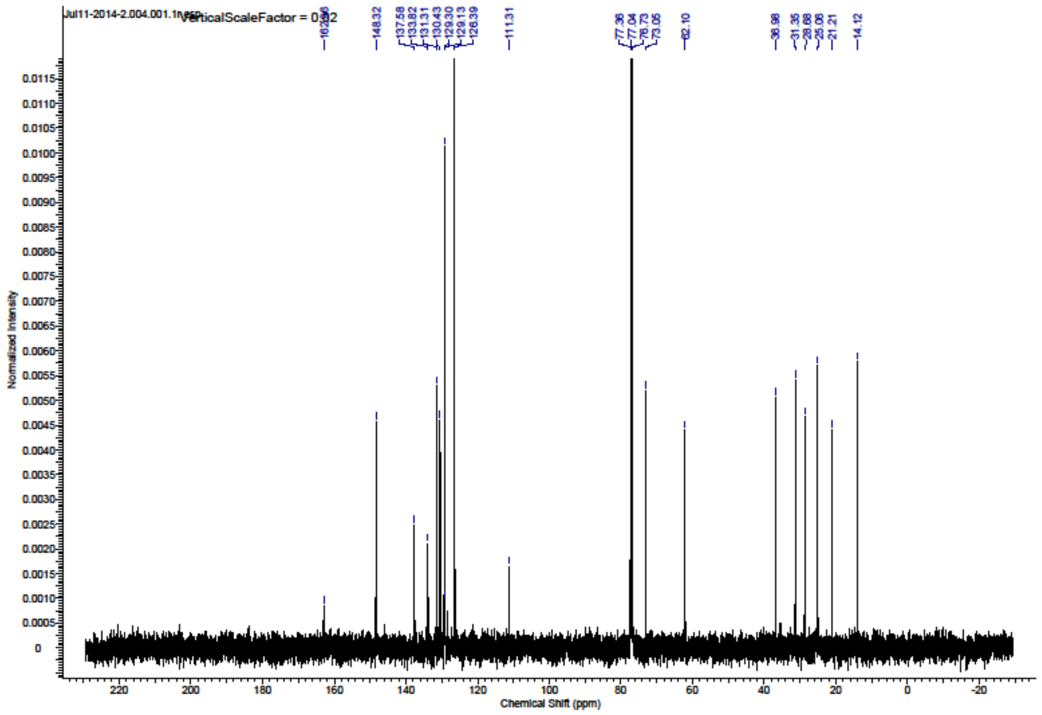
Jul11-2014-2.004.001.1r.esp
VerticalScaleFactor = 0.02
148.32
137.58
133.82
131.31
130.43
129.30
129.13
126.39
111.31
77.36
77.04
76.73
73.05
62.10
36.98
31.36
28.68
25.06
21.21
14.12
Chemical Shift (ppm)
Normalized Intensity

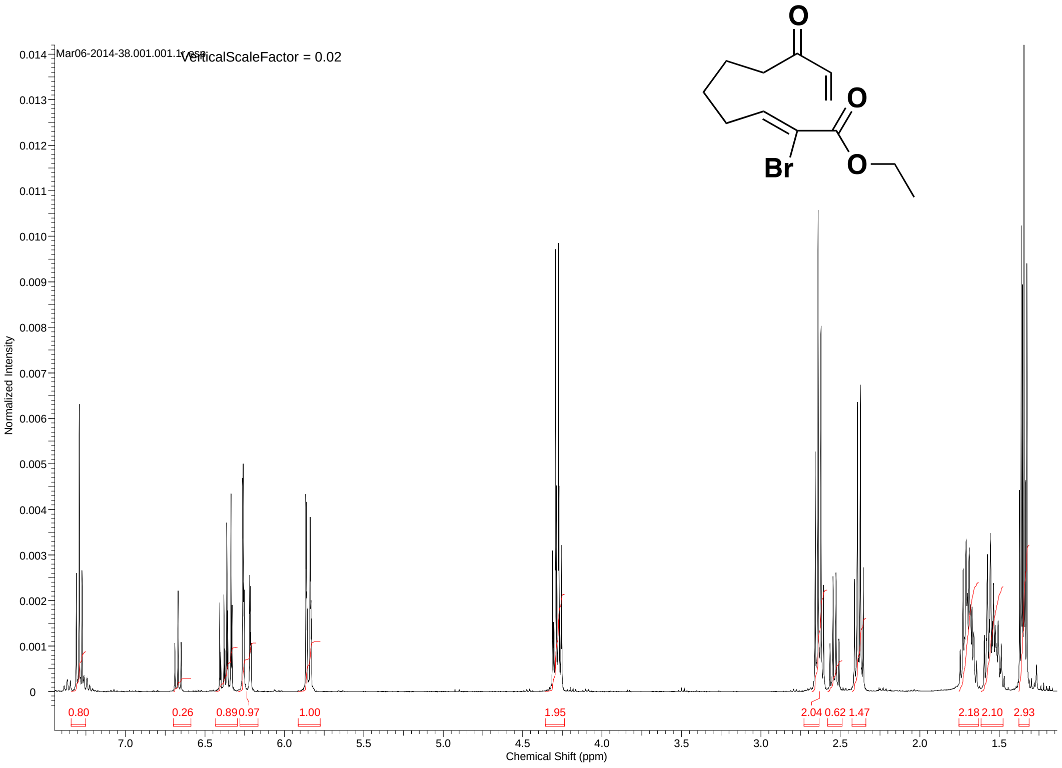
Mar06-2014-38.001.001.1r.esp
VerticalScaleFactor = 0.02
Normalized Intensity
Chemical Shift (ppm)
0.80 0.26 0.89 0.97 1.00 1.95 2.04 0.62 1.47 2.18 2.10 2.93
O
O
Br
O

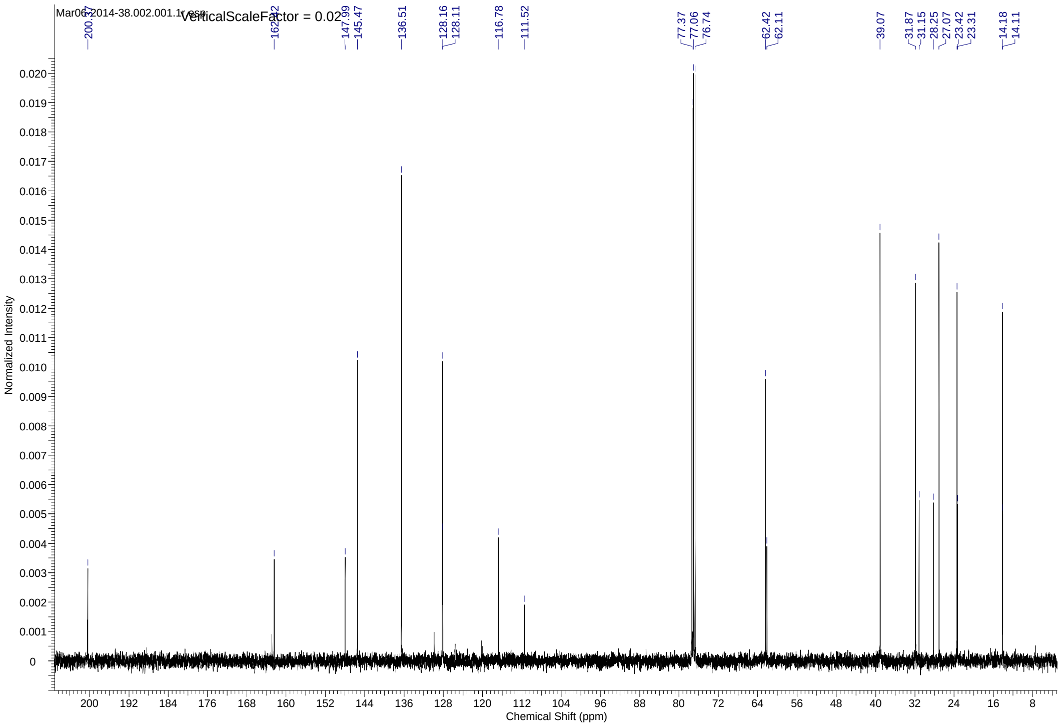
Mar06-2014-38.002.001.1r.esp
VerticalScaleFactor = 0.02
200.37
162.42
147.99
145.47
136.51
128.16
128.11
116.78
111.52
77.37
77.06
76.74
62.42
62.11
39.07
31.87
31.15
28.25
27.07
23.42
23.31
14.18
14.11
Normalized Intensity
Chemical Shift (ppm)

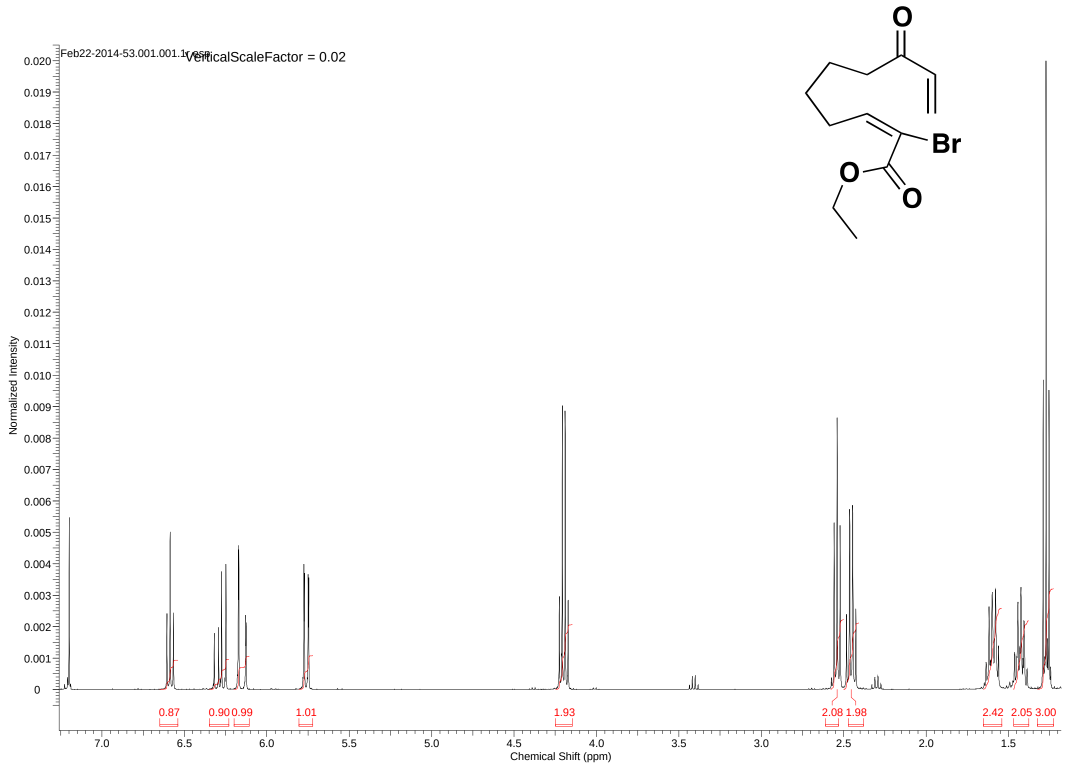
Feb22-2014-53.001.001.1r.esp
VerticalScaleFactor = 0.02
Normalized Intensity
Chemical Shift (ppm)
0.87
0.90
0.99
1.01
1.93
2.08
1.98
2.42
2.05
3.00
O
Br
O
O

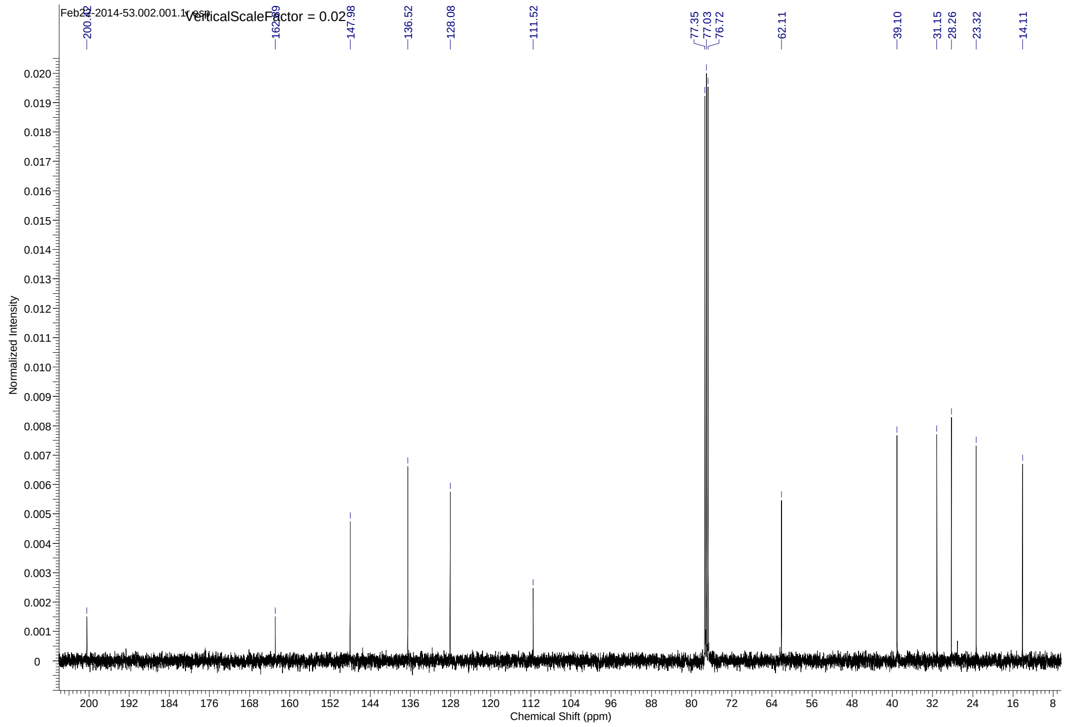
Feb22-2014-53.002.001.1r.esp
VerticalScaleFactor = 0.02
200.32
162.99
147.98
136.52
128.08
111.52
77.35
77.03
76.72
62.11
39.10
31.15
28.26
23.32
14.11
Normalized Intensity
Chemical Shift (ppm)

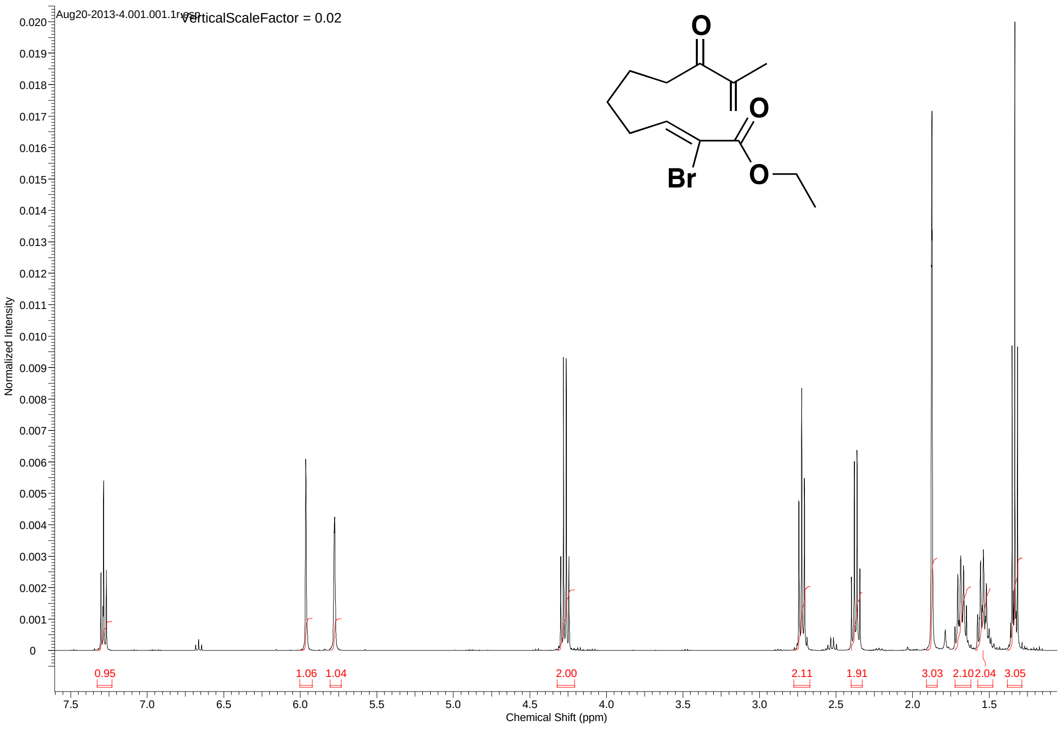
Aug20-2013-4.001.001.1r.esp
VerticalScaleFactor = 0.02
O
O
Br
O
Normalized Intensity
Chemical Shift (ppm)
0.95
1.06
1.04
2.00
2.11
1.91
3.03
2.10
2.04
3.05

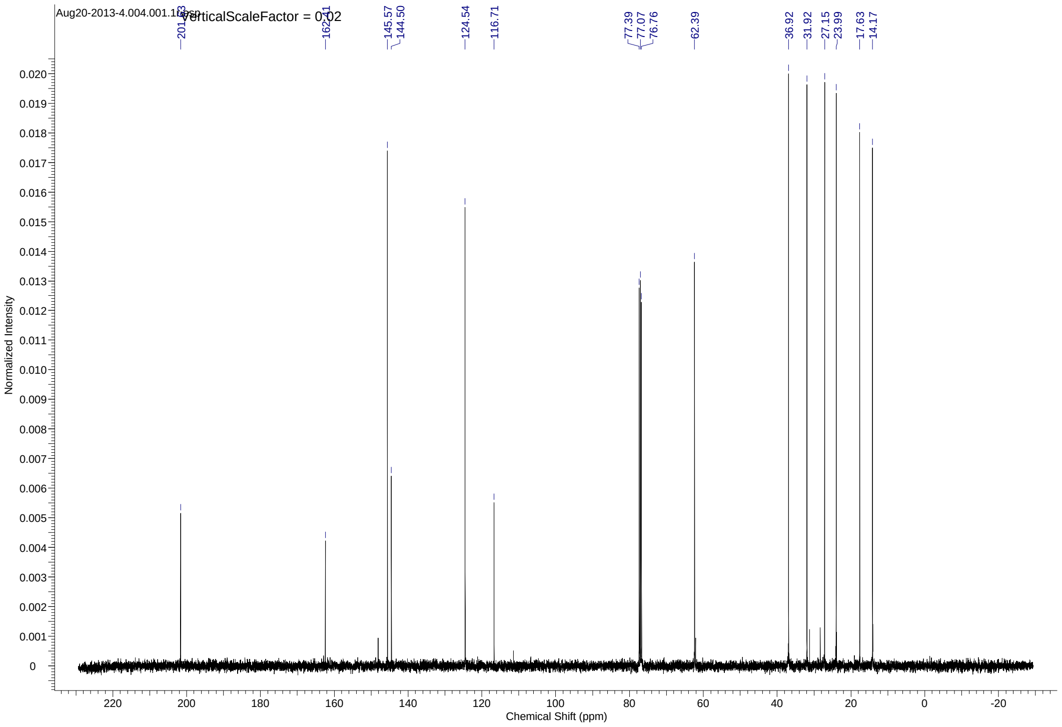
Aug20-2013-4.004.001.1r.esp
VerticalScaleFactor = 0.02
201.13
162.11
145.57
144.50
124.54
116.71
77.39
77.07
76.76
62.39
36.92
31.92
27.15
23.99
17.63
14.17
Normalized Intensity
Chemical Shift (ppm)

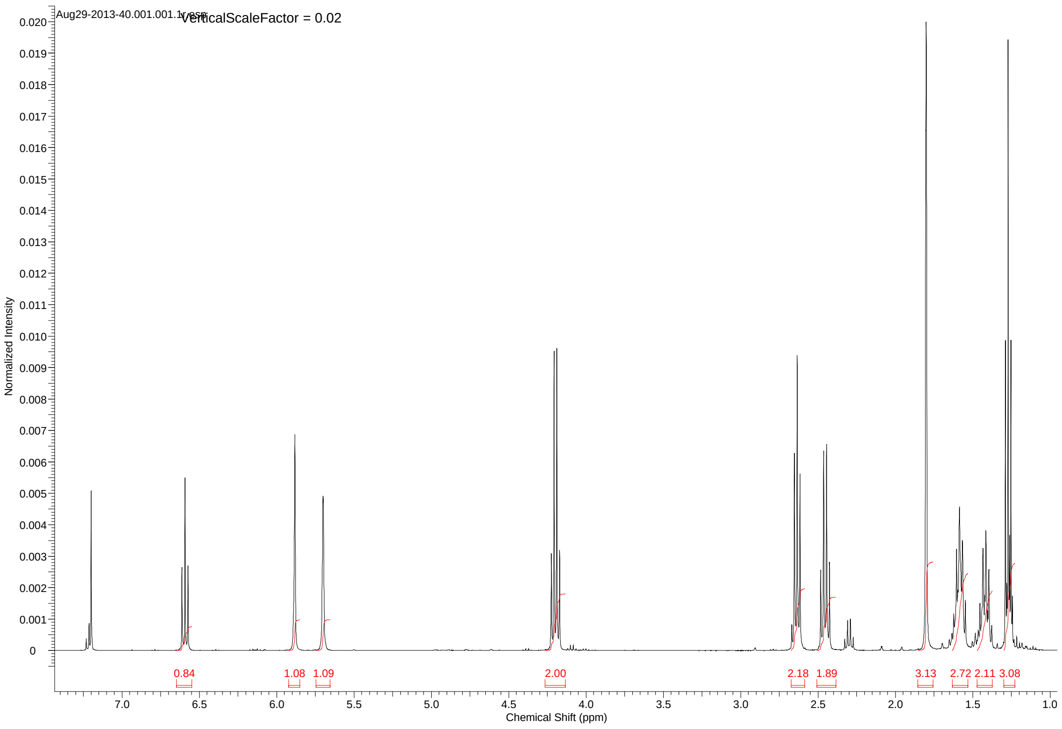
Aug29-2013-40.001.001.1r.esp
VerticalScaleFactor = 0.02
Normalized Intensity
0.84
1.08
1.09
2.00
2.18
1.89
3.13
2.72
2.11
3.08
Chemical Shift (ppm)

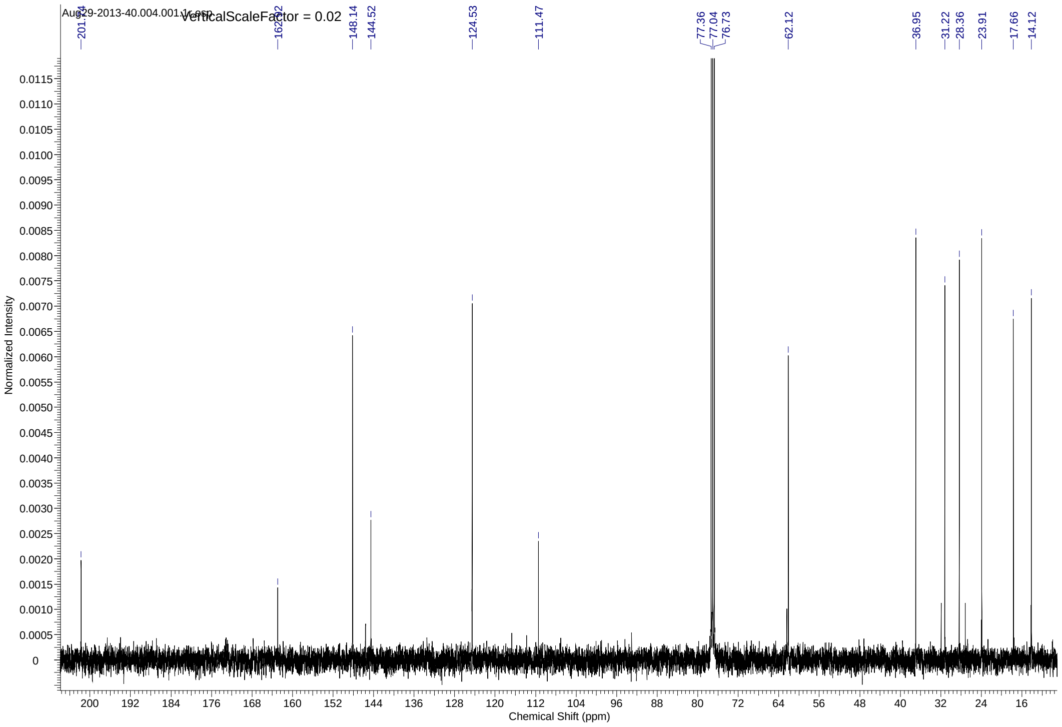
Aug29-2013-40.004.001.1r.esp
VerticalScaleFactor = 0.02
201.34
162.92
148.14
144.52
124.53
111.47
77.36
77.04
76.73
62.12
36.95
31.22
28.36
23.91
17.66
14.12
Normalized Intensity
Chemical Shift (ppm)

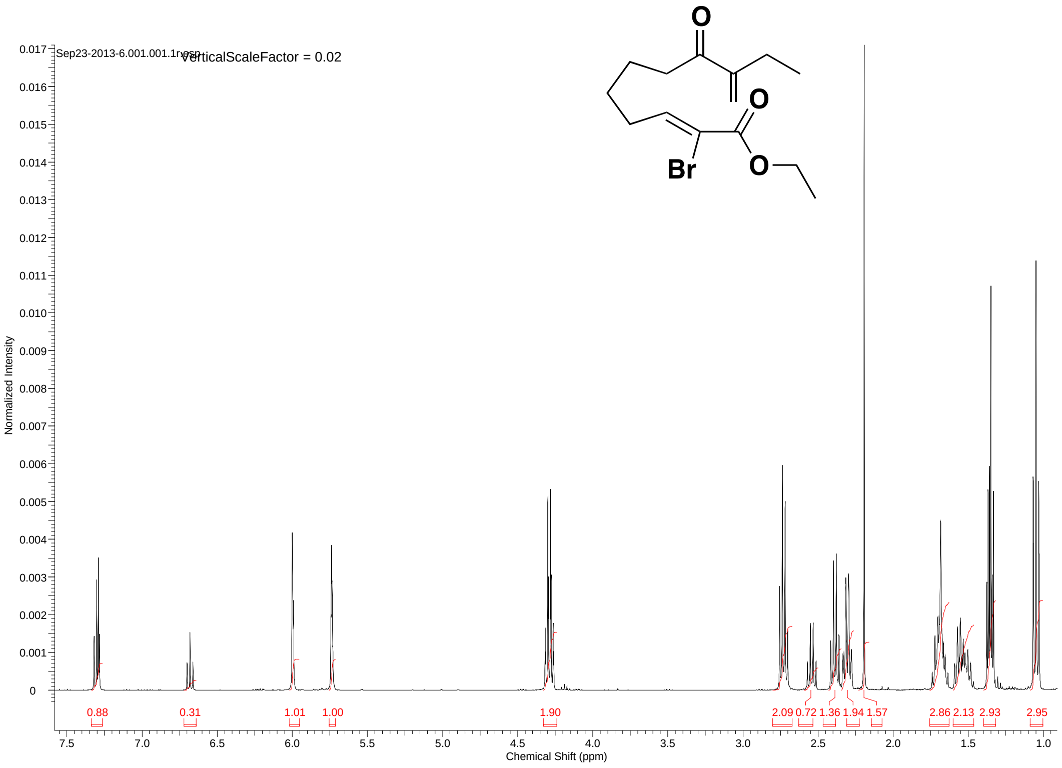

Sep23-2013-6.001.001.1r.esp
VerticalScaleFactor = 0.02
O
O
Br
O
Normalized Intensity
Chemical Shift (ppm)
0.88
0.31
1.01
1.00
1.90
2.09
0.72
1.36
1.94
1.57
2.86
2.13
2.93
2.95

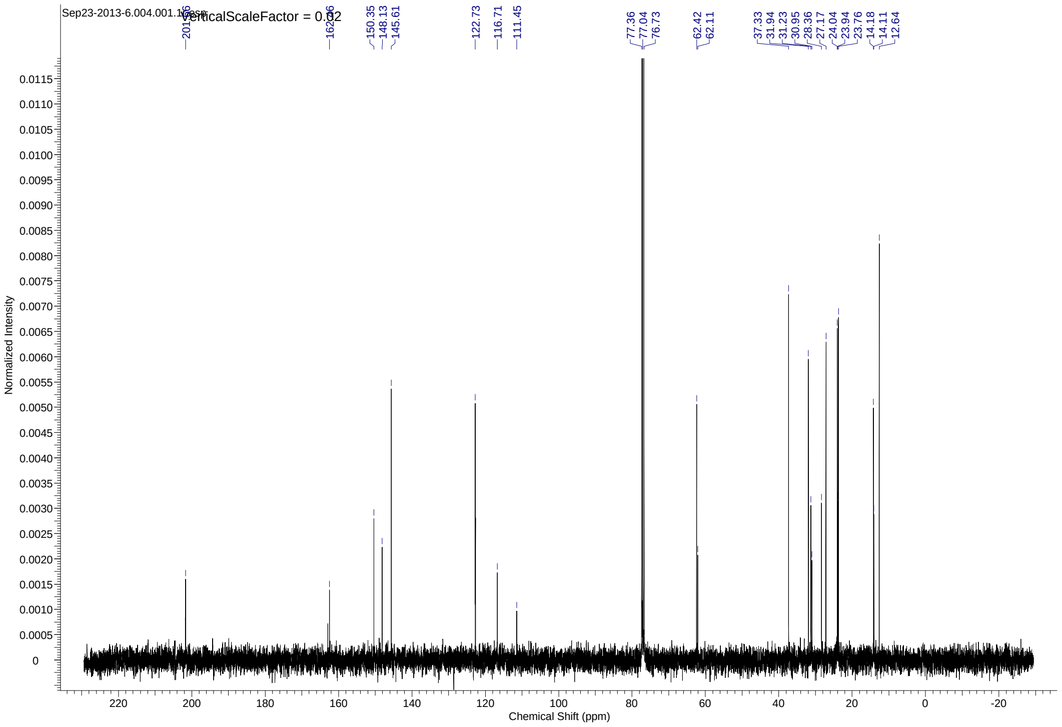

Sep23-2013-6.004.001.1r.esp
VerticalScaleFactor = 0.02
201.55
162.36
150.35
148.13
145.61
122.73
116.71
111.45
77.36
77.04
76.73
62.42
62.11
37.33
31.94
31.23
30.95
28.36
27.17
24.04
23.94
23.76
14.18
14.11
12.64
Normalized Intensity
Chemical Shift (ppm)

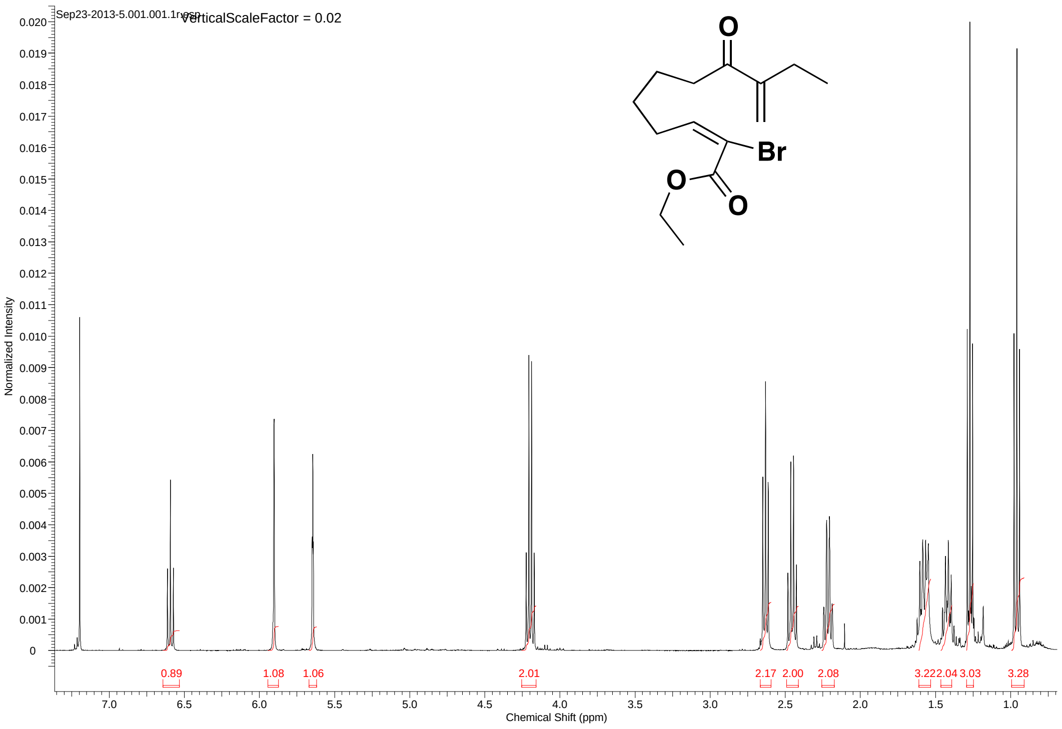
Sep23-2013-5.001.001.1r.esp
VerticalScaleFactor = 0.02
O
Br
O
O
Normalized Intensity
0.89
1.08
1.06
2.01
2.17
2.00
2.08
3.22
2.04
3.03
3.28
Chemical Shift (ppm)

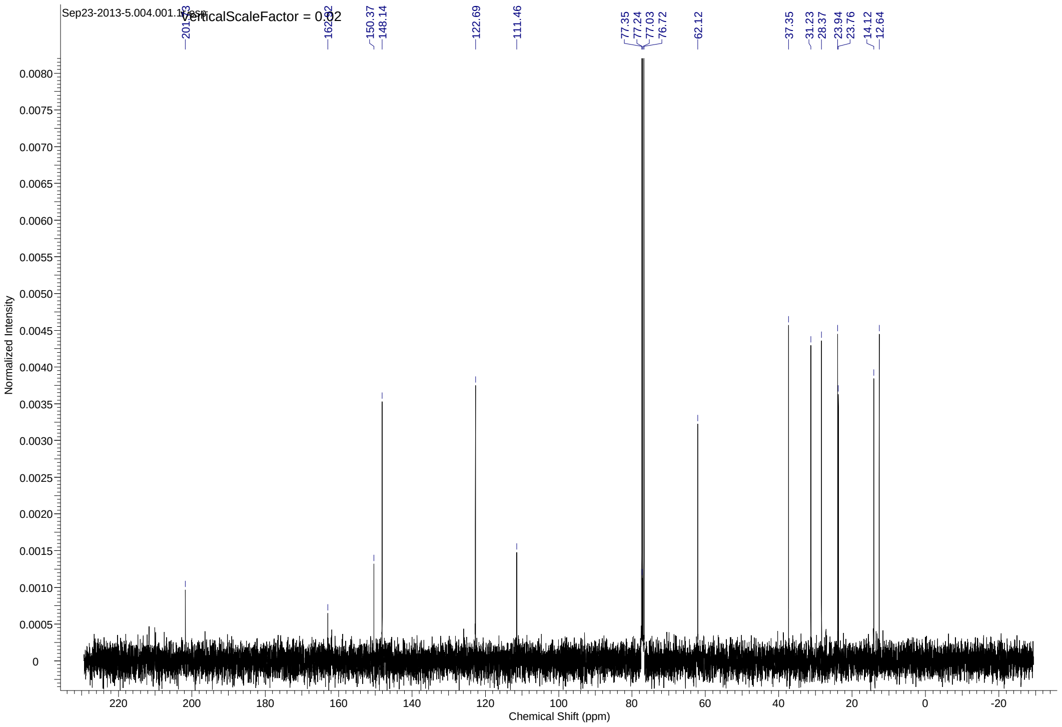
Sep23-2013-5.004.001.1r.esp
VerticalScaleFactor = 0.02
201.63
162.32
150.37
148.14
122.69
111.46
77.35
77.24
77.03
76.72
62.12
37.35
31.23
28.37
23.94
23.76
14.12
12.64
Normalized Intensity
Chemical Shift (ppm)

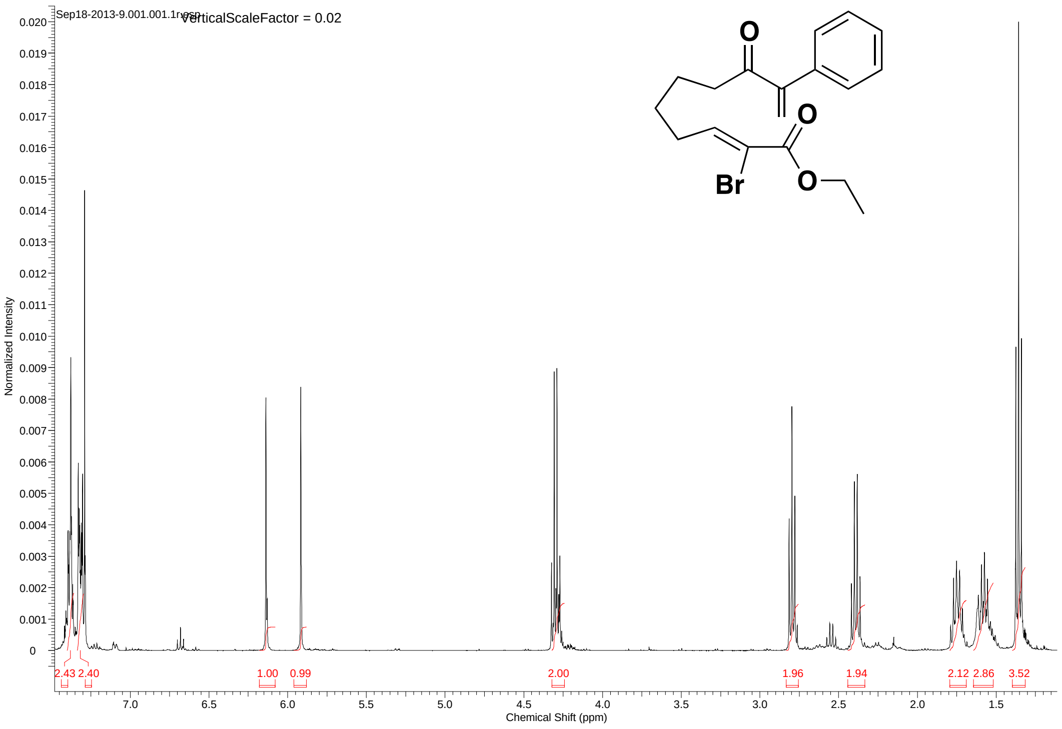
Sep18-2013-9.001.001.1r.esp
VerticalScaleFactor = 0.02
O
O
Br
O
Normalized Intensity
Chemical Shift (ppm)
2.43
2.40
1.00
0.99
2.00
1.96
1.94
2.12
2.86
3.52

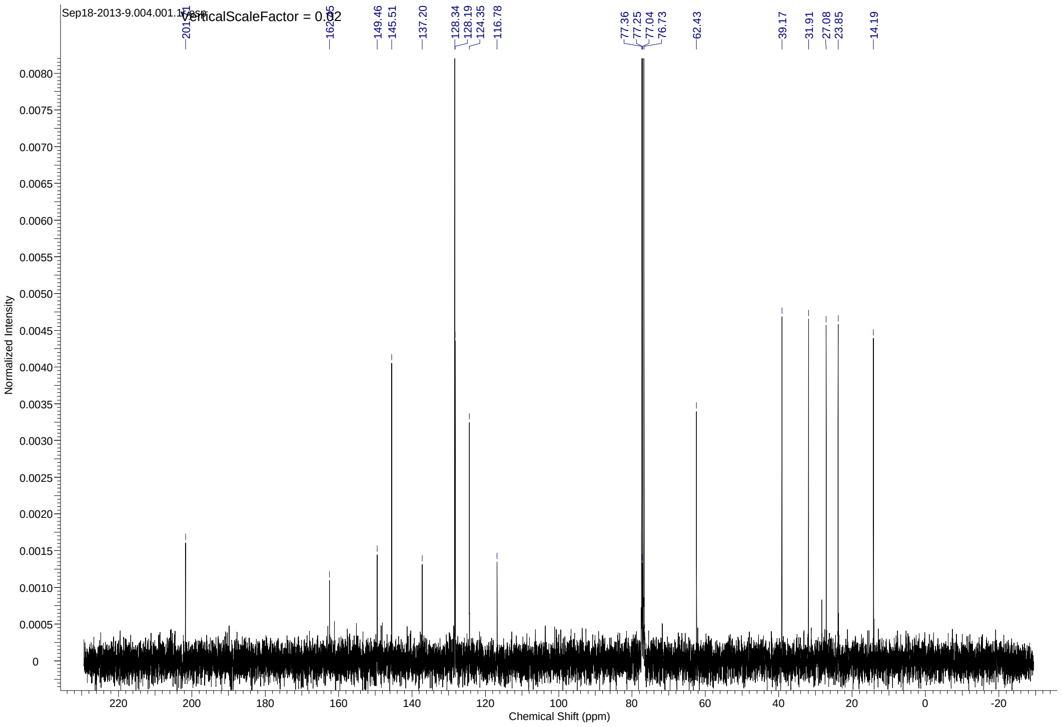
Sep18-2013-9.004.001.1r.esp
VerticalScaleFactor = 0.02
201.71
162.95
149.46
145.51
137.20
128.34
128.19
124.35
116.78
77.36
77.25
77.04
76.73
62.43
39.17
31.91
27.08
23.85
14.19
Normalized Intensity
Chemical Shift (ppm)

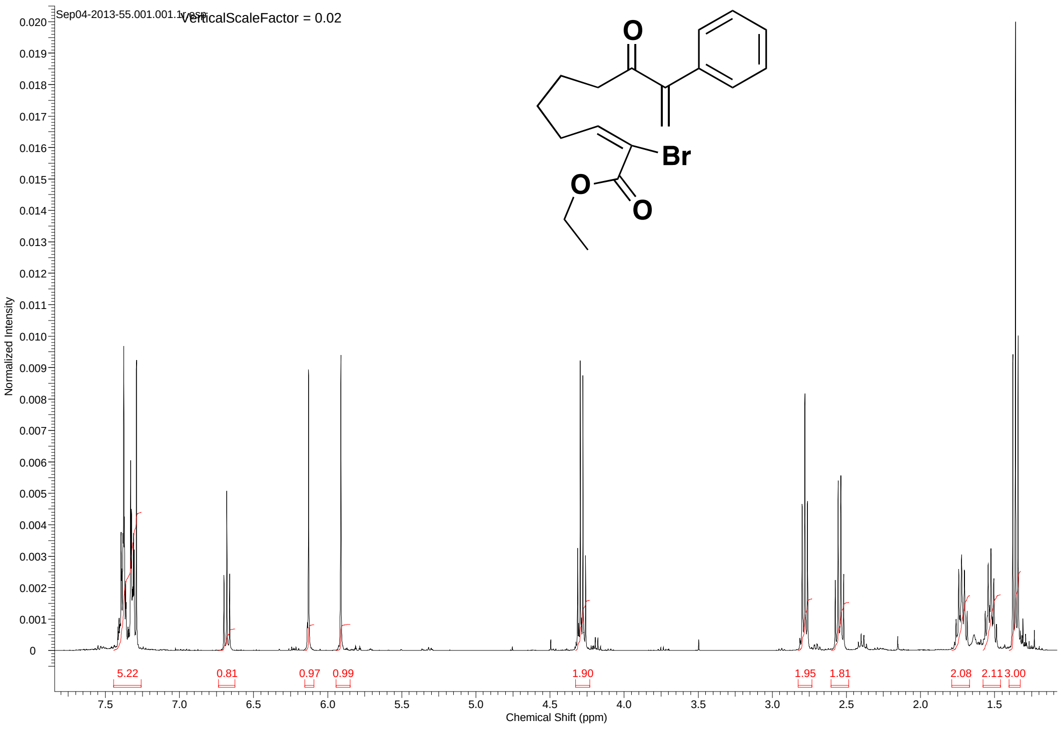
Sep04-2013-55.001.001.1r.esp
VerticalScaleFactor = 0.02
O
Br
O
O
Normalized Intensity
5.22
0.81
0.97
0.99
1.90
1.95
1.81
2.08
2.11
3.00
Chemical Shift (ppm)

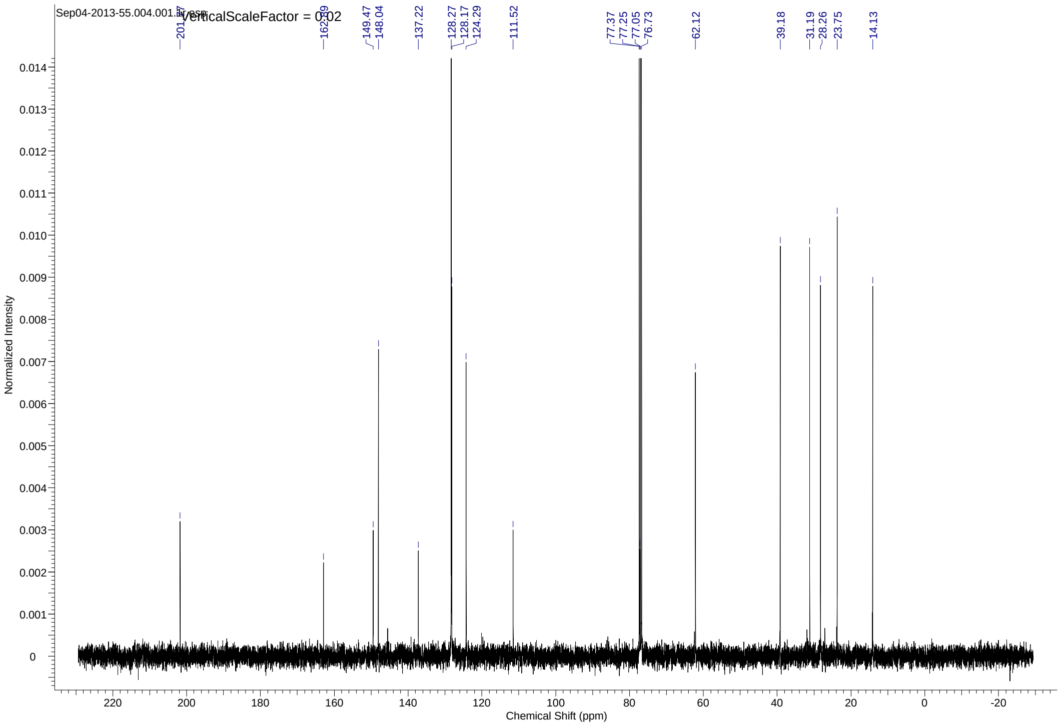
Sep04-2013-55.004.001.1r.esp
VerticalScaleFactor = 0.02
201.37
162.39
149.47
148.04
137.22
128.27
128.17
124.29
111.52
77.37
77.25
77.05
76.73
62.12
39.18
31.19
28.26
23.75
14.13
Normalized Intensity
Chemical Shift (ppm)

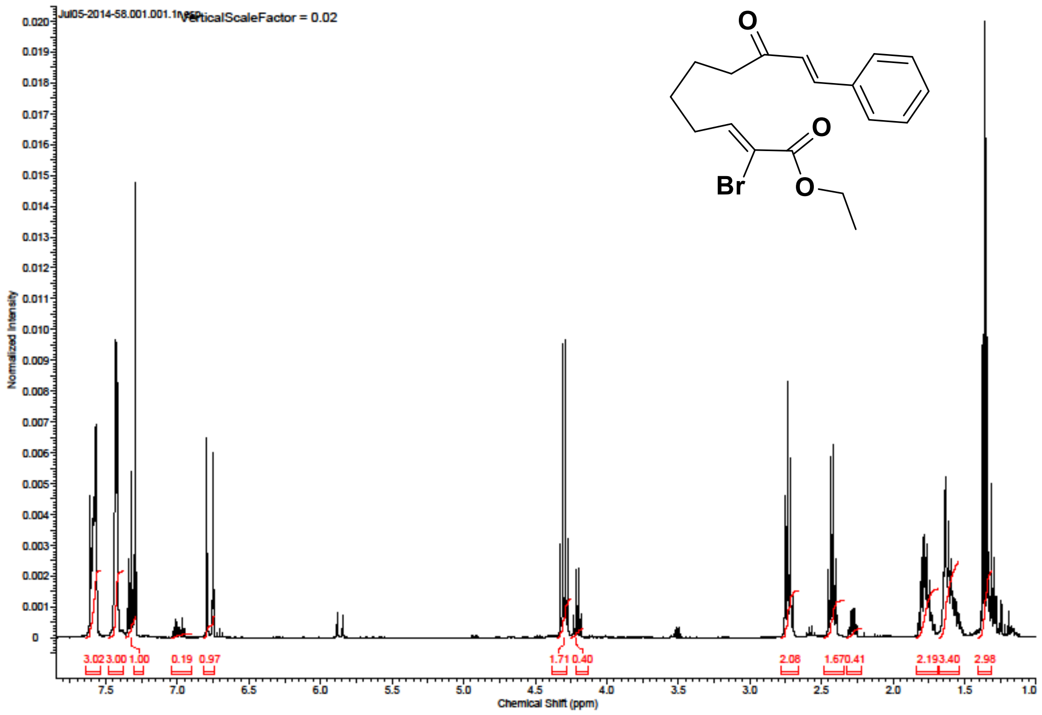

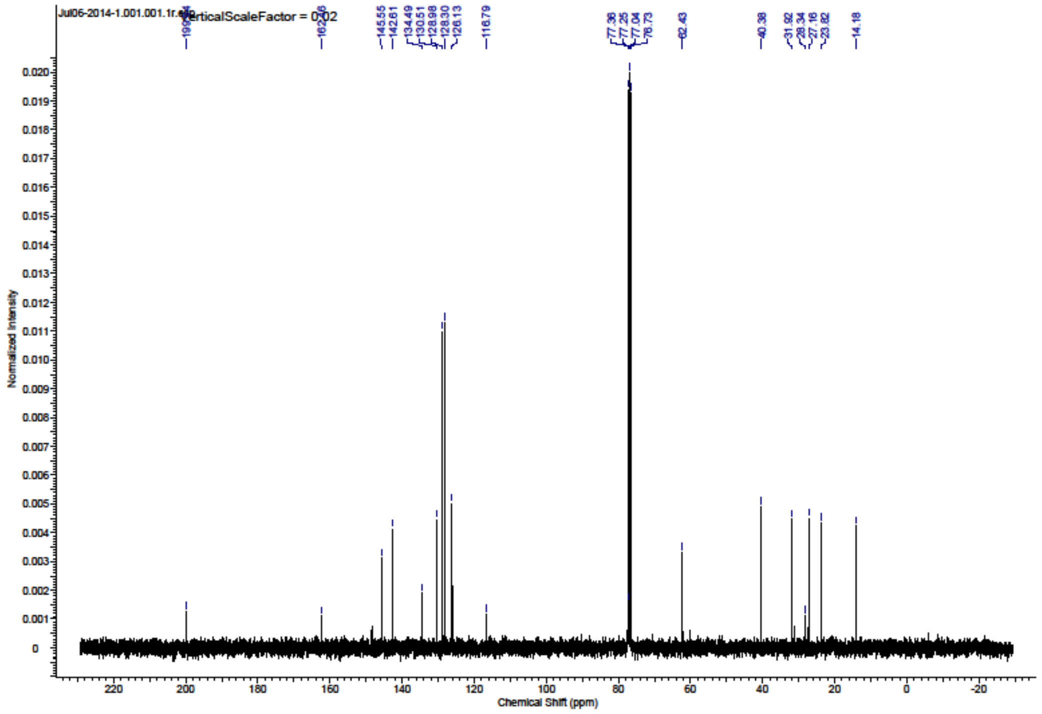

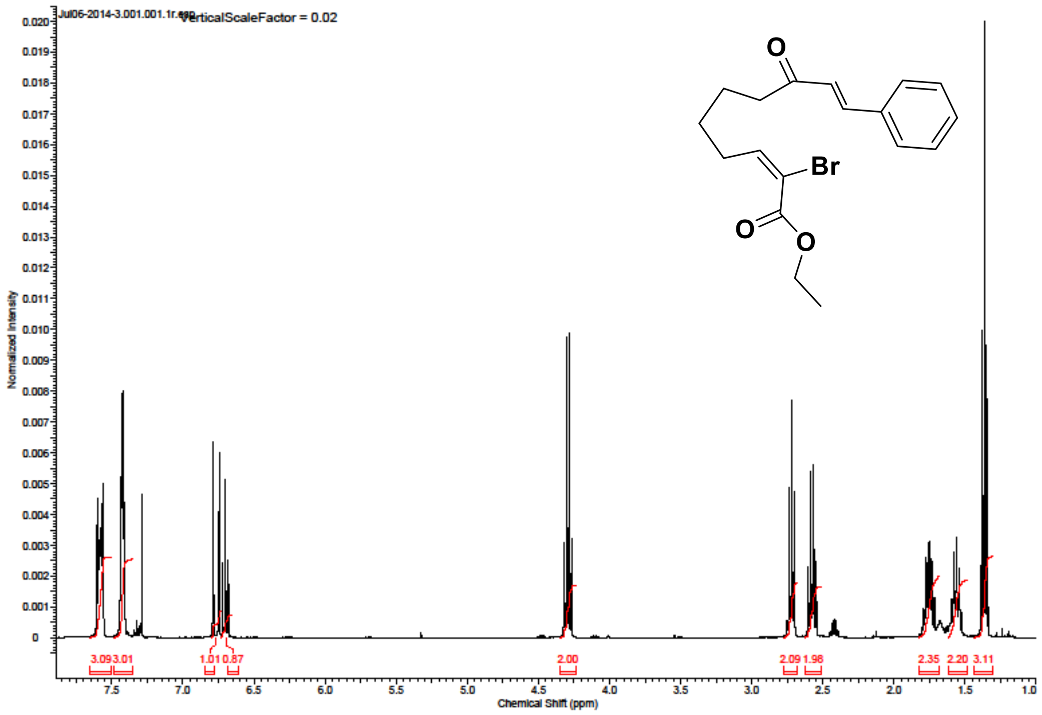
Jul06-2014-3.001.001.1r.esp
VerticalScaleFactor = 0.02
O
Br
O
O
3.09
3.01
1.01
0.87
2.00
2.09
1.96
2.35
2.20
3.11
Normalized Intensity
Chemical Shift (ppm)

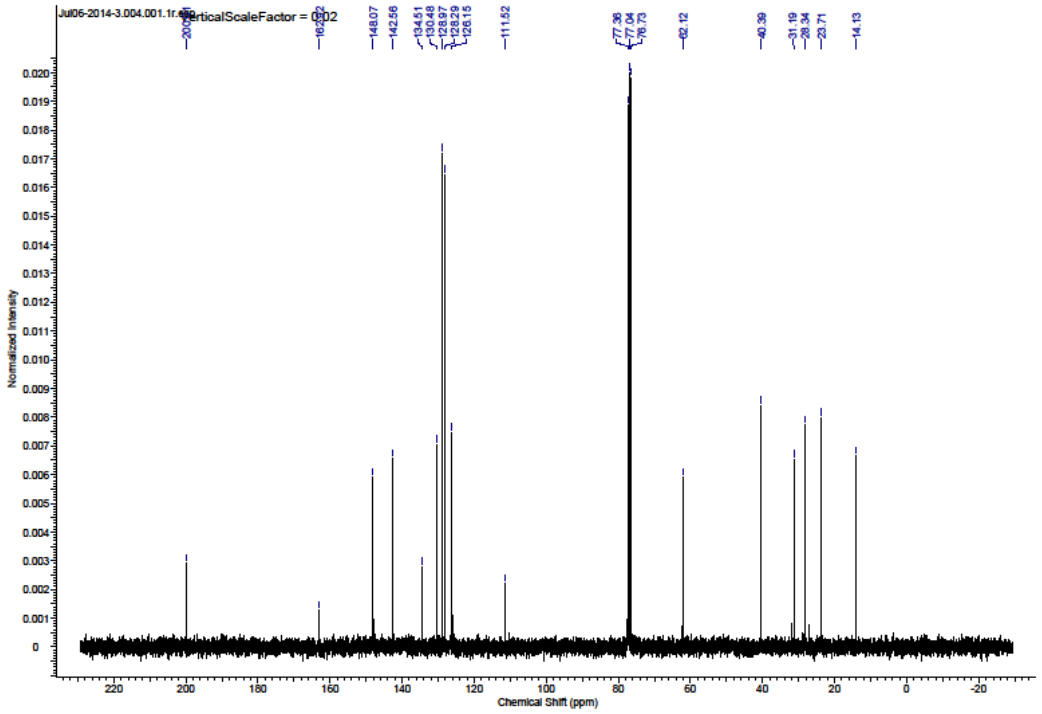
Jul06-2014-3.004.001.1r.esp
VerticalScaleFactor = 0.02
148.07
142.56
134.51
130.48
128.97
128.29
126.15
111.52
77.36
77.04
76.73
62.12
40.39
31.19
28.34
23.71
14.13
Normalized Intensity
Chemical Shift (ppm)

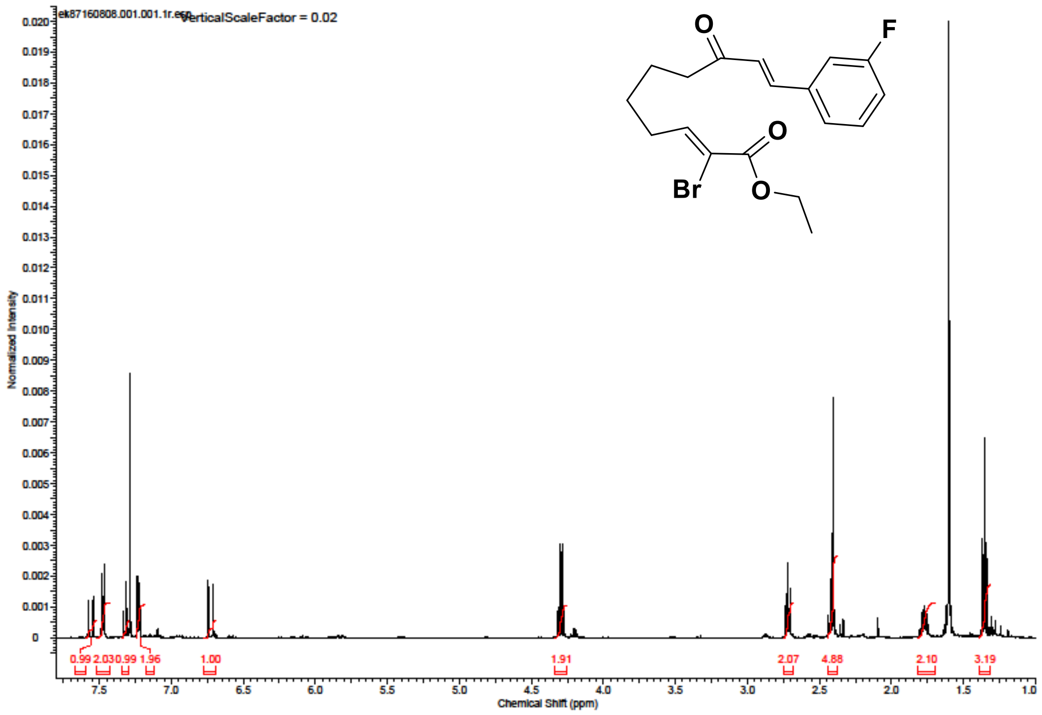
ek87160808.001.001.1r.esp
VerticalScaleFactor = 0.02
O
F
O
Br
O
Normalized Intensity
0.99
2.03
0.99
1.96
1.00
1.91
2.07
4.88
2.10
3.19
Chemical Shift (ppm)

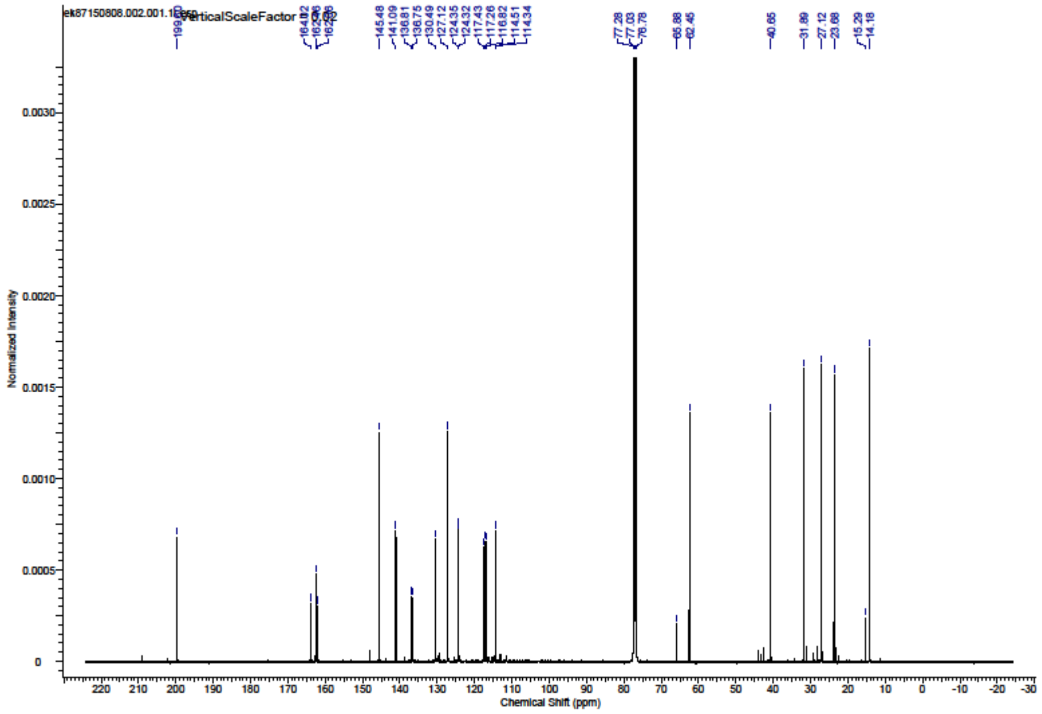
ek87150808.002.001.1r.esp
VerticalScaleFactor = 0.02
145.48
141.09
136.81
136.75
130.49
127.12
124.35
124.32
117.43
117.26
116.82
114.51
114.34
77.28
77.03
76.78
65.88
62.45
40.65
31.99
27.12
23.68
15.29
14.18
Normalized Intensity
Chemical Shift (ppm)

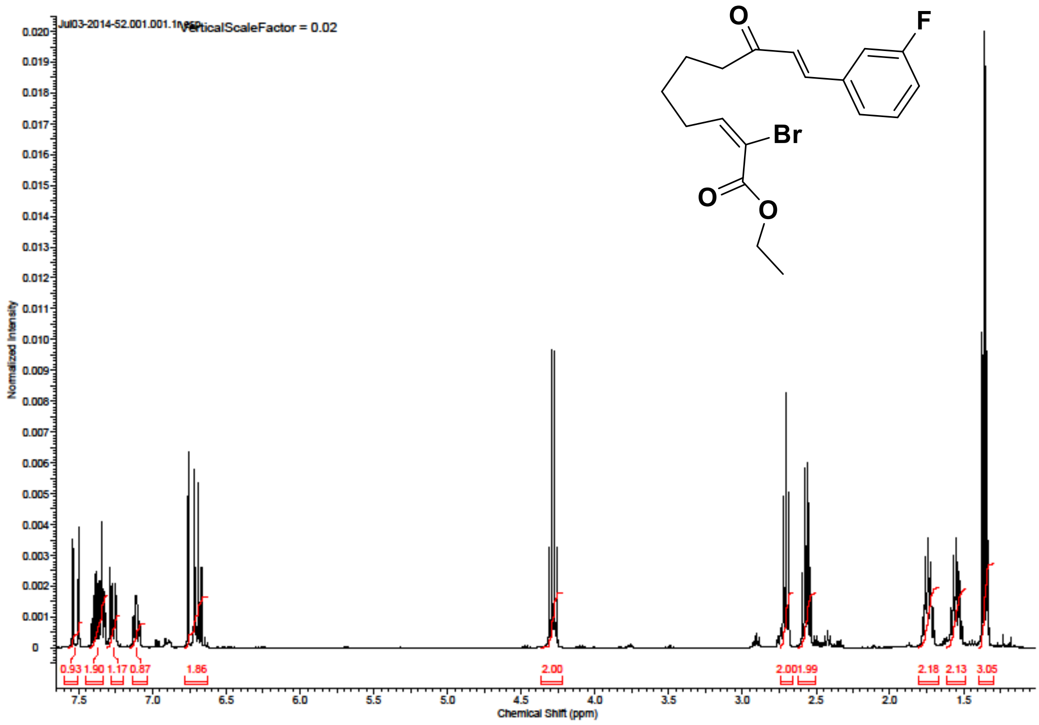
Jul03-2014-52.001.001.1r.esp
VerticalScaleFactor = 0.02
Normalized Intensity
Chemical Shift (ppm)
0.93 1.90 1.17 0.87 1.86 2.00 2.00 1.99 2.18 2.13 3.05
O
F
Br
O
O

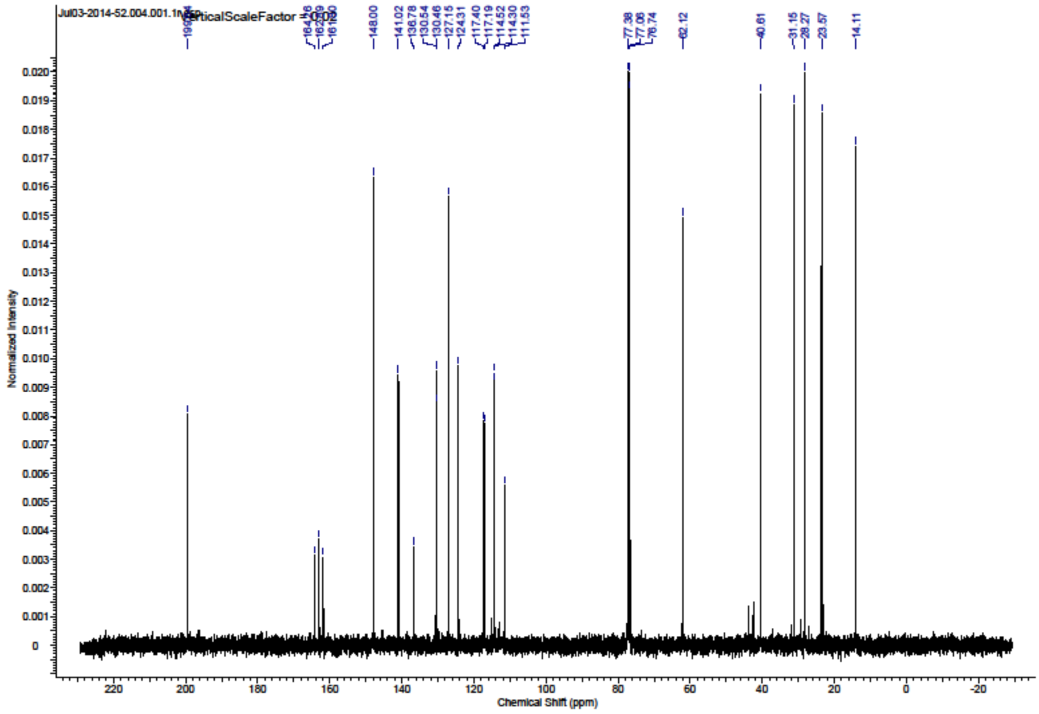
Jul03-2014-52.004.001.1r.esp
VerticalScaleFactor = 0.02
Normalized Intensity
Chemical Shift (ppm)
148.00
141.02
136.78
130.54
130.46
127.15
124.31
117.40
117.19
114.52
114.30
111.53
77.38
77.06
76.74
62.12
40.61
31.15
28.27
23.57
14.11

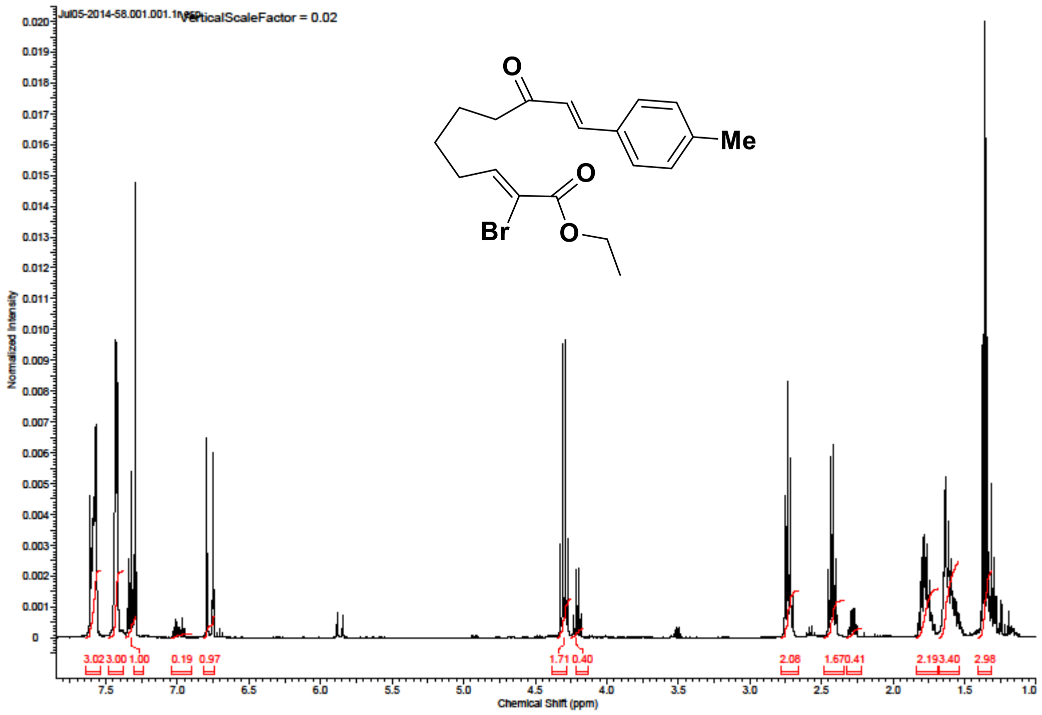
Jul05-2014-58.001.001.1r.esp
VerticalScaleFactor = 0.02
O
Me
O
Br
O
Normalized Intensity
3.02 3.00 1.00 0.19 0.97 1.71 0.40 2.08 1.67 0.41 2.19 3.40 2.98
Chemical Shift (ppm)

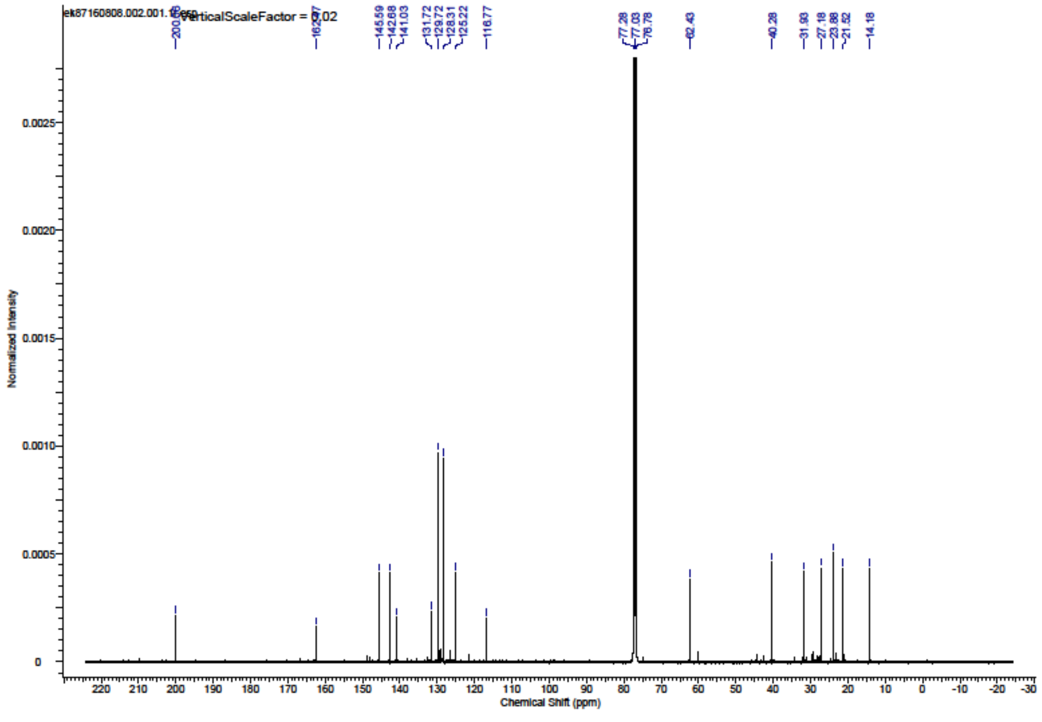
ek87160808.002.001.1r.esp
VerticalScaleFactor = 0.02
145.59
142.68
141.03
131.72
129.72
128.31
125.22
116.77
77.28
77.03
76.78
62.43
40.28
31.93
27.18
23.88
21.52
14.18
Normalized Intensity
Chemical Shift (ppm)

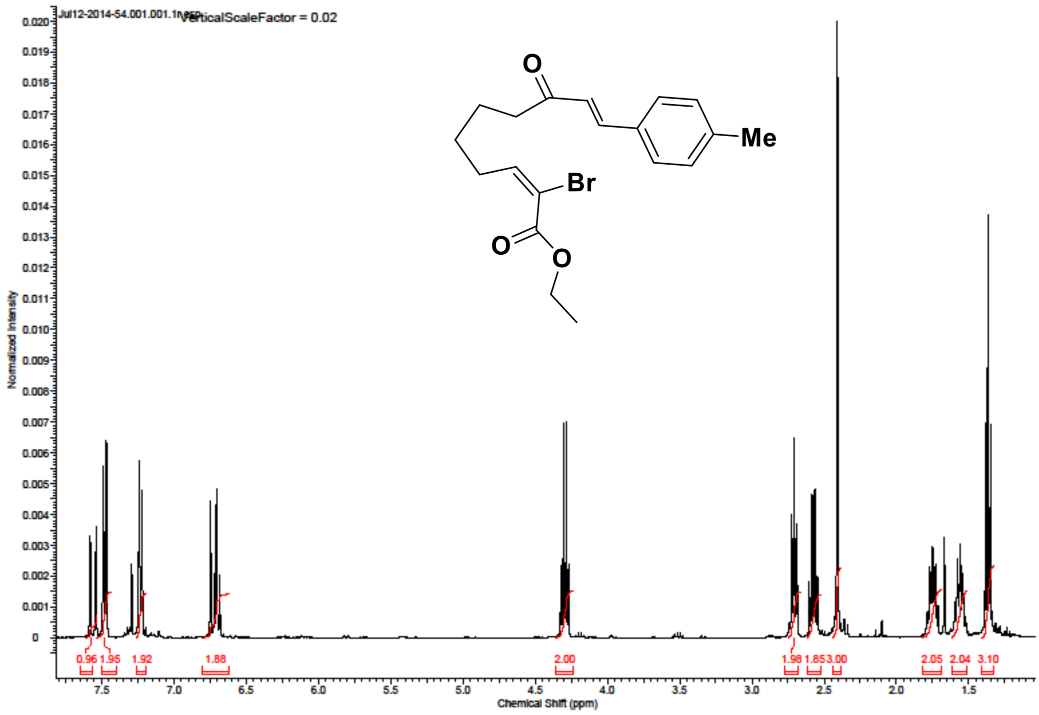

VerticalScaleFactor = 0.02
O
Br
Me
O
O
Normalized Intensity
0.96 1.95 1.92 1.88 2.00 1.98 1.85 3.00 2.05 2.04 3.10
Chemical Shift (ppm)

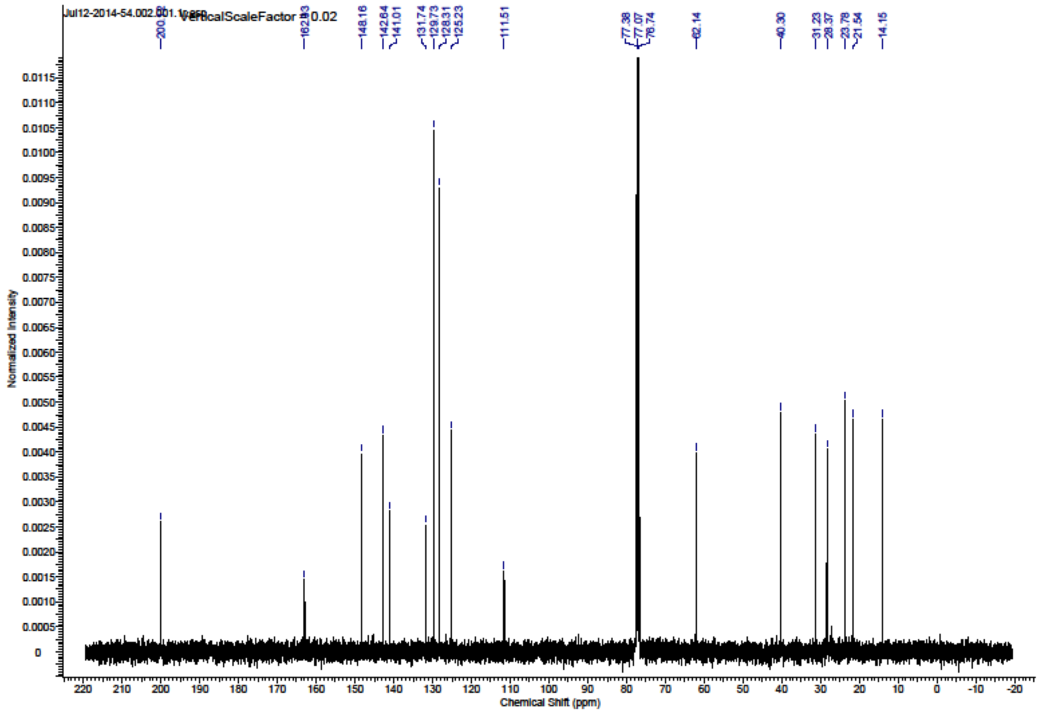

VerticalScaleFactor = 0.02
148.16
142.64
141.01
131.74
129.73
128.31
125.23
111.51
77.38
77.07
76.74
62.14
40.30
31.23
28.37
23.78
21.54
14.15
Normalized Intensity
Chemical Shift (ppm)

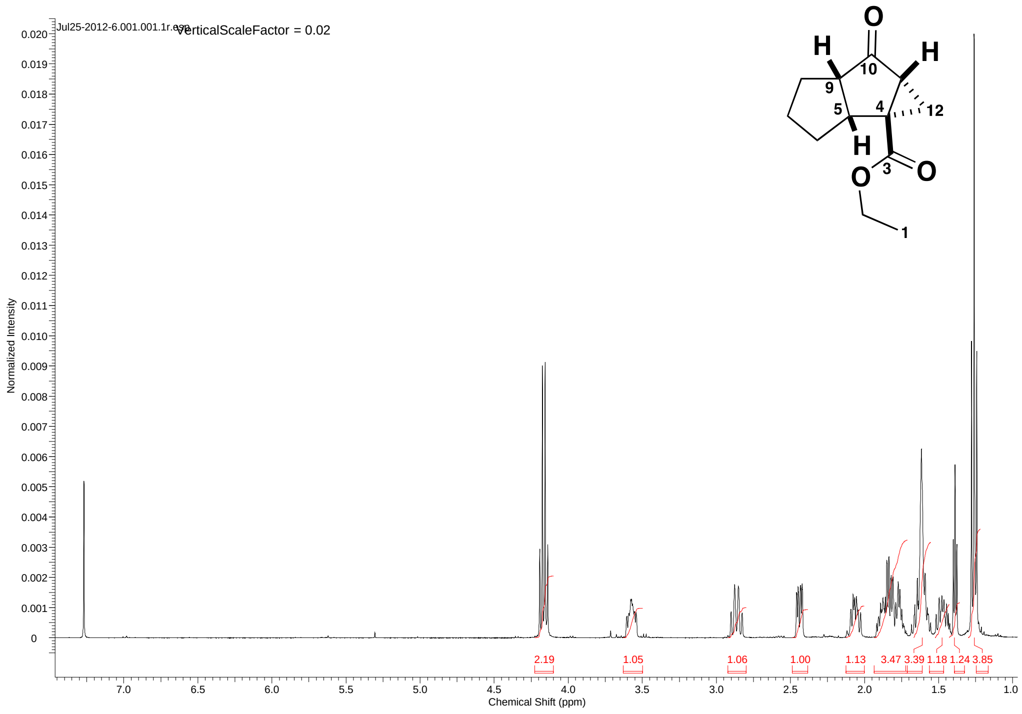
Jul25-2012-6.001.001.1r.esp
VerticalScaleFactor = 0.02
Normalized Intensity
Chemical Shift (ppm)
2.19
1.05
1.06
1.00
1.13
3.47
3.39
1.18
1.24
3.85
O
H
H
H
10
9
5
4
12
3
O
O
1

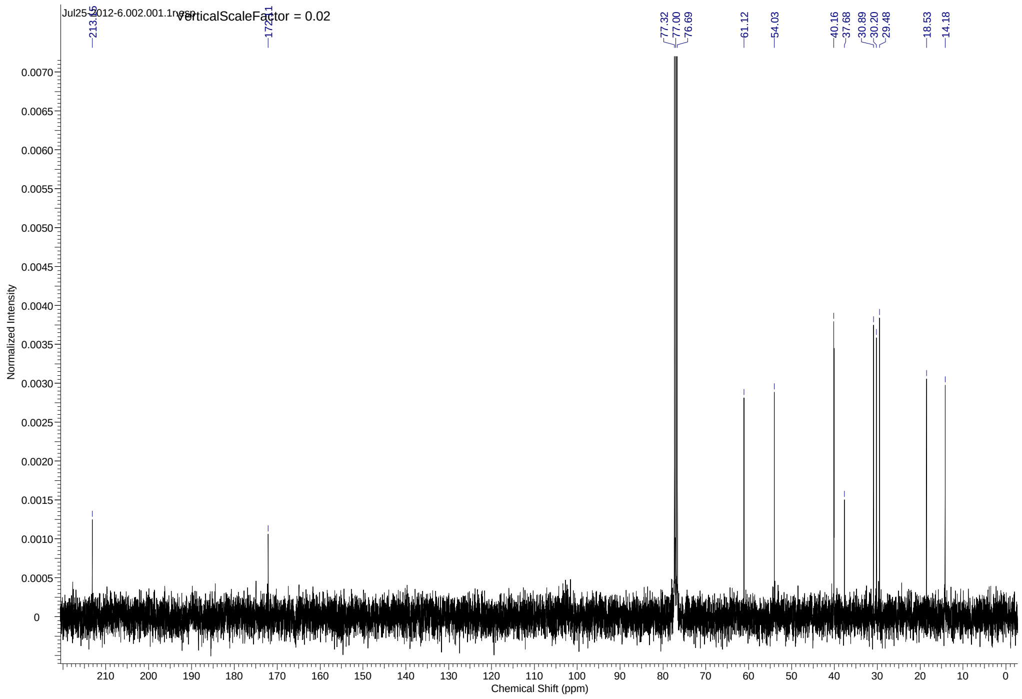
Jul25-2012-6.002.001.1r.esp
VerticalScaleFactor = 0.02
Normalized Intensity
Chemical Shift (ppm)
213.45
172.11
77.32
77.00
76.69
61.12
54.03
40.16
37.68
30.89
30.20
29.48
18.53
14.18

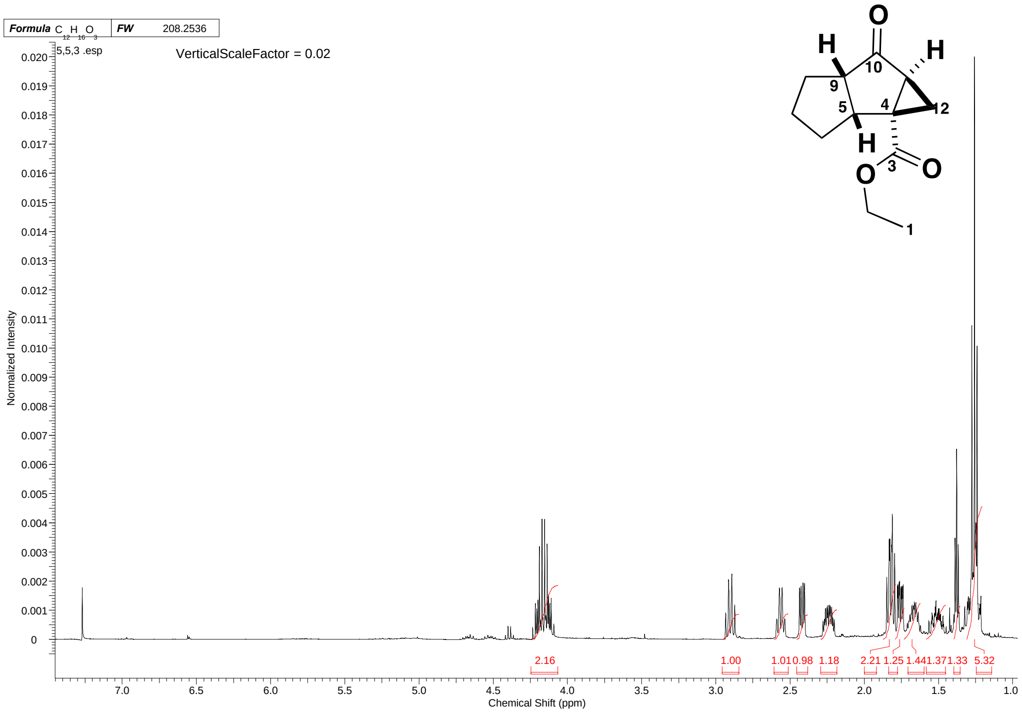
Formula C12H16O3
FW 208.2536
5,5,3 .esp
VerticalScaleFactor = 0.02
Normalized Intensity
Chemical Shift (ppm)
2.16
1.00
1.01
0.98
1.18
2.21
1.25
1.44
1.37
1.33
5.32
O
H
H
10
9
5
4
12
H
3
O
O
1

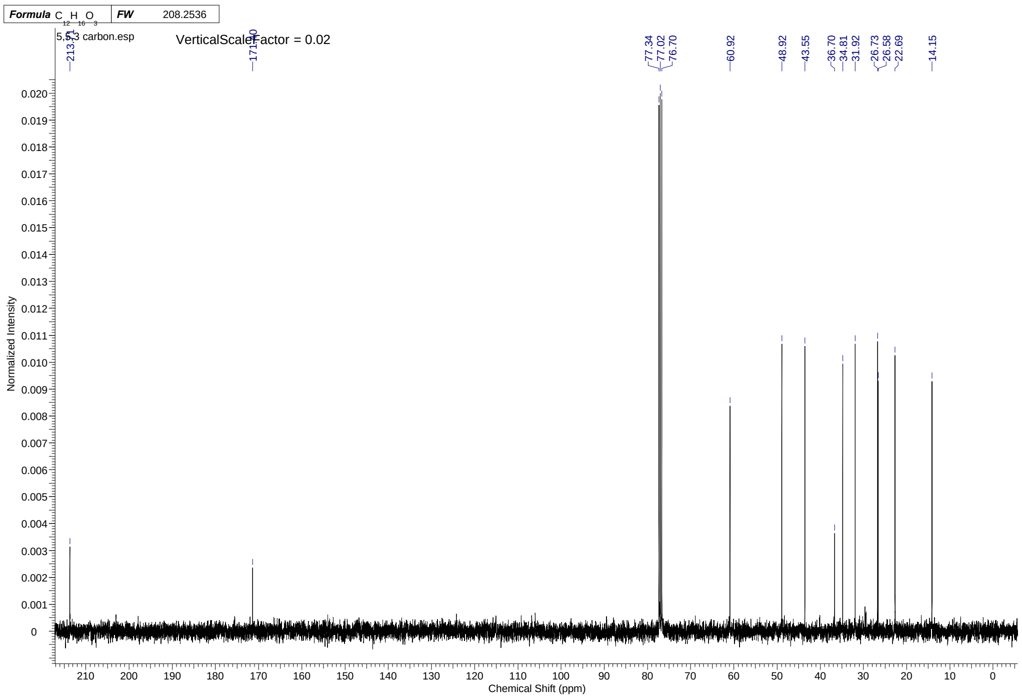
Formula C12H16O3
FW 208.2536
5,5,3 carbon.esp
VerticalScaleFactor = 0.02
77.34
77.02
76.70
60.92
48.92
43.55
36.70
34.81
31.92
26.73
26.58
22.69
14.15
Normalized Intensity
Chemical Shift (ppm)

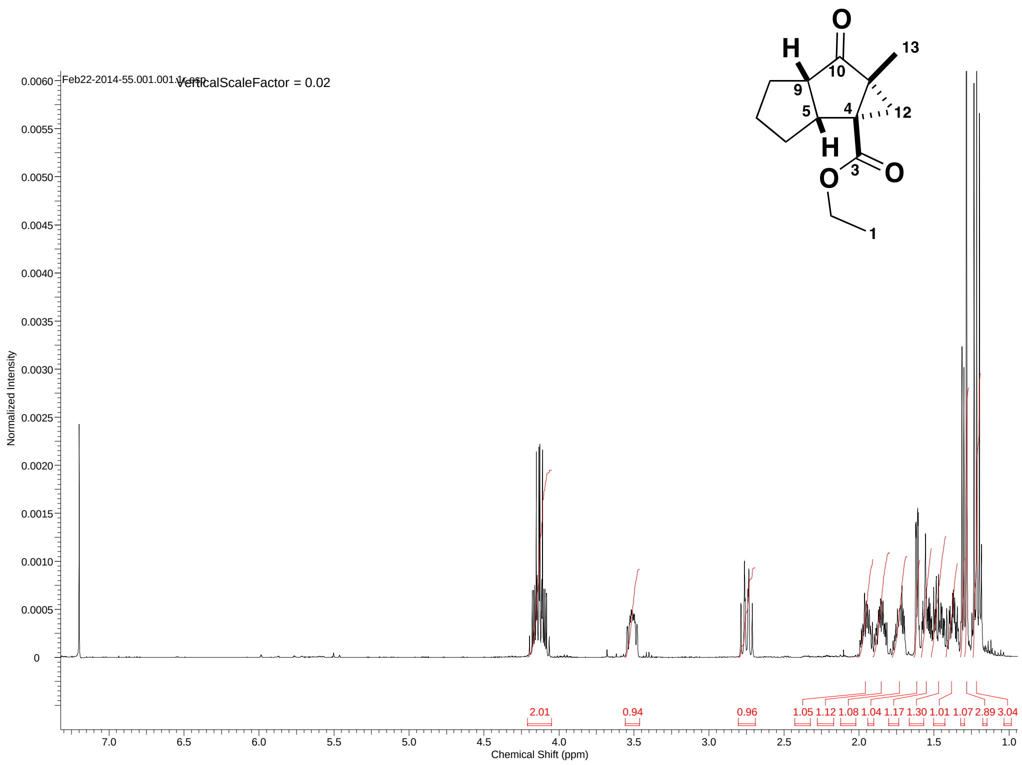
Feb22-2014-55.001.001.1r.esp
VerticalScaleFactor = 0.02
Normalized Intensity
Chemical Shift (ppm)
2.01
0.94
0.96
1.05 1.12 1.08 1.04 1.17 1.30 1.01 1.07 2.89 3.04
O
H
10
13
9
5
4
12
H
3
O
O
1

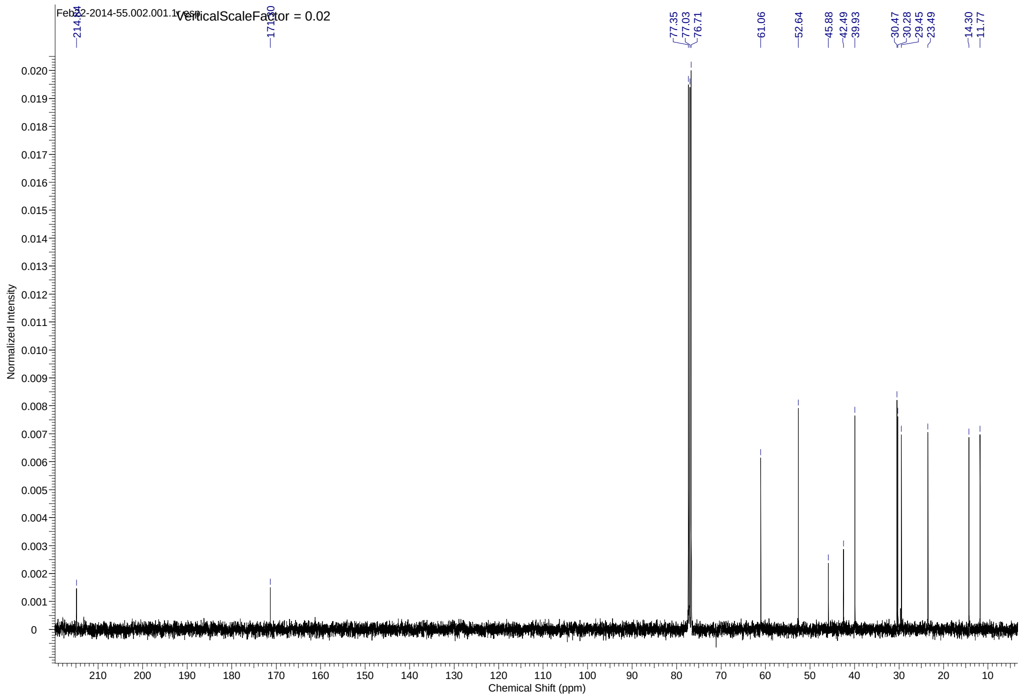
Feb22-2014-55.002.001.1r.esp
VerticalScaleFactor = 0.02
214.84
171.30
77.35
77.03
76.71
61.06
52.64
45.88
42.49
39.93
30.47
30.28
29.45
23.49
14.30
11.77
Normalized Intensity
Chemical Shift (ppm)

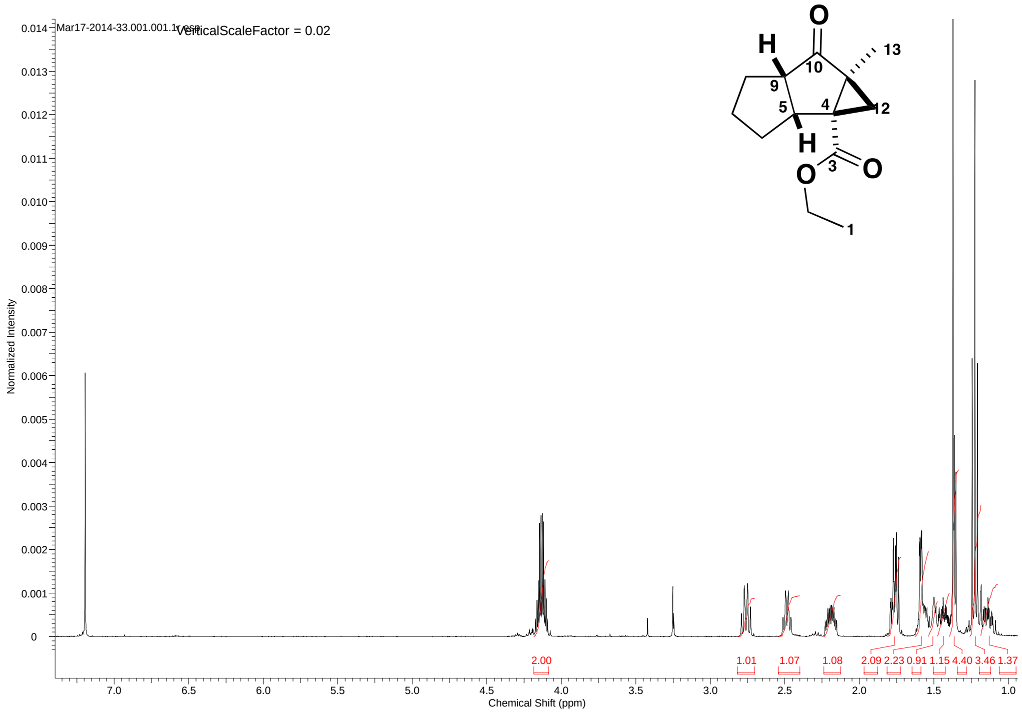

Mar17-2014-33.001.001.1r.esp
VerticalScaleFactor = 0.02
Normalized Intensity
Chemical Shift (ppm)
2.00
1.01
1.07
1.08
2.09
2.23
0.91
1.15
4.40
3.46
1.37

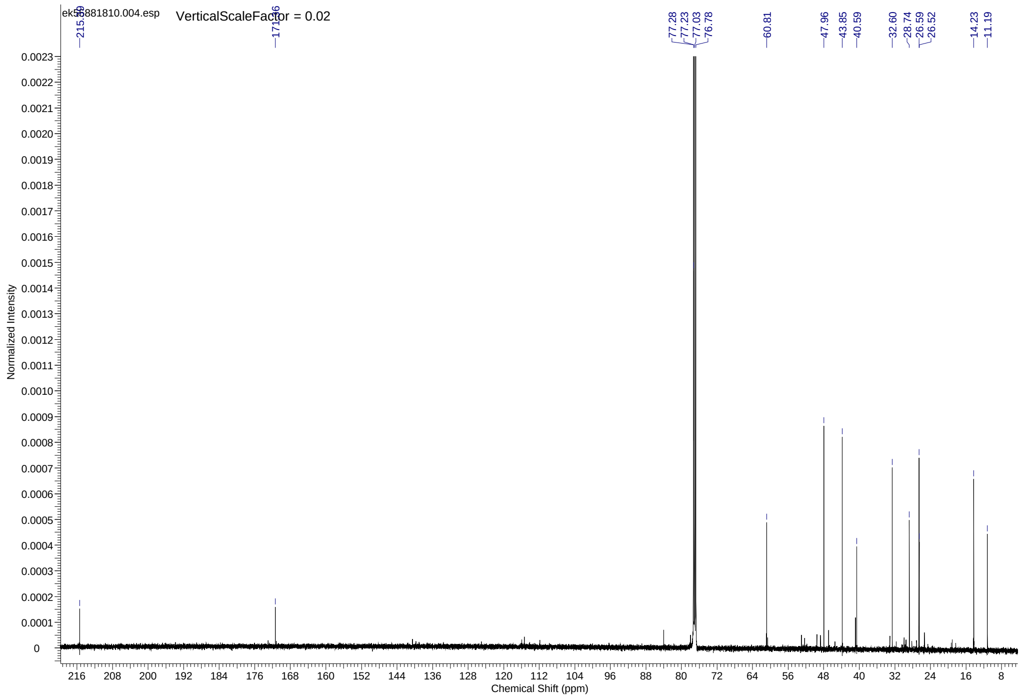

VerticalScaleFactor = 0.02
77.28
77.23
77.03
76.78
60.81
47.96
43.85
40.59
32.60
28.74
26.59
26.52
14.23
11.19
Normalized Intensity
Chemical Shift (ppm)

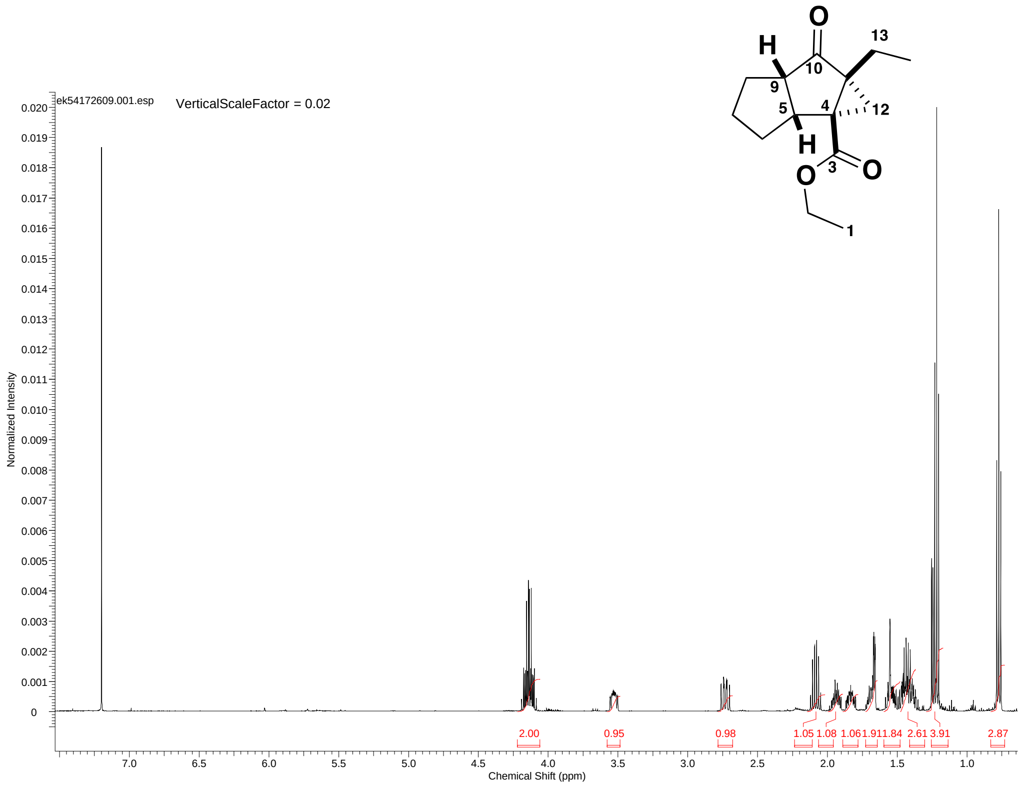
H
O
13
10
9
5
4
12
H
3
O
O
1
ek54172609.001.esp
VerticalScaleFactor = 0.02
Normalized Intensity
Chemical Shift (ppm)
2.00
0.95
0.98
1.05
1.08
1.06
1.91
1.84
2.61
3.91
2.87

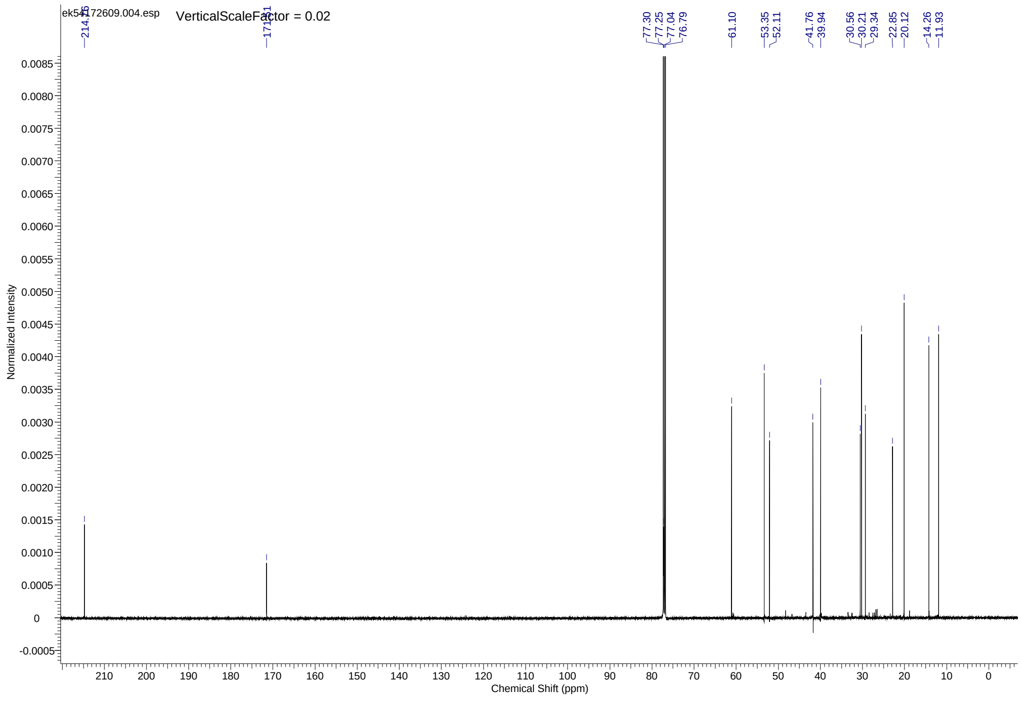
ek54172609.004.esp
VerticalScaleFactor = 0.02
214.45
171.41
77.30
77.25
77.04
76.79
61.10
53.35
52.11
41.76
39.94
30.56
30.21
29.34
22.85
20.12
14.26
11.93
Normalized Intensity
Chemical Shift (ppm)

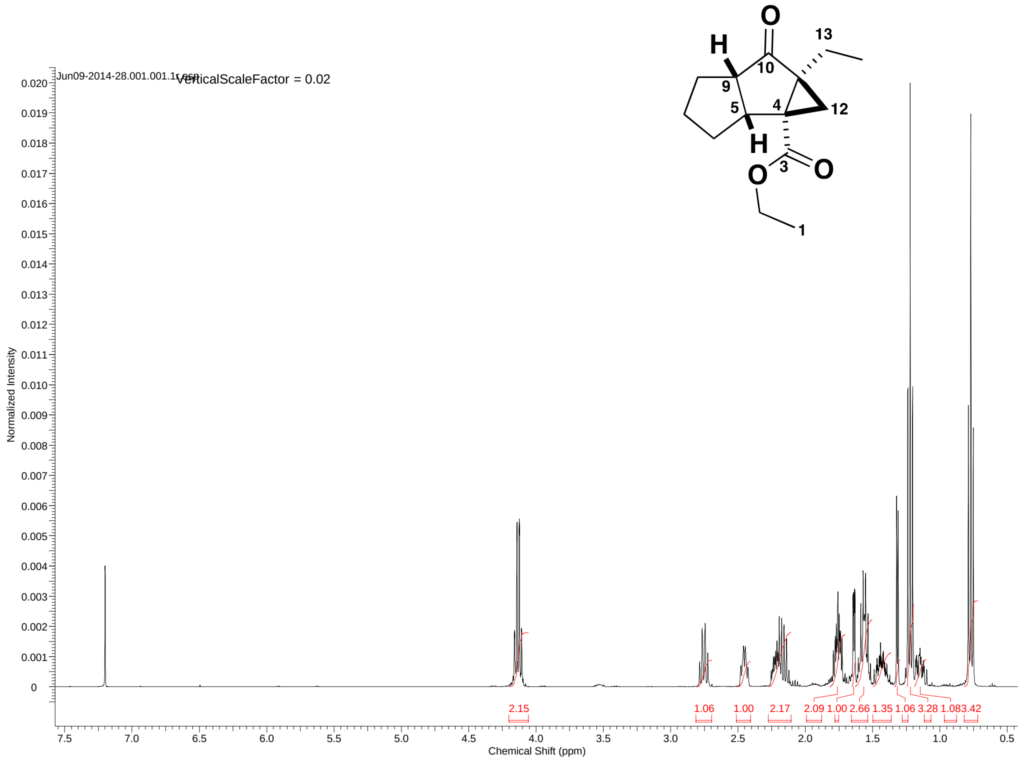
Jun09-2014-28.001.001.1r.esp
VerticalScaleFactor = 0.02
Normalized Intensity
Chemical Shift (ppm)
2.15
1.06
1.00
2.17
2.09
1.00
2.66
1.35
1.06
3.28
1.08
3.42
13
10
9
5
4
12
3
1
H
H
O
O
O

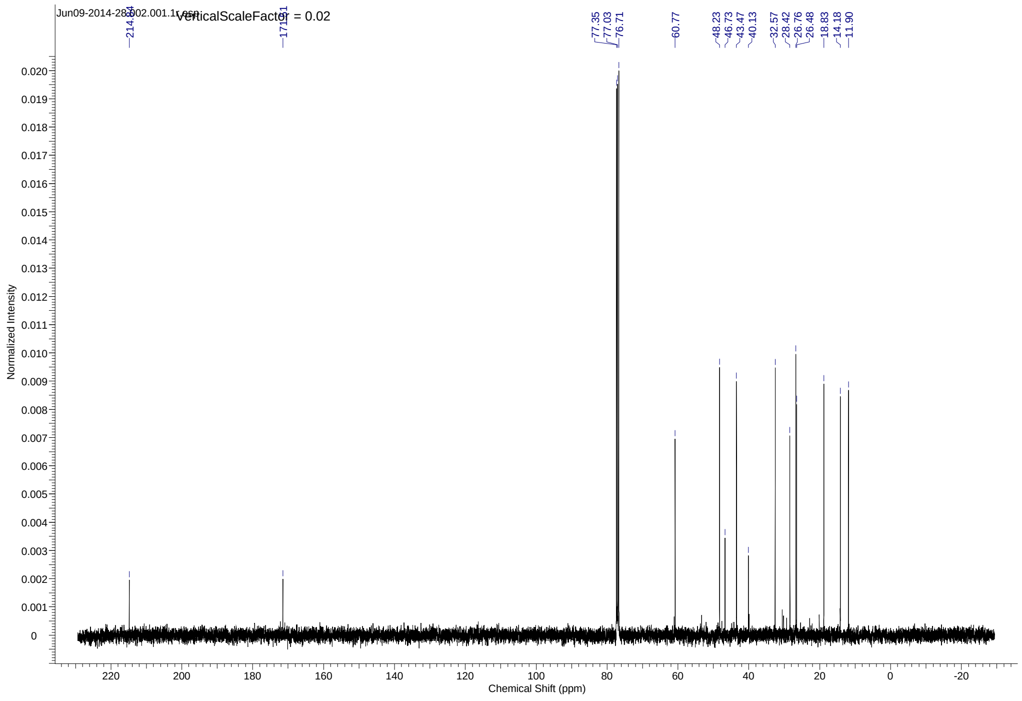
Jun09-2014-28.002.001.1r.esp
VerticalScaleFactor = 0.02
214.84
171.61
77.35
77.03
76.71
60.77
48.23
46.73
43.47
40.13
32.57
28.42
26.76
26.48
18.83
14.18
11.90
Normalized Intensity
Chemical Shift (ppm)

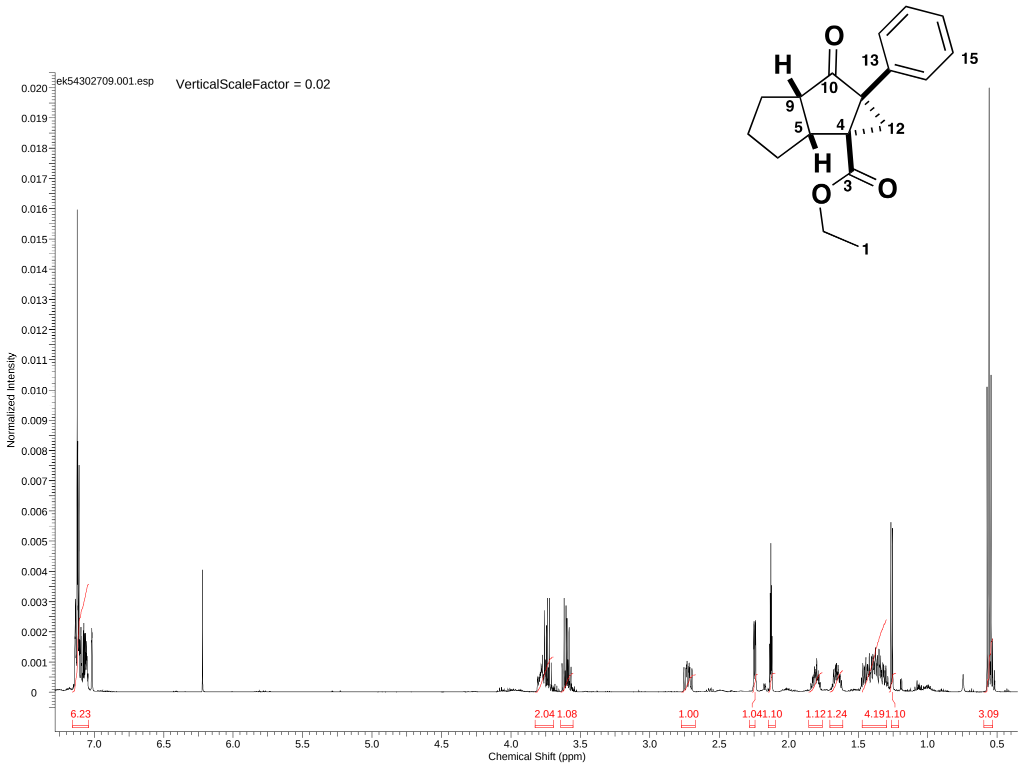
ek54302709.001.esp
VerticalScaleFactor = 0.02
Normalized Intensity
Chemical Shift (ppm)
6.23
2.04 1.08
1.00
1.04 1.10
1.12 1.24
4.19 1.10
3.09
O
H
13
15
10
9
5
4
12
H
3
O
O
1

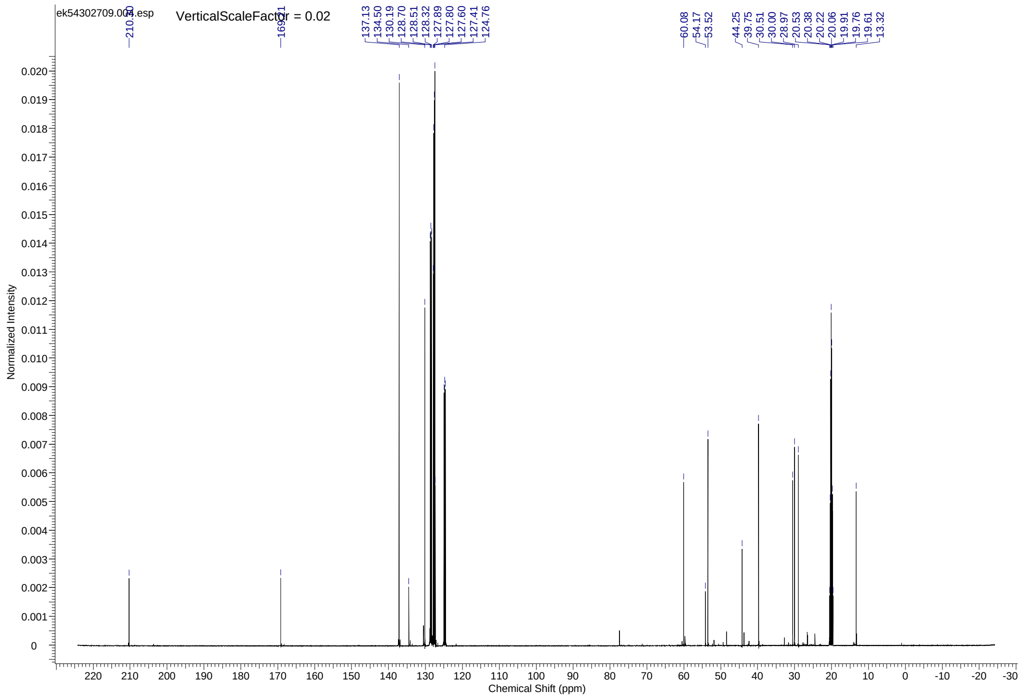
ek54302709.004.esp
VerticalScaleFactor = 0.02
210.30
169.51
137.13
134.50
130.19
128.70
128.51
128.32
127.89
127.80
127.60
127.41
124.76
60.08
54.17
53.52
44.25
39.75
30.51
30.00
28.97
20.53
20.38
20.22
20.06
19.91
19.76
19.61
13.32
Normalized Intensity
Chemical Shift (ppm)

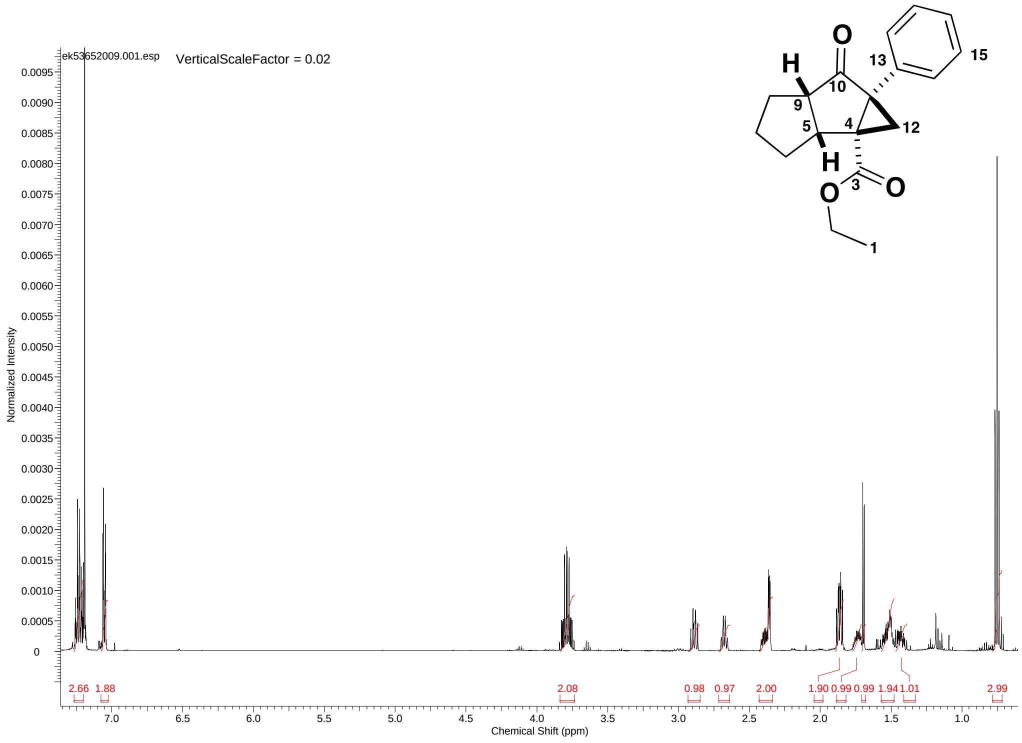
ek53652009.001.esp
VerticalScaleFactor = 0.02
Normalized Intensity
Chemical Shift (ppm)
2.66 1.88 2.08 0.98 0.97 2.00 1.90 0.99 0.99 1.94 1.01 2.99
H
O
10
9
5
4
13
15
12
H
3
O
O
1

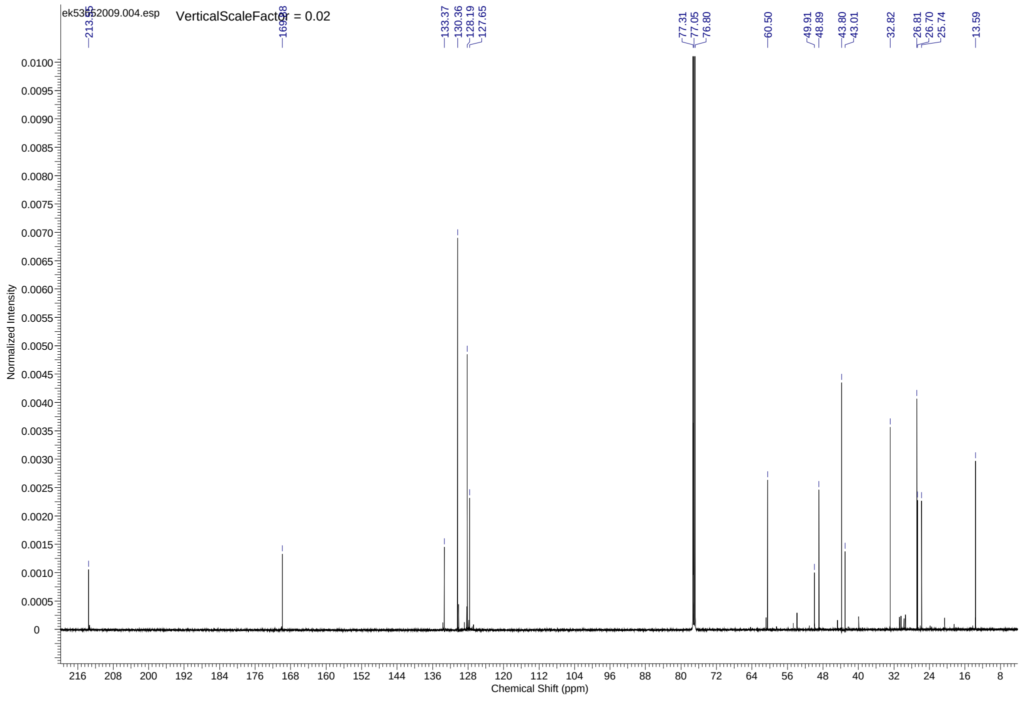
ek53652009.004.esp
VerticalScaleFactor = 0.02
Normalized Intensity
Chemical Shift (ppm)
213.55
169.98
133.37
130.36
128.19
127.65
77.31
77.05
76.80
60.50
49.91
48.89
43.80
43.01
32.82
26.81
26.70
25.74
13.59

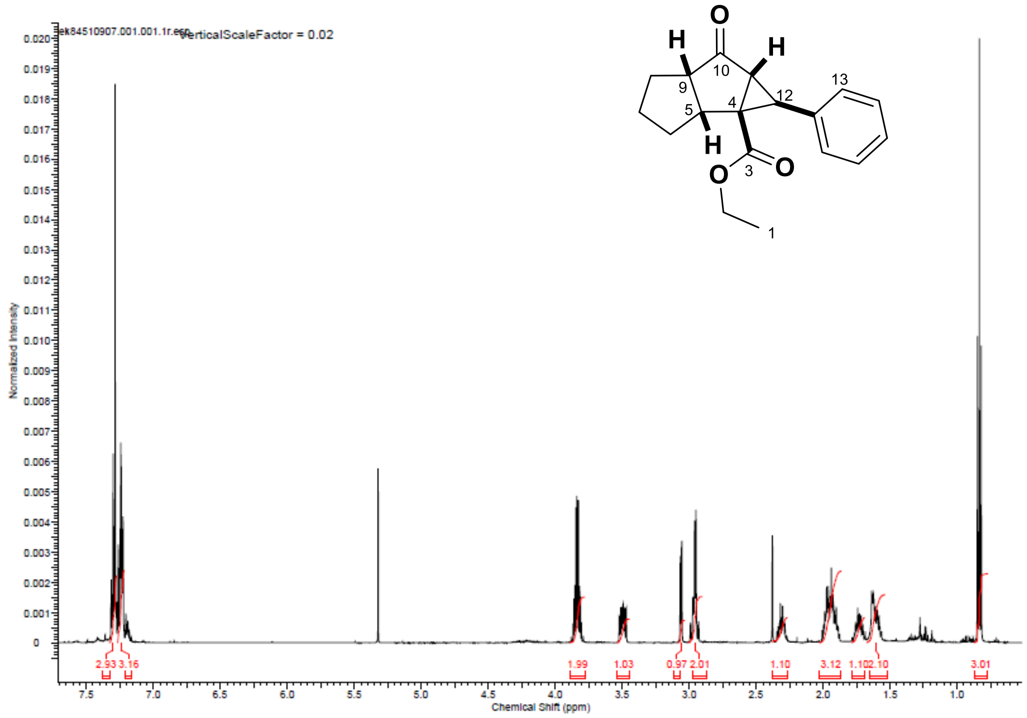
ek84510907.001.001.1r.esp
verticalScaleFactor = 0.02
Normalized Intensity
Chemical Shift (ppm)
2.93
3.16
1.99
1.03
0.97
2.01
1.10
3.12
1.10
2.10
3.01
O
H
H
H
10
9
5
4
12
13
3
O
O
1

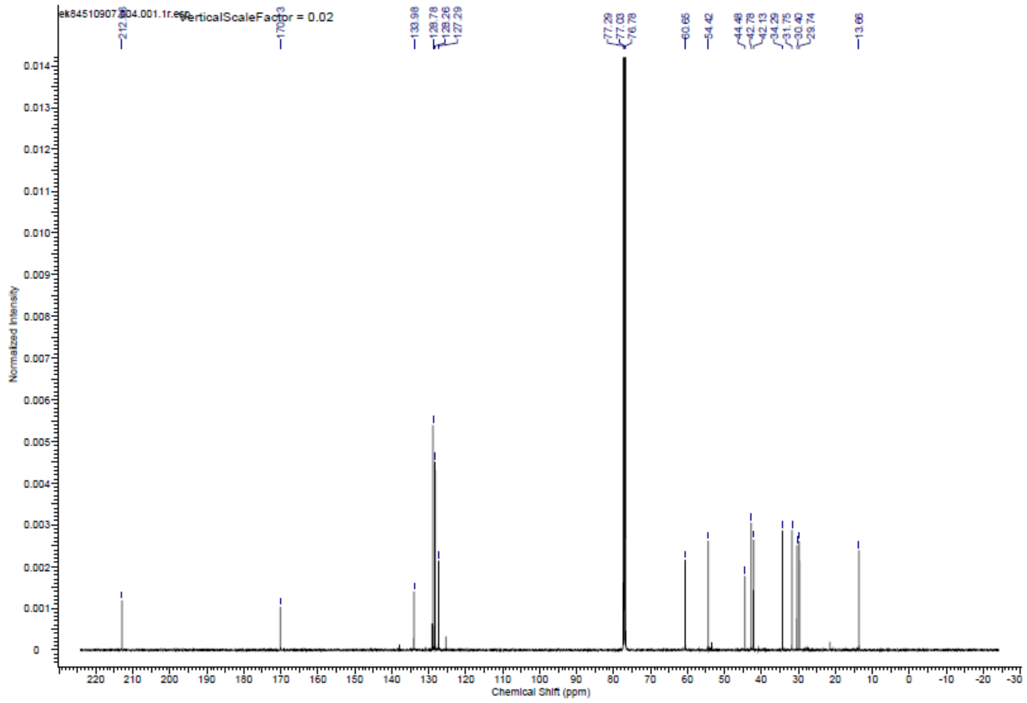
verticalScaleFactor = 0.02
133.98
128.78
128.26
127.29
77.29
77.03
76.78
60.65
54.42
44.48
42.78
42.13
34.29
31.75
30.40
29.74
13.66
Normalized Intensity
Chemical Shift (ppm)

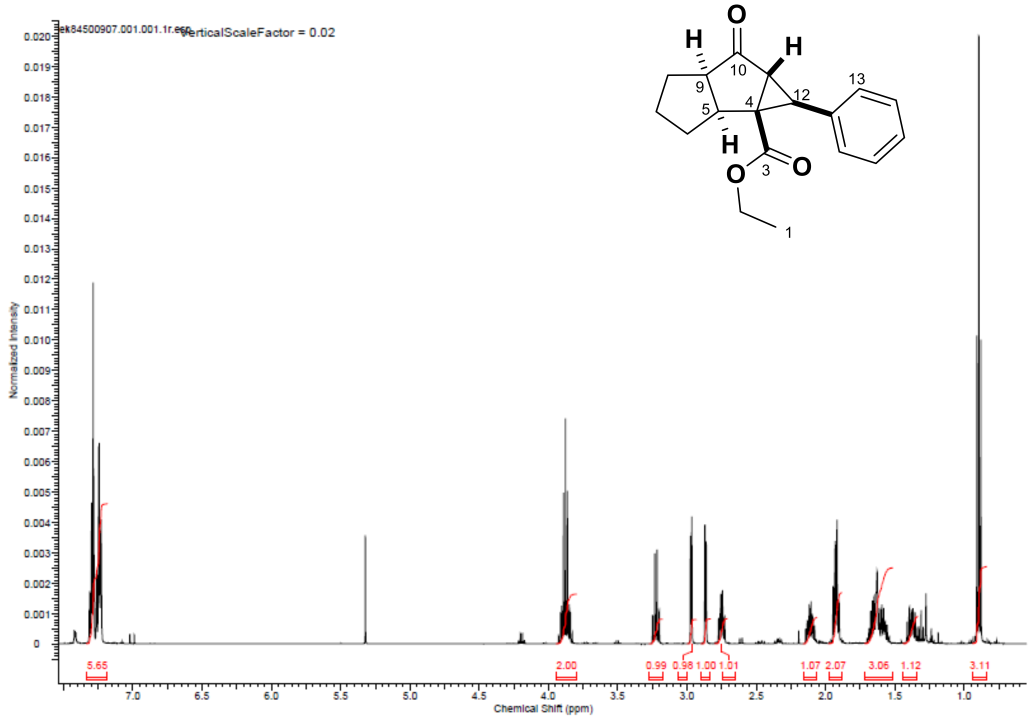
ek84500907.001.001.1r.esp
verticalScaleFactor = 0.02
Normalized Intensity
Chemical Shift (ppm)
5.65
2.00
0.99
0.98
1.00
1.01
1.07
2.07
3.06
1.12
3.11

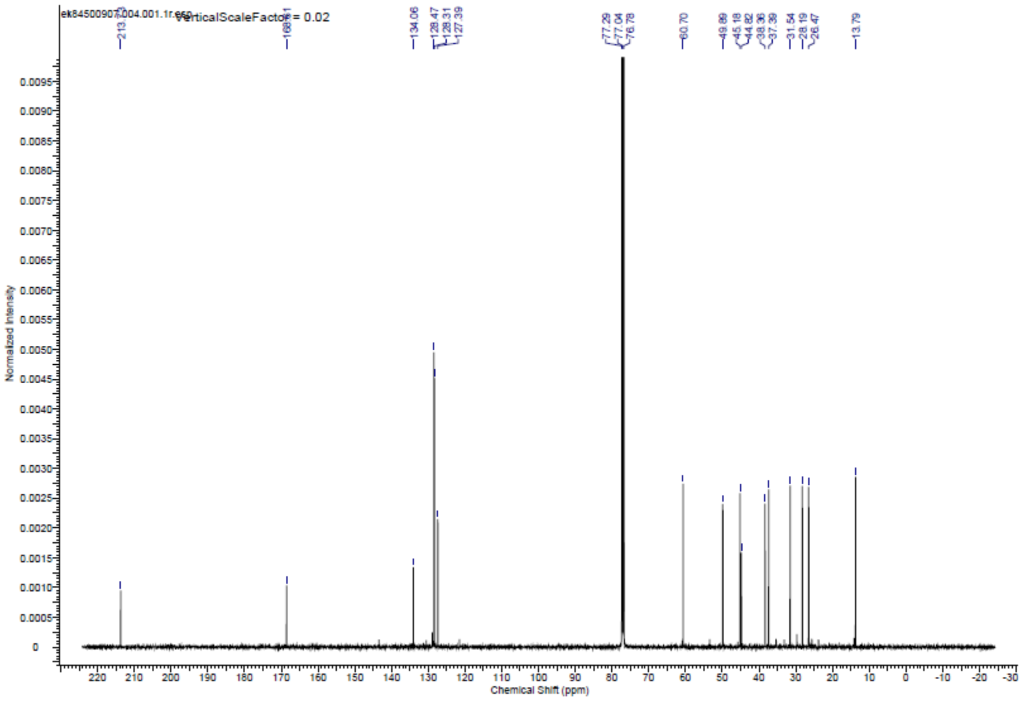
ek84500907.004.001.1r.esp
verticalScaleFactor = 0.02
134.06
128.47
128.31
127.39
77.29
77.04
76.78
60.70
49.89
45.18
44.82
38.36
37.39
31.54
28.19
26.47
13.79
Normalized Intensity
Chemical Shift (ppm)

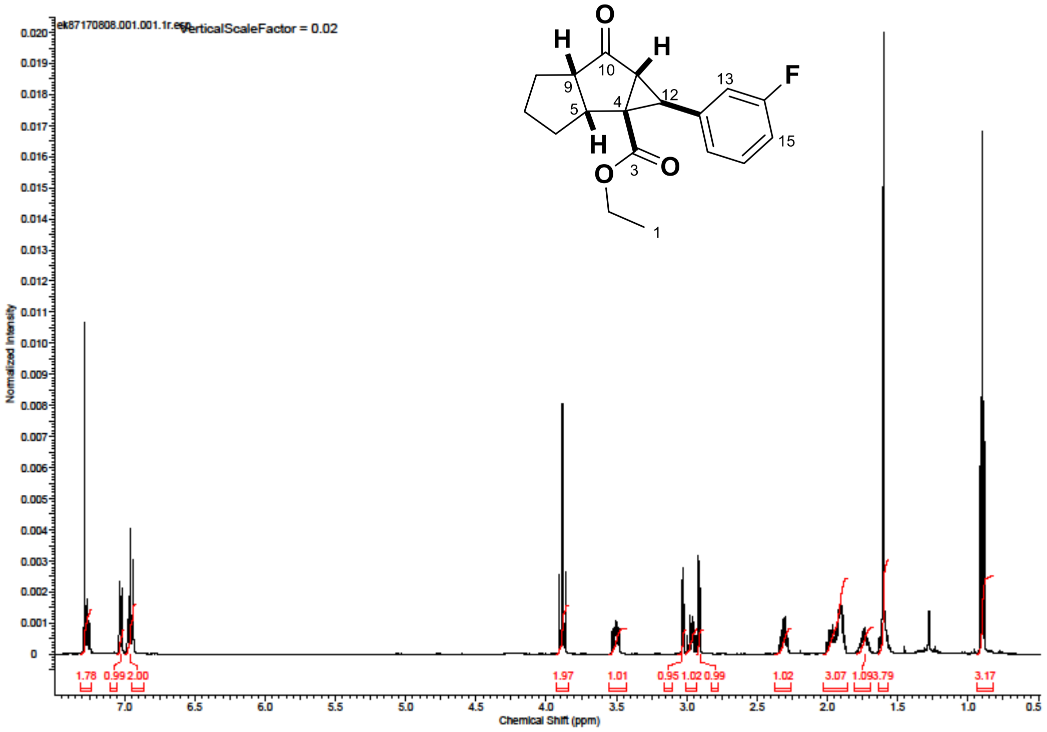
ek87170808.001.001.1r.esp
VerticalScaleFactor = 0.02
O
H
H
10
9
4
12
13
F
5
H
15
3
O
O
1
1.78
0.99
2.00
1.97
1.01
0.95
1.02
0.99
1.02
3.07
1.09
3.79
3.17
Chemical Shift (ppm)
Normalized Intensity

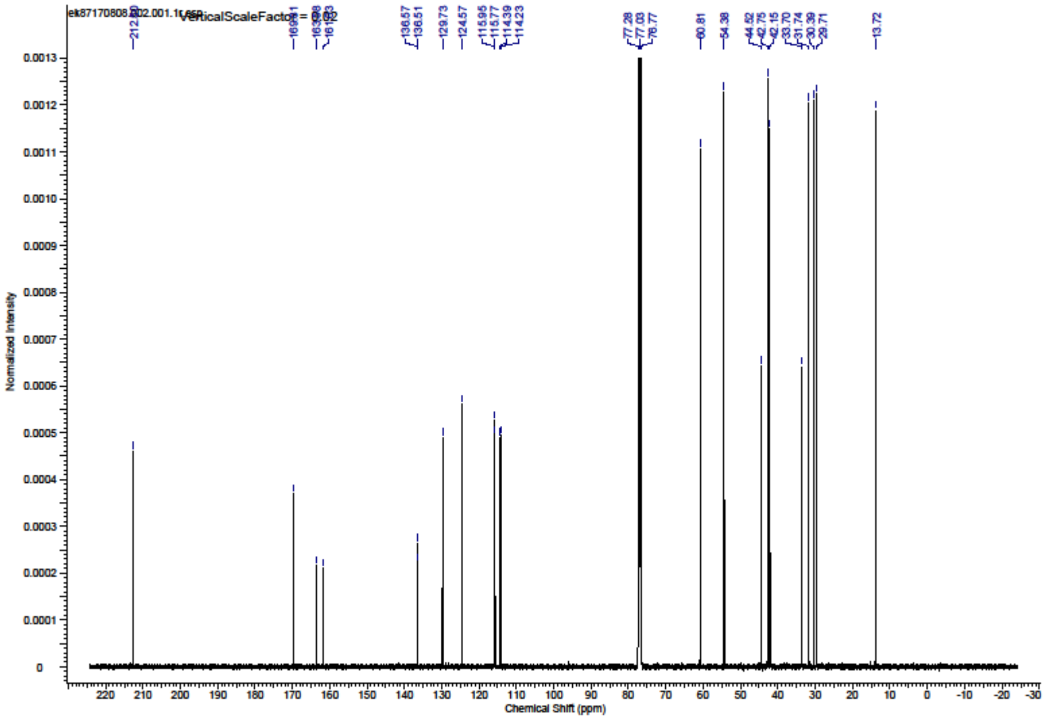
VerticalScaleFactor
212.80
169.81
136.57
136.51
129.73
124.57
115.95
115.77
114.39
114.23
77.28
77.03
76.77
60.81
54.38
44.52
42.75
42.15
33.70
31.74
30.39
29.71
13.72
Chemical Shift (ppm)
Normalized Intensity

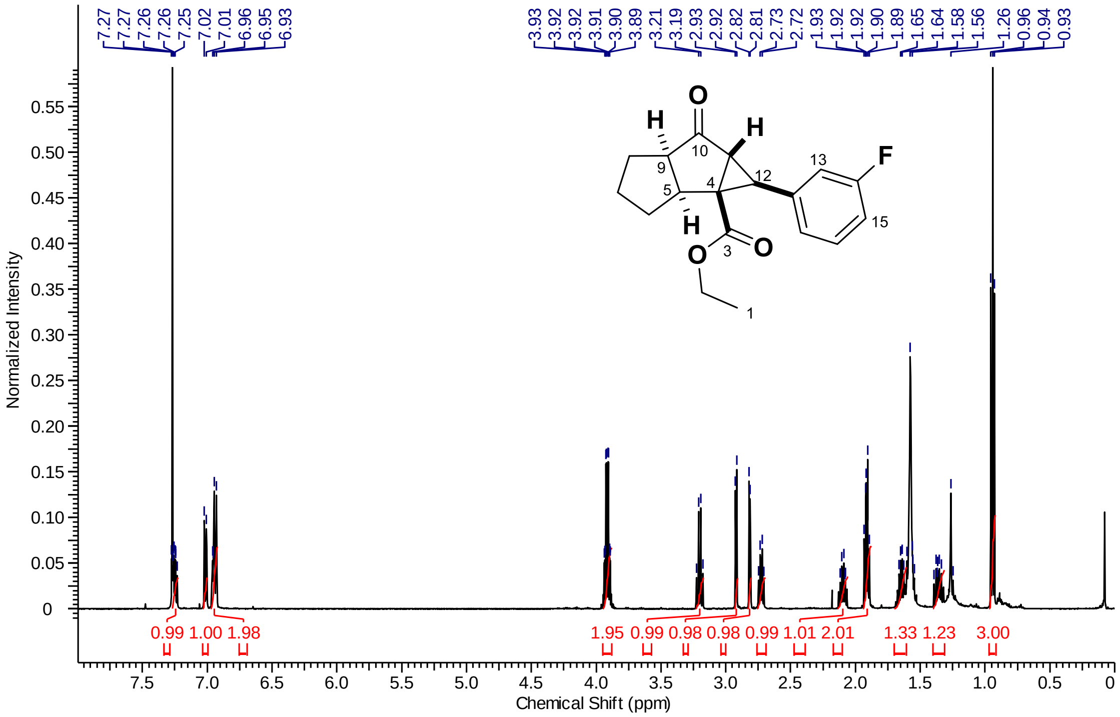

7.27 7.27 7.26 7.26 7.25 7.02 7.01 6.96 6.95 6.93
3.93 3.92 3.92 3.91 3.90 3.89 3.21 3.19 2.93 2.92 2.82 2.81 2.73 2.72 1.93 1.92 1.92 1.90 1.89 1.65 1.64 1.58 1.56 1.26 0.96 0.94 0.93
0.99 1.00 1.98 1.95 0.99 0.98 0.98 0.99 1.01 2.01 1.33 1.23 3.00
Normalized Intensity
Chemical Shift (ppm)
O
H
H
H
F
10
9
5
4
12
13
15
3
O
O
1

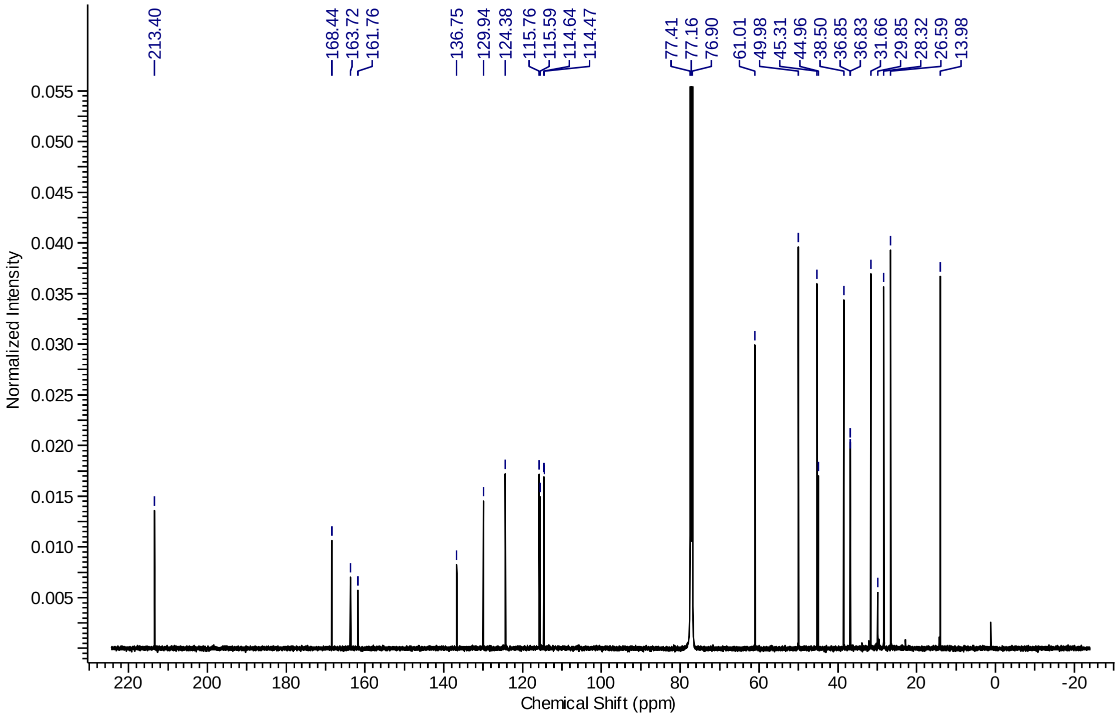

213.40
168.44 163.72 161.76
136.75 129.94 124.38 115.76 115.59 114.64 114.47
77.41 77.16 76.90
61.01 49.98 45.31 44.96 38.50 36.85 36.83 31.66 29.85 28.32 26.59 13.98
Normalized Intensity
Chemical Shift (ppm)

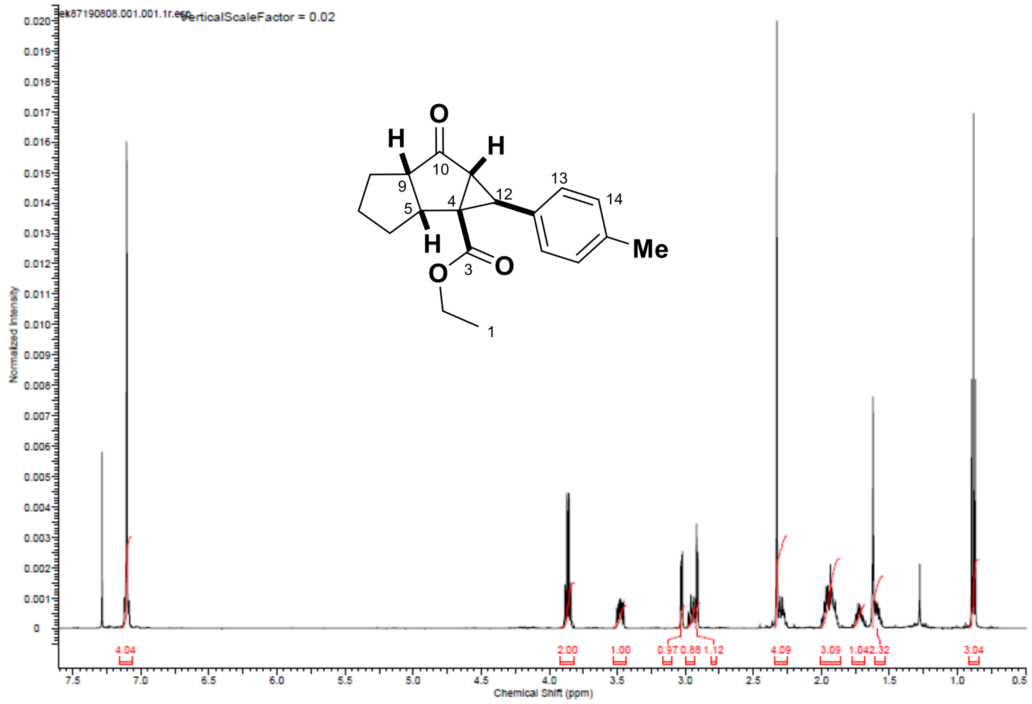
ek87190808.001.001.1r.esp
verticalScaleFactor = 0.02
Normalized Intensity
Chemical Shift (ppm)
4.04
2.00
1.00
0.97
0.88
1.12
4.09
3.09
1.04
2.32
3.04
Me

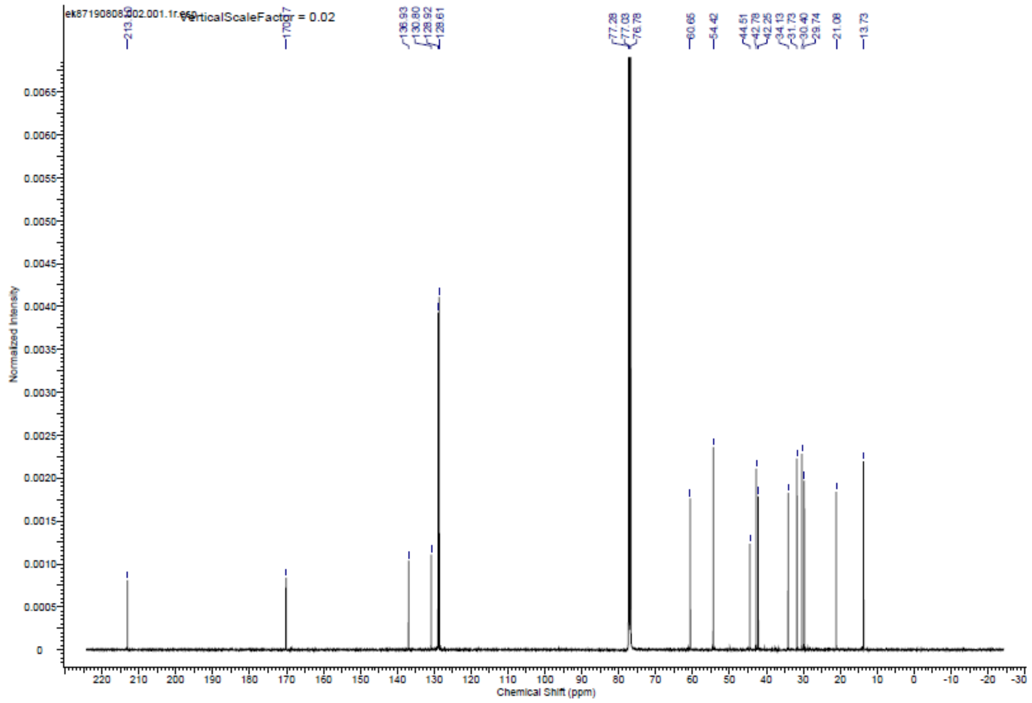
ek87190808.002.001.1r.esp
verticalScaleFactor = 0.02
Normalized Intensity
Chemical Shift (ppm)
170.77
136.93
130.80
128.92
128.61
77.28
77.03
76.78
60.65
54.42
44.51
42.78
42.25
34.13
31.73
30.40
29.74
21.08
13.73

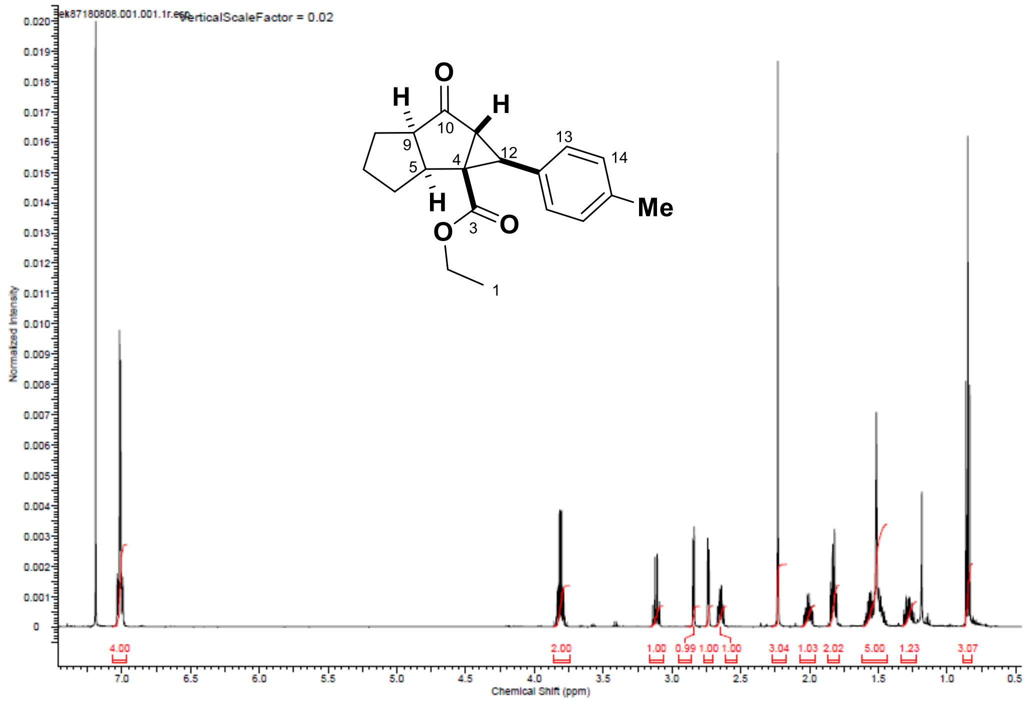
ek87180808.001.001.1r.esp
verticalScaleFactor = 0.02
Normalized Intensity
Chemical Shift (ppm)
4.00
2.00
1.00
0.99
1.00
1.00
3.04
1.03
2.02
5.00
1.23
3.07
O
H
H
H
10
9
5
4
12
13
14
Me
3
O
O
1

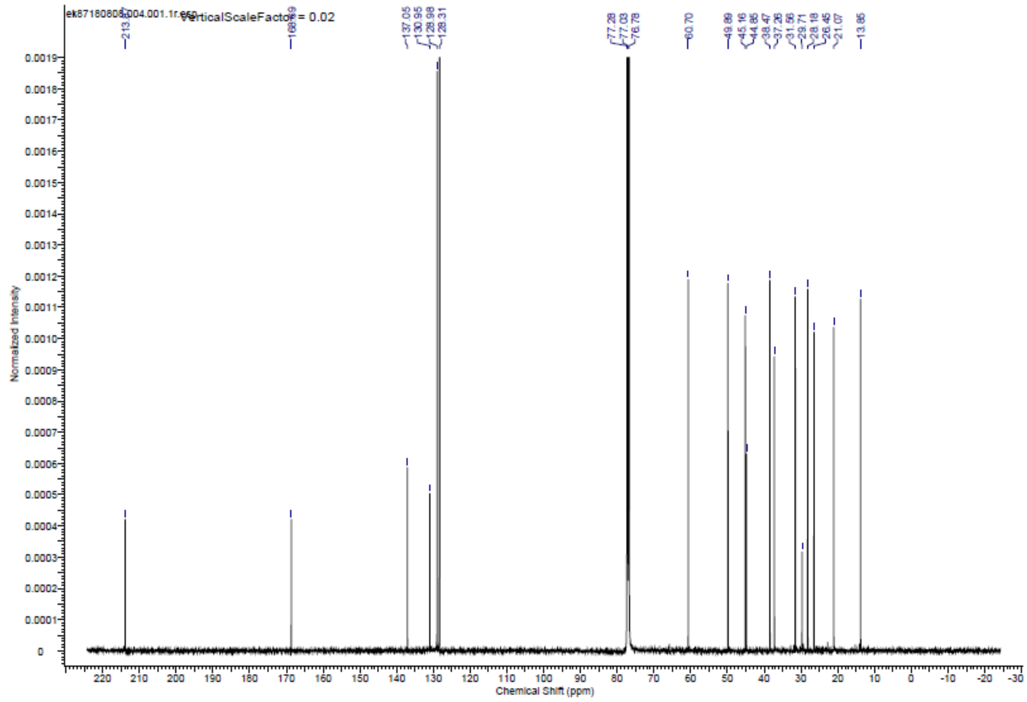
ek87180808.004.001.1r.esp
verticalScaleFactor = 0.02
Normalized Intensity
Chemical Shift (ppm)
168.89
137.05
130.95
128.98
128.31
77.28
77.03
76.78
60.70
49.89
45.16
44.85
38.47
37.26
31.56
29.71
28.18
26.45
21.07
13.85

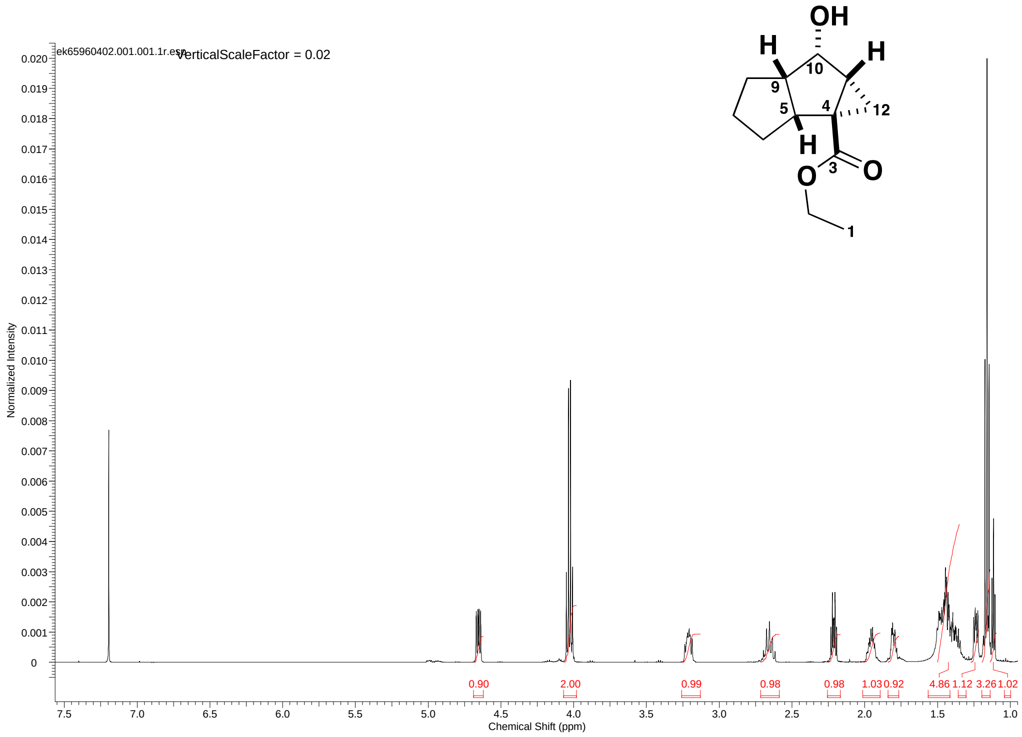
ek65960402.001.001.1r.esp
VerticalScaleFactor = 0.02
Normalized Intensity
Chemical Shift (ppm)
0.90
2.00
0.99
0.98
0.98
1.03
0.92
4.86
1.12
3.26
1.02
OH
H
H
H
10
9
5
4
12
3
O
O
1

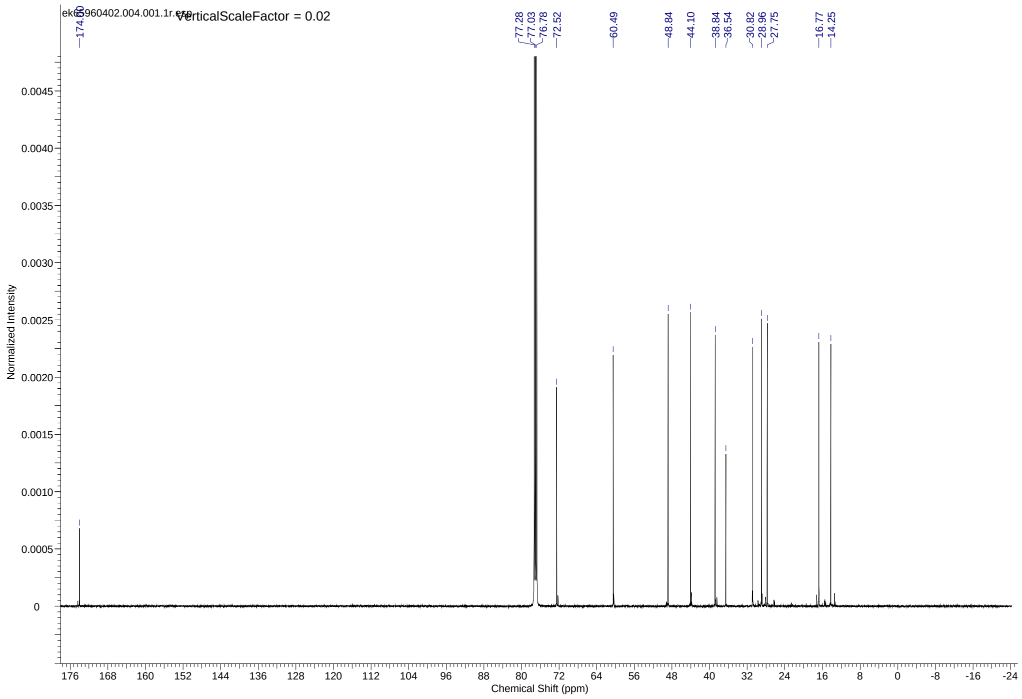
ek65960402.004.001.1r.esp
VerticalScaleFactor = 0.02
Normalized Intensity
Chemical Shift (ppm)
174.00
77.28
77.03
76.78
72.52
60.49
48.84
44.10
38.84
36.54
30.82
28.96
27.75
16.77
14.25

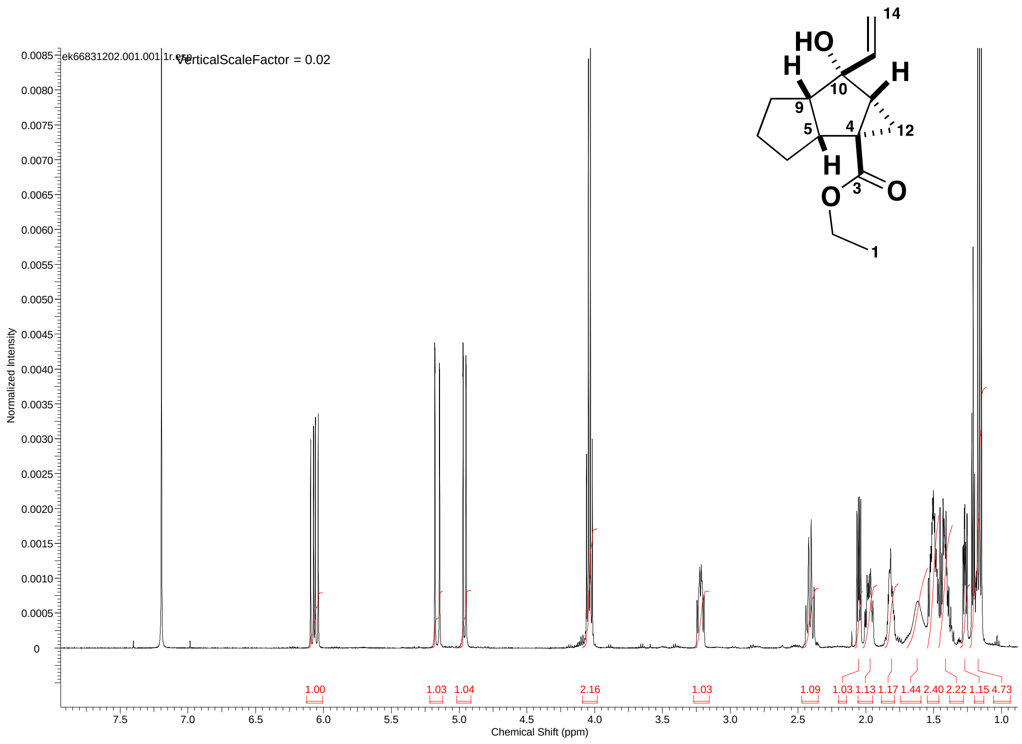
ek66831202.001.001.1r.esp
VerticalScaleFactor = 0.02
Normalized Intensity
Chemical Shift (ppm)
1.00
1.03
1.04
2.16
1.03
1.09
1.03
1.13
1.17
1.44
2.40
2.22
1.15
4.73
HO
H
H
H
O
O
14
10
9
5
4
12
3
1

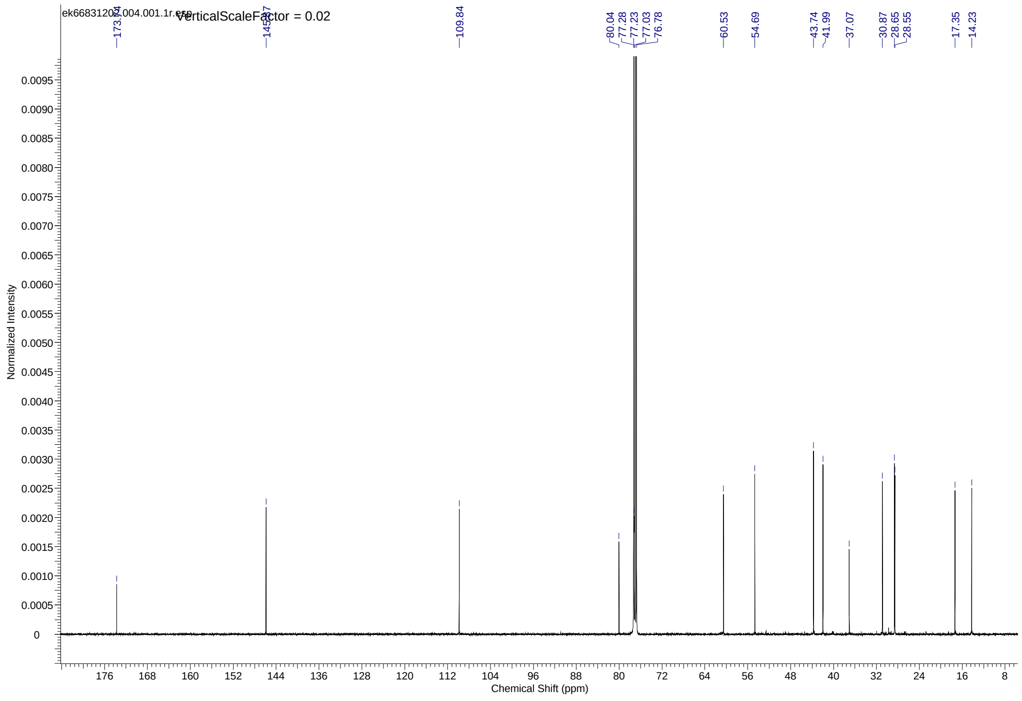
VerticalScaleFactor = 0.02
Normalized Intensity
Chemical Shift (ppm)
109.84
80.04
77.28
77.23
77.03
76.78
60.53
54.69
43.74
41.99
37.07
30.87
28.65
28.55
17.35
14.23

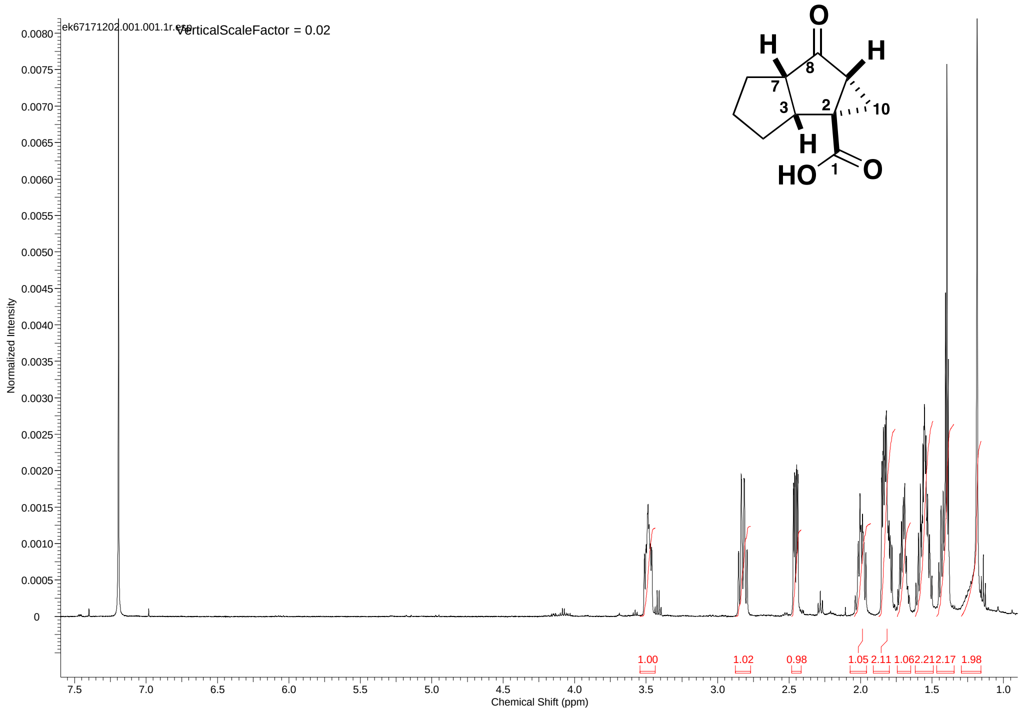
ek67171202.001.001.1r.esp
VerticalScaleFactor = 0.02
Normalized Intensity
Chemical Shift (ppm)
1.00
1.02
0.98
1.05
2.11
1.06
2.21
2.17
1.98
O
H
H
H
HO
O
8
7
3
2
10
1

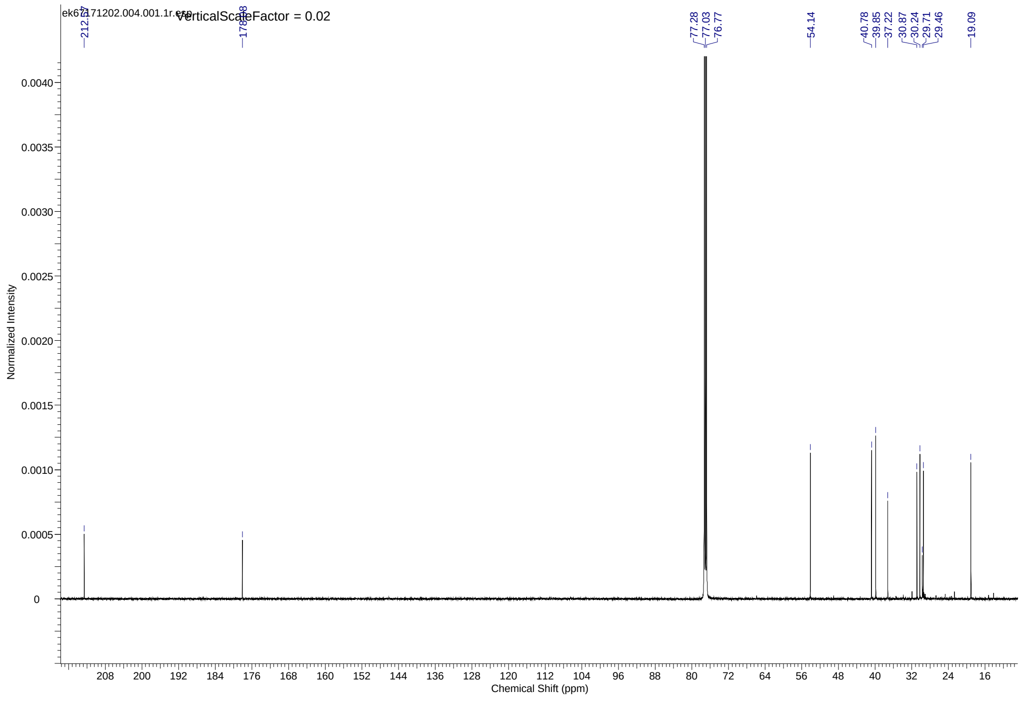
ek67171202.004.001.1r.esp
VerticalScaleFactor = 0.02
77.28
77.03
76.77
54.14
40.78
39.85
37.22
30.87
30.24
29.71
29.46
19.09
Normalized Intensity
Chemical Shift (ppm)

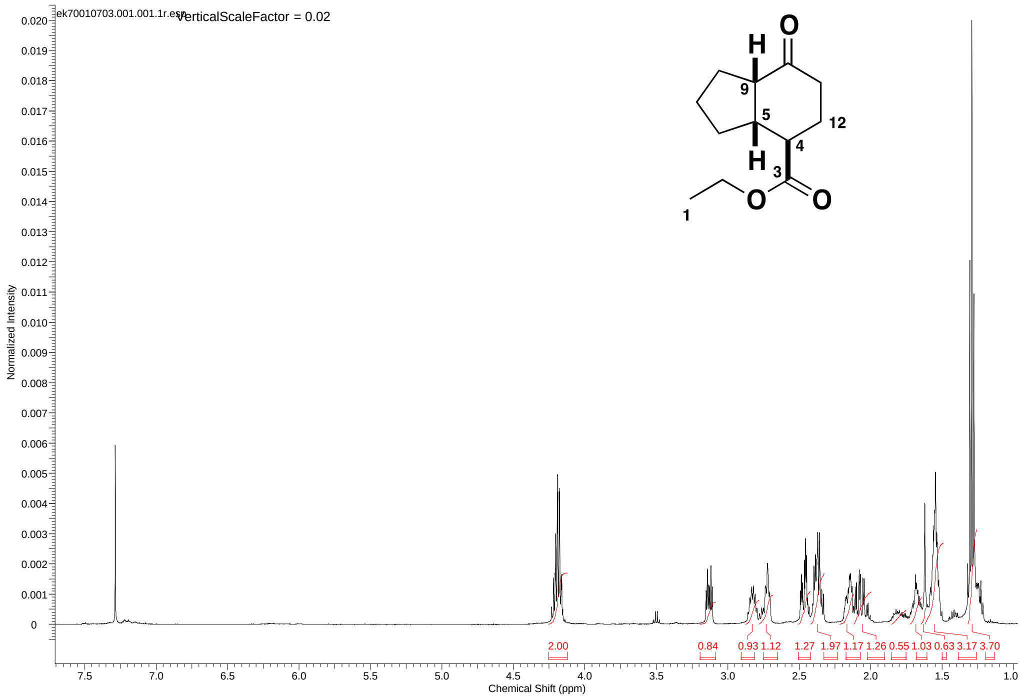
ek70010703.001.001.1r.esp
VerticalScaleFactor = 0.02
Normalized Intensity
Chemical Shift (ppm)
2.00
0.84
0.93
1.12
1.27
1.97
1.17
1.26
0.55
1.03
0.63
3.17
3.70
9
5
4
3
12
1

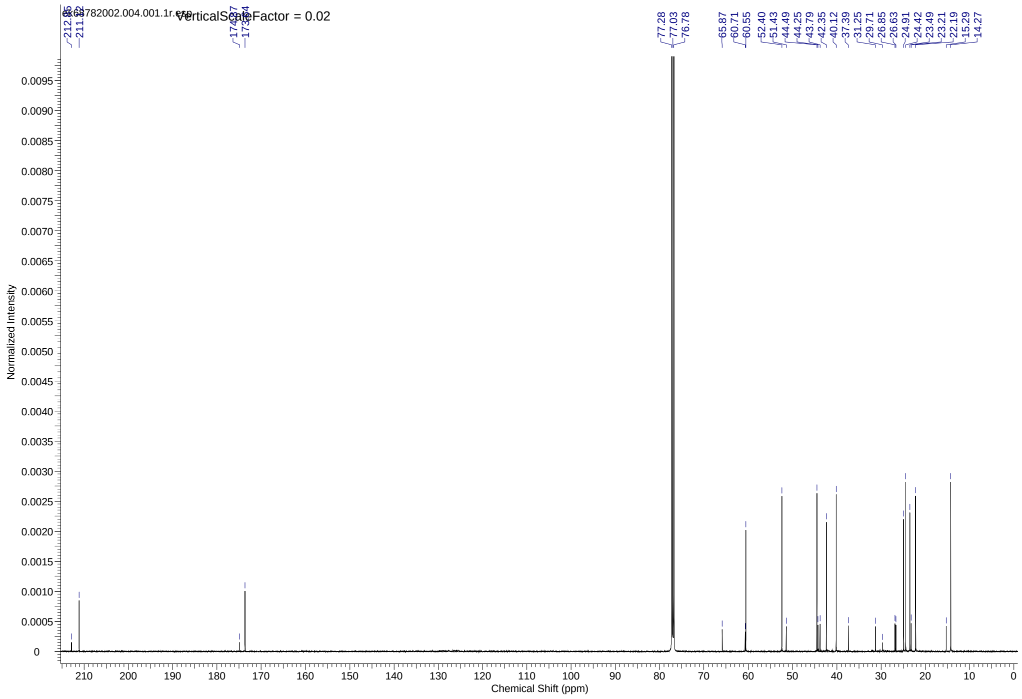
VerticalScaleFactor = 0.02
Normalized Intensity
Chemical Shift (ppm)
77.28
77.03
76.78
65.87
60.71
60.55
52.40
51.43
44.49
44.25
43.79
42.35
40.12
37.39
31.25
29.71
26.85
26.63
24.91
24.42
23.49
23.21
22.19
15.29
14.27

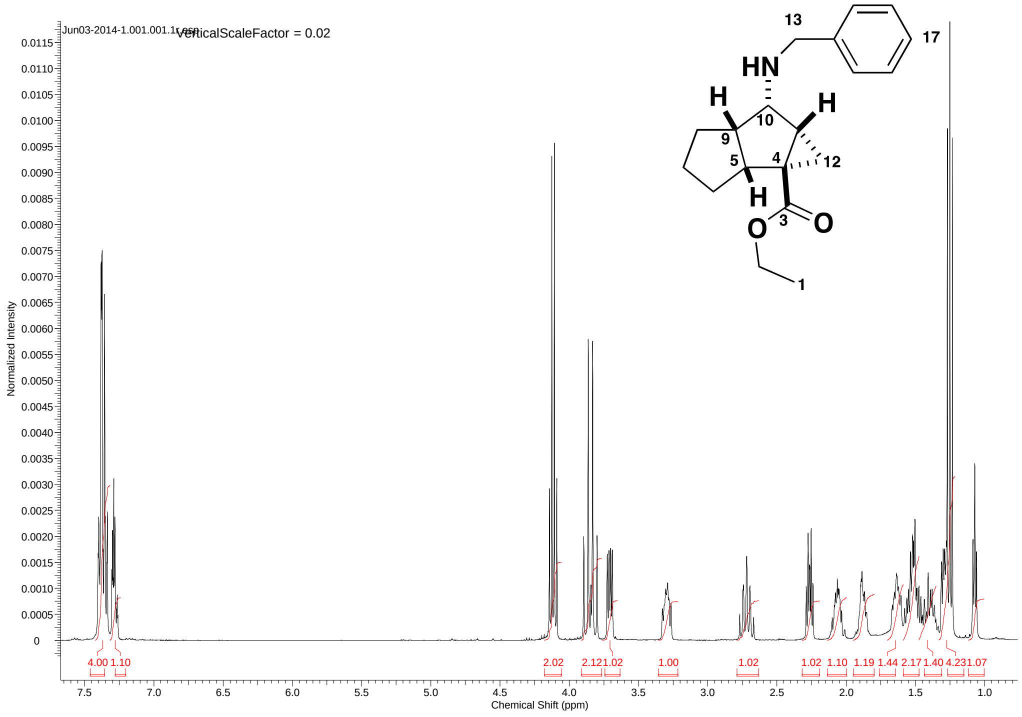

Jun03-2014-1.001.001.1r.esp
VerticalScaleFactor = 0.02
Normalized Intensity
Chemical Shift (ppm)
4.00
1.10
2.02
2.12
1.02
1.00
1.02
1.02
1.10
1.19
1.44
2.17
1.40
4.23
1.07
HN
H
H
H
13
17
10
9
5
4
12
3
O
O
1

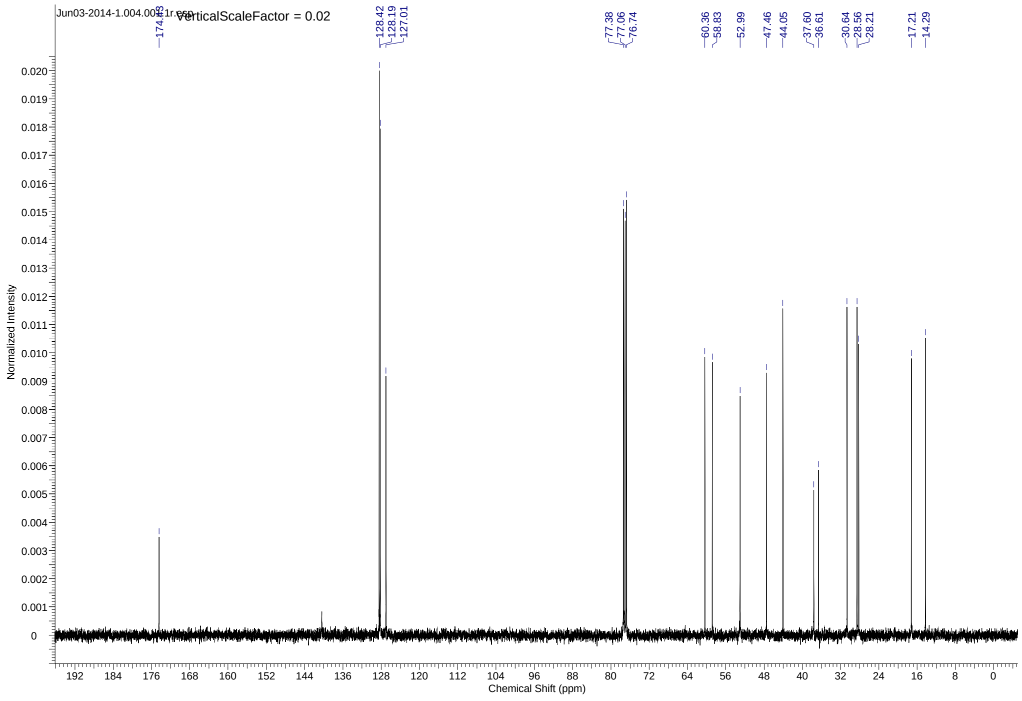

Jun03-2014-1.004.001.1r.esp
VerticalScaleFactor = 0.02
174.43
128.42
128.19
127.01
77.38
77.06
76.74
60.36
58.83
52.99
47.46
44.05
37.60
36.61
30.64
28.56
28.21
17.21
14.29
Normalized Intensity
Chemical Shift (ppm)

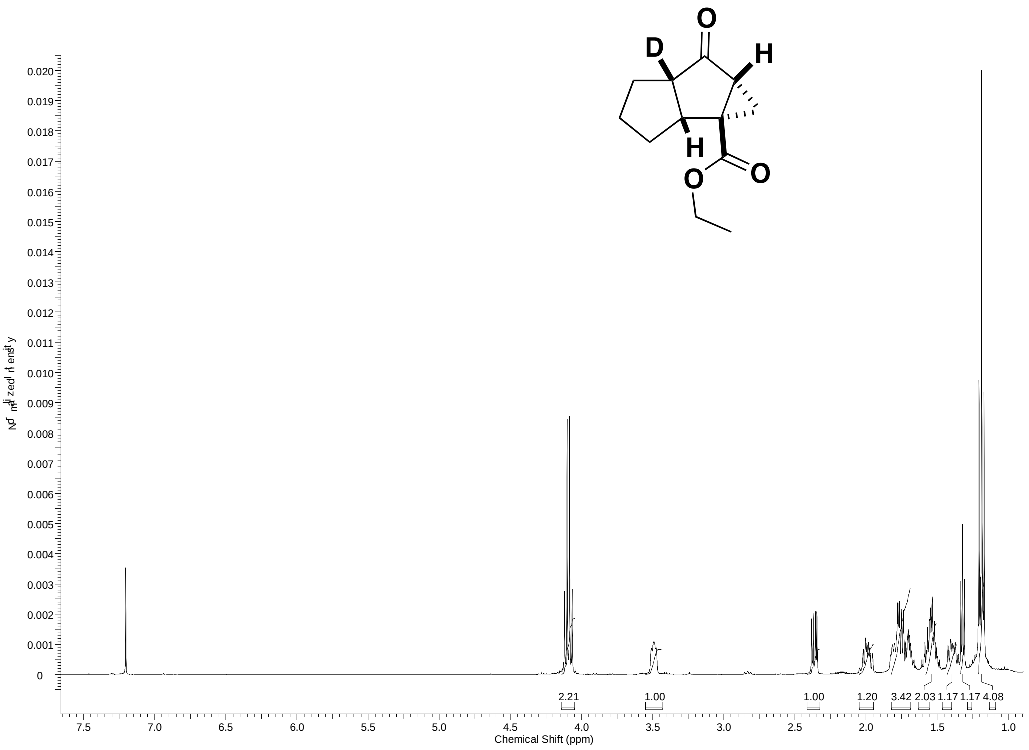
D
O
H
H
O
O
Chemical Shift (ppm)
2.21
1.00
1.00
1.20
3.42
2.03
1.17
1.17
4.08

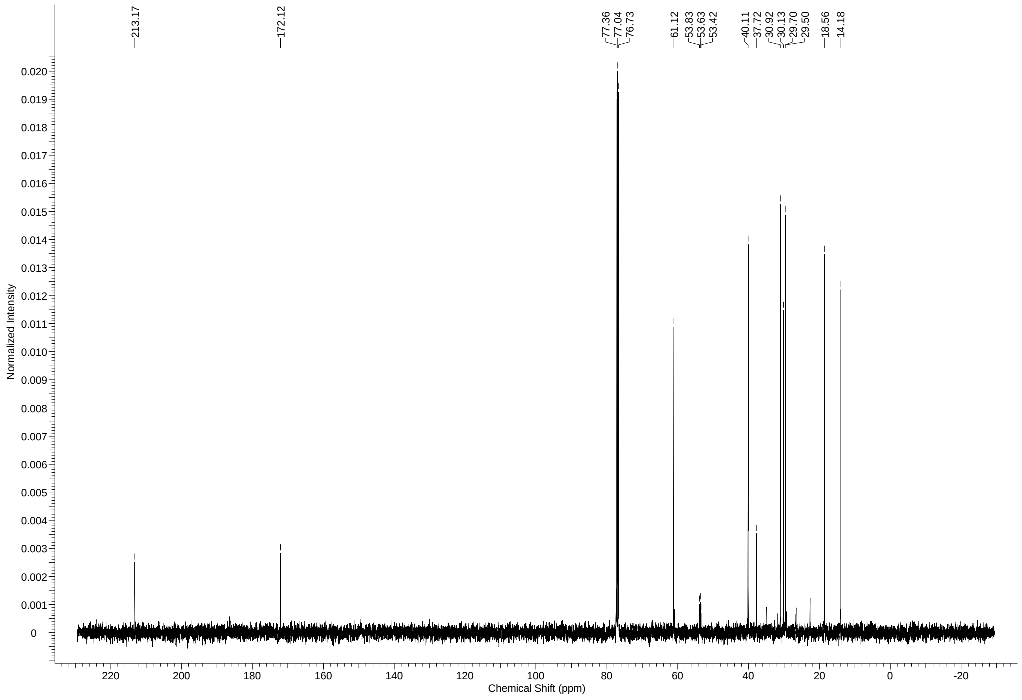
213.17
172.12
77.36
77.04
76.73
61.12
53.83
53.63
53.42
40.11
37.72
30.92
30.13
29.70
29.50
18.56
14.18
Normalized Intensity
Chemical Shift (ppm)

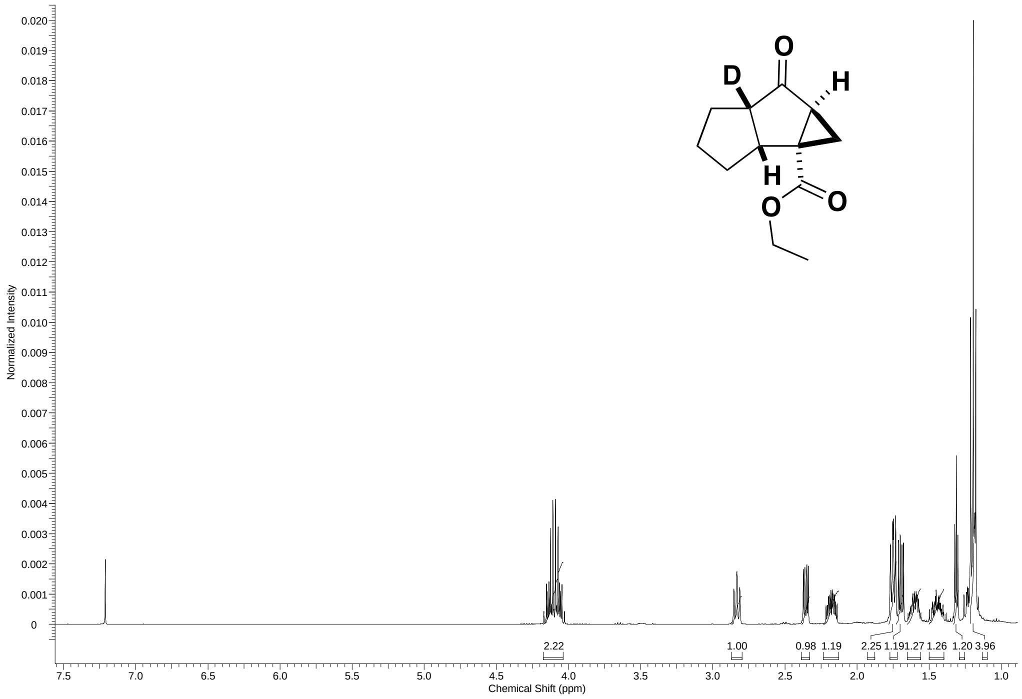
D
O
H
H
O
O
Normalized Intensity
2.22
1.00
0.98
1.19
2.25
1.19
1.27
1.26
1.20
3.96
Chemical Shift (ppm)

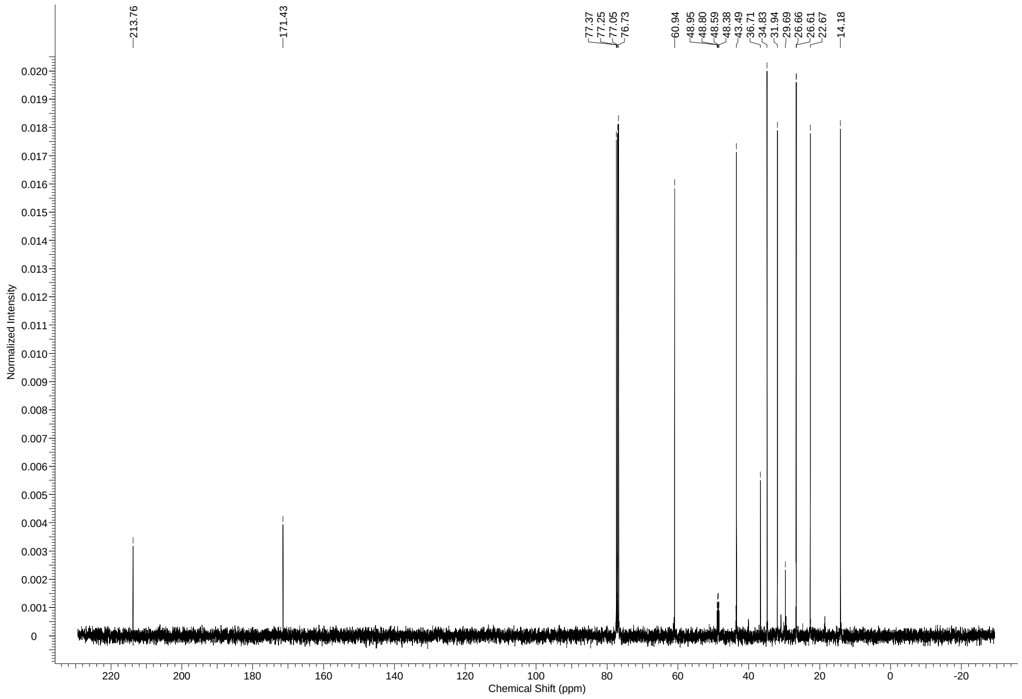
213.76
171.43
77.37
77.25
77.05
76.73
60.94
48.95
48.80
48.59
48.38
43.49
36.71
34.83
31.94
29.69
26.66
26.61
22.67
14.18
Normalized Intensity
Chemical Shift (ppm)
